# Lipid-Inspired Low Melting Ionic Liquids via Synergistic Cyclopropanation and Branching of Terpenoids

**DOI:** 10.1021/acsmaterialsau.5c00089

**Published:** 2025-07-21

**Authors:** Muhammadiqboli Musozoda, Richard A. O’Brien, Zachary J. Metott, Raychell A. Jerdo, Christopher M. Butch, Matthias Zeller, Gregory R. Boyce, Patrick C. Hillesheim, Arsalan Mirjafari

**Affiliations:** † Department of Chemistry, 14828 State University of New York at Oswego, Oswego, New York 13126, United States; ‡ Department of Chemistry, 5557 The University of South Alabama, Mobile, Alabama 36688, United States; § Department of Chemistry, 8522 Purdue University, West Lafayette, Indiana 47907, United States; ∥ Department of Chemistry and Biochemistry, 3087 East Stroudsburg University, East Stroudsburg, Pennsylvania 18301, United States; ⊥ Department of Chemistry, 6049 Illinois State University, Normal, Illinois 61761, United States

**Keywords:** ionic liquids, bioinspired materials, chiral materials, molecular engineering, lipid-like ionic liquids, structure−property relationship, cyclopropane

## Abstract

Bacteria employ cyclopropane motifs as bioisosteres for unsaturations to modulate lipid bilayer fluidity and protect cellular membranes under environmental stress. Drawing inspiration from this biological strategy, we investigated how cyclopropanation impacts the thermophysical properties of lipid-inspired ionic liquids. We synthesized a series of imidazolium-based ionic liquids incorporating cyclopropanated derivatives of three renewable terpenoids: phytol, farnesol, and geraniol. Through an integrated approach combining property-driven design, thermophysical analysis, X-ray crystallography, and computational modeling, we systematically examined how these structural modifications influence quantitative structure–property relationships. Our findings demonstrate that ionic liquids with long alkyl appendages respond to side-chain modifications—particularly the synergistic combination of cyclopropanation and branching—in a manner that mimics homeoviscous adaptation in living organisms. The strategic incorporation of cyclopropyl moieties combined with chiral methyl branching produced dramatic melting point depressions, with phytol-derived ionic liquids achieving the lowest melting points reported to date for these bioinspired materials. This effectiveness results from positioning these structural elements within the symmetry-breaking region of alkyl chains, where they maximally disrupt molecular packing and enhance fluidity. X-ray crystallographic analysis of a cyclopropanated citronellyl-based ionic liquid revealed that the cyclopropyl ring induces significant conformational distortions that prevent efficient molecular organization. The use of terpenoids from the chiral pool as starting materials imparts inherent sustainability to these ILs. Enantiopure ILs can be synthesized from renewable feedstocks like phytol and citronellol while exploiting bioinspired structural design principles. This work provides new insights into IL structure–property relationships that both complement and extend previous discoveries, establishing a framework for the rational design of lipidic ionic liquid systems with enhanced fluidity and chemical stability from renewable resources.

## Graphical abstract

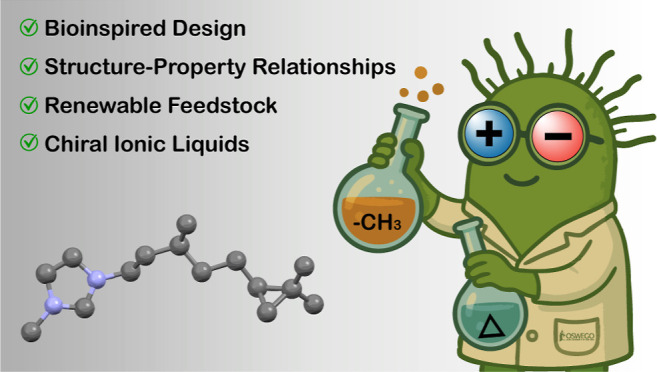

## Introduction

Cyclopropyl moieties serve as crucial structural features in natural fatty acids, enhancing membrane fluidity despite their inherent ring strain.^1^ These lipids containing cyclopropane ring fatty acids (LCFAs) are particularly prevalent in bacterial species including *Escherichia coli*, *Streptococcus*, and *Salmonella*. By introducing geometric constraints that disrupt the linear parallel packing of lipid acyl chains, the cyclopropane rings create kinks in membrane structures that enhance fluidity at lower temperatures.^2^ Certain bacteria can produce LCFAs as an adaptive response to environmental stress through in situ methylenation of pre-existing *cis*-unsaturated fatty acids. LCFA concentrations increase under harsh conditions including high osmotic pressure, extreme temperatures, acidic environments, nutrient deprivation, and high alcohol concentrations. This protective mechanism extends to bacterial pathogenicity, as exemplified by *Mycobacterium tuberculosis*, where cyclopropanated mycolic acids create a protective barrier that reduces membrane permeability and enhances antibiotic resistance.^3,4^

The nanoscale structural organization of lipid bilayers share notable parallels with ionic liquids (ILs), with both systems’ functionality dependent on fluidity as measured by melting point (*T*_m_). However, unlike lipid bilayers, ILs typically exhibit significant increases in *T*_m_ values as the ancillary aliphatic chains on the cation centers lengthen beyond eight carbons.^5^ This presents a challenge in designing lipophilic ILs that maintain melting points below room temperature.

To address this challenge, we developed lipid-inspired (or lipid-like) ILs using principles derived from homeoviscous adaptation (HVA),^6^ the same phenomenon that drives bacterial LCFA production. Our approach aimed to create ILs that simultaneously achieve high lipophilicity, low melting points, and potential biocompatibility.^7,8^ We have successfully prepared diverse classes of lipid-like ILs with remarkably low *T*_m_ values while incorporating alkyl appendages of C_16_–C_20_ by integrating specific structural motifs into the aliphatic side chains of imidazolium cations. These modifications included the addition of olefins, thioethers, methyl branches, and cyclopropane moieties that result in the disruption of alkyl-chain packing, yielding substantially lower *T*_m_ values compared to linear, saturated analogs.^7–14^

Among these modifications, cyclopropanated lipid-like ILs demonstrate distinct advantages. They exhibit superior chemical and thermal stability compared to olefin- and thioether-containing analogs, which remain susceptible to aerobic oxidation.^15^ This enhanced stability directly parallels the protective mechanisms observed in bacterial LCFA.^2^ The *T*_m_- depression efficiency of cyclopropanation exhibits chain-length dependence. In C_18_ systems, olefinic modifications achieve lower melting points than cyclopropanated variants. However, recent studies from our group revealed that this relationship can invert in shorter chain systems, with C_16_ cyclopropanated ILs demonstrating superior performance compared to their olefinic counterparts.^10^

Building upon these results, we synthesized six new lipidic ILs from high-purity (98%) bioderived terpenoid alcohols: phytol, farnesol, and geraniol. These renewable feedstock chemicals, with established safety profiles and industrial applications,^16^ provide inherent structural advantages including defined branching patterns. Enantiomerically pure phytol alcohol was used as the starting material for all IL syntheses. In phytyl-bearing ILs, the chirality originating from nature’s chiral pool introduces an additional symmetry-breaking element that further contributes to *T*_m_ depression. The resulting ILs possess alkyl chains of C_16_, C_12_, and C_8_, respectively, enabling systematic investigation of structure–property relationships across varying molecular architectures.

This work strategically positioned a cyclopropyl ring at critical ″symmetry-breaking″ positions^17^ and incorporated multiple side-chain branches to generate cumulative disruptions in molecular packing efficiency.^9,10^ The bis­(trifluoromethanesulfonyl)­imide ([Tf_2_N]^−^) anion was selected to maintain hydrophobic character and ensure thermal stability.^18^ Through facile synthesis from renewable feedstocks, we created a new class of lipid-inspired materials that successfully reconcile the opposing requirements of high lipophilicity and low melting temperature—a combination of attributes that is frequently antithetical but highly desirable from several application-specific standpoints.

## Results and Discussion

### Synthesis

Since commercially available cyclopropanated alcohols remain scarce despite their natural abundance, we synthesized the necessary alkylating agents for the ILs through a systematic four-step protocol, starting from three high-purity terpenoids: phytol, farnesol, and geraniol (Figure 1). The Simmons–Smith reaction^19,20^ successfully converted these renewable starting materials to their cyclopropanated counterparts, with transformation confirmed through NMR analysis showing characteristic upfield-shifted resonances for the exomethylene protons and backbone methine protons, as well as distinctive *exo*-CH_2_ carbon signals in the ^13^C NMR spectra. The cyclopropanated terpenoids were then converted to the corresponding mesylates and subsequently to iodides via the Finkelstein reaction. While mesylates could theoretically serve as alkylating agents, our previous investigations demonstrated that fatty alcohol iodides exhibit substantially superior alkylation efficiency.^12^

**Figure 1:**
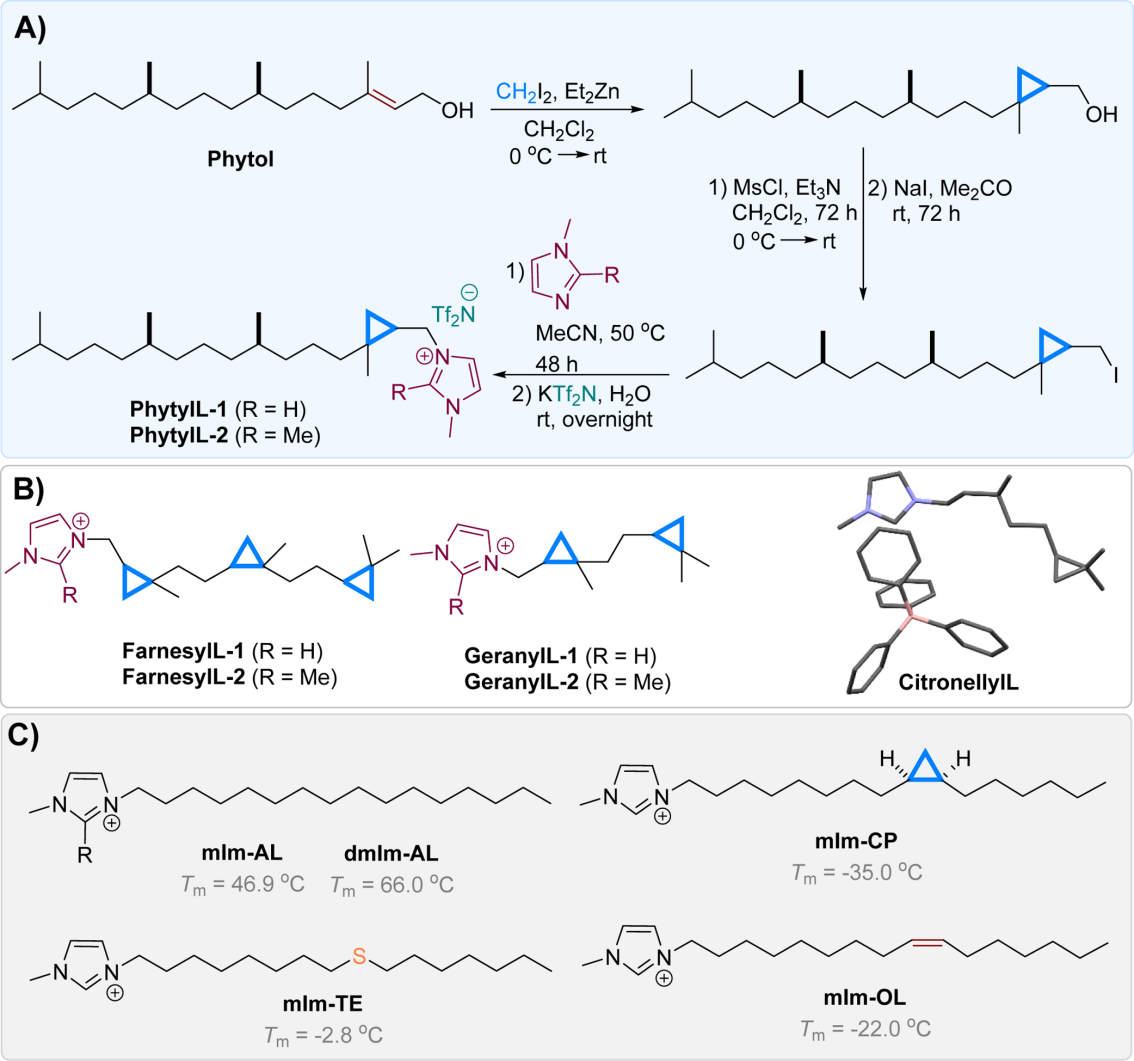
(A) Synthetic pathway for the cyclopropanated imidazolium-based IL from phytol as a representative method for the synthesis of the cyclopropanated ILs. (B) Structures of the synthesized cyclopropanated ILs derived from teraponid alcohols: phytol, farnesol, and geraniol. The crystal structure of citronellyl-derived ILs with [BPh_4_]^−^ anion. (C) Structures of benchmark lipid-like ILs with their corresponding melting points (*T*_m_).^10^ The [Tf_2_N]^−^ anion was omitted for clarity.

Substitution of the prepared iodides with methylimidazole derivatives proceeded via the S_N_2 Menschutkin reaction,^21^ reaching completion within 48 h at 50 °C in acetonitrile. The final anion metathesis^22^ step involved treating the crude iodide salts with a 20% molar excess of aqueous KTf_2_N solution, which after stirring overnight at room temperature yielded a clear phase separation with the desired IL products in the lower phase. This protocol successfully produced six target ILs—**PhytylL-1**, **PhytylL-2**, **FarnesylL-1**, **FarnesylL-2**, **GeranylL-1**, and **GeranylL-2**,—in high to excellent yields following solvent removal in vacuo (Figure 1). Structural confirmation of the final IL products was further verified via mass spectrometry.

### Structure-Melting Point Relationship Studies

Differential scanning calorimetry (DSC) analysis of the synthesized ILs revealed striking *T*_m_ depressions resulting from the strategic incorporation of cyclopropyl and branching moieties (Table 1). As noted, various self-assembling dynamics were observed for **PhytylL-1** and **PhytylL-2**, exhibiting the multifeatured traces characteristic of lipidic materials, which is often the case for ILs^23^ and lipids.^24^ The phytol-derived ILs, **PhytylL-1** and **PhytylL-2**, exhibited remarkably low *T*_m_ values compared to benchmark imidazolium-based ILs containing equivalent C_16_ side chains [i.e., saturated (**mIm-AL** and **dmIm-AL**), olefinic (**mIm-OL**), cyclopropanated (**mIm-CP**), and thioether-functionalized (**mIm-TE**)] as shown in Figure 1. Specifically, the magnitude of *T*_m_ depression achieved through the *synergistic* combination of cyclopropyl moieties and chiral branching resulted in a Δ*T*_m_ of −87.0 °C for **PhytylL-1** versus the saturated benchmark **mIm-AL**, and Δ*T*_m_ = −87.2 °C for **PhytylL-2** versus **dmIm-AL**. Notably, **PhytylL-1** exhibits the lowest *T*_m_ values reported to date for the lipid-like ILs.^7^

**Table 1:** Thermal Data of the Synthesized Lipid-Inspired ILs^a^

ILs	*T*_m_ (°C) (±0.1–0.4% °C)	*T*_g_ (°C) (±0.2 °C)	*T*_onset5%_ (°C) (±1 °C)
**PhytyIL-1**	–40.1		310
**PhytylL-2**	–21.2		331
**FarnesylL-1**	–12.9		305
**FarnesylL-2**	–7.6		303
**GeranyIL-1**		–9.3	309
**GeranylL-2**		4.9	331

a Uncertainties are calculated as the standard deviation of the mean.

Interestingly, the overall *T*_m_ depression from the inclusion of a cyclopropyl moiety and methyl branching in the IL cation side chain are substantial regardless of whether the imidazolium cation is methylated at the C2 position. This suggests two important structural considerations: (i) While electrostatics are the largest energetic contribution to lattice energy for ILs, interactions of the alkyl chains have a significant contribution to the fluidity of these ILs. (ii) Methylation of the C2 position of the imidazolium headgroup represents a valuable design strategy that increases hydrophobicity as well as chemical and thermal stability, while simultaneously decreasing fluidity (i.e., increases melting point and viscosity) through entropic effects.^25^ Moreover, this observation aligns with our earlier studies,^10,23^ confirming that strategies employed in nature to modulate *T*_m_ in lipidic materials exhibit parallel effects when applied within an IL context.

The cyclopropanated ILs displayed lower *T*_m_ values than their unsaturated counterparts, with both **PhytylL-1** and **mIm-CP** exhibiting *T*_m_ values below that of the olefinic **mIm-OL**. This unexpected trend likely results from the shorter C_16_ chain length positioning the cyclopropyl motif in the ″symmetry breaking″ region of the cation, which further disrupts solid-phase packing and reduces dispersion force interactions. The modest *T*_m_ difference between **PhytylL-1** and **mIm-CP** (Δ*T*_m_ = −5.1 °C) highlights the additional contribution of methyl branching to fluidity enhancement (Figure 2). These observations align with our understanding that cyclopropanation restricts conformational freedom in the liquid phase, thereby decreasing liquid entropy and fusion enthalpy, ultimately influencing *T*_m_.

**Figure 2:**
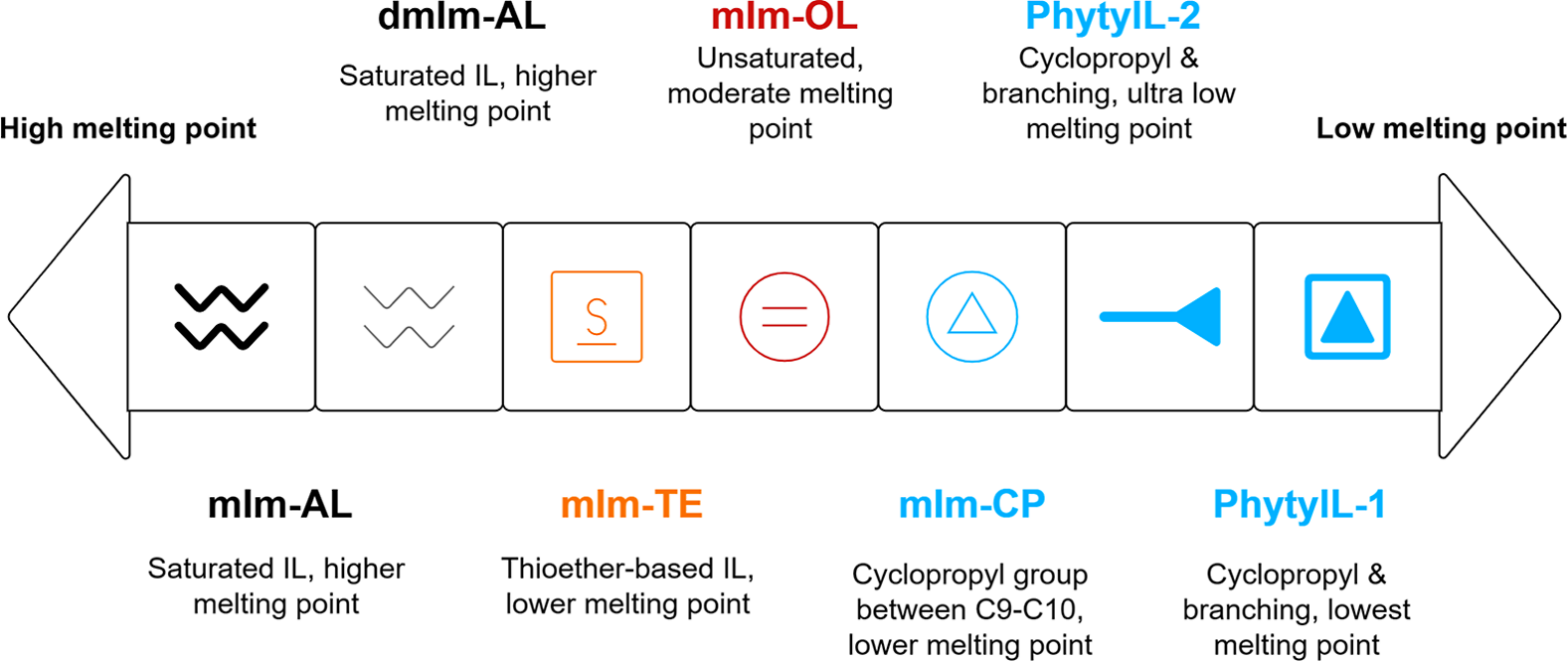
Schematic showing the relationship between IL headgroup (C2–H vs C2–Me) and side chain structures and *T*_m_.

The polycyclopropanated farnesyl derivatives **FarnsylL-1** and **FarnsylL-2** followed a similar trend, demonstrating that multiple cyclopropyl rings within a single aliphatic chain provide cumulative disruption of molecular ordering. Notably, these shorter-chain ILs achieved ultralow melting temperatures (Table 1), with **FarnsylL-1** showing a remarkable Δ*T*_m_ of −35.4 °C compared to the fully saturated analog [C_12_mim]­[Tf_2_N] (*T*_m_ = 22.5 °C).^26^ These results demonstrate that polycyclopropanation can overcome the inherent crystallization tendency of medium-chain ILs,^27^ providing a reliable pathway to low-melting lipophilic materials.

Expectedly, the profound influence of C2 methylation on the thermal properties of the ILs is exemplified by the striking 62.5 and 20.5 °C increase in *T*_m_ values observed between phytyl-based and farnesyl-based ILs, respectively. This substantial Δ*T*_m_ aligns with the molecular dynamics simulations reported by Zhang and Maginn,^28^ which demonstrated that the substitution of a methyl group for a hydrogen at the C2 position of the cation ring leads to an increase in both the *T*_m_ and viscosity. The methylation at the C2 position disrupts the conformational flexibility of the cation, reducing the entropy of the liquid phase while simultaneously affecting the H-bonding network between cations and anions. This modification effectively restricts molecular motion and increases the energy barrier for the solid-to-liquid phase transition, resulting in the dramatically elevated *T*_m_ of **PhytylL-2** and **FarnesylL-2** (Figure 2).

Most notably, the geranyl-derived ILs, **GeranylL-1** and **GeranylL-2**, which feature shorter chains with two cyclopropane moieties, exhibited only glass transitions without detectable melting transitions (Table 1). This complete suppression of crystallization indicates that the conformational constraints imposed by multiple cyclopropyl groups in shorter chains prevent efficient molecular packing entirely, favoring amorphous solid formation over crystalline structures.

The resultant thermal properties of polycyclopropanation in combination with methylation of the alkyl chain expand our understanding of structure–property relationships in the lipid-like ILs. The strategic incorporation of cyclopropane rings and branching provides a powerful tool for engineering materials with predictable phase transition behaviors, achieving melting point depressions comparable to olefin-containing ILs while offering enhanced thermo-oxidative stability. The observed transition from crystalline to amorphous character with increasing cyclopropyl content represents a critical design principle for applications requiring specific low-temperature properties.

### Thermal Stability Assessment

Thermal gravimetric analysis (TGA) was employed to evaluate the short-term thermal stability of the ILs, with particular focus on the temperature at which 5% mass loss occurred (*T*_onset5%_). While cyclopropanation is expected to improve oxidative stability compared to olefinic ILs based on the known chemical properties,^5^ the thermal stability measurements reported here were conducted under nitrogen atmosphere and thus reflect thermal degradation behavior.

Conventional methods for evaluating the thermal stability of ILs typically employ rapid heating protocols conducted in inert gas environments, where the temperatures corresponding to significant decomposition events (5% mass loss) serve as indicators of thermal stability.^29^ Furthermore, these short-duration decomposition temperatures frequently provide overly optimiztic estimates of the ILs’ real-world thermal stability when used in actual operational settings.^30^

The resulting data, compiled in Table 1, revealed high thermal stability across the entire IL series, with all compounds exhibiting initial decomposition temperatures of ≥300 °C. This high thermal stability demonstrates that the incorporation of cyclopropyl moieties and branching does not compromise the thermal robustness characteristic of imidazolium-based ILs. Analysis of the decomposition mechanism, combined with the structural characteristics of these compounds, indicates that the initial mass loss likely results from cleavage of the C–N^+^ bond via Hofmann elimination.^31^

The combination of high thermal stability and low melting points creates an exceptionally wide liquid range for these cyclopropanated ILs. This expanded operational temperature window, spanning from subambient temperatures to above 300 °C, significantly enhances their utility for high-temperature applications where conventional ILs might be limited by either crystallization or thermal decomposition. Furthermore, the enhanced thermal stability of these cyclopropanated salts compared to their olefinic analogs provides an additional practical advantage; that replacing oxidatively labile *enes* with cyclopropyl moieties addresses thermal and oxidative stability concerns without losing low-temperature fluidity.

### Enhanced Cholesterol Solubility

Not surprisingly, these ILs exhibit enhanced solubility for lipophilic biomolecules. Cholesterol was selected as a model compound due to its role as a fundamental membrane component across numerous organisms, its poor solubility in aqueous and protic systems, and the environmental challenges associated with its extraction using conventional chlorinated solvents. **PhytylL-1** dissolves cholesterol up to 33 mass percent at room temperature, while conventional ILs like [C_4_mim]­[Tf_2_N] show negligible solubility. This enhanced solubility arises from the biomimetic design of **PhytylL-1**, where cyclopropane rings serve as oxidatively stable isosteres that maintain the lipophilic character necessary for favorable interactions with cholesterol’s structure. These results suggest that such ILs could serve as effective solvents for membrane-localized biomolecules (e.g., Coenzyme Q10) and pharmaceuticals that either target membrane components or traverse phospholipid bilayers for cellular entry, thereby expanding their potential applications in biotechnology and drug delivery.

### X-ray Crystallographic Characterization

We performed the single-crystal X-ray diffraction (SCXRD) to elucidate the definitive structures of the cyclopropanated ILs and examine their molecular packing arrangements. Despite extensive crystallization attempts using various conditions and techniques, the target ILs resisted crystallization due to the conformational flexibility of their long, cyclopropanated tails, which disrupts ordered lattice formation. To overcome this challenge, we synthesized a model compound based on citronellol, incorporating a shorter C_8_ alkyl chain with a single cyclopropyl ring positioned outside the symmetry-breaking region. The tetraphenylborate ([BPh_4_]^−^) anion was selected as the counterion for its bulky, highly symmetric structure that promotes crystallization. This crystal-engineering approach successfully produced single crystals of the **CitronellylL** salt suitable for room-temperature SCXRD analysis (Figures 1 and 3).

**Figure 3:**
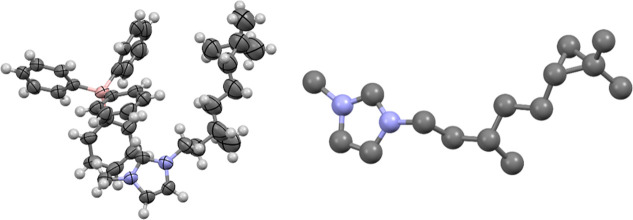
Molecular structure of the crystal of the CitronelIyIL (left) and a depiction of the cation, for clarity (right). Hydrogens and disorder are omitted for clarity. The chiral methyl groups and the cyclopropane ring help form *gauche* conformations of the alkyl chain, hindering the linear stacking of the alkyl chains and lowering the melting points.

The crystal structure is extensively disordered, with both cations in the asymmetric unit exhibiting no less than five molecular conformations (Supporting Information, Figure S1). The extensive disorder, thus, precludes analysis with respect to interactions and would make arguments of interaction geometries tenuous at best. However, despite this, there are several key details to be gleaned from the crystal structure and the impacts of the methyl and cyclopropanation of the alkyl chain.

First, ten distinct cation conformations are resolved in the structure (Supporting Information, Figure S1) underscoring the pronounced conformational freedom of the alkyl chain. As documented in earlier studies,^32^ *gauche* (*G*), *trans* (*T*) and *syn* (*S*) torsions about the N—C and C—C bonds adjacent to the imidazolium nitrogen are all observed, reflecting the broad torsional landscape accessible to IL side chains. The coexistence of these conformers further demonstrates that neither the added methyl branch nor the embedded cyclopropane ring limits this flexibility, an essential feature for achieving low melting points in imidazolium-based ILs.

Second, based on the crystallographic data gathered, there does not appear to be a significant preference for any of the aforementioned *G*, *T*, or *S* conformations. We base this observation on the percentage of the disordered parts detailed in the CIF file (see the Supporting Information). It should be noted that the molecule is observed in the solid-state and there would certainly be a different distribution of conformations in the molten state. However, given the link between the solid-state and solution structure of ILs,^13^ reasonable inferences can be drawn from the crystalline state with respect to the molten state.

Third, the pronounced disorder and the challenge of crystallizing this compound likely point to a shallow crystallographic potential-energy surface with several accessible minima.^33^ Combined with the disordered alkyl chains, this suggests that electrostatic interactions dominate the lattice enthalpy, as expected, while the alkyl chain interactions are less energetically important^34^ due to inefficient chain interactions brought about by the branching and cyclopropanation.^35^ It should be stated, however, that this model compound has a shorter alkyl chain than the other compounds in this work. As previously reported, longer alkyl chains have a significant impact on crystallinity and solidification of ILs.^36^ Collectively, these observations exemplify the “*anti-crystal-engineering*” principles that underpin the design of low-melting ILs.^37^

### Computational Analysis

To complement the crystallographic analysis, we optimized the cation extracted from the **CitronellyIL** crystal structure to explore its electronic features and assess the conformational freedom of the alkyl chain. The X-ray–derived coordinates were imported into Spartan’24 (Wave function, 2024) and the geometry relaxed to provide the optimized structure and Frontier-orbital surfaces are shown in Figure 4.

**Figure 4:**
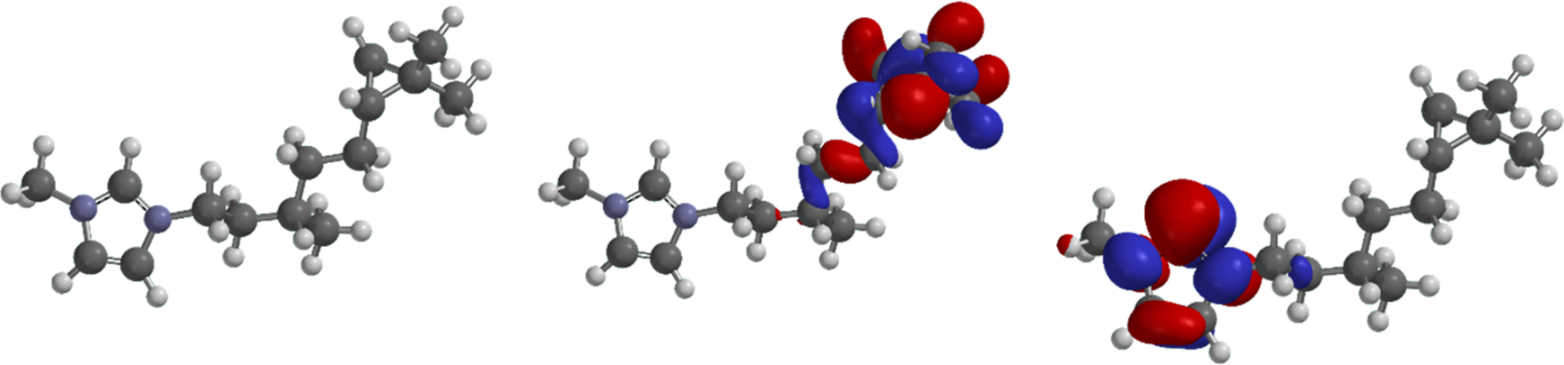
Optimized cation structure of **CitronellyIL** from the crystal (left) and depictions of the HOMO (middle) and LUMO (right).

Electronic structure is critical to understanding intermolecular interactions^38^ and crystallization^39^ in ILs. In the **CitronellyIL** cation, the LUMO is a π* orbital localized on the imidazolium heterocycle, whereas the HOMO is centered on the alkyl side chain, with density over the cyclopropane ring. These findings mirror those reported for other dialkylated imidazolium salts and suggest that we do not observe any notable impacts within this system.

Because alkyl-chain flexibility is a key factor in melting-point depression, we carried out a systematic conformer search. The global minimum, depicted in Figure 5, features stabilizing contacts between the imidazolium ring and the side chain. In total, 48 additional conformers lie within ≈16 kJ mol^–1^ of this lowest-energy structure. Notably, the crystal does not exhibit a conformation matching the computed minimum, a common divergence between gas-phase and solid-state. Several of the higher-energy conformers, however, resemble those observed crystallographically. A detailed follow-up study is under way to reconcile these differences.

**Figure 5:**
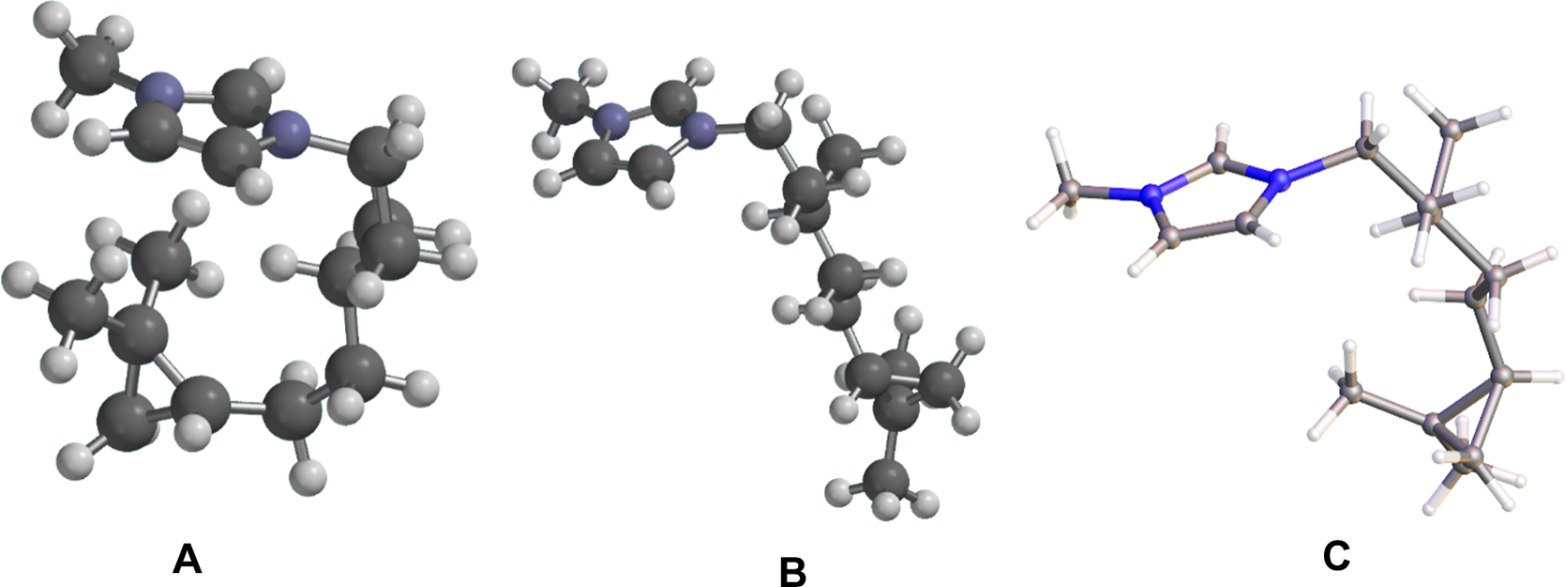
Depiction of the calculated lowest energy conformer of the **CitronellyIL** cation (A). (B,C) are a calculated conformer and a structure taken from the crystal, respectively, showing similarities between the theoretical and experimental data.

For comparison, we performed an analogous conformer search on the 1-methyl-3-octylimidazolium cation. Here, 50 conformers fall within ≈12 kJ mol^–1^ of the minimum, indicating that cyclopropanation marginally reduces the pool of energetically accessible conformers. This loss of flexibility is offset by the geometric kink imposed by the ring, which disrupts efficient crystal packing and therefore still favors a low melting point.

## Experimental Section

The detailed synthetic procedure, and the crystallographic and computational methods used in this work are described in the Supporting Information.

## Conclusions

This work represents the culmination of our decade-long investigation into lipid-like ILs, demonstrating that strategic cyclopropanation combined with branching achieves unprecedented fluidity and stability. By incorporating cyclopropyl motifs—a strategy organisms employ to modulate membrane fluidity—we have developed ILs with remarkable melting point depressions that match or exceed those achieved through olefinic modifications, while gaining the crucial advantage of enhanced oxidative stability.

The synergistic combination of cyclopropanation and branching yielded extraordinary results, with **PhytylL-1** achieving the lowest melting point reported for this class of soft materials. Beyond simple melting point reduction, cyclopropanation fundamentally alters the phase behavior and crystallization dynamics of these ILs. Through systematic investigation of diverse molecular architectures ranging from linear alkyl chains to complex terpene-derived systems, we have established a comprehensive framework for rationally designing ILs with precisely controlled properties.

Our bioinspired approach leverages renewable terpene-derived alcohols from nature’s chiral pool, producing enantiopure ILs with low melting points, excellent thermo-oxidative stability, and exceptionally wide liquidus ranges. While current synthesis requires reagents (e.g., Et_2_Zn), the development of greener alternatives, including potential biosynthetic routes,^40^ will enhance the sustainability of this platform. The unique combination of properties positions these materials for diverse applications, particularly in biocatalysis where their enhanced biomolecule solubility could facilitate enzyme-mediated transformations, therapeutic delivery systems that require dissolution of lipophilic drugs, specialized extraction media for natural products and as components in sustainable aviation fuels where cyclopropane rings provide high energy density. Ongoing work in our laboratories continues to explore these promising applications.

## Supplementary Material







## Notes

TThe Supporting Information is available free of charge at https://pubs.acs.org/doi/10.1021/acsmaterialsau.5c00089.

Synthesis and characterizations of the ILs; ^1^H, and ^13^C NMR spectra; DSC and TGA thermograms; additional computational and crystallographic data (PDF)
CitronellyIL (CIF)
CitronellyIL (CheckCIF) (PDF)
