# Supplementary material for: Lipid-Inspired Low Melting Ionic Liquids via Synergistic Cyclopropanation and Branching of Terpenoids
Source: ACS Mater Au. 2025 Jul 21;5(5):878–85. doi: 10.1021/acsmaterialsau.5c00089 (PMC12426778; doi:10.1021/acsmaterialsau.5c00089)
Supplement: Supplementary file 1 [file mg5c00089_si_001.pdf]

SUPPORTING INFORMATION

Lipid-Inspired Low Melting Ionic Liquids via Synergistic Cyclopropanation and Branching of Terpenoids

Muhammadiqboli Musozoda,[a] Richard A. O'Brien,[b] Zachary J. Metott,[a] Raychell A. Jerdo,[a] Christopher M. Butch,[a] Matthias Zeller,[c] Gregory R. Boyce,*,[d] Patrick C. Hillesheim,*,[e] Arsalan Mirjafari*,[a]

[a] Department of Chemistry, State University of New York at Oswego, Oswego, New York 13126, United States

[b] Department of Chemistry, The University of South Alabama, Mobile, Alabama 36688, United States

[c] Department of Chemistry, Purdue University, West Lafayette, Indiana 47907, United States

[d] Department of Chemistry and Biochemistry, East Stroudsburg University, East Stroudsburg, Pennsylvania 18301, United States

[e] Department of Chemistry, Illinois State University, Normal, Illinois 61761, United States

Corresponding Authors:

Emails: arsalan.mirjafari@oswego.edu (AM), pchille@ilstu.edu (PCH), gboyce@esu.edu (GRB)

EXPERIMENTAL PROCEDURES AND CHARACTERIZATION DATA

Materials and Instrumentation

All commercial chemicals were used as received unless otherwise noted. ^{1}H and ^{13}C NMR analyses were performed on a Bruker 500 MHz NMR at 295 K with the chemical shifts (δ) notated as parts per million (ppm) and referenced to the residual ^{1}H signal of $CDCl_3$ or DMSO-d_6 as solvents at room temperature.

The mass spectrometry (MS) data were obtained using a Thermo Scientific Altis TSQ triple-quadrupole mass spectrometer. Samples were prepared at 10 ppm in LCMS grade acetonitrile (Fisher Optima) and introduced into the MS directly by syringe pump set to mix 5 uL/min of this solution into a 0.2 mL/min flow from the HPLC consisting of acetonitrile with 0.1% formic acid (Fisher Optima). Data were collected using the automated optimization for "selected reaction monitoring" (SRM) analysis in the Chromeleon software, using argon (1.5 mTorr) in the collision chamber and a capillary temperature of 325 °C. Each type of gas flow was optimized for each molecular ion and was within a 20% range of the default values.

The reported optimal isolation mass for the molecular *m/z* is the peak of this band; this is a low-resolution mass spectrometer, so the mass agreement is uniformly excellent. This was optimized for source voltage (CID), which was 0 V, except where otherwise noted. The capillary voltage (VCAP) was also optimized and is reported. The top 5 SRM reactions are reported with their max intensity at optimized collision energy/ voltage (CV) for that product. These are reported with their relative maximum intensity as well as the intensity of the SRM ion relative to the molecular ion maximum intensity at CV = 0.

Single crystal XRD experiments were carried out with a Bruker AXS D8 Quest diffractometer with a PhotonIII charge-integrating pixel array detector (CPAD) and an I-mu-S 3.0 Cu-*Kα* radiation microsource X-ray tube. Absorption was corrected by multi-scan methods using *SADABS*. Additional details are provided in the Crystallographic Data section.

General Synthetic Procedure

The synthesis of cyclopropanated ionic liquids was accomplished through a five-step sequence starting from commercially available unsaturated alcohols, following our previously reported methodology with slight modifications.[1] Briefly,

Simmons-Smith Cyclopropanation. The unsaturated alcohol (1.0 equiv.) was treated with diiodomethane (6.2 equiv.) and diethylzinc (5.9 equiv., 1.0 M in hexanes) in CH_2Cl_2 at 0 °C. After warming to room temperature and stirring overnight, the reaction was quenched with water to afford the cyclopropanated alcohol in 79% yield.

Mesylate Formation. The cyclopropanated alcohol was converted to the corresponding methanesulfonate ester using methanesulfonyl chloride (2.2 equiv.) and Et_3N (2.2 equiv.) in CH_2Cl_2 at room temperature for 72 hours, providing the mesylate in 92% yield.

Finkelstein Reaction. The methanesulfonate was transformed to the corresponding iodide through treatment with sodium iodide (8.6 equiv.) in acetone at room temperature for 3 days, yielding the alkyl iodide in 94% yield.

Menschutkin Reaction. Quaternization of 1-methylimidazole/1,2-dimethylimidazole (1.2 equiv.) with the alkyl iodide was performed in acetonitrile at 50 °C for two days to yield the imidazolium iodide salt in 86%.

Anion Metathesis. The final ionic liquid was obtained by anion exchange of the imidazolium iodide with potassium bis(trifluoromethanesulfonyl)imide (1.2 equiv.) in a biphasic chloroform/water system. After extraction and washing with hexanes, the target cyclopropanated ionic liquid was isolated in 98% yield as a viscous liquid.

For the preparation of citronellyl-based salt, we employed the modified conditions for the Appel reaction[2] to convert the cyclopropanated citronellol to the corresponding bromide. The resulting bromide underwent quaternization via an S_N2 Menschutkin reaction with the appropriate *N*-methylimidazole derivatives. The final **CitronellylL** salt was obtained through anion metathesis, where the bromide salts were treated with sodium tetraphenylborate in water. For X-ray crystallographic studies, the **CitronellylL** was recrystallized by slow evaporation from hot methanol, which provided single crystals suitable for X-ray diffraction analysis.

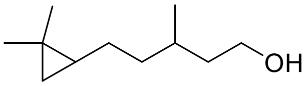

5-(2,2-Dimethylcyclopropyl)-3-methylpentan-1-ol. (±)-Citronellol (5.0 mL, 16.4 mmol, 1 equiv.) and anhydrous CH_2Cl_2 (0.10 M, 164 mL) were added to a flame-dried flask under an N_2 atmosphere. The solution was cooled to 0 °C and a 1.0 M diethylzinc in hexanes (55 mL, 55 mmol, 3.3 equiv.) was added dropwise over 30 minutes. The solution was allowed to stir for 10 minutes, then diiodomethane (2.64 mL, 32.8 mmol, 2 equiv.) was added dropwise. The reaction was allowed to warm to room temperature overnight then quenched with water (50 mL). The reaction mixture was diluted with ether and washed with a saturated solution of NH_4Cl followed by a saturated solution of $Na_2S_2O_3$, and brine. The combined organic layers were dried with anhydrous $MgSO_4$ and filtered. The solvent was removed under reduced pressure and the residue was purified by column chromatography (10% EtOAc/Hexanes) to give the title compound (2.53 g, 90%) as a colorless oil with spectroscopic data in accordance with the literature.[3]

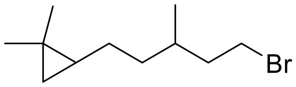

2-(5-Bromo-3-methylpentyl)-1,1-dimethylcyclopropane. Imidazole (745 mg, 11.3 mmol, 1.5 equiv.), PPh_3 (2.57 g, 9.8 mmol, 1.3 equiv.), and anhydrous CH_2Cl_2 (23 mL) were added to a flame-dried flask and placed under an N_2 atmosphere. The mixture was cooled to 0 °C and a solution of CBr_4 (3.24 g, 9.8 mmol, 1.3 equiv.) in CH_2Cl_2 (2.5 mL) was added dropwise. The reaction mixture was stirred at 0 °C for 5 minutes then 5-(2,2-dimethylcyclopropyl)-3-methylpentan-1-ol (1.28 g, 7.5 mmol, 1.0 equiv.) in CH_2Cl_2 (5 mL) was added dropwise to the flask. The reaction was then allowed to warm to room temperature over 1 hour. The reaction was quenched with water (20 mL) and extracted with dichloromethane. The combined organic layers were dried with Na_2SO_4, filtered, and concentrated under reduced pressure. The residue was triturated with a hexanes-ether solution (10:1, 10 mL) then purified by column chromatography (100% hexanes) to yield the title compound (1.14 g, 65%) as a colorless oil with spectroscopic data in accordance with the literature.[4]

PhytylL-1. ^{1}H NMR (500 MHz, $CDCl_3$) δ 8.61 (s, 1H), 7.34 (t, *J* = 1.9 Hz, 1H), 7.25 (d, *J* = 1.8 Hz, 1H), 5.38 (td, *J* = 7.5, 1.4 Hz, 1H), 4.76 (d, *J* = 7.5 Hz, 2H), 3.92 (s, 3H), 2.08 (dt, *J* = 9.0, 5.9 Hz, 2H), 1.78 (d, *J* = 1.3 Hz, 3H), 1.53 (dp, *J* = 13.3, 6.6 Hz, 1H), 1.23 (m, 19H), 0.86 (m, 13H); ^{13}C NMR (125 MHz, $CDCl_3$) δ 147.3, 135.5, 123.8, 123.6, 121.8, 121.1, 118.5, 116.0, 114.5, 47.3, 39.8, 39.4, 37.4, 37.3, 36.7, 36.2, 32.8, 27.9, 25.0, 24.8, 24.4, 22.7, 22.6, 19.7, 16.3; MS (ESI): *m/z* 375.376 (M = $C_{25}H_{47}N_2^+$, calculated 375.373).

PhytylL-2. ^{1}H NMR (500 MHz, $CDCl_3$) δ 7.18 (d, *J* = 2.1 Hz, 1H), 7.09 (t, *J* = 2.0 Hz, 1H), 5.26 (m, 1H), 4.63 (m, 2H), 3.79 (s, 3H), 2.59 (s, 3H), 2.13 (m, 1H), 2.07 (dt, *J* = 8.4, 5.7 Hz, 1H), 1.81 (d, *J* = 1.3 Hz, 1H), 1.53 (dp, *J* = 13.2, 6.6 Hz, 1H), 1.39 (s, 4H), 1.26 (m, 9H), 1.10 (m, 7H), 0.87 (dd, *J* = 6.6, 0.9 Hz, 14H); ^{13}C NMR (125 MHz, $CDCl_3$) δ 146.2, 143.7, 122.2, 121.1, 120.2, 118.5, 115.4, 114.6, 46.5, 46.3, 39.8, 39.4, 37.4, 36.8, 36.7, 35.2, 32.8, 32.3, 27.9, 25.1, 24.8, 23.2, 22.7, 19.7, 19.5, 16.4; MS (ESI): *m/z* 389.385 (M = $C_{26}H_{49}N_2^+$, calculated 389.389).

FarnesylL-1. ^{1}H NMR (500 MHz, $CDCl_3$) 8.52-8.77 (m, 3H), 5.40 (d, J = 2.7 Hz, 2H), 5.09 (s, 1H), 4.81 (s, 2H), 3.98 (s, 3H), 1.62-2.38 (m, 23H); ^{13}C NMR (125 MHz, $CDCl_3$) δ ^{13}C NMR (126 MHz, $CDCl_3$) 147.1, 136.1, 135.7, 131.6, 124.2, 123.2, 121.8, 118.3, 114.92, 77.1, 47.4, 39.8, 39.5, 36.5, 26.7, 25.8, 23.5, 17.8, 16.6, 16.1; MS (ESI): *m/z* 301.266 (M = $C_{20}H_{33}N_2^+$, calculated 301.264).

FarnesylL-2. ^{1}H NMR (500 MHz, $CDCl_3$) δ 7.17 (m, 4H), 7.10 (d, *J* = 2.2 Hz, 3H), 5.27 (s, 3H), 5.08 (s, 1H), 4.63 (d, *J* = 7.2 Hz, 2H), 3.78 (s, 3H), 2.62 (s, 1H), 1.62-2.58 (m, 18H); ^{13}C NMR (125 MHz, $CDCl_3$) δ 145.9, 143.7, 136.0, 131.5, 124.1, 123.9, 122.2, 121.0, 118.5, 114.8, 46.5, 39.7, 39.4, 31.9, 26.7, 26.5, 26.0, 25.7, 23.3, 17.7, 16.5, MS (ESI): *m/z* 315.273 (M = $C_{21}H_{35}N_2^+$, calculated 315.279).

GeranylL-1. ^{1}H NMR (500 MHz, $CDCl_3$) δ 8.60 (d, *J* = 1.9 Hz, 1H), 7.33 (t, *J* = 1.8 Hz, 1H), 7.24 (q, *J* = 1.7 Hz, 1H), 5.38 (ddt, *J* = 7.5, 6.1, 1.3 Hz, 1H), 5.05 (ddp, *J* = 3.9, 2.6, 1.2 Hz, 1H), 4.75 (d, *J* = 7.5 Hz, 3H), 3.92 (s, 5H), 2.14 (d, *J* = 3.3 Hz, 5H), 1.84 (q, *J* = 1.1 Hz, 1H), 1.78 (d, *J* = 1.4 Hz, 4H), 1.67 (dd, *J* = 6.2, 1.3 Hz, 4H); ^{13}C NMR (125 MHz, $CDCl_3$) δ 146.8, 135.5, 132.9, 132.3, 123.8, 122.8, 121.8, 121.1, 118.5, 114.8, 39.3, 31.9, 25.9, 25.6, 17.6, 16.3; MS (ESI): *m/z* 247.211 (M = $C_{16}H_{27}N_2^+$, calculated 247.217).

GeranylL-2. ^{1}H NMR (500 MHz, DMSO-d_6) δ 9.05 (s, 1H), 7.69 (m, 4H), 5.40 (s, 1H), 5.08 (s, 1H), 4.80 (d, *J* = 7.5 Hz, 3H), 3.85 (s, 5H), 2.09 (d, *J* = 10.7 Hz, 3H), 1.77 (d, *J* = 1.6 Hz, 4H), 1.65 (s, 2H), 1.57 (s, 2H), 1.06 (s, 2H), 1.00 (s, 1H); ^{13}C NMR (125 MHz, DMSO-d_6) δ 144.1, 136.6, 131.7, 124.1, 122.6, 121.2, 118.7, 117.3, 116.1, 77.4, 72.0, 46.8, 36.2, 26.1, 25.9, 24.9, 18.0, 16.7; MS (ESI): *m/z* 261.235 (M = $C_{17}H_{29}N_2^+$, calculated 261.233).

Differential Scanning Calorimetry (DSC)

In this work, the melting points are reported as the transition from crystalline solid state to the isotropic liquid state, distinguished by the magnitude of enthalpy for the transition and the shape of the DSC curve. Melting points and glass transition temperatures were measured using a TA Discovery 250 DSC Differential Scanning Calorimeter, calibrated using indium (melting point) and sapphire (heat capacity) references. For each experiment, 5−10 mg of the sample were loaded into an open aluminum pan and heated to 120 °C for 20 min to remove any water absorbed from the environment, residual solvents, or volatile contaminants from synthesis. The samples were

then cooled to −50 °C, equilibrated for 2 min and then heated at a ramp rate of 5 °C/min to 200 °C. Determined by the TRIOS analysis software, melting points are reported as the melting onset temperature and glass transition temperatures are reported as the midpoints of the phase transitions. The samples underwent 8–10 heating and subsequent cooling processes at a rate of 5 °C/min, alternating with 5 min isothermal periods to identify the correct phase transitions by observing three overlapping cycles and reported values are the average of three measurements. All measurements were carried out under a nitrogen atmosphere (50 mL/min) and were reproducible to within ±0.1–0.4 °C.

Thermogravimetric Analysis (TGA)

Thermogravimetric analyses were performed on a TA instrument TGA 550 under nitrogen atmosphere flow using a platinum pan. The samples were heated from room temperature at a rate of 10 °C/min to a maximum temperature of 500 °C. For each experiment, ~5 mg of the sample was loaded into an open platinum pan and heated to 120 °C for 10 min to remove any water absorbed from the environment, residual solvents, or volatile contaminants from synthesis. Claims regarding improved oxidative stability relative to olefinic ILs are based on established chemical principles[5] rather than direct oxidative stability testing. The data was reported as an average of three attempts. Uncertainties were calculated from a minimum of three independent measurements using standard deviation of the mean.

X-Ray Crystallographic Data

Structural data for **CitronellylIL** were collected on a Bruker Quest diffractometer with kappa geometry, a Cu Kα wavelength (λ = 1.54178 Å) I-μ-S 3.0 microsource X-ray tube, Montel optics for monochromatization, a Photon III area detector and an Oxford Cryosystems low temperature device. Examination and data collection were performed at 150 K. The space group was assigned using XPREP within the SHELXTL suite of programs,[6,7] the structure was solved by dual methods using ShelXT[8] and refined by full matrix least squares against F^2 with all reflections using Shelxl2019[9] using the graphical interfaces Shelxle.[10] H atoms were positioned geometrically and constrained to ride on their parent atoms. C-H bond distances were constrained to 0.95 Å for imidazole C—H moieties, and to 0.99 and 0.98 Å for aliphatic CH_2 and CH_3 moieties, respectively. Methyl H atoms were allowed to rotate, but not to tip, to best fit the experimental electron density. U_{iso}(H) values were set to a multiple of U_{eq}(C) with 1.5 for CH_3 and 1.2 for C—H and CH_2 units, respectively.

The structure, and especially the cations, show extensive disorder. For both cations, several orientations are clearly resolved, and several more are apparent but assignment of exact conformation is ambiguous. Five orientations were refined for each cation. Additional disorder is apparent but was not modeled.

Anions were refined as disordered over each two domains. For the anions, the four disordered moieties were restrained to have similar geometries. U_{ij} components of ADPs for disordered atoms closer to each other than 2.0 Å were restrained to be similar. Subject to these conditions the occupancy ratios refined to 0.526(9) to 0.474(9) for anion1, and to 0.719(4) to 0.281(4) for anion 2.

For the cations, disordered moieties were restrained to have similar geometries. In addition, all alkyl bond lengths and some bond angles that are expected to be similar in length

(chemically equivalent) were restrained to be similar. The imidazolium segments were restrained to be close to planar. U_{ij} components of ADPs for disordered atoms closer to each other than 2.0 Å were restrained to be similar. Atoms C4 to C4E of cation 1 were constrained to have identical ADPs. Subject to these conditions, the occupancy ratios refined to 0.445(3) to 0.216(3) to 0.113(2) to 0.140(2) to 0.086(2) for cation 1, and to 0.297(2) to 0.230(2) to 0.182(2) to 0.161(2) to 0.131(2) for cation 2.

Complete crystallographic data, in CIF format, was deposited with the Cambridge Crystallographic Data Centre with CCDC number 2456646 that contains the supplementary crystallographic data for this paper. These data can be obtained free of charge from The Cambridge Crystallographic Data Centre via www.ccdc.cam.ac.uk/data_request/cif.

References

(1) O'Brien, R. A.; Hillesheim, P. C.; Soltani, M.; Badilla-Nunez, K. J.; Siu, B.; Musozoda, M.; West, K. N.; Davis, J. H., Jr.; Mirjafari, A. Cyclopropane as an Unsaturation "Effect Isostere": Lowering the Melting Points in Lipid-like Ionic Liquids. *J. Phys. Chem. B* **2023**, *127* (6), 1429–1442.

(2) Fujihara, T.; Horimoto, Y.; Mizoe, T.; Sayyed, F. B.; Tani, Y.; Terao, J.; Sakaki, S.; Tsuji, Y. Nickel-Catalyzed Double Carboxylation of Alkynes Employing Carbon Dioxide. *Org. Lett.* **2014**, *16* (18), 4960–4963.

(3) Brunner, G.; Elmer, S.; Schröder, F. Transition-Metal-Catalyzed Cyclopropanation of Nonactivated Alkenes in Dibromomethane with Triisobutylaluminum. *Eur. J. Org. Chem.* **2011**, *2011* (24), 4623–4633.

(4) Hovde, H. R.; Kleveland, K.; Nilsen, N. O.; Stenstrom, Y.; Skattebol, L. Preparation of Juvenile Hormone Analogues and Their Use as Antifouling Agents. WO2001006853, February 1, 2001.

(5) Murray, S. M.; O'Brien, R. A.; Mattson, K. M.; Ceccarelli, C.; Sykora, R. E.; West, K. N.; Davis, J. H. The Fluid-Mosaic Model, Homeoviscous Adaptation, and Ionic Liquids: Dramatic Lowering of the Melting Point by Side-Chain Unsaturation. *Angew. Chem., Int. Ed.* **2010**, *49* (15), 2755–2758.

(6) SHELXTL Suite of Programs, Version 6.14, 2000-2003, Bruker Advanced X-Ray Solutions.

(7) Sheldrick, G. M. A Short History of *SHELX*. *Acta Crystallogr., Sect. A: Found. Crystallogr.* **2008**, *64* (1), 112–122.

(8) Sheldrick, G. M. *SHELXT* – Integrated Space-Group and Crystal-Structure Determination. *Acta Crystallogr., Sect. A: Found. Adv.* **2015**, *71* (1), 3–8.

(9) Sheldrick, G. M. Crystal Structure Refinement with *SHELXL*. *Acta Crystallogr., Sect. C: Struct. Chem.* **2015**, *71* (1), 3–8.

(10) Hübschle, C. B.; Sheldrick, G. M.; Dittrich, B. *ShelXle*: A Qt Graphical User Interface for *SHELXL*. *J. Appl. Crystallogr.* **2011**, *44* (6), 1281–1284.

(11) Lin, Y.-S.; Li, G.-D.; Mao, S.-P.; Chai, J.-D. Long-Range Corrected Hybrid Density Functionals with Improved Dispersion Corrections. *J. Chem. Theory Comput.* **2013**, *9* (1), 263–272.

(12) Ditchfield, R.; Hehre, W. J.; Pople, J. A. Self-Consistent Molecular-Orbital Methods. IX. An Extended Gaussian-Type Basis for Molecular-Orbital Studies of Organic Molecules. *J. Chem. Phys.* **1971**, *54* (2), 724–728.

Table S1. Experimental details

	**CitronellylL**
Crystal data	
Chemical formula	$C_{24}H_{20}B \cdot C_{15}H_{27}N_2$
M_r	554.59
Crystal system, space group	Monoclinic, $P2_1/n$
Temperature (K)	150
a, *b*, *c* (Å)	11.2099 (5), 37.121 (2), 16.4619 (9)
β (°)	102.007 (4)
V (Å^3)	6700.2 (6)
Z	8
Radiation type	Cu $K\alpha$
μ (mm^{-1})	0.47
Crystal size (mm)	0.32 × 0.19 × 0.09
Data collection	
Diffractometer	Bruker AXS D8 Quest
Absorption correction	Multi-scan<br>*SADABS* 2016/2: Krause, L., Herbst-Irmer, R., Sheldrick G.M. & Stalke D., J. Appl. Cryst. 48 (2015) 3-10
T_{min}, T_{max}	0.603, 0.754
No. of measured, independent and observed [$I > 2\sigma(I)$] reflections	54636, 14223, 11040
R_{int}	0.035
(sin θ/λ)$_{max}$ (Å^{-1})	0.639
Refinement	
$R[F^2 > 2\sigma(F^2)]$, $wR(F^2)$, S	0.069, 0.242, 1.08
No. of reflections	14223
No. of parameters	2421
No. of restraints	14659
H-atom treatment	H-atom parameters constrained
$\Delta\rho_{max}$, $\Delta\rho_{min}$ (e Å^{-3})	0.25, −0.20

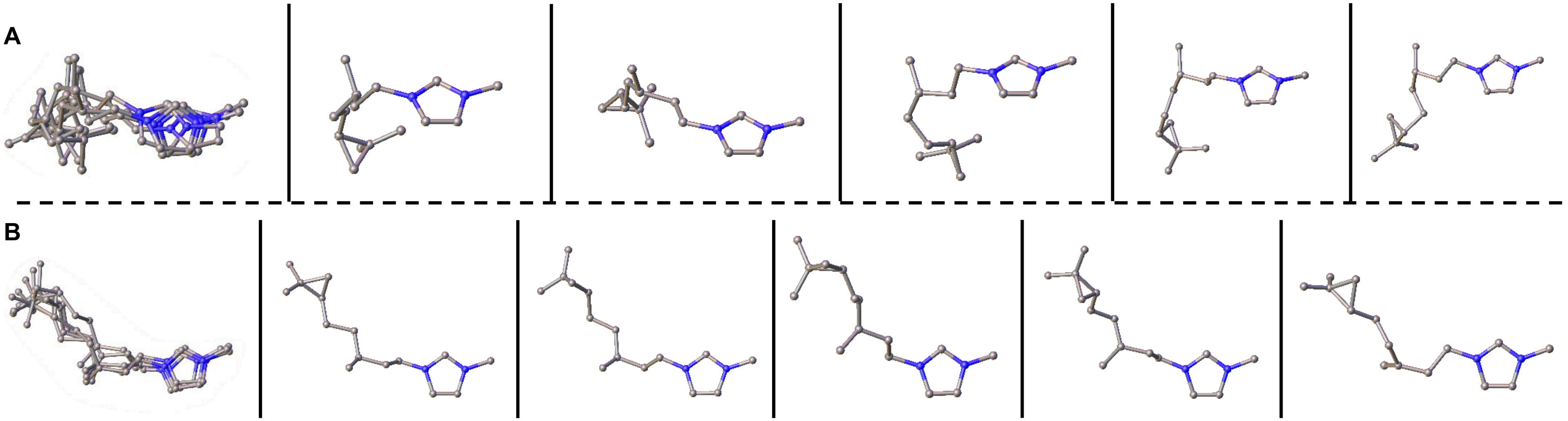

Figure S1. Conformational diversity in the crystal structure of **CitronellyIL**. The individual cations from the asymmetric unit are shown and labeled as **A** and **B**. These cations show extensive disorder with 5 models comprising the final solution for the cations. These five conformers are shown separately (right panels) to highlight the range of torsion angles and alkyl chain geometries accessible to the citronellyl substituent, demonstrating the conformational flexibility that contributes to packing frustration and melting point depression.

Computational Data

All calculations were completed using Spartan'24 (Wavefunction, 2024). The coordinates of the CitronellylIL cation from the crystal structure were loaded into Spartan and the geometry relaxed using the ωB97X-D functional[11] with a 6-311+G** basis set.[12] For the 1-methyl-3-octylimidazolium cation, the same procedure was completed.

A conformer search was then completed on the relaxed cations with an energy cutoff of 20 kJ/mol with an option to keep a maximum of 50 conformers. The coordinates of the final conformers are provided herein. The change in energy (Δ*E*) of the conformers is also provided as reference.

Table S2. Tabulated relative energies of the conformers of the **CitronellylL** and 1-methyl-octylimidazolium (Omim) cations.

CitronellylL Cation Conformer	Δ*E* (kJ/mol)
**1**	0.00
**2**	0.39
**3**	0.87
**4**	1.87
**5**	2.34
**6**	3.02
**7**	3.80
**8**	3.84
**9**	5.89
**10**	6.08
**11**	8.50
**12**	8.58
**13**	9.38
**14**	9.82
**15**	9.90
**16**	10.56
**17**	10.87
**18**	10.90
**19**	10.98
**20**	11.49
**21**	11.52
**22**	11.55
**23**	11.56
**24**	11.83
**25**	12.01
**26**	12.07
**27**	12.76
**28**	12.83

Omim Conformer	Δ*E* (kJ/mol)
**1**	0.00
**2**	3.26
**3**	4.09
**4**	4.20
**5**	4.37
**6**	5.29
**7**	5.80
**8**	6.09
**9**	6.12
**10**	6.18
**11**	6.28
**12**	6.36
**13**	6.47
**14**	7.16
**15**	7.43
**16**	7.61
**17**	7.84
**18**	7.88
**19**	7.91
**20**	8.03
**21**	8.06
**22**	8.38
**23**	8.41
**24**	8.50
**25**	8.56
**26**	8.91
**27**	8.97
**28**	8.98

**29**	12.86
**30**	13.33
**31**	13.59
**32**	13.60
**33**	13.66
**34**	13.68
**35**	13.70
**36**	13.86
**37**	14.08
**38**	14.16
**39**	14.16
**40**	14.24
**41**	14.45
**42**	14.51
**43**	14.91
**44**	14.98
**45**	15.09
**46**	15.16
**47**	15.25
**48**	15.78
**49**	16.13
**50**	-

**29**	9.15
**30**	9.21
**31**	9.43
**32**	9.71
**33**	9.85
**34**	9.89
**35**	9.94
**36**	9.96
**37**	10.04
**38**	10.05
**39**	10.09
**40**	10.13
**41**	10.26
**42**	10.41
**43**	10.46
**44**	10.48
**45**	10.54
**46**	10.68
**47**	10.75
**48**	10.88
**49**	11.27
**50**	11.28

Computational Log for Optimization of the **CintronelyIL**

SPARTAN'24

build 1.3.0 (Apr 1 2025)

Wavefunction Developers:

B.J. Deppmeier, A.J. Driessen, W.J. Hehre, T.S. Hehre,

J.A. Johnson, W.S. Ohlinger, P.E. Klunzinger

Please cite Spartan as:

Spartan'24

Wavefunction Inc.

Irvine CA

Q-Chem, Inc., Pleasanton, CA (2025)

Q-Chem Developers:

Yihan Shao, Zhengting Gan, E. Epifanovsky, A. T. B. Gilbert, M. Wormit, J. Kussmann, A. W. Lange, A. Behn, Jia Deng, Xintian Feng, D. Ghosh, M. Goldey, P. R. Horn, L. D. Jacobson, I. Kaliman, T. Kus, A. Landau, Jie Liu, E. I. Proynov, R. M. Richard, R. P. Steele, E. J. Sundstrom, H. L. Woodcock III, P. M. Zimmerman, D. Zuev, B. Alam, B. Albrecht, E. Alguire, S. A. Baeppler, D. Barton, Z. Benda, Y. A. Bernard, E. J. Berquist, K. B. Bravaya, H. Burton, K. Carter-Fenk, D. Casanova, Chun-Min Chang, Yunqing Chen, A. Chien, K. D. Closser, M. P. Coons, S. Coriani, S. Dasgupta, A. L. Dempwolff, M. Diedenhofen, Hainam Do, R. G. Edgar, Po-Tung Fang, S. Faraji, S. Fatehi, Qingguo Feng, J. Fosso-Tande, J. Gayvert, Qinghui Ge, A. Ghysels, G. Gidofalvi, J. Gomes, J. Gonthier, A. Gunina, D. Hait, M. W. D. Hanson-Heine, P. H. P. Harbach, A. W. Hauser, M. F. Herbst, J. E. Herr, E. G. Hohenstein, Z. C. Holden, Kerwin Hui, B. C. Huynh, T.-C. Jagau, Hyunjun Ji, B. Kaduk, K. Khistyaev, Jaehoon Kim, P. Klunzinger, K. Koh, D. Kosenkov, L. Koulias, T. Kowalczyk, C. M. Krauter, A. Kunitsa, Ka Un Lao, A. Laurent, K. V. Lawler, Joonho Lee, D. Lefrancois, S. Lehtola, D. S. Levine, Yi-Pei Li, You-Sheng Lin, Fenglai Liu, Kuan-Yu Liu, E. Livshits, M. Loipersberger, A. Luenser, P. Manohar, E. Mansoor, S. F. Manzer, Shan-Ping Mao, Yuezhi Mao, N. Mardirossian, A. V. Marenich, T. Markovich, L. A. Martinez-Martinez, S. A. Maurer, N. J. Mayhall, S. C. McKenzie, J.-M. Mewes, P. Morgante, A. F. Morrison, J. W. Mullinax, K. Nanda, T. S. Nguyen-Beck, R. Olivares-Amaya, J. A. Parkhill, S. K. Paul, Zheng Pei, T. M. Perrine, F. Plasser, P. Pokhilko, S. Prager, A. Prociuk, E. Ramos, B. Rana, D. R. Rehn, F. Rob, M. Scheurer, M. Schneider, N. Sergueev, S. M. Sharada, S. Sharma, D. W. Small, T. Stauch, C. J. Stein, T. Stein, Yu-Chuan Su, S. P. Veccham, A. J. W. Thom, A. Tkatchenko, T. Tsuchimochi, N. M. Tubman, L. Vogt, M. L. Vidal, O. Vydrov, M. A. Watson, J. Wenzel,

M. de Wergifosse, T. A. Wesolowski, A. White, J. Witte, A. Yamada, Jun Yang, K. Yao, S. Yeganeh, S. R. Yost, Zhi-Qiang You, A. Zech, Igor Ying Zhang, Xing Zhang, Yan Zhao, Ying Zhu, B. R. Brooks, G. K. L. Chan, C. J. Cramer, M. S. Gordon, W. J. Hehre, A. Klamt, M. W. Schmidt, C. D. Sherrill, D. G. Truhlar, A. Aspuru-Guzik, R. Baer, A. T. Bell, N. A. Besley, Jeng-Da Chai, A. E. DePrince, III, R. A. DiStasio Jr., A. Dreuw, B. D. Dunietz, T. R. Furlani, Chao-Ping Hsu, Yousung Jung, Jing Kong, D. S. Lambrecht, WanZhen Liang, C. Ochsenfeld, V. A. Rassolov, L. V. Slipchenko, J. E. Subotnik, T. Van Voorhis, J. M. Herbert, A. I. Krylov, P. M. W. Gill, M. Head-Gordon,

Contributors to earlier versions of Q-Chem not listed above:

R. D. Adamson, B. Austin, J. Baker, G. J. O. Beran, K. Brandhorst, S. T. Brown, E. F. C. Byrd, A. K. Chakraborty, C.-L. Cheng, Siu Hung Chien, D. M. Chipman, D. L. Crittenden, H. Dachsel, R. J. Doerksen, A. D. Dutoi, L. Fusti-Molnar, W. A. Goddard III, A. Golubeva-Zadorozhnaya, S. R. Gwaltney, G. Hawkins, A. Heyden, S. Hirata, G. Kedziora, F. J. Keil, C. Kelley, Jihan Kim, R. A. King, R. Z. Khaliullin, P. P. Korambath, W. Kurlancheek, A. M. Lee, M. S. Lee, S. V. Levchenko, Ching Yeh Lin, D. Liotard, R. C. Lochan, I. Lotan, P. E. Maslen, N. Nair, D. P. O'Neill, D. Neuhauser, E. Neuscamman, C. M. Oana, R. Olson, B. Peters, R. Peverati, P. A. Pieniazek, Y. M. Rhee, J. Ritchie, M. A. Rohrdanz, E. Rosta, N. J. Russ, H. F. Schaefer III, N. E. Schultz, N. Shenvi, A. C. Simmonett, A. Sodt, D. Stuck, K. S. Thanthiriwatte, V. Vanovschi, Tao Wang, A. Warshel, C. F. Williams, Q. Wu, X. Xu, W. Zhang,

Please cite Q-Chem as follows :

E. Epifanovsky et al., J. Chem. Phys. 155, 084801 (2021)

https://doi.org/10.1063/5.0055522

Parts of Q-Chem use Armadillo 8.300.2 (tropical Shenanigans).

http://arma.sourceforge.net/

Please cite Spooky-Net as:

Unke, O. T., Chmiela, S., Gastegger, M., Schutt, K. T., Sauceda,
H. E., & Müller, K. R. (2021).
Spookynet: Learning force fields with electronic degrees of freedom
and nonlocal effects. Nat. Commun. 12(1), 2021, 1-14.

Wavefunction Inc. Sales: sales@wavefun.com
Irvine CA Support: support@wavefun.com
Web: www.wavefun.com

Copyright © 1995 - 2025

Wavefunction Version of Q-Chem

Parts of Q-Chem use Armadillo 8.300.2 (Tropical Shenanigans).

http://arma.sourceforge.net/

Q-Chem begins on Mon Jun 2 13:47:27 2025

Scratch files written to C:/Users/hille/AppData/Local/Temp/WF0E714C87DACF7CA2//scratch///

Processing default memory

... MEM_TOTAL 4076 MB (default) [16 cores]

Processing $rem in C:/Program Files/Wavefunction/Spartan24v130/P4e//../auxdir/config/preferences:

(site specific preferences)

... THRESH 9

... SMALL_PROD_XCMAT 9

```
 ... BASIS_LIN_DEP_THRESH     5
 ... SCF_ALGORITHM          DIIS_GDM
 ... MAXSCF              250
 ... MAXDIIS          45
 ... THRESHDIIS        -1  (i.e. don't switch on delta-E)
 ... ECP_FIT          TRUE (Convert deprecated ECP files)
 ... GUI             GUI_SPARTAN
 ... TERSE_OUTPUT     TRUE !turn on spartan printing
 ... SCF_CONVERGENCE  7
 ... CCMAN2 FALSE  (qc4.3)
 ... SYMMETRY   FALSE  ! turn of symmetry for spartan16
 ... SYM_IGNORE TRUE   ! ..use FORCESYMMETRY to override
 ... GEOM_OPT_TOL_GRADIENT       700  ! loosen tolernaces for organic geometries
 ... GEOM_OPT_TOL_DISPLACEMENT  1400  ! was 1200 = .0012
 ... GEOM_OPT_TOL_ENERGY       2000  ! was  100 = .000 001
 ... GEN_SCFMAN  FALSE
Processing $rem in input file
 ... JOBTYPE    OPT
 ... TIDY_SYM   TRUE
 ... METHOD            WB97X-D
 ... xc_grid           75000302  (75,302)
 ... BASIS             6-311+G**
 ... THRESH             12  #diffuse default
 ... MAXSCF            350  #diffuse default
 ... VARTHRESH       2    (default DFT)
 ... INCDFT            TRUE (default DFT)
 ... GEOM_OPT_HESSIAN      READ (main opt)
 ... EXTERNAL_HESSIAN      1
 ... GUI            GUI_SPARTAN
```

... TERSE_OUTPUT TRUE

NAlpha2: 130

NElect 130

Mult 1

Checking the input file for inconsistencies... ...done.

User input:

```
$comment
Cation Optimization
$end
$molecule
 1 1
   7     2.044588203    0.66762810918     5.0945765221
   7    2.0507217036    0.69383680629     2.9248920394
   6    1.2749100021     0.7334327491      4.006793847
   1   0.19734764532    0.80744361587     4.0026219037
   6    3.3620230505    0.59534427493     3.3327723003
   1     4.176714474    0.54674218358     2.6266058642
   6    3.3598519571    0.58028263473     4.6930427999
   1    4.1711384402    0.52268440338     5.4024018127
   6    1.5788701969     0.6971017519     6.4830835099
   1    1.8905854961    -0.2187264142     6.9870434299
   1   0.49103803124    0.76358047615      6.489371785
   1    2.0001513646     1.5683600318     6.9865231544
   6    1.5776011636    0.67669884864     1.5274032419
   1   0.59393821556     1.1494789229     1.5253802064
   1    2.2558433127     1.3153357493    0.95467344744
```

6	1.537760483	-0.74402362779	0.96844647859
1	0.80405388957	-1.3332212237	1.5357775174
1	2.5171022747	-1.2126933727	1.1299624858
6	1.1941424077	-0.80291246937	-0.53090718754
1	1.9712981646	-0.24437390331	-1.0750689878
6	1.2493396137	-2.2623901673	-0.99394518544
1	2.1912610884	-2.7375476288	-0.6982643376
1	0.42581899588	-2.841044237	-0.55702174134
1	1.1729937258	-2.3401211102	-2.0814552246
6	-0.16406953072	-0.14824938592	-0.82882581768
1	-0.13288268364	0.91438623751	-0.54810120133
1	-0.93802902679	-0.61503315955	-0.19998121108
6	-0.60817039396	-0.22368469474	-2.293027818
1	0.1961864546	0.15330444553	-2.9411024796
1	-0.76976975441	-1.26955577	-2.5722781224
6	-1.8732240711	0.57180124633	-2.5278534426
1	-1.7680895422	1.6287745104	-2.2779298003
6	-3.2316230085	-0.029739380359	-2.2288656749
1	-3.9838133402	0.5858876076	-1.7417258463
1	-3.2588249708	-1.0793353582	-1.9405383825
6	-2.8440868821	0.29221065712	-3.6517653255
6	-3.5016408915	1.4949915236	-4.3013906621
1	-3.6685367547	2.3021966007	-3.5793875967
1	-2.8763526085	1.8909752598	-5.1108295634
1	-4.4737037784	1.2224555957	-4.7290446002
6	-2.5906234426	-0.84767723477	-4.6200374651
1	-2.2118615792	-1.747452467	-4.125749625
1	-3.5228691721	-1.1237966634	-5.1261857813
1	-1.8671089234	-0.55335597384	-5.3900892657

```
$end
$rem
JOBTYPE    OPT
TIDY_SYM   TRUE
METHOD       WB97X-D
xc_grid          75000302  (75,302)
BASIS            6-311+G**
THRESH          12  #diffuse default
MAXSCF        350  #diffuse default
VARTHRESH            2    (default DFT)
INCDFT          TRUE (default DFT)
GEOM_OPT_HESSIAN      READ (main opt)
EXTERNAL_HESSIAN      1
GUI                  GUI_SPARTAN
TERSE_OUTPUT       TRUE
$end
$opt
$end
-----------------------------------------------------------------
           Standard Nuclear Orientation (Angstroms)
   I     Atom          X               Y               Z
 -----------------------------------------------------------------
    1     N      2.0445882030     0.6676281092     5.0945765221
    2     N      2.0507217036     0.6938368063     2.9248920394
    3     C      1.2749100021     0.7334327491     4.0067938470
    4     H      0.1973476453     0.8074436159     4.0026219037
    5     C      3.3620230505     0.5953442749     3.3327723003
    6     H      4.1767144740     0.5467421836     2.6266058642
    7     C      3.3598519571     0.5802826347     4.6930427999
```

8	H	4.1711384402	0.5226844034	5.4024018127
9	C	1.5788701969	0.6971017519	6.4830835099
10	H	1.8905854961	-0.2187264142	6.9870434299
11	H	0.4910380312	0.7635804762	6.4893717850
12	H	2.0001513646	1.5683600318	6.9865231544
13	C	1.5776011636	0.6766988486	1.5274032419
14	H	0.5939382156	1.1494789229	1.5253802064
15	H	2.2558433127	1.3153357493	0.9546734474
16	C	1.5377604830	-0.7440236278	0.9684464786
17	H	0.8040538896	-1.3332212237	1.5357775174
18	H	2.5171022747	-1.2126933727	1.1299624858
19	C	1.1941424077	-0.8029124694	-0.5309071875
20	H	1.9712981646	-0.2443739033	-1.0750689878
21	C	1.2493396137	-2.2623901673	-0.9939451854
22	H	2.1912610884	-2.7375476288	-0.6982643376
23	H	0.4258189959	-2.8410442370	-0.5570217413
24	H	1.1729937258	-2.3401211102	-2.0814552246
25	C	-0.1640695307	-0.1482493859	-0.8288258177
26	H	-0.1328826836	0.9143862375	-0.5481012013
27	H	-0.9380290268	-0.6150331596	-0.1999812111
28	C	-0.6081703940	-0.2236846947	-2.2930278180
29	H	0.1961864546	0.1533044455	-2.9411024796
30	H	-0.7697697544	-1.2695557700	-2.5722781224
31	C	-1.8732240711	0.5718012463	-2.5278534426
32	H	-1.7680895422	1.6287745104	-2.2779298003
33	C	-3.2316230085	-0.0297393804	-2.2288656749
34	H	-3.9838133402	0.5858876076	-1.7417258463
35	H	-3.2588249708	-1.0793353582	-1.9405383825
36	C	-2.8440868821	0.2922106571	-3.6517653255

37	C	-3.5016408915	1.4949915236	-4.3013906621
38	H	-3.6685367547	2.3021966007	-3.5793875967
39	H	-2.8763526085	1.8909752598	-5.1108295634
40	H	-4.4737037784	1.2224555957	-4.7290446002
41	C	-2.5906234426	-0.8476772348	-4.6200374651
42	H	-2.2118615792	-1.7474524670	-4.1257496250
43	H	-3.5228691721	-1.1237966634	-5.1261857813
44	H	-1.8671089234	-0.5533559738	-5.3900892657

\--

Nuclear Repulsion Energy = 1184.89457808 hartrees

There are 65 alpha and 65 beta electrons

Requested basis set is 6-311+G(d,p)

There are 210 shells and 536 basis functions

Total QAlloc Memory Limit 4076 MB

Mega-Array Size 188 MB

MEM_STATIC part 192 MB

.. (5.2.P)

\---

\- Entering fldman on Mon Jun 2 13:47:27 2025 -

\---

A cutoff of 1.0D-12 yielded 14734 shell pairs

There are 99287 function pairs (105038 Cartesian)

Smallest overlap matrix eigenvalue = 3.26E-06

Linear dependence detected in AO basis

Tighter screening thresholds may be required for diffuse basis sets

Use S2THRESH > 12 and THRESH = 14 in case of SCF convergence issues

Number of orthogonalized atomic orbitals = 533

Maximum deviation from orthogonality = 1.899E-11

Scale SEOQF with 1.000000e-01/1.000000e-01/1.000000e-02

Standard Electronic Orientation quadrupole field applied

Nucleus-field energy = 0.0000000163 hartrees

- Entering gesman on Mon Jun 2 13:47:27 2025 -

Guess from superposition of atomic densities

Warning: Energy on first SCF cycle will be non-variational

SAD guess density has 131.000000 electrons

- Entering scfman on Mon Jun 2 13:47:27 2025 -

Long-range K will be added via erf

Coulomb attenuation parameter = 0.2 bohr**(-1)

A restricted hybrid HF-DFT SCF calculation will be

performed using Pulay DIIS + Geometric Direct Minimization

Exchange: 0.2220 Hartree-Fock + 1.0000 wB97X-D + LR-HF

Correlation: 1.0000 wB97X-D

Using Euler-Maclaurin-Lebedev (75,302) quadrature formula

Dispersion: Grimme D

SCF converges when RMS gradient is below 1.0E-07

Exchange: 0.2220 Hartree-Fock + 1.0000 wB97X-D + LR-HF

Correlation: 1.0000 wB97X-D

Using Euler-Maclaurin-Lebedev (75,302) quadrature formula

Dispersion: Grimme D

using 6 threads for integral computing

OpenMP Integral computing Module

Release: version 1.0, May 2013, Q-Chem Inc. Pittsburgh

----

using 6 threads for integral computing

OpenMP Integral computing Module

Release: version 1.0, May 2013, Q-Chem Inc. Pittsburgh

Cycle	Energy	DIIS Error
1	-702.7348756554	2.80E-02
2	-696.9506058586	1.92E-03
3	-697.0221332709	1.48E-03
4	-697.0969119314	2.01E-04
5	-697.0980075950	1.12E-04
6	-697.0984059755	1.82E-05
7	-697.0984157966	8.53E-06
8	-697.0984180570	2.73E-06
9	-697.0984183181	8.23E-07
10	-697.0984183512	2.22E-07
11	-697.0984183554	9.44E-08 Convergence criterion met

SCF time: CPU 550.48 s wall 94.10 s

SCF energy in the final basis set = -697.09841836

Total energy in the final basis set = -697.09841836

- Entering anlman on Mon Jun 2 13:49:01 2025 -

Orbital Energies (a.u.)

Alpha MOs

-- Occupied --

```
-14.6917 -14.6864 -10.5481 -10.4978 -10.4970 -10.4880 -10.4813 -10.4144
-10.3975 -10.3748 -10.3654 -10.3616 -10.3612 -10.3561 -10.3439 -10.3338
-10.3313  -1.3434  -1.2219  -1.0688  -1.0452  -1.0390  -1.0130  -0.9717
 -0.9333  -0.9028  -0.8827  -0.8798  -0.8629  -0.8490  -0.8302  -0.8040
 -0.7681  -0.7503  -0.7392  -0.7279  -0.7227  -0.7092  -0.7003  -0.6932
 -0.6878  -0.6750  -0.6530  -0.6513  -0.6362  -0.6313  -0.6103  -0.6014
 -0.5895  -0.5793  -0.5698  -0.5611  -0.5513  -0.5414  -0.5335  -0.5302
 -0.5229  -0.5168  -0.5095  -0.5043  -0.4997  -0.4938  -0.4778  -0.4237
 -0.4098
```

-- Virtual --

```
 -0.1106  -0.0770  -0.0635  -0.0445  -0.0438  -0.0303  -0.0271  -0.0229
 -0.0171  -0.0070  -0.0065  -0.0039   0.0039   0.0098   0.0116   0.0172
```

0.0233	0.0248	0.0271	0.0366	0.0381	0.0389	0.0444	0.0487
0.0504	0.0546	0.0551	0.0614	0.0664	0.0718	0.0744	0.0762
0.0775	0.0829	0.0860	0.0885	0.0920	0.0937	0.0979	0.1004
0.1053	0.1087	0.1125	0.1159	0.1174	0.1192	0.1256	0.1274
0.1306	0.1318	0.1365	0.1401	0.1415	0.1441	0.1469	0.1495
0.1533	0.1558	0.1575	0.1633	0.1639	0.1690	0.1710	0.1716
0.1765	0.1800	0.1817	0.1874	0.1889	0.1928	0.1947	0.1966
0.2035	0.2080	0.2092	0.2137	0.2187	0.2208	0.2232	0.2270
0.2309	0.2321	0.2371	0.2413	0.2458	0.2480	0.2518	0.2557
0.2609	0.2636	0.2664	0.2703	0.2729	0.2796	0.2853	0.2886
0.2933	0.2977	0.3044	0.3067	0.3149	0.3169	0.3279	0.3371
0.3475	0.3501	0.3541	0.3599	0.3683	0.3723	0.3772	0.3811
0.3887	0.4007	0.4132	0.4223	0.4276	0.4289	0.4383	0.4554
0.4647	0.4691	0.4772	0.4787	0.4828	0.4950	0.4985	0.5129
0.5186	0.5255	0.5284	0.5344	0.5418	0.5445	0.5498	0.5564
0.5671	0.5780	0.5852	0.5873	0.5974	0.6000	0.6040	0.6112
0.6171	0.6190	0.6215	0.6301	0.6345	0.6414	0.6550	0.6642
0.6662	0.6709	0.6756	0.6780	0.6799	0.6813	0.6862	0.6903
0.6936	0.6985	0.7040	0.7081	0.7101	0.7155	0.7224	0.7250
0.7309	0.7356	0.7392	0.7421	0.7466	0.7539	0.7573	0.7607
0.7702	0.7765	0.7874	0.7938	0.7967	0.8043	0.8089	0.8111
0.8399	0.8538	0.8653	0.8700	0.8839	0.9026	0.9075	0.9165
0.9316	0.9389	0.9465	0.9700	0.9821	0.9838	0.9913	1.0013
1.0054	1.0306	1.0533	1.0540	1.0684	1.0833	1.0861	1.1027
1.1118	1.1206	1.1435	1.1592	1.1830	1.1987	1.2149	1.2387
1.2562	1.2611	1.2831	1.3120	1.3304	1.3400	1.3455	1.3695
1.3705	1.3761	1.3847	1.4072	1.4195	1.4243	1.4321	1.4461
1.4554	1.4600	1.4634	1.4688	1.4782	1.4906	1.4975	1.5038
1.5144	1.5274	1.5341	1.5406	1.5458	1.5522	1.5531	1.5628

1.5651 1.5753 1.5771 1.5850 1.5972 1.6009 1.6068 1.6104

1.6143 1.6257 1.6366 1.6409 1.6457 1.6516 1.6544 1.6602

1.6719 1.6792 1.6874 1.6938 1.7034 1.7049 1.7127 1.7144

1.7257 1.7300 1.7330 1.7372 1.7443 1.7508 1.7567 1.7699

1.7743 1.7865 1.7893 1.7981 1.8204 1.8247 1.8350 1.8422

1.8478 1.8623 1.8911 1.8984 1.9176 1.9246 1.9357 1.9537

1.9634 1.9820 1.9925 2.0006 2.0166 2.0324 2.0442 2.0460

2.0508 2.0645 2.0802 2.0897 2.1008 2.1123 2.1202 2.1329

2.1376 2.1428 2.1547 2.1688 2.1782 2.1843 2.1937 2.1991

2.2144 2.2297 2.2540 2.2572 2.2682 2.2921 2.3051 2.3280

2.3362 2.3538 2.3685 2.3759 2.3808 2.4032 2.4144 2.4244

2.4310 2.4511 2.4540 2.4650 2.4710 2.4794 2.4860 2.4892

2.4933 2.5051 2.5059 2.5147 2.5188 2.5232 2.5322 2.5418

2.5516 2.5590 2.5705 2.5720 2.5787 2.5838 2.5958 2.6041

2.6067 2.6160 2.6206 2.6342 2.6425 2.6551 2.6592 2.6713

2.6808 2.6891 2.6968 2.7137 2.7208 2.7303 2.7338 2.7384

2.7468 2.7581 2.7733 2.7829 2.7992 2.8093 2.8178 2.8339

2.8418 2.8537 2.8620 2.8785 2.8826 2.9027 2.9127 2.9273

2.9310 2.9363 2.9511 2.9629 2.9679 2.9956 3.0096 3.0316

3.0362 3.0677 3.0740 3.0878 3.1362 3.1567 3.1823 3.2294

3.2602 3.2949 3.3435 3.3542 3.4531 3.5033 3.5222 3.5388

3.5886 3.6241 3.6363 3.6670 3.6788 3.6969 3.7517 3.7578

3.7851 3.7962 3.8134 3.8182 3.8222 3.8420 3.8479 3.8576

3.8621 3.8660 3.8771 3.8786 3.8920 3.9981 4.0541 4.0765

4.1390 4.1936 4.3074 4.3412 4.3761 4.5474 4.6363 4.6753

4.8741 4.9159 5.2127 23.7212 23.8420 23.8745 23.8771 23.8894

23.8947 23.9158 23.9309 23.9569 23.9686 24.0090 24.0252 24.0515

24.0605 24.1521 35.4349 35.5884

Ground-State Mulliken Net Atomic Charges

Atom	Charge (a.u.)
1 N	-0.002555
2 N	0.032502
3 C	-0.085520
4 H	0.216544
5 C	-0.133445
6 H	0.216643
7 C	-0.099743
8 H	0.213607
9 C	-0.264249
10 H	0.226157
11 H	0.193824
12 H	0.228445
13 C	-0.310455
14 H	0.190923
15 H	0.224708
16 C	-0.325240
17 H	0.191542
18 H	0.172198
19 C	0.006865
20 H	0.183311
21 C	-0.710868
22 H	0.157854
23 H	0.188491
24 H	0.186608

25 C	-0.282293
26 H	0.136631
27 H	0.178648
28 C	-0.344486
29 H	0.175657
30 H	0.137287
31 C	-0.059640
32 H	0.145022
33 C	-0.624014
34 H	0.170651
35 H	0.165496
36 C	0.390411
37 C	-0.497425
38 H	0.139625
39 H	0.166793
40 H	0.167524
41 C	-0.535788
42 H	0.129176
43 H	0.171236
44 H	0.171346

Sum of atomic charges = 1.000000

Cartesian Multipole Moments

Charge (ESU x 10^10)

4.8032

Dipole Moment (Debye)

X	8.4074	Y	2.4230	Z	16.7950
Tot	18.9375				

Quadrupole Moments (Debye-Ang)

XX	-74.7718	XY	4.2324	YY	-102.1576
XZ	38.4673	YZ	12.9569	ZZ	-3.0504

Traceless Quadrupole Moments (Debye-Ang)

QXX	-44.3357	QYY	-126.4929	QZZ	170.8286
QXY	12.6972	QXZ	115.4020	QYZ	38.8707

Octopole Moments (Debye-Ang^2)

XXX	52.4063	XXY	9.2896	XYY	-17.2355
YYY	-24.8230	XXZ	78.4861	XYZ	22.5252
YYZ	-20.8362	XZZ	159.6516	YZZ	57.6604
ZZZ	475.1178				

Traceless Octopole Moments (Debye-Ang^2)

XXX	-967.3069	YYY	-751.4879	ZZZ	2331.8575
XXY	12.9630	XXZ	-421.0116	XYY	-842.9995
XYZ	337.8776	XZZ	1810.3064	YYZ	-1910.8459
YZZ	738.5250				

Hexadecapole Moments (Debye-Ang^3)

XXXX	-2904.2781	XXXY	71.5715	XXYY	-679.4469
XYYY	29.0769	YYYY	-747.2845	XXXZ	-1601.6283
XXYZ	-37.5273	XYYZ	-682.7258	YYYZ	-282.0862
XXZZ	-1222.8456	XYZZ	125.1692	YYZZ	-1361.7384
XZZZ	-874.7777	YZZZ	96.3984	ZZZZ	-4061.9514

Traceless Hexadecapole Moments (Debye-Ang^3)

XXXX	-532.0319	XXXY	-2646.7822	XXXZ	-26010.0444
XXYY	-141.0445	XXYZ	-586.1623	XXZZ	673.0764
XYYY	-7108.7167	XYYZ	-24299.2315	XYZZ	9755.4989
XZZZ	50309.2759	YYYY	44323.2273	YYYZ	-19556.4452

YYZZ -44182.1828 YZZZ 20142.6075 ZZZZ 43509.1065

- Entering drvman on Mon Jun 2 13:49:01 2025 -

Calculating analytic gradient of the SCF energy

Gradient of SCF Energy

	1	2	3	4	5	6
1	0.0003247	0.0005120	-0.0018250	-0.0014663	0.0003449	0.0010843
2	-0.0000549	-0.0001550	0.0002840	0.0000677	0.0005149	-0.0000731
3	0.0021879	-0.0023945	0.0000738	-0.0000075	-0.0021780	-0.0011739

	7	8	9	10	11	12
1	0.0004471	0.0010256	0.0000003	0.0004106	-0.0011307	0.0005412
2	-0.0002980	-0.0000634	-0.0000041	-0.0010137	0.0000755	0.0009603
3	0.0023292	0.0011990	0.0003817	0.0003710	-0.0001491	0.0003626

	13	14	15	16	17	18
1	-0.0002171	-0.0008553	0.0008815	0.0000330	-0.0007803	0.0012220
2	-0.0001326	0.0005847	0.0006512	-0.0002482	-0.0006073	-0.0004516
3	-0.0003190	0.0004144	-0.0003486	0.0003309	0.0006549	0.0002150

	19	20	21	22	23	24
1	-0.0003149	0.0009714	-0.0002742	0.0012492	-0.0010138	0.0000421
2	0.0003534	0.0005164	0.0004888	-0.0006868	-0.0007806	-0.0002071
3	-0.0003040	-0.0006405	0.0003941	0.0003134	0.0004658	-0.0015526

	25	26	27	28	29	30
1	0.0000983	0.0001061	-0.0009810	0.0000963	0.0010220	-0.0001093
2	-0.0003147	0.0013014	-0.0005371	0.0002234	0.0004277	-0.0013834
3	-0.0002106	0.0001927	0.0006892	0.0001170	-0.0008548	-0.0001599

	31	32	33	34	35	36
1	-0.0004888	0.0004211	0.0008831	-0.0013681	-0.0003511	0.0001284
2	-0.0004588	0.0016229	0.0002728	0.0008630	-0.0016643	-0.0001985
3	-0.0001653	0.0005583	-0.0007667	0.0009757	0.0007225	0.0004643

	37	38	39	40	41	42
1	0.0001085	-0.0003078	0.0007910	-0.0012984	-0.0002229	0.0004690
2	-0.0005037	0.0012477	0.0006356	-0.0002466	0.0005506	-0.0013682
3	-0.0001712	0.0008611	-0.0010419	-0.0005239	-0.0001474	0.0004898

	43	44
1	-0.0012271	0.0010184
2	-0.0004631	0.0002726
3	-0.0006869	-0.0009680

Max gradient component = 2.395E-03

RMS gradient = 8.029E-04

Gradient time: CPU 194.06 s wall 35.32 s

- Entering optman on Mon Jun 2 13:49:37 2025 -

Geometry Optimization Parameters

NAtoms,	NIC,	NZ,	NCons,	NDum,	NFix,	NCnnct,	MaxDiis
44	348	0	0	0	0	0	0

Cartesian Hessian read from HESS file

** GEOMETRY OPTIMIZATION IN DELOCALIZED INTERNAL COORDINATES **

Searching for a Minimum

Optimization Cycle: 1

	Coordinates (Angstroms)		
ATOM	X	Y	Z
1 N	2.0445882030	0.6676281092	5.0945765221
2 N	2.0507217036	0.6938368063	2.9248920394
3 C	1.2749100021	0.7334327491	4.0067938470
4 H	0.1973476453	0.8074436159	4.0026219037
5 C	3.3620230505	0.5953442749	3.3327723003
6 H	4.1767144740	0.5467421836	2.6266058642
7 C	3.3598519571	0.5802826347	4.6930427999
8 H	4.1711384402	0.5226844034	5.4024018127
9 C	1.5788701969	0.6971017519	6.4830835099
10 H	1.8905854961	-0.2187264142	6.9870434299
11 H	0.4910380312	0.7635804762	6.4893717850
12 H	2.0001513646	1.5683600318	6.9865231544
13 C	1.5776011636	0.6766988486	1.5274032419
14 H	0.5939382156	1.1494789229	1.5253802064
15 H	2.2558433127	1.3153357493	0.9546734474
16 C	1.5377604830	-0.7440236278	0.9684464786
17 H	0.8040538896	-1.3332212237	1.5357775174
18 H	2.5171022747	-1.2126933727	1.1299624858
19 C	1.1941424077	-0.8029124694	-0.5309071875
20 H	1.9712981646	-0.2443739033	-1.0750689878
21 C	1.2493396137	-2.2623901673	-0.9939451854
22 H	2.1912610884	-2.7375476288	-0.6982643376
23 H	0.4258189959	-2.8410442370	-0.5570217413
24 H	1.1729937258	-2.3401211102	-2.0814552246
25 C	-0.1640695307	-0.1482493859	-0.8288258177

26	H	-0.1328826836	0.9143862375	-0.5481012013
27	H	-0.9380290268	-0.6150331596	-0.1999812111
28	C	-0.6081703940	-0.2236846947	-2.2930278180
29	H	0.1961864546	0.1533044455	-2.9411024796
30	H	-0.7697697544	-1.2695557700	-2.5722781224
31	C	-1.8732240711	0.5718012463	-2.5278534426
32	H	-1.7680895422	1.6287745104	-2.2779298003
33	C	-3.2316230085	-0.0297393804	-2.2288656749
34	H	-3.9838133402	0.5858876076	-1.7417258463
35	H	-3.2588249708	-1.0793353582	-1.9405383825
36	C	-2.8440868821	0.2922106571	-3.6517653255
37	C	-3.5016408915	1.4949915236	-4.3013906621
38	H	-3.6685367547	2.3021966007	-3.5793875967
39	H	-2.8763526085	1.8909752598	-5.1108295634
40	H	-4.4737037784	1.2224555957	-4.7290446002
41	C	-2.5906234426	-0.8476772348	-4.6200374651
42	H	-2.2118615792	-1.7474524670	-4.1257496250
43	H	-3.5228691721	-1.1237966634	-5.1261857813
44	H	-1.8671089234	-0.5533559738	-5.3900892657

Point Group: c1   Number of degrees of freedom: 126

Energy is -697.098418355

Attempting to generate delocalized internal coordinates

Transforming Cartesian Hessian to Internal Coordinates

Hessian Transformation does not Include Derivative of B-matrix

internal optimization (0)

126 Hessian modes will be used to form the next step

Hessian Eigenvalues:

0.000191	0.000680	0.001517	0.001625	0.001889	0.002162
0.002486	0.002991	0.003344	0.003935	0.008183	0.009004
0.012646	0.013737	0.014907	0.016095	0.017385	0.021396
0.021618	0.023648	0.025756	0.033370	0.034757	0.037559
0.039341	0.040087	0.040301	0.040938	0.044546	0.045770
0.046174	0.046809	0.046982	0.047625	0.047734	0.048622
0.048783	0.049221	0.049560	0.050773	0.051016	0.053170
0.061968	0.063024	0.065856	0.069052	0.072955	0.074790
0.074966	0.078266	0.082326	0.083627	0.105855	0.106445
0.107705	0.110850	0.119385	0.126213	0.127310	0.130053
0.131780	0.134477	0.137682	0.140557	0.147211	0.149402
0.151227	0.151715	0.154255	0.155358	0.166608	0.173652
0.178634	0.183988	0.184547	0.194454	0.196318	0.202257
0.214623	0.231078	0.242022	0.246188	0.248688	0.267092
0.284435	0.287844	0.300800	0.305126	0.307843	0.313418
0.324265	0.325604	0.327240	0.330011	0.333675	0.334768
0.337885	0.340099	0.340598	0.341135	0.341261	0.341917
0.344969	0.345363	0.346470	0.348732	0.349137	0.349785
0.351374	0.352715	0.353245	0.356118	0.356501	0.356823
0.358150	0.359733	0.363486	0.364364	0.387411	0.388277
0.389352	0.394502	0.430956	0.466147	0.502938	0.604016

WARNING Magnitude of eigenvalue 1 too small. Replaced by 0.001000

WARNING Magnitude of eigenvalue 2 too small. Replaced by 0.001000

Minimum search - taking simple RFO step

Searching for Lamda that Minimizes Along All modes

Value Taken   Lamda = -0.00038928

Step Taken. Stepsize is 0.211122 0.014472

	Maximum	Tolerance	Cnvgd?
Gradient	0.003748	0.000700	NO
Displacement	0.079520	0.001400	NO
Energy change	*********	0.000020	NO

New Cartesian Coordinates Obtained by Inverse Iteration

Displacement from previous Coordinates is: 0.935627

Standard Nuclear Orientation (Angstroms)

I	Atom	X	Y	Z
1	N	2.0187164159	0.6841379198	5.0839680195
2	N	2.0613311889	0.6693221279	2.9187364149
3	C	1.2755061447	0.7878256055	3.9839824465
4	H	0.2091861763	0.9452098637	3.9599440940
5	C	3.3530832491	0.4762281066	3.3505448157
6	H	4.1719521231	0.3517422424	2.6620583840
7	C	3.3282885166	0.4885887710	4.7068837832
8	H	4.1206669941	0.3805562292	5.4284030137
9	C	1.5328099161	0.7760200944	6.4608706111
10	H	1.7642247510	-0.1479718774	6.9891632043
11	H	0.4546360854	0.9234666760	6.4462752230
12	H	2.0073294408	1.6217843380	6.9569559091
13	C	1.6123815992	0.6667428644	1.5153648429
14	H	0.6371960125	1.1530136526	1.4983123710

15	H	2.3065506956	1.2959901786	0.9543367882
16	C	1.5577327763	-0.7478488845	0.9486928306
17	H	0.8213606195	-1.3333061438	1.5129294256
18	H	2.5307574407	-1.2270668668	1.1042445682
19	C	1.2101038663	-0.7931881200	-0.5470849696
20	H	1.9727654032	-0.2143312256	-1.0867293962
21	C	1.2871190732	-2.2427284788	-1.0307793196
22	H	2.2417079037	-2.7009673023	-0.7582619225
23	H	0.4848779033	-2.8425892383	-0.5879443476
24	H	1.1923336460	-2.3069008476	-2.1153439951
25	C	-0.1590042263	-0.1582413073	-0.8276490981
26	H	-0.1509284927	0.8947285679	-0.5185843601
27	H	-0.9212587079	-0.6569222899	-0.2127924452
28	C	-0.6007767330	-0.2018078554	-2.2914312651
29	H	0.1939548987	0.2098429000	-2.9265801834
30	H	-0.7399867161	-1.2398062535	-2.6017390411
31	C	-1.8800482174	0.5712232353	-2.5068361559
32	H	-1.7962418683	1.6247894157	-2.2460597176
33	C	-3.2239420045	-0.0594029711	-2.2075967511
34	H	-3.9824981658	0.5348683605	-1.7101173494
35	H	-3.2272942842	-1.1108543082	-1.9352276621
36	C	-2.8511645557	0.2869362075	-3.6273552584
37	C	-3.5337497643	1.4830782924	-4.2572771366
38	H	-3.7110485657	2.2752986943	-3.5247945169
39	H	-2.9215586908	1.8996501216	-5.0634284551
40	H	-4.5007317830	1.1976316377	-4.6826465548
41	C	-2.5818510218	-0.8367880234	-4.6064627229
42	H	-2.1797961906	-1.7299730264	-4.1240962334
43	H	-3.5106589062	-1.1280728558	-5.1056736435
44	H	-1.8740339464	-0.5199082269	-5.3791742442

Nuclear Repulsion Energy = 1186.83557036 hartrees

There are 65 alpha and 65 beta electrons

- Entering fldman on Mon Jun 2 13:49:37 2025 -

Applying Cartesian multipole field

Component	Value
---------	-----
(2,0,0)	1.00000E-11
(0,2,0)	2.00000E-11
(0,0,2)	-3.00000E-12

Nucleus-field energy = 0.0000000163 hartrees

- Entering gesman on Mon Jun 2 13:49:37 2025 -

Requested basis set is 6-311+G(d,p)

There are 210 shells and 536 basis functions

A cutoff of 1.0D-12 yielded 14757 shell pairs

There are 99431 function pairs (105188 Cartesian)

Smallest overlap matrix eigenvalue = 3.12E-06

Linear dependence detected in AO basis

Tighter screening thresholds may be required for diffuse basis sets

Use S2THRESH > 12 and THRESH = 14 in case of SCF convergence issues

Number of orthogonalized atomic orbitals = 533

Maximum deviation from orthogonality = 2.738E-11

Guess MOs from SCF MO coefficient file

Reading MOs from coefficient file

Reading MOs from coefficient file

```
 -  Entering scfman on Mon Jun  2 13:49:37 2025  -
-----------------------------------------------
 Long-range K will be added via erf
 Coulomb attenuation parameter = 0.2 bohr**(-1)
 A restricted hybrid HF-DFT SCF calculation will be
 performed using Pulay DIIS + Geometric Direct Minimization
 Exchange:     0.2220 Hartree-Fock + 1.0000 wB97X-D + LR-HF
 Correlation:  1.0000 wB97X-D
 Using Euler-Maclaurin-Lebedev (75,302) quadrature formula
 Dispersion:   Grimme D
 SCF converges when RMS gradient is below 1.0E-07
 Geometry optimization detected.  Setting ReadMinima to 0
 Setting SaveMinima to 0
 using 6 threads for integral computing
 -------------------------------------------------------
 OpenMP Integral computing Module
 Release: version 1.0, May 2013, Q-Chem Inc. Pittsburgh
 -------------------------------------------------------
 using 6 threads for integral computing
 -------------------------------------------------------
 OpenMP Integral computing Module
 Release: version 1.0, May 2013, Q-Chem Inc. Pittsburgh
 -------------------------------------------------------
 ---------------------------------------
  Cycle       Energy         DIIS Error
 ---------------------------------------
    1    -697.1096313287      9.15E-04
    2    -697.0976342495      1.00E-04
    3    -697.0985127490      4.42E-05
    4    -697.0985972239      2.58E-05
```

5 -697.0986244686 6.53E-06

6 -697.0986260697 2.47E-06

7 -697.0986262784 9.24E-07

8 -697.0986263117 3.45E-07

9 -697.0986263161 7.20E-08 Convergence criterion met

SCF time: CPU 450.53 s wall 76.77 s

SCF energy in the final basis set = -697.09862632

Total energy in the final basis set = -697.09862632

- Entering anlman on Mon Jun 2 13:50:54 2025 -

Orbital Energies (a.u.)

Alpha MOs

-- Occupied --

-14.6914 -14.6862 -10.5476 -10.4971 -10.4962 -10.4873 -10.4807 -10.4136

-10.3967 -10.3739 -10.3646 -10.3609 -10.3603 -10.3554 -10.3432 -10.3330

-10.3305 -1.3452 -1.2228 -1.0700 -1.0465 -1.0402 -1.0139 -0.9724

-0.9342 -0.9041 -0.8833 -0.8802 -0.8640 -0.8500 -0.8307 -0.8051

-0.7692 -0.7512 -0.7398 -0.7287 -0.7233 -0.7099 -0.7013 -0.6937

-0.6878 -0.6761 -0.6535 -0.6519 -0.6369 -0.6322 -0.6105 -0.6020

-0.5902 -0.5800 -0.5708 -0.5605 -0.5520 -0.5417 -0.5338 -0.5305

-0.5236 -0.5174 -0.5098 -0.5052 -0.5000 -0.4943 -0.4782 -0.4237

-0.4098

-- Virtual --

-0.1098 -0.0765 -0.0635 -0.0445 -0.0436 -0.0300 -0.0270 -0.0229

-0.0171 -0.0069 -0.0064 -0.0040 0.0044 0.0097 0.0117 0.0171

0.0238	0.0249	0.0273	0.0368	0.0384	0.0388	0.0450	0.0494
0.0506	0.0543	0.0554	0.0608	0.0657	0.0719	0.0752	0.0764
0.0780	0.0830	0.0856	0.0894	0.0920	0.0930	0.0978	0.1008
0.1048	0.1088	0.1123	0.1166	0.1182	0.1201	0.1252	0.1285
0.1319	0.1329	0.1371	0.1402	0.1415	0.1443	0.1475	0.1495
0.1556	0.1568	0.1579	0.1630	0.1652	0.1697	0.1714	0.1719
0.1768	0.1807	0.1820	0.1870	0.1895	0.1929	0.1950	0.1969
0.2045	0.2080	0.2098	0.2145	0.2197	0.2212	0.2244	0.2269
0.2307	0.2323	0.2378	0.2416	0.2467	0.2488	0.2517	0.2570
0.2613	0.2650	0.2669	0.2699	0.2727	0.2806	0.2865	0.2888
0.2937	0.2981	0.3044	0.3070	0.3153	0.3177	0.3295	0.3393
0.3482	0.3497	0.3532	0.3612	0.3681	0.3734	0.3775	0.3824
0.3882	0.4022	0.4150	0.4222	0.4284	0.4299	0.4379	0.4555
0.4659	0.4688	0.4777	0.4792	0.4813	0.4951	0.4970	0.5125
0.5184	0.5255	0.5309	0.5338	0.5422	0.5467	0.5499	0.5555
0.5668	0.5785	0.5854	0.5885	0.5967	0.5999	0.6059	0.6139
0.6177	0.6197	0.6232	0.6313	0.6351	0.6419	0.6546	0.6654
0.6679	0.6718	0.6764	0.6785	0.6798	0.6829	0.6869	0.6903
0.6942	0.6998	0.7040	0.7083	0.7111	0.7172	0.7229	0.7252
0.7301	0.7381	0.7399	0.7432	0.7461	0.7542	0.7576	0.7632
0.7717	0.7779	0.7880	0.7927	0.7977	0.8029	0.8115	0.8134
0.8392	0.8537	0.8647	0.8691	0.8832	0.9052	0.9083	0.9192
0.9326	0.9395	0.9467	0.9702	0.9807	0.9820	0.9919	1.0009
1.0073	1.0312	1.0538	1.0572	1.0696	1.0839	1.0867	1.1045
1.1119	1.1244	1.1421	1.1576	1.1841	1.1986	1.2138	1.2384
1.2556	1.2626	1.2830	1.3117	1.3319	1.3409	1.3463	1.3710
1.3711	1.3763	1.3846	1.4076	1.4202	1.4260	1.4331	1.4469
1.4554	1.4628	1.4649	1.4701	1.4787	1.4918	1.4970	1.5058
1.5165	1.5296	1.5354	1.5417	1.5487	1.5529	1.5540	1.5631
1.5662	1.5759	1.5795	1.5854	1.5978	1.6012	1.6089	1.6119

1.6152	1.6259	1.6377	1.6443	1.6472	1.6528	1.6545	1.6638
1.6737	1.6805	1.6902	1.6967	1.7039	1.7059	1.7141	1.7165
1.7280	1.7318	1.7349	1.7397	1.7464	1.7520	1.7601	1.7702
1.7768	1.7895	1.7909	1.7980	1.8216	1.8248	1.8348	1.8441
1.8487	1.8628	1.8933	1.8991	1.9180	1.9276	1.9379	1.9559
1.9649	1.9834	1.9950	2.0006	2.0177	2.0332	2.0421	2.0473
2.0534	2.0665	2.0831	2.0945	2.1032	2.1146	2.1231	2.1362
2.1416	2.1463	2.1574	2.1709	2.1804	2.1875	2.1959	2.2013
2.2186	2.2308	2.2571	2.2595	2.2718	2.2953	2.3087	2.3318
2.3406	2.3564	2.3712	2.3788	2.3844	2.4061	2.4186	2.4295
2.4350	2.4536	2.4572	2.4679	2.4740	2.4829	2.4909	2.4936
2.4972	2.5088	2.5095	2.5183	2.5224	2.5270	2.5335	2.5447
2.5542	2.5620	2.5726	2.5746	2.5823	2.5873	2.5985	2.6080
2.6101	2.6184	2.6225	2.6361	2.6436	2.6569	2.6602	2.6748
2.6838	2.6919	2.7007	2.7145	2.7214	2.7324	2.7365	2.7402
2.7494	2.7610	2.7771	2.7814	2.8011	2.8125	2.8182	2.8356
2.8473	2.8565	2.8637	2.8811	2.8837	2.9045	2.9144	2.9302
2.9339	2.9401	2.9523	2.9654	2.9697	3.0030	3.0118	3.0344
3.0365	3.0721	3.0780	3.0910	3.1375	3.1600	3.1914	3.2329
3.2641	3.2993	3.3512	3.3607	3.4585	3.5061	3.5272	3.5449
3.5934	3.6290	3.6398	3.6721	3.6826	3.7005	3.7496	3.7633
3.7894	3.8001	3.8208	3.8233	3.8284	3.8489	3.8530	3.8642
3.8692	3.8724	3.8791	3.8844	3.8977	3.9992	4.0573	4.0848
4.1469	4.2014	4.3143	4.3479	4.3811	4.5533	4.6417	4.6806
4.8800	4.9213	5.2165	23.7239	23.8468	23.8802	23.8824	23.8954
23.8994	23.9206	23.9373	23.9623	23.9744	24.0155	24.0313	24.0580
24.0670	24.1661	35.4375	35.5936				

Ground-State Mulliken Net Atomic Charges

Atom	Charge (a.u.)
1 N	-0.002613
2 N	0.035850
3 C	-0.084630
4 H	0.215417
5 C	-0.132055
6 H	0.216323
7 C	-0.104087
8 H	0.212856
9 C	-0.264280
10 H	0.226073
11 H	0.193533
12 H	0.228375
13 C	-0.300569
14 H	0.186034
15 H	0.228588
16 C	-0.330636
17 H	0.192080
18 H	0.169528
19 C	0.010621
20 H	0.182335
21 C	-0.716038
22 H	0.157718
23 H	0.187603
24 H	0.185612
25 C	-0.274166
26 H	0.134049
27 H	0.178461

28 C	-0.334328
29 H	0.175302
30 H	0.135586
31 C	-0.068574
32 H	0.145806
33 C	-0.612960
34 H	0.170600
35 H	0.165452
36 C	0.378790
37 C	-0.495711
38 H	0.139211
39 H	0.166505
40 H	0.167288
41 C	-0.535684
42 H	0.128599
43 H	0.171112
44 H	0.171021

Sum of atomic charges = 1.000000

Cartesian Multipole Moments

Charge (ESU x 10^10)

4.8032

Dipole Moment (Debye)

X 8.3590 Y 2.4548 Z 16.7461

Tot 18.8767

Quadrupole Moments (Debye-Ang)

XX -75.0936 XY 3.3411 YY -101.8840

XZ 38.0359 YZ 13.4261 ZZ -3.4803

Traceless Quadrupole Moments (Debye-Ang)

QXX -44.8231 QYY -125.1940 QZZ 170.0171

QXY 10.0233 QXZ 114.1076 QYZ 40.2782

Octopole Moments (Debye-Ang^2)

XXX 50.1216 XXY 5.4018 XYY -17.7042

YYY -22.7219 XXZ 76.0780 XYZ 19.6911

YYZ -19.4133 XZZ 156.6317 YZZ 61.2712

ZZZ 471.0278

Traceless Octopole Moments (Debye-Ang^2)

XXX -949.6182 YYY -736.3884 ZZZ 2316.1838

XXY -50.8265 XXZ -441.9072 XYY -832.7106

XYZ 295.3660 XZZ 1782.3288 YYZ -1874.2766

YZZ 787.2149

Hexadecapole Moments (Debye-Ang^3)

XXXX -2915.6080 XXXY 62.0826 XXYY -681.4927

XYYY 42.8744 YYYY -737.4668 XXXZ -1593.6844

XXYZ -53.9198 XYYZ -678.4352 YYYZ -281.6183

XXZZ -1228.9032 XYZZ 119.2963 YYZZ -1343.2320

XZZZ -879.1596 YZZZ 112.9954 ZZZZ -4044.1077

Traceless Hexadecapole Moments (Debye-Ang^3)

XXXX 361.5691 XXXY -3572.7308 XXXZ -25529.2956

XXYY -347.1184 XXYZ -2308.5110 XXZZ -14.4506

XYYY -5589.5861 XYYZ -23966.5090 XYZZ 9162.3169

XZZZ 49495.8047 YYYY 43323.2793 YYYZ -19510.7060

YYZZ -42976.1609 YZZZ 21819.2170 ZZZZ 42990.6115

- Entering drvman on Mon Jun 2 13:50:54 2025 -

Calculating analytic gradient of the SCF energy

Gradient of SCF Energy

	1	2	3	4	5	6
1	-0.0000343	0.0000195	-0.0000129	-0.0000457	-0.0000227	0.0000253
2	0.0000089	0.0000949	-0.0000894	0.0000028	0.0000886	-0.0000270
3	0.0000069	-0.0000713	0.0000522	0.0000015	-0.0000077	-0.0000544

	7	8	9	10	11	12
1	0.0000363	0.0000009	0.0000484	-0.0000302	-0.0000352	0.0000153
2	0.0000103	-0.0000235	-0.0000278	0.0000034	0.0000017	0.0000222
3	0.0000105	0.0000518	-0.0000278	0.0000508	-0.0000029	-0.0000073

	13	14	15	16	17	18
1	-0.0000352	0.0000332	0.0000295	-0.0000552	-0.0000074	0.0000425
2	-0.0000463	0.0000589	-0.0000129	-0.0000226	-0.0000158	0.0000168
3	0.0000345	0.0000668	-0.0000191	-0.0000043	-0.0000018	0.0000233

	19	20	21	22	23	24
1	0.0000175	0.0000220	-0.0000197	0.0000244	-0.0000220	0.0000469
2	0.0000470	-0.0000087	0.0000200	-0.0000075	-0.0000203	-0.0000144
3	-0.0000077	-0.0000094	0.0000095	0.0000091	-0.0000010	-0.0000109

	25	26	27	28	29	30
1	0.0000120	-0.0000174	-0.0000129	0.0000091	0.0000028	-0.0000665
2	-0.0000055	0.0000298	-0.0000140	0.0000002	0.0000023	-0.0000152
3	-0.0000043	-0.0000672	0.0000158	0.0000073	-0.0000250	-0.0000523

	31	32	33	34	35	36
1	-0.0000076	0.0000092	0.0000430	-0.0000414	-0.0000065	-0.0000075
2	-0.0000121	0.0000247	-0.0000084	0.0000060	-0.0000418	0.0000072
3	-0.0000024	0.0000297	-0.0000474	0.0000329	0.0000210	0.0000033

	37	38	39	40	41	42
1	0.0000036	-0.0000084	0.0000168	-0.0000276	0.0000193	0.0000312

```
   2  -0.0000248   0.0000203   0.0000044  -0.0000130   0.0000225  -0.0000285
   3  -0.0000121   0.0000191  -0.0000164  -0.0000097   0.0000211   0.0000243
          43          44
   1  -0.0000184   0.0000259
   2  -0.0000174   0.0000042
   3  -0.0000148  -0.0000140
 Max gradient component =       9.485E-05
 RMS gradient           =       3.015E-05
 Gradient time:  CPU 193.20 s  wall 34.54 s

------------------------------------------------
-  Entering optman on Mon Jun  2 13:51:28 2025  -
------------------------------------------------

 Geometry Optimization Parameters
   NAtoms,    NIC,     NZ,  NCons,   NDum,   NFix, NCnnct, MaxDiis
       44     348       0       0       0       0       0       0

 Cartesian Hessian Update
 Hessian updated using BFGS update


** GEOMETRY OPTIMIZATION IN DELOCALIZED INTERNAL COORDINATES **
   Searching for a Minimum

   Optimization Cycle:   2

                       Coordinates (Angstroms)
     ATOM              X               Y               Z
      1  N        2.0187164159    0.6841379198    5.0839680195
```

2	N	2.0613311889	0.6693221279	2.9187364149
3	C	1.2755061447	0.7878256055	3.9839824465
4	H	0.2091861763	0.9452098637	3.9599440940
5	C	3.3530832491	0.4762281066	3.3505448157
6	H	4.1719521231	0.3517422424	2.6620583840
7	C	3.3282885166	0.4885887710	4.7068837832
8	H	4.1206669941	0.3805562292	5.4284030137
9	C	1.5328099161	0.7760200944	6.4608706111
10	H	1.7642247510	-0.1479718774	6.9891632043
11	H	0.4546360854	0.9234666760	6.4462752230
12	H	2.0073294408	1.6217843380	6.9569559091
13	C	1.6123815992	0.6667428644	1.5153648429
14	H	0.6371960125	1.1530136526	1.4983123710
15	H	2.3065506956	1.2959901786	0.9543367882
16	C	1.5577327763	-0.7478488845	0.9486928306
17	H	0.8213606195	-1.3333061438	1.5129294256
18	H	2.5307574407	-1.2270668668	1.1042445682
19	C	1.2101038663	-0.7931881200	-0.5470849696
20	H	1.9727654032	-0.2143312256	-1.0867293962
21	C	1.2871190732	-2.2427284788	-1.0307793196
22	H	2.2417079037	-2.7009673023	-0.7582619225
23	H	0.4848779033	-2.8425892383	-0.5879443476
24	H	1.1923336460	-2.3069008476	-2.1153439951
25	C	-0.1590042263	-0.1582413073	-0.8276490981
26	H	-0.1509284927	0.8947285679	-0.5185843601
27	H	-0.9212587079	-0.6569222899	-0.2127924452
28	C	-0.6007767330	-0.2018078554	-2.2914312651
29	H	0.1939548987	0.2098429000	-2.9265801834
30	H	-0.7399867161	-1.2398062535	-2.6017390411
31	C	-1.8800482174	0.5712232353	-2.5068361559

32	H	-1.7962418683	1.6247894157	-2.2460597176
33	C	-3.2239420045	-0.0594029711	-2.2075967511
34	H	-3.9824981658	0.5348683605	-1.7101173494
35	H	-3.2272942842	-1.1108543082	-1.9352276621
36	C	-2.8511645557	0.2869362075	-3.6273552584
37	C	-3.5337497643	1.4830782924	-4.2572771366
38	H	-3.7110485657	2.2752986943	-3.5247945169
39	H	-2.9215586908	1.8996501216	-5.0634284551
40	H	-4.5007317830	1.1976316377	-4.6826465548
41	C	-2.5818510218	-0.8367880234	-4.6064627229
42	H	-2.1797961906	-1.7299730264	-4.1240962334
43	H	-3.5106589062	-1.1280728558	-5.1056736435
44	H	-1.8740339464	-0.5199082269	-5.3791742442

Point Group: c1 Number of degrees of freedom: 126

Energy is -697.098626316

Hessian updated using BFGS update

internal optimization (0)

126 Hessian modes will be used to form the next step

Hessian Eigenvalues:

0.000194	0.000722	0.001518	0.001629	0.001888	0.002160
0.002486	0.003007	0.003345	0.003926	0.008192	0.009001
0.012644	0.013740	0.014907	0.016092	0.017384	0.021397
0.021618	0.023648	0.025754	0.033366	0.034754	0.037557
0.039352	0.040083	0.040297	0.040936	0.044567	0.045753
0.046075	0.046809	0.046993	0.047627	0.047752	0.048623
0.048756	0.049211	0.049540	0.050766	0.050996	0.053190

0.061968	0.063025	0.065829	0.069036	0.072949	0.074746
0.074955	0.078266	0.082667	0.083484	0.105716	0.106503
0.107709	0.110910	0.119386	0.126214	0.127301	0.130040
0.131761	0.134459	0.137752	0.140555	0.147205	0.149408
0.151221	0.151723	0.154290	0.155383	0.166607	0.173663
0.178714	0.183979	0.184504	0.194412	0.196313	0.202187
0.214419	0.231226	0.242071	0.246107	0.248653	0.267039
0.284430	0.287850	0.300836	0.304935	0.307485	0.313098
0.324445	0.325423	0.327229	0.329974	0.333352	0.334733
0.336531	0.338395	0.340237	0.340995	0.341191	0.341499
0.341938	0.345502	0.345840	0.347829	0.348737	0.349206
0.350802	0.352173	0.353224	0.356118	0.356391	0.356768
0.358355	0.359739	0.362500	0.364147	0.387040	0.387508
0.389326	0.394503	0.430922	0.466127	0.502958	0.598515

WARNING Magnitude of eigenvalue 1 too small. Replaced by 0.001000

WARNING Magnitude of eigenvalue 2 too small. Replaced by 0.001000

Minimum search - taking simple RFO step

Searching for Lamda that Minimizes Along All modes

Value Taken Lamda = -0.00000659

dLimit reduced 2.100000000000000E-003 0.300000000000000

6.197912880627689E-003 2.065451614531968E-004 7.000000000000000E-004

0

Calculated Step part too Large. Step scaled by 0.101647

Step Taken. Stepsize is 0.006524 0.006198

	Maximum	Tolerance	Cnvgd?
Gradient	0.000207	0.000700	YES
Displacement	0.002283	0.001400	NO
Energy change	-0.000208	0.000020	NO

New Cartesian Coordinates Obtained by Inverse Iteration

Displacement from previous Coordinates is: 0.024906

Standard Nuclear Orientation (Angstroms)

I	Atom	X	Y	Z
1	N	2.0177628187	0.6847515507	5.0838670670
2	N	2.0614140513	0.6685434976	2.9186662226
3	C	1.2753008114	0.7892111363	3.9834467941
4	H	0.2092983363	0.9486003718	3.9587885474
5	C	3.3525895142	0.4732710125	3.3512283918
6	H	4.1715825949	0.3469077613	2.6632451501
7	C	3.3271438895	0.4865004719	4.7075411326
8	H	4.1189785039	0.3774270730	5.4294938071
9	C	1.5313073511	0.7781686593	6.4604729818
10	H	1.7609113747	-0.1459561335	6.9893200335
11	H	0.4533932493	0.9274099108	6.4452522988
12	H	2.0069891056	1.6233720702	6.9563971265
13	C	1.6131935219	0.6663163334	1.5150610031
14	H	0.6379195787	1.1523964243	1.4976948806
15	H	2.3075062450	1.2958378262	0.9545197553
16	C	1.5588922741	-0.7481015971	0.9479570640
17	H	0.8228141209	-1.3339402807	1.5121748749
18	H	2.5320706280	-1.2271278741	1.1031024731
19	C	1.2109156976	-0.7930184320	-0.5477537627
20	H	1.9730757649	-0.2134626347	-1.0873534148
21	C	1.2886648327	-2.2422908482	-1.0321067554

22	H	2.2437808775	-2.6999364319	-0.7604589758
23	H	0.4871798614	-2.8429198753	-0.5889511503
24	H	1.1931345474	-2.3060690992	-2.1166279724
25	C	-0.1586316818	-0.1587577153	-0.8277120871
26	H	-0.1511816674	0.8940111695	-0.5179620860
27	H	-0.9204997791	-0.6583056738	-0.2130902064
28	C	-0.6005108926	-0.2016723058	-2.2914837874
29	H	0.1940537484	0.2105628771	-2.9264581935
30	H	-0.7393799697	-1.2395560610	-2.6023100446
31	C	-1.8799993535	0.5711248563	-2.5064081550
32	H	-1.7962758232	1.6246713590	-2.2455409123
33	C	-3.2237177383	-0.0596957453	-2.2067959057
34	H	-3.9821550435	0.5344187351	-1.7089620291
35	H	-3.2268619860	-1.1111905818	-1.9346128871
36	C	-2.8514792221	0.2868943039	-3.6266320502
37	C	-3.5343935728	1.4830819915	-4.2561131384
38	H	-3.7115111886	2.2751592826	-3.5234375484
39	H	-2.9225294758	1.8998455741	-5.0624089309
40	H	-4.5014949258	1.1976171559	-4.6811906503
41	C	-2.5825021622	-0.8366616207	-4.6060229077
42	H	-2.1801228045	-1.7298718838	-4.1239816923
43	H	-3.5115225067	-1.1279693763	-5.1048171265
44	H	-1.8751035063	-0.5195972341	-5.3790372340

Nuclear Repulsion Energy = 1186.84124348 hartrees

There are 65 alpha and 65 beta electrons

- Entering fldman on Mon Jun 2 13:51:28 2025 -

```
Applying Cartesian multipole field
   Component          Value
   ---------          -----
    (2,0,0)       1.00000E-11
    (0,2,0)       2.00000E-11
    (0,0,2)      -3.00000E-12
Nucleus-field energy     =     0.0000000163 hartrees

------------------------------------------------
-  Entering gesman on Mon Jun  2 13:51:28 2025  -
------------------------------------------------

Requested basis set is 6-311+G(d,p)
There are 210 shells and 536 basis functions
A cutoff of  1.0D-12 yielded  14758 shell pairs
There are     99435 function pairs (     105192 Cartesian)
Smallest overlap matrix eigenvalue = 3.12E-06
Linear dependence detected in AO basis
Tighter screening thresholds may be required for diffuse basis sets
Use S2THRESH > 12 and THRESH = 14 in case of SCF convergence issues
Number of orthogonalized atomic orbitals = 533
Maximum deviation from orthogonality = 1.945E-11
Guess MOs from SCF MO coefficient file
Reading MOs from coefficient file
Reading MOs from coefficient file

------------------------------------------------
-  Entering scfman on Mon Jun  2 13:51:29 2025  -
------------------------------------------------
```

Long-range K will be added via erf

Coulomb attenuation parameter = 0.2 bohr**(-1)

A restricted hybrid HF-DFT SCF calculation will be

performed using Pulay DIIS + Geometric Direct Minimization

Exchange: 0.2220 Hartree-Fock + 1.0000 wB97X-D + LR-HF

Correlation: 1.0000 wB97X-D

Using Euler-Maclaurin-Lebedev (75,302) quadrature formula

Dispersion: Grimme D

SCF converges when RMS gradient is below 1.0E-07

Geometry optimization detected. Setting ReadMinima to 0

Setting SaveMinima to 0

using 6 threads for integral computing

OpenMP Integral computing Module

Release: version 1.0, May 2013, Q-Chem Inc. Pittsburgh

----

using 6 threads for integral computing

OpenMP Integral computing Module

Release: version 1.0, May 2013, Q-Chem Inc. Pittsburgh

Cycle	Energy	DIIS Error
1	-697.0986746419	2.30E-05
2	-697.0986263364	2.41E-06
3	-697.0986268760	9.21E-07
4	-697.0986269205	5.78E-07
5	-697.0986269343	1.39E-07

6 -697.0986269351 6.09E-08 Convergence criterion met

SCF time: CPU 288.50 s wall 49.24 s

SCF energy in the final basis set = -697.09862694

Total energy in the final basis set = -697.09862694

- Entering anlman on Mon Jun 2 13:52:18 2025 -

Orbital Energies (a.u.)

Alpha MOs

-- Occupied --

-14.6914 -14.6862 -10.5476 -10.4971 -10.4962 -10.4873 -10.4807 -10.4136

-10.3967 -10.3739 -10.3646 -10.3609 -10.3603 -10.3554 -10.3432 -10.3330

-10.3305 -1.3452 -1.2228 -1.0700 -1.0465 -1.0402 -1.0139 -0.9724

-0.9342 -0.9041 -0.8833 -0.8802 -0.8640 -0.8500 -0.8307 -0.8051

-0.7692 -0.7512 -0.7398 -0.7287 -0.7233 -0.7099 -0.7013 -0.6937

-0.6878 -0.6761 -0.6535 -0.6519 -0.6369 -0.6323 -0.6105 -0.6020

-0.5903 -0.5800 -0.5708 -0.5604 -0.5520 -0.5417 -0.5338 -0.5305

-0.5236 -0.5174 -0.5098 -0.5052 -0.5000 -0.4943 -0.4782 -0.4237

-0.4098

-- Virtual --

-0.1098 -0.0765 -0.0635 -0.0445 -0.0436 -0.0300 -0.0271 -0.0229

-0.0171 -0.0069 -0.0064 -0.0040 0.0044 0.0097 0.0117 0.0171

0.0238	0.0249	0.0273	0.0368	0.0384	0.0388	0.0451	0.0494
0.0506	0.0543	0.0555	0.0608	0.0657	0.0719	0.0752	0.0764
0.0780	0.0830	0.0856	0.0894	0.0920	0.0930	0.0978	0.1008
0.1048	0.1088	0.1122	0.1166	0.1182	0.1201	0.1252	0.1286
0.1319	0.1329	0.1371	0.1402	0.1415	0.1443	0.1475	0.1495
0.1556	0.1568	0.1579	0.1630	0.1652	0.1697	0.1714	0.1719
0.1768	0.1806	0.1821	0.1869	0.1895	0.1929	0.1950	0.1969
0.2046	0.2080	0.2099	0.2146	0.2197	0.2212	0.2244	0.2269
0.2307	0.2323	0.2378	0.2415	0.2467	0.2489	0.2517	0.2570
0.2612	0.2650	0.2669	0.2699	0.2727	0.2806	0.2865	0.2888
0.2937	0.2980	0.3043	0.3070	0.3153	0.3177	0.3295	0.3393
0.3482	0.3496	0.3532	0.3612	0.3681	0.3734	0.3774	0.3824
0.3882	0.4022	0.4150	0.4222	0.4284	0.4299	0.4379	0.4554
0.4659	0.4688	0.4778	0.4792	0.4813	0.4951	0.4969	0.5124
0.5184	0.5254	0.5309	0.5338	0.5422	0.5468	0.5499	0.5555
0.5667	0.5784	0.5853	0.5885	0.5967	0.5999	0.6059	0.6140
0.6177	0.6197	0.6233	0.6313	0.6351	0.6419	0.6545	0.6654
0.6679	0.6718	0.6764	0.6785	0.6797	0.6829	0.6869	0.6902
0.6941	0.6998	0.7040	0.7083	0.7111	0.7172	0.7229	0.7251
0.7301	0.7381	0.7399	0.7432	0.7460	0.7542	0.7576	0.7632
0.7717	0.7779	0.7879	0.7926	0.7977	0.8029	0.8115	0.8134
0.8392	0.8537	0.8646	0.8690	0.8832	0.9053	0.9083	0.9193
0.9326	0.9395	0.9467	0.9702	0.9806	0.9820	0.9920	1.0009
1.0073	1.0312	1.0539	1.0573	1.0695	1.0839	1.0867	1.1045
1.1119	1.1244	1.1420	1.1575	1.1841	1.1987	1.2137	1.2384
1.2556	1.2627	1.2830	1.3116	1.3319	1.3409	1.3463	1.3710
1.3711	1.3763	1.3846	1.4076	1.4202	1.4260	1.4331	1.4469
1.4553	1.4629	1.4649	1.4701	1.4787	1.4918	1.4970	1.5058
1.5165	1.5297	1.5354	1.5417	1.5487	1.5529	1.5540	1.5631
1.5662	1.5758	1.5795	1.5853	1.5977	1.6012	1.6090	1.6118

1.6152	1.6259	1.6377	1.6444	1.6472	1.6528	1.6545	1.6638
1.6736	1.6805	1.6902	1.6967	1.7038	1.7059	1.7141	1.7164
1.7280	1.7318	1.7349	1.7398	1.7464	1.7520	1.7601	1.7701
1.7768	1.7895	1.7909	1.7980	1.8216	1.8248	1.8348	1.8441
1.8487	1.8628	1.8933	1.8991	1.9180	1.9276	1.9379	1.9559
1.9649	1.9834	1.9950	2.0005	2.0177	2.0332	2.0419	2.0473
2.0534	2.0665	2.0831	2.0946	2.1032	2.1145	2.1231	2.1362
2.1416	2.1463	2.1574	2.1710	2.1804	2.1875	2.1958	2.2013
2.2187	2.2308	2.2571	2.2595	2.2718	2.2953	2.3087	2.3318
2.3407	2.3564	2.3712	2.3788	2.3844	2.4061	2.4187	2.4295
2.4350	2.4536	2.4572	2.4679	2.4740	2.4829	2.4909	2.4936
2.4972	2.5088	2.5096	2.5183	2.5224	2.5270	2.5335	2.5447
2.5542	2.5621	2.5726	2.5746	2.5823	2.5873	2.5984	2.6080
2.6101	2.6184	2.6225	2.6360	2.6435	2.6569	2.6601	2.6748
2.6838	2.6920	2.7007	2.7145	2.7214	2.7323	2.7365	2.7402
2.7495	2.7610	2.7770	2.7813	2.8011	2.8125	2.8182	2.8356
2.8474	2.8565	2.8637	2.8811	2.8837	2.9045	2.9144	2.9302
2.9339	2.9401	2.9523	2.9654	2.9697	3.0031	3.0118	3.0344
3.0365	3.0721	3.0780	3.0910	3.1374	3.1600	3.1915	3.2329
3.2641	3.2993	3.3513	3.3607	3.4585	3.5061	3.5272	3.5450
3.5935	3.6291	3.6398	3.6721	3.6826	3.7006	3.7494	3.7633
3.7894	3.8001	3.8208	3.8234	3.8285	3.8489	3.8530	3.8642
3.8692	3.8724	3.8791	3.8844	3.8977	3.9991	4.0573	4.0848
4.1469	4.2014	4.3142	4.3479	4.3811	4.5533	4.6418	4.6806
4.8800	4.9213	5.2165	23.7239	23.8468	23.8802	23.8824	23.8954
23.8994	23.9206	23.9374	23.9623	23.9744	24.0155	24.0313	24.0580
24.0670	24.1661	35.4375	35.5936				

Ground-State Mulliken Net Atomic Charges

Atom	Charge (a.u.)
1 N	-0.002659
2 N	0.036027
3 C	-0.084601
4 H	0.215402
5 C	-0.131987
6 H	0.216322
7 C	-0.104249
8 H	0.212857
9 C	-0.264312
10 H	0.226065
11 H	0.193530
12 H	0.228386
13 C	-0.300421
14 H	0.185900
15 H	0.228709
16 C	-0.330682
17 H	0.192105
18 H	0.169469
19 C	0.010828
20 H	0.182331
21 C	-0.716112
22 H	0.157730
23 H	0.187582
24 H	0.185592
25 C	-0.274069
26 H	0.133989
27 H	0.178463

28 C	-0.334328
29 H	0.175306
30 H	0.135602
31 C	-0.068870
32 H	0.145827
33 C	-0.612749
34 H	0.170598
35 H	0.165458
36 C	0.378676
37 C	-0.495761
38 H	0.139208
39 H	0.166504
40 H	0.167287
41 C	-0.535688
42 H	0.128599
43 H	0.171113
44 H	0.171022

Sum of atomic charges = 1.000000

Cartesian Multipole Moments

Charge (ESU x 10^10)

4.8032

Dipole Moment (Debye)

X 8.3568 Y 2.4557 Z 16.7451

Tot 18.8750

Quadrupole Moments (Debye-Ang)

XX -75.1068 XY 3.3200 YY -101.8760

```
    XZ      38.0213     YZ      13.4408     ZZ      -3.4865
   Traceless Quadrupole Moments (Debye-Ang)
    QXX     -44.8511    QYY    -125.1587    QZZ     170.0097
    QXY       9.9601    QXZ     114.0639    QYZ      40.3225
   Octopole Moments (Debye-Ang^2)
    XXX      50.0447    XXY       5.3070    XYY     -17.7089
    YYY     -22.6657    XXZ      75.9973    XYZ      19.6271
    YYZ     -19.3713    XZZ     156.5443    YZZ      61.3800
    ZZZ     470.9713
   Traceless Octopole Moments (Debye-Ang^2)
    XXX    -949.2506    YYY    -736.1775    ZZZ    2316.1942
    XXY     -52.4591    XXZ    -442.8330    XYY    -832.2744
    XYZ     294.4070    XZZ    1781.5249    YYZ   -1873.3612
    YZZ     788.6366
   Hexadecapole Moments (Debye-Ang^3)
    XXXX  -2916.1198   XXXY      61.9534   XXYY    -681.5505
    XYYY      43.3034   YYYY    -737.3737   XXXZ   -1593.4902
    XXYZ     -54.3191   XYYZ    -678.2836   YYYZ    -281.6627
    XXZZ  -1229.1669   XYZZ     119.2083   YYZZ   -1342.8059
    XZZZ    -879.2286   YZZZ     113.4733   ZZZZ   -4043.5853
   Traceless Hexadecapole Moments (Debye-Ang^3)
    XXXX     385.6391   XXXY   -3595.8252   XXXZ  -25521.3656
    XXYY    -346.6710   XXYZ   -2358.7757   XXZZ     -38.9682
    XYYY   -5554.0745   XYYZ  -23954.7377   XYZZ    9149.8997
    XZZZ   49476.1033   YYYY   43294.3436   YYYZ  -19540.4037
    YYZZ  -42947.6727   YZZZ   21899.1793   ZZZZ   42986.6408
 -----------------------------------------------------------------


 -------------------------------------------------
 -  Entering drvman on Mon Jun  2 13:52:18 2025  -
```

Calculating analytic gradient of the SCF energy

Gradient of SCF Energy

	1	2	3	4	5	6
1	-0.0000321	0.0000151	-0.0000125	-0.0000421	-0.0000184	0.0000230
2	0.0000081	0.0000898	-0.0000818	0.0000029	0.0000807	-0.0000252
3	0.0000052	-0.0000664	0.0000503	0.0000010	-0.0000059	-0.0000508

	7	8	9	10	11	12
1	0.0000339	-0.0000004	0.0000451	-0.0000286	-0.0000327	0.0000137
2	0.0000096	-0.0000216	-0.0000250	0.0000039	0.0000014	0.0000198
3	0.0000104	0.0000474	-0.0000266	0.0000478	-0.0000026	-0.0000069

	13	14	15	16	17	18
1	-0.0000323	0.0000306	0.0000266	-0.0000504	-0.0000065	0.0000396
2	-0.0000433	0.0000541	-0.0000111	-0.0000203	-0.0000143	0.0000155
3	0.0000319	0.0000623	-0.0000175	-0.0000046	-0.0000012	0.0000217

	19	20	21	22	23	24
1	0.0000165	0.0000203	-0.0000172	0.0000230	-0.0000201	0.0000424
2	0.0000441	-0.0000076	0.0000181	-0.0000064	-0.0000187	-0.0000122
3	-0.0000076	-0.0000086	0.0000086	0.0000085	-0.0000005	-0.0000105

	25	26	27	28	29	30
1	0.0000111	-0.0000162	-0.0000120	0.0000086	0.0000026	-0.0000589
2	-0.0000058	0.0000276	-0.0000135	0.0000002	0.0000025	-0.0000137
3	-0.0000037	-0.0000622	0.0000144	0.0000064	-0.0000231	-0.0000463

	31	32	33	34	35	36
1	-0.0000070	0.0000084	0.0000391	-0.0000379	-0.0000056	-0.0000066
2	-0.0000113	0.0000226	-0.0000081	0.0000050	-0.0000388	0.0000058
3	-0.0000021	0.0000270	-0.0000432	0.0000300	0.0000191	0.0000033

	37	38	39	40	41	42
1	0.0000032	-0.0000080	0.0000154	-0.0000254	0.0000172	0.0000281

```
  2  -0.0000229   0.0000180   0.0000037  -0.0000125   0.0000200  -0.0000266
  3  -0.0000115   0.0000176  -0.0000152  -0.0000091   0.0000188   0.0000213
        43          44
  1  -0.0000166   0.0000240
  2  -0.0000163   0.0000037
  3  -0.0000138  -0.0000132
 Max gradient component =       8.981E-05
 RMS gradient           =       2.776E-05
 Gradient time:  CPU 193.11 s  wall 34.54 s

------------------------------------------------
-  Entering optman on Mon Jun  2 13:52:53 2025  -
------------------------------------------------

 Geometry Optimization Parameters
   NAtoms,    NIC,     NZ,  NCons,   NDum,   NFix, NCnnct, MaxDiis
      44     348      0       0       0       0       0       0

 Cartesian Hessian Update
 Hessian updated using BFGS update


** GEOMETRY OPTIMIZATION IN DELOCALIZED INTERNAL COORDINATES **
   Searching for a Minimum

   Optimization Cycle:   3

                      Coordinates (Angstroms)
     ATOM              X              Y              Z
      1  N        2.0177628187    0.6847515507    5.0838670670
```

2	N	2.0614140513	0.6685434976	2.9186662226
3	C	1.2753008114	0.7892111363	3.9834467941
4	H	0.2092983363	0.9486003718	3.9587885474
5	C	3.3525895142	0.4732710125	3.3512283918
6	H	4.1715825949	0.3469077613	2.6632451501
7	C	3.3271438895	0.4865004719	4.7075411326
8	H	4.1189785039	0.3774270730	5.4294938071
9	C	1.5313073511	0.7781686593	6.4604729818
10	H	1.7609113747	-0.1459561335	6.9893200335
11	H	0.4533932493	0.9274099108	6.4452522988
12	H	2.0069891056	1.6233720702	6.9563971265
13	C	1.6131935219	0.6663163334	1.5150610031
14	H	0.6379195787	1.1523964243	1.4976948806
15	H	2.3075062450	1.2958378262	0.9545197553
16	C	1.5588922741	-0.7481015971	0.9479570640
17	H	0.8228141209	-1.3339402807	1.5121748749
18	H	2.5320706280	-1.2271278741	1.1031024731
19	C	1.2109156976	-0.7930184320	-0.5477537627
20	H	1.9730757649	-0.2134626347	-1.0873534148
21	C	1.2886648327	-2.2422908482	-1.0321067554
22	H	2.2437808775	-2.6999364319	-0.7604589758
23	H	0.4871798614	-2.8429198753	-0.5889511503
24	H	1.1931345474	-2.3060690992	-2.1166279724
25	C	-0.1586316818	-0.1587577153	-0.8277120871
26	H	-0.1511816674	0.8940111695	-0.5179620860
27	H	-0.9204997791	-0.6583056738	-0.2130902064
28	C	-0.6005108926	-0.2016723058	-2.2914837874
29	H	0.1940537484	0.2105628771	-2.9264581935
30	H	-0.7393799697	-1.2395560610	-2.6023100446
31	C	-1.8799993535	0.5711248563	-2.5064081550

32	H	-1.7962758232	1.6246713590	-2.2455409123
33	C	-3.2237177383	-0.0596957453	-2.2067959057
34	H	-3.9821550435	0.5344187351	-1.7089620291
35	H	-3.2268619860	-1.1111905818	-1.9346128871
36	C	-2.8514792221	0.2868943039	-3.6266320502
37	C	-3.5343935728	1.4830819915	-4.2561131384
38	H	-3.7115111886	2.2751592826	-3.5234375484
39	H	-2.9225294758	1.8998455741	-5.0624089309
40	H	-4.5014949258	1.1976171559	-4.6811906503
41	C	-2.5825021622	-0.8366616207	-4.6060229077
42	H	-2.1801228045	-1.7298718838	-4.1239816923
43	H	-3.5115225067	-1.1279693763	-5.1048171265
44	H	-1.8751035063	-0.5195972341	-5.3790372340

Point Group: c1   Number of degrees of freedom: 126

Energy is   -697.098626935

Hessian updated using BFGS update

internal optimization (0)

126 Hessian modes will be used to form the next step

Hessian Eigenvalues:

0.000200	0.001276	0.001521	0.001681	0.001859	0.002120
0.002486	0.002908	0.003341	0.003774	0.007880	0.008990
0.012617	0.013649	0.014895	0.016068	0.017382	0.021381
0.021493	0.023644	0.025739	0.033156	0.034361	0.037264
0.039279	0.040076	0.040298	0.040903	0.044367	0.045234
0.045853	0.046806	0.046958	0.047518	0.047646	0.048606
0.048633	0.049131	0.049517	0.050780	0.050990	0.053178

0.061963	0.063012	0.065782	0.069020	0.072950	0.074222
0.074967	0.078264	0.082449	0.083339	0.104940	0.106523
0.107672	0.110580	0.119397	0.126218	0.127300	0.130040
0.131803	0.134397	0.137634	0.140557	0.147184	0.149440
0.151218	0.151629	0.154310	0.155393	0.166078	0.173641
0.178158	0.182882	0.184009	0.194368	0.196177	0.200759
0.211461	0.231183	0.241646	0.245944	0.248623	0.267075
0.284366	0.287833	0.299758	0.304029	0.306991	0.312110
0.324233	0.325442	0.327206	0.329881	0.332330	0.334717
0.335057	0.338378	0.340196	0.340825	0.341165	0.341282
0.341933	0.345368	0.345864	0.347771	0.348739	0.349199
0.350792	0.351966	0.353089	0.356119	0.356391	0.356784
0.357969	0.359736	0.362464	0.364147	0.386850	0.387488
0.389307	0.394527	0.430917	0.466120	0.502869	0.597951

Minimum search - taking simple RFO step

Searching for Lamda that Minimizes Along All modes

Value Taken Lamda = -0.00000559

dLimit reduced 2.100000000000000E-003 0.300000000000000

6.510406398883708E-003 1.953573787882356E-004 7.000000000000000E-004

0

Calculated Step part too Large. Step scaled by 0.096768

Step Taken. Stepsize is 0.005119 0.006510

	Maximum	Tolerance	Cnvgd?
Gradient	0.000195	0.000700	YES
Displacement	0.002175	0.001400	NO
Energy change	-0.000001	0.000020	YES

Final energy is -697.098626935093

****

** OPTIMIZATION CONVERGED **

****

Coordinates (Angstroms)

ATOM		X	Y	Z
1	N	2.0177628187	0.6847515507	5.0838670670
2	N	2.0614140513	0.6685434976	2.9186662226
3	C	1.2753008114	0.7892111363	3.9834467941
4	H	0.2092983363	0.9486003718	3.9587885474
5	C	3.3525895142	0.4732710125	3.3512283918
6	H	4.1715825949	0.3469077613	2.6632451501
7	C	3.3271438895	0.4865004719	4.7075411326
8	H	4.1189785039	0.3774270730	5.4294938071
9	C	1.5313073511	0.7781686593	6.4604729818
10	H	1.7609113747	-0.1459561335	6.9893200335
11	H	0.4533932493	0.9274099108	6.4452522988
12	H	2.0069891056	1.6233720702	6.9563971265
13	C	1.6131935219	0.6663163334	1.5150610031
14	H	0.6379195787	1.1523964243	1.4976948806
15	H	2.3075062450	1.2958378262	0.9545197553
16	C	1.5588922741	-0.7481015971	0.9479570640
17	H	0.8228141209	-1.3339402807	1.5121748749
18	H	2.5320706280	-1.2271278741	1.1031024731
19	C	1.2109156976	-0.7930184320	-0.5477537627
20	H	1.9730757649	-0.2134626347	-1.0873534148
21	C	1.2886648327	-2.2422908482	-1.0321067554
22	H	2.2437808775	-2.6999364319	-0.7604589758

23	H	0.4871798614	-2.8429198753	-0.5889511503
24	H	1.1931345474	-2.3060690992	-2.1166279724
25	C	-0.1586316818	-0.1587577153	-0.8277120871
26	H	-0.1511816674	0.8940111695	-0.5179620860
27	H	-0.9204997791	-0.6583056738	-0.2130902064
28	C	-0.6005108926	-0.2016723058	-2.2914837874
29	H	0.1940537484	0.2105628771	-2.9264581935
30	H	-0.7393799697	-1.2395560610	-2.6023100446
31	C	-1.8799993535	0.5711248563	-2.5064081550
32	H	-1.7962758232	1.6246713590	-2.2455409123
33	C	-3.2237177383	-0.0596957453	-2.2067959057
34	H	-3.9821550435	0.5344187351	-1.7089620291
35	H	-3.2268619860	-1.1111905818	-1.9346128871
36	C	-2.8514792221	0.2868943039	-3.6266320502
37	C	-3.5343935728	1.4830819915	-4.2561131384
38	H	-3.7115111886	2.2751592826	-3.5234375484
39	H	-2.9225294758	1.8998455741	-5.0624089309
40	H	-4.5014949258	1.1976171559	-4.6811906503
41	C	-2.5825021622	-0.8366616207	-4.6060229077
42	H	-2.1801228045	-1.7298718838	-4.1239816923
43	H	-3.5115225067	-1.1279693763	-5.1048171265
44	H	-1.8751035063	-0.5195972341	-5.3790372340

Z-matrix Print:

$molecule

1 1

N

C 1 1.329019

H 2 1.078135 1 125.446437

N 2 1.331573 1 108.975641 3 -179.845859 0

C 1 1.375636 2 108.429889 3 -179.853320 0

H 5 1.077051 1 121.972605 2 179.924799 0

C 5 1.356616 1 107.180656 2 -0.330127 0

H 7 1.077086 5 130.946688 1 -179.475014 0

C 4 1.463014 2 125.957911 1 179.257760 0

H 9 1.088303 4 108.961018 2 1.168687 0

H 9 1.089222 4 109.425870 2 120.727282 0

H 9 1.089304 4 109.434175 2 -118.436174 0

C 1 1.473436 2 125.690746 3 -3.089837 0

H 13 1.089832 1 106.662279 2 21.419917 0

H 13 1.092050 1 107.138783 2 136.507711 0

C 13 1.524839 1 111.501137 2 -101.239897 0

H 16 1.095725 13 108.737427 1 -53.658465 0

H 16 1.096978 13 109.146354 1 62.544410 0

C 16 1.536312 13 113.407464 1 -175.140428 0

H 19 1.099064 16 107.788286 13 58.805283 0

C 19 1.530044 16 108.927968 13 175.734571 0

H 21 1.090587 19 111.453327 16 -171.654969 0

H 21 1.093379 19 111.235668 16 -51.820985 0

H 21 1.095226 19 110.743258 16 68.303345 0

C 19 1.535032 16 111.565357 13 -59.770458 0

H 25 1.097416 19 109.806585 16 60.645498 0

H 25 1.098977 19 109.189773 16 -55.697357 0

C 25 1.529616 19 114.877246 16 -178.204275 0

H 28 1.092291 25 109.615084 19 64.455269 0

H 28 1.097480 25 109.510697 19 -52.063554 0

C 28 1.510132 25 111.506019 19 -173.799869 0

H 31 1.088587 28 113.328332 25 60.254768 0

C 31 1.509789 28 123.672030 25 -154.343089 0

C 33 1.508185 31 60.235628 28 108.796434 0

H 34 1.084453 33 118.612813 31 108.674084 0

H 34 1.086156 33 117.329075 31 -107.048253 0

C 33 1.514425 31 116.735165 28 -143.310492 0

H 37 1.093422 33 111.512370 31 -33.320723 0

H 37 1.094288 33 110.728519 31 -153.384659 0

H 37 1.094615 33 110.783707 31 86.948761 0

C 33 1.514574 31 120.343075 28 0.810197 0

H 41 1.091832 33 112.757787 31 40.392121 0

H 41 1.093954 33 109.972249 31 160.027474 0

H 41 1.094757 33 110.885711 31 -80.974442 0

$end

\---

\- Entering anlman on Mon Jun 2 13:52:53 2025 -

\---

\--

Orbital Energies (a.u.)

\--

Alpha MOs

\-- Occupied --

-14.6914 -14.6862 -10.5476 -10.4971 -10.4962 -10.4873 -10.4807 -10.4136

-10.3967 -10.3739 -10.3646 -10.3609 -10.3603 -10.3554 -10.3432 -10.3330

-10.3305 -1.3452 -1.2228 -1.0700 -1.0465 -1.0402 -1.0139 -0.9724

-0.9342 -0.9041 -0.8833 -0.8802 -0.8640 -0.8500 -0.8307 -0.8051

-0.7692 -0.7512 -0.7398 -0.7287 -0.7233 -0.7099 -0.7013 -0.6937

-0.6878	-0.6761	-0.6535	-0.6519	-0.6369	-0.6323	-0.6105	-0.6020
-0.5903	-0.5800	-0.5708	-0.5604	-0.5520	-0.5417	-0.5338	-0.5305
-0.5236	-0.5174	-0.5098	-0.5052	-0.5000	-0.4943	-0.4782	-0.4237
-0.4098							

-- Virtual --

-0.1098	-0.0765	-0.0635	-0.0445	-0.0436	-0.0300	-0.0271	-0.0229
-0.0171	-0.0069	-0.0064	-0.0040	0.0044	0.0097	0.0117	0.0171
0.0238	0.0249	0.0273	0.0368	0.0384	0.0388	0.0451	0.0494
0.0506	0.0543	0.0555	0.0608	0.0657	0.0719	0.0752	0.0764
0.0780	0.0830	0.0856	0.0894	0.0920	0.0930	0.0978	0.1008
0.1048	0.1088	0.1122	0.1166	0.1182	0.1201	0.1252	0.1286
0.1319	0.1329	0.1371	0.1402	0.1415	0.1443	0.1475	0.1495
0.1556	0.1568	0.1579	0.1630	0.1652	0.1697	0.1714	0.1719
0.1768	0.1806	0.1821	0.1869	0.1895	0.1929	0.1950	0.1969
0.2046	0.2080	0.2099	0.2146	0.2197	0.2212	0.2244	0.2269
0.2307	0.2323	0.2378	0.2415	0.2467	0.2489	0.2517	0.2570
0.2612	0.2650	0.2669	0.2699	0.2727	0.2806	0.2865	0.2888
0.2937	0.2980	0.3043	0.3070	0.3153	0.3177	0.3295	0.3393
0.3482	0.3496	0.3532	0.3612	0.3681	0.3734	0.3774	0.3824
0.3882	0.4022	0.4150	0.4222	0.4284	0.4299	0.4379	0.4554
0.4659	0.4688	0.4778	0.4792	0.4813	0.4951	0.4969	0.5124
0.5184	0.5254	0.5309	0.5338	0.5422	0.5468	0.5499	0.5555
0.5667	0.5784	0.5853	0.5885	0.5967	0.5999	0.6059	0.6140
0.6177	0.6197	0.6233	0.6313	0.6351	0.6419	0.6545	0.6654
0.6679	0.6718	0.6764	0.6785	0.6797	0.6829	0.6869	0.6902
0.6941	0.6998	0.7040	0.7083	0.7111	0.7172	0.7229	0.7251
0.7301	0.7381	0.7399	0.7432	0.7460	0.7542	0.7576	0.7632
0.7717	0.7779	0.7879	0.7926	0.7977	0.8029	0.8115	0.8134
0.8392	0.8537	0.8646	0.8690	0.8832	0.9053	0.9083	0.9193
0.9326	0.9395	0.9467	0.9702	0.9806	0.9820	0.9920	1.0009

1.0073	1.0312	1.0539	1.0573	1.0695	1.0839	1.0867	1.1045
1.1119	1.1244	1.1420	1.1575	1.1841	1.1987	1.2137	1.2384
1.2556	1.2627	1.2830	1.3116	1.3319	1.3409	1.3463	1.3710
1.3711	1.3763	1.3846	1.4076	1.4202	1.4260	1.4331	1.4469
1.4553	1.4629	1.4649	1.4701	1.4787	1.4918	1.4970	1.5058
1.5165	1.5297	1.5354	1.5417	1.5487	1.5529	1.5540	1.5631
1.5662	1.5758	1.5795	1.5853	1.5977	1.6012	1.6090	1.6118
1.6152	1.6259	1.6377	1.6444	1.6472	1.6528	1.6545	1.6638
1.6736	1.6805	1.6902	1.6967	1.7038	1.7059	1.7141	1.7164
1.7280	1.7318	1.7349	1.7398	1.7464	1.7520	1.7601	1.7701
1.7768	1.7895	1.7909	1.7980	1.8216	1.8248	1.8348	1.8441
1.8487	1.8628	1.8933	1.8991	1.9180	1.9276	1.9379	1.9559
1.9649	1.9834	1.9950	2.0005	2.0177	2.0332	2.0419	2.0473
2.0534	2.0665	2.0831	2.0946	2.1032	2.1145	2.1231	2.1362
2.1416	2.1463	2.1574	2.1710	2.1804	2.1875	2.1958	2.2013
2.2187	2.2308	2.2571	2.2595	2.2718	2.2953	2.3087	2.3318
2.3407	2.3564	2.3712	2.3788	2.3844	2.4061	2.4187	2.4295
2.4350	2.4536	2.4572	2.4679	2.4740	2.4829	2.4909	2.4936
2.4972	2.5088	2.5096	2.5183	2.5224	2.5270	2.5335	2.5447
2.5542	2.5621	2.5726	2.5746	2.5823	2.5873	2.5984	2.6080
2.6101	2.6184	2.6225	2.6360	2.6435	2.6569	2.6601	2.6748
2.6838	2.6920	2.7007	2.7145	2.7214	2.7323	2.7365	2.7402
2.7495	2.7610	2.7770	2.7813	2.8011	2.8125	2.8182	2.8356
2.8474	2.8565	2.8637	2.8811	2.8837	2.9045	2.9144	2.9302
2.9339	2.9401	2.9523	2.9654	2.9697	3.0031	3.0118	3.0344
3.0365	3.0721	3.0780	3.0910	3.1374	3.1600	3.1915	3.2329
3.2641	3.2993	3.3513	3.3607	3.4585	3.5061	3.5272	3.5450
3.5935	3.6291	3.6398	3.6721	3.6826	3.7006	3.7494	3.7633
3.7894	3.8001	3.8208	3.8234	3.8285	3.8489	3.8530	3.8642
3.8692	3.8724	3.8791	3.8844	3.8977	3.9991	4.0573	4.0848

4.1469   4.2014   4.3142   4.3479   4.3811   4.5533   4.6418   4.6806
4.8800   4.9213   5.2165 23.7239 23.8468 23.8802 23.8824 23.8954
23.8994 23.9206 23.9374 23.9623 23.9744 24.0155 24.0313 24.0580
24.0670 24.1661 35.4375 35.5936

Ground-State Mulliken Net Atomic Charges

Atom	Charge (a.u.)
1 N	-0.002659
2 N	0.036027
3 C	-0.084601
4 H	0.215402
5 C	-0.131987
6 H	0.216322
7 C	-0.104249
8 H	0.212857
9 C	-0.264312
10 H	0.226065
11 H	0.193530
12 H	0.228386
13 C	-0.300421
14 H	0.185900
15 H	0.228709
16 C	-0.330682
17 H	0.192105
18 H	0.169469
19 C	0.010828
20 H	0.182331

21 C	-0.716112
22 H	0.157730
23 H	0.187582
24 H	0.185592
25 C	-0.274069
26 H	0.133989
27 H	0.178463
28 C	-0.334328
29 H	0.175306
30 H	0.135602
31 C	-0.068870
32 H	0.145827
33 C	-0.612749
34 H	0.170598
35 H	0.165458
36 C	0.378676
37 C	-0.495761
38 H	0.139208
39 H	0.166504
40 H	0.167287
41 C	-0.535688
42 H	0.128599
43 H	0.171113
44 H	0.171022

Sum of atomic charges = 1.000000

Cartesian Multipole Moments

Charge (ESU x 10^10)

4.8032

Dipole Moment (Debye)

X 8.3568 Y 2.4557 Z 16.7451

Tot 18.8750

Quadrupole Moments (Debye-Ang)

XX -75.1068 XY 3.3200 YY -101.8760

XZ 38.0213 YZ 13.4408 ZZ -3.4865

Traceless Quadrupole Moments (Debye-Ang)

QXX -44.8511 QYY -125.1587 QZZ 170.0097

QXY 9.9601 QXZ 114.0639 QYZ 40.3225

Octopole Moments (Debye-Ang^2)

XXX 50.0447 XXY 5.3070 XYY -17.7089

YYY -22.6657 XXZ 75.9973 XYZ 19.6271

YYZ -19.3713 XZZ 156.5443 YZZ 61.3800

ZZZ 470.9713

Traceless Octopole Moments (Debye-Ang^2)

XXX -949.2506 YYY -736.1775 ZZZ 2316.1942

XXY -52.4591 XXZ -442.8330 XYY -832.2744

XYZ 294.4070 XZZ 1781.5249 YYZ -1873.3612

YZZ 788.6366

Hexadecapole Moments (Debye-Ang^3)

XXXX -2916.1198 XXXY 61.9534 XXYY -681.5505

XYYY 43.3034 YYYY -737.3737 XXXZ -1593.4902

XXYZ -54.3191 XYYZ -678.2836 YYYZ -281.6627

XXZZ -1229.1669 XYZZ 119.2083 YYZZ -1342.8059

XZZZ -879.2286 YZZZ 113.4733 ZZZZ -4043.5853

Traceless Hexadecapole Moments (Debye-Ang^3)

XXXX 385.6391 XXXY -3595.8252 XXXZ -25521.3656

XXYY -346.6710 XXYZ -2358.7757 XXZZ -38.9682

XYYY	-5554.0745	XYYZ	-23954.7377	XYZZ	9149.8997
XZZZ	49476.1033	YYYY	43294.3436	YYYZ	-19540.4037
YYZZ	-42947.6727	YZZZ	21899.1793	ZZZZ	42986.6408

\---

Total job time: 326.24s(wall), 1871.30s(cpu)

Mon Jun 2 13:52:53 2025

The following are the XYZ coordinates for the 40 confomers of the Citronilyl IL cation.

Conformer 1 (Lowest Energy)

N	2.784783	0.037789	0.897456
N	1.192840	-0.301941	2.331063
C	1.911259	-0.856967	1.357177
H	1.799045	-1.866244	0.992330
C	1.613763	0.998810	2.493098
H	1.173706	1.652888	3.229430
C	2.613756	1.211956	1.597352
H	3.214109	2.085994	1.399601
C	3.712126	-0.165100	-0.214139
H	3.388233	0.439971	-1.063128
H	3.705962	-1.218440	-0.494492
H	4.716932	0.121330	0.097499
C	0.036803	-0.921428	3.003313
H	0.166802	-2.002682	2.928626
H	0.098765	-0.653362	4.060217
C	-1.279931	-0.454173	2.379853
H	-1.401337	0.617735	2.579715
H	-2.088776	-0.964388	2.917412
C	-1.398439	-0.711508	0.871316
H	-0.558294	-0.200740	0.380636
C	-1.337631	-2.205974	0.540449
H	-1.462082	-2.378610	-0.531107
H	-0.388282	-2.675679	0.825581
H	-2.136543	-2.747697	1.059886
C	-2.682640	-0.078023	0.313040
H	-2.836399	0.902363	0.784356
H	-3.543451	-0.695483	0.601002

C	-2.674240	0.122362	-1.208614
H	-2.515824	-0.831787	-1.724768
H	-3.670579	0.460219	-1.516736
C	-1.663121	1.155384	-1.663410
H	-1.761929	2.096083	-1.120198
C	-1.284619	1.313679	-3.113335
H	-1.218629	2.314587	-3.530193
H	-1.660234	0.567887	-3.809899
C	-0.242363	0.866985	-2.119588
C	0.814668	1.870251	-1.704633
H	0.424008	2.892755	-1.701535
H	1.174736	1.656302	-0.687307
H	1.673290	1.848147	-2.388493
C	0.286155	-0.547816	-2.236597
H	0.724142	-0.894541	-1.288523
H	-0.489460	-1.264564	-2.520691
H	1.068920	-0.596333	-3.003022

Conformer 2

N	2.097273	1.632988	1.291037
N	0.976643	0.328424	2.609439
C	0.900708	1.091498	1.520626
H	0.024008	1.221543	0.900654
C	2.267299	0.376156	3.087442
H	2.568774	-0.167902	3.968302
C	2.970204	1.198907	2.265716
H	4.001288	1.515902	2.292855
C	2.424556	2.535503	0.188264
H	2.659444	3.525481	0.581790

H	1.564836	2.601238	-0.477798
H	3.276886	2.136257	-0.363804
C	-0.103487	-0.530392	3.122429
H	0.027936	-0.589665	4.205107
H	-1.042994	-0.006763	2.935833
C	-0.075833	-1.921315	2.486476
H	-0.905867	-2.486723	2.928451
H	0.845462	-2.429702	2.797245
C	-0.185719	-1.955888	0.953376
H	0.633727	-1.347709	0.541652
C	0.032823	-3.391636	0.464443
H	0.000387	-3.454276	-0.626542
H	1.006232	-3.775804	0.787058
H	-0.741161	-4.058810	0.860881
C	-1.528220	-1.390806	0.456270
H	-1.796623	-0.474875	1.002899
H	-2.313218	-2.119607	0.696603
C	-1.585479	-1.058330	-1.040315
H	-1.278033	-1.925307	-1.637392
H	-2.632194	-0.872306	-1.300551
C	-0.747747	0.143426	-1.431095
H	0.320404	0.022461	-1.238313
C	-1.299183	1.552253	-1.262260
H	-0.659965	2.366511	-0.921613
H	-2.332080	1.646361	-0.933856
C	-1.048216	0.992967	-2.642029
C	0.146318	1.539188	-3.400427
H	0.995558	1.735093	-2.733907
H	0.483073	0.826633	-4.161815
H	-0.107158	2.477670	-3.906120

C	-2.232282	0.678134	-3.535446
H	-1.992838	-0.141563	-4.221779
H	-3.125897	0.398872	-2.971438
H	-2.489646	1.555915	-4.138350

Confomer 03

N	2.842435	-0.136005	0.966330
N	1.115603	-0.875759	2.050664
C	2.003384	-1.162573	1.101042
H	2.035043	-2.078983	0.531935
C	1.386616	0.385599	2.530788
H	0.793534	0.837746	3.309688
C	2.470620	0.848910	1.854314
H	3.008598	1.780496	1.930688
C	3.955494	-0.053429	0.021982
H	3.762787	0.748319	-0.692693
H	4.040040	-1.000861	-0.509113
H	4.879512	0.139570	0.568683
C	-0.031363	-1.715387	2.439615
H	0.239074	-2.748824	2.213469
H	-0.131157	-1.630117	3.523803
C	-1.308839	-1.281399	1.721760
H	-1.525368	-0.245473	2.007123
H	-2.127955	-1.889277	2.124936
C	-1.265556	-1.422421	0.193187
H	-0.349699	-0.937856	-0.176575
C	-1.237306	-2.890783	-0.240742
H	-1.184102	-2.970334	-1.330698
H	-0.379162	-3.440091	0.165740

H	-2.143570	-3.412507	0.085768
C	-2.439128	-0.686883	-0.470229
H	-3.384649	-1.135264	-0.137550
H	-2.375040	-0.873218	-1.548176
C	-2.482231	0.832762	-0.231548
H	-3.197496	1.269754	-0.936634
H	-2.891538	1.054045	0.762463
C	-1.136061	1.518521	-0.358911
H	-0.480132	1.349317	0.496907
C	-0.989597	2.881647	-0.988655
H	-0.322644	3.603531	-0.525523
H	-1.876551	3.323769	-1.434273
C	-0.393972	1.675203	-1.669285
C	1.112661	1.517146	-1.620482
H	1.518702	1.917234	-0.682661
H	1.393262	0.456503	-1.697861
H	1.594870	2.049527	-2.448265
C	-1.009570	1.192074	-2.967302
H	-0.765640	0.139076	-3.153489
H	-2.098554	1.290585	-2.969897
H	-0.625353	1.776112	-3.810321

Confomer 04

N	2.970076	0.126107	0.404871
N	1.533874	1.001235	1.773491
C	2.116868	-0.134158	1.395347
H	1.929381	-1.106693	1.823388
C	2.023150	2.022163	0.989588
H	1.694559	3.041900	1.113082

C	2.924545	1.475001	0.132492
H	3.532336	1.922916	-0.637577
C	3.772092	-0.859298	-0.317268
H	3.406333	-0.936944	-1.342911
H	3.677244	-1.825006	0.178644
H	4.818764	-0.550614	-0.311086
C	0.471776	1.137831	2.785797
H	0.667685	0.396170	3.563145
H	0.601713	2.125001	3.234068
C	-0.919384	0.979531	2.172108
H	-0.991179	1.659884	1.315412
H	-1.642477	1.339941	2.913971
C	-1.291471	-0.454459	1.762917
H	-0.468621	-0.879527	1.171068
C	-1.517329	-1.342658	2.990190
H	-1.727822	-2.373123	2.688370
H	-0.650589	-1.372832	3.661587
H	-2.373486	-0.987005	3.574943
C	-2.537872	-0.456396	0.861323
H	-3.340064	0.105233	1.359211
H	-2.898687	-1.489415	0.777314
C	-2.323914	0.096560	-0.554197
H	-3.288269	0.086532	-1.076708
H	-2.020176	1.150068	-0.512352
C	-1.317648	-0.710398	-1.349968
H	-1.512496	-1.782429	-1.314593
C	0.149478	-0.334228	-1.395606
H	0.892895	-1.127351	-1.332413
H	0.437263	0.601511	-0.917612
C	-0.698137	-0.237855	-2.641923

C	-0.473935	-1.279869	-3.720015
H	-0.238875	-2.259886	-3.290760
H	-1.369407	-1.393350	-4.340763
H	0.354193	-0.990430	-4.377661
C	-1.029982	1.137105	-3.187452
H	-1.974875	1.117068	-3.742305
H	-1.120554	1.891384	-2.400623
H	-0.246977	1.470786	-3.878534

Conformer 05

N	3.024272	0.611411	0.275055
N	1.600347	0.516942	1.908182
C	2.136015	1.308689	0.981320
H	1.887049	2.347212	0.824762
C	2.159396	-0.734359	1.785210
H	1.875633	-1.549509	2.431722
C	3.052961	-0.676374	0.762468
H	3.703489	-1.429498	0.346857
C	3.779474	1.103135	-0.875543
H	4.832970	0.850941	-0.749240
H	3.672250	2.186960	-0.935940
H	3.384413	0.646507	-1.785588
C	0.502988	0.896633	2.816100
H	0.609373	0.279784	3.710073
H	0.675393	1.934356	3.110957
C	-0.863253	0.729168	2.150793
H	-0.897307	1.391385	1.277922
H	-1.611017	1.109401	2.858284
C	-1.224262	-0.707493	1.745246

H	-0.412623	-1.106177	1.121261
C	-1.387041	-1.614711	2.968432
H	-1.626463	-2.637170	2.661209
H	-0.481850	-1.668306	3.586003
H	-2.201878	-1.262266	3.611605
C	-2.500488	-0.733046	0.887499
H	-3.324852	-0.274650	1.450070
H	-2.781717	-1.783080	0.737749
C	-2.388666	-0.063037	-0.489515
H	-3.342154	-0.203177	-1.012632
H	-2.269687	1.021410	-0.379408
C	-1.265569	-0.633947	-1.332222
H	-1.233175	-1.723837	-1.316635
C	0.090373	0.038692	-1.412849
H	0.984801	-0.581432	-1.386401
H	0.191754	1.006494	-0.923450
C	-0.802512	-0.021265	-2.629977
C	-0.405009	-0.972941	-3.741383
H	0.046742	-1.889560	-3.346454
H	-1.279696	-1.261493	-4.334639
H	0.317654	-0.503445	-4.419160
C	-1.434644	1.263413	-3.128909
H	-2.372277	1.054142	-3.655301
H	-1.656776	1.965703	-2.320048
H	-0.764433	1.768397	-3.833484

Conformer 06

N	2.587878	1.142772	1.358117
N	1.248731	-0.328542	2.223482

C	1.532063	0.971169	2.153657
H	0.995043	1.758644	2.659592
C	2.147653	-1.009923	1.435768
H	2.114400	-2.082310	1.329914
C	2.988451	-0.089728	0.893329
H	3.824676	-0.202906	0.221890
C	3.197531	2.423641	1.003031
H	4.253755	2.409435	1.275120
H	2.692483	3.220679	1.548262
H	3.084496	2.588225	-0.070105
C	0.101285	-0.913411	2.945976
H	0.260473	-1.992606	2.945690
H	0.153082	-0.570720	3.982026
C	-1.226435	-0.532555	2.291340
H	-1.391782	0.544500	2.426161
H	-2.019638	-1.036384	2.857526
C	-1.329880	-0.886109	0.801447
H	-0.511085	-0.371072	0.279530
C	-1.192664	-2.393163	0.565917
H	-1.346653	-2.643746	-0.486342
H	-0.201880	-2.777340	0.835945
H	-1.936159	-2.945958	1.152306
C	-2.645985	-0.347599	0.217950
H	-2.844948	0.648298	0.637631
H	-3.474464	-0.988993	0.545554
C	-2.656765	-0.227553	-1.311970
H	-2.488137	-1.205170	-1.778014
H	-3.661198	0.079971	-1.625670
C	-1.659057	0.791170	-1.823487
H	-1.746445	1.749415	-1.308605

C	-1.302463	0.905768	-3.283452
H	-1.240372	1.895134	-3.727546
H	-1.689686	0.143008	-3.954704
C	-0.248659	0.479396	-2.293961
C	0.825055	1.480280	-1.917908
H	0.442853	2.506668	-1.924412
H	1.199626	1.265075	-0.906017
H	1.674312	1.432265	-2.610386
C	0.264592	-0.943994	-2.371799
H	0.706459	-1.265701	-1.418656
H	-0.521846	-1.657607	-2.632795
H	1.041306	-1.022424	-3.141333

Conformer 07

N	2.482145	1.903651	0.862568
N	1.527356	0.510734	2.222330
C	1.328838	1.317874	1.183092
H	0.390644	1.462454	0.668720
C	2.857137	0.573823	2.574174
H	3.257231	0.000413	3.395071
C	3.456096	1.450646	1.725654
H	4.477062	1.794000	1.666434
C	2.672172	2.871805	-0.216651
H	2.972414	3.832922	0.203662
H	1.730525	2.986655	-0.755214
H	3.439108	2.504110	-0.899412
C	0.521224	-0.394227	2.804868
H	0.670333	-0.381791	3.886817
H	-0.456457	0.046803	2.602896

C	0.644380	-1.811793	2.244133
H	-0.151296	-2.405983	2.710373
H	1.592792	-2.243294	2.587815
C	0.555528	-1.932663	0.714311
H	1.360892	-1.319864	0.277544
C	0.820746	-3.385965	0.308122
H	0.879098	-3.494339	-0.777778
H	1.766176	-3.746971	0.725787
H	0.020619	-4.042143	0.669413
C	-0.783062	-1.418449	0.163467
H	-0.993449	-0.407568	0.540084
H	-1.596342	-2.049321	0.546891
C	-0.852133	-1.359895	-1.365499
H	0.031991	-0.831567	-1.748966
H	-0.797968	-2.370436	-1.786187
C	-2.119300	-0.701133	-1.865416
H	-3.018372	-1.269370	-1.629066
C	-2.139803	0.028523	-3.184639
H	-2.995303	-0.096626	-3.841668
H	-1.191929	0.134187	-3.708350
C	-2.326567	0.800722	-1.902093
C	-3.716207	1.318613	-1.586936
H	-4.487672	0.627061	-1.940012
H	-3.853781	1.448317	-0.506022
H	-3.892612	2.287870	-2.068045
C	-1.226742	1.747510	-1.461554
H	-1.342023	1.994118	-0.394739
H	-0.227086	1.328620	-1.630645
H	-1.286403	2.691968	-2.015335

Conformer 08

N	2.126535	1.771413	1.256783
N	0.374070	0.923499	2.211807
C	1.660453	0.720641	1.931681
H	2.233441	-0.151462	2.206215
C	0.003637	2.140320	1.688829
H	-0.999804	2.522046	1.789525
C	1.101179	2.673703	1.090766
H	1.239856	3.605536	0.566002
C	3.483744	1.927912	0.736749
H	3.451238	1.932402	-0.354588
H	4.095558	1.093721	1.081166
H	3.904959	2.864170	1.107563
C	-0.504021	-0.001885	2.954972
H	-0.146432	-0.042665	3.987058
H	-1.487604	0.468998	2.966187
C	-0.557155	-1.396892	2.333362
H	-1.323637	-1.949603	2.890612
H	0.388082	-1.918662	2.533350
C	-0.858839	-1.459523	0.827308
H	-0.045471	-0.943871	0.295365
C	-0.854450	-2.927961	0.391252
H	-1.053208	-3.030919	-0.677731
H	0.112231	-3.402043	0.593143
H	-1.624816	-3.492368	0.929458
C	-2.176847	-0.765251	0.452223
H	-2.259706	0.202159	0.968319
H	-3.017143	-1.372974	0.812661
C	-2.342622	-0.484362	-1.047731

H	-2.338032	-1.419776	-1.616808
H	-3.334237	-0.044165	-1.206741
C	-1.293177	0.472770	-1.575966
H	-1.160411	1.342358	-0.928009
C	-1.145906	0.771762	-3.046841
H	-1.006105	1.803804	-3.356416
H	-1.741322	0.180081	-3.737529
C	-0.032498	0.065640	-2.318784
C	1.226337	0.848488	-2.007485
H	1.000807	1.894124	-1.766210
H	1.747027	0.396363	-1.150150
H	1.917904	0.841076	-2.857672
C	0.247021	-1.382203	-2.665007
H	0.750239	-1.900694	-1.839764
H	-0.663015	-1.937226	-2.907648
H	0.902138	-1.438480	-3.541277

Conformer 09

N	-1.330267	1.944449	1.796447
N	-0.246999	0.347902	2.784256
C	-0.129375	1.391496	1.967120
H	0.785397	1.722846	1.500937
C	-1.570437	0.219609	3.140488
H	-1.910645	-0.565656	3.796855
C	-2.249827	1.221491	2.524286
H	-3.294934	1.487488	2.547606
C	-1.623773	3.139677	1.007337
H	-2.386345	2.902915	0.263784
H	-0.715740	3.460568	0.498322

H	-1.976669	3.932591	1.668454
C	0.835311	-0.571490	3.172315
H	1.774565	-0.042821	2.990400
H	0.744319	-0.724995	4.250420
C	0.805721	-1.897651	2.415698
H	-0.121100	-2.438452	2.647515
H	1.617905	-2.492428	2.849710
C	0.994915	-1.784763	0.888978
H	1.601241	-0.886604	0.692277
C	1.774583	-2.995023	0.364331
H	1.966711	-2.915943	-0.708134
H	2.743460	-3.089619	0.864661
H	1.213590	-3.920721	0.536670
C	-0.353795	-1.629761	0.162250
H	-0.929329	-0.807723	0.609553
H	-0.943733	-2.540672	0.334073
C	-0.261008	-1.371020	-1.346661
H	0.157389	-2.245685	-1.857551
H	-1.281050	-1.264994	-1.729655
C	0.560347	-0.148609	-1.697925
H	1.623827	-0.260713	-1.488678
C	0.009508	1.245177	-1.471385
H	0.662916	2.021897	-1.076001
H	-1.031401	1.320578	-1.159955
C	0.268821	0.749712	-2.875650
C	1.466112	1.328314	-3.604464
H	2.305991	1.509210	-2.924063
H	1.811199	0.642537	-4.386501
H	1.208556	2.280253	-4.082670
C	-0.915602	0.478954	-3.782136

H	-0.678977	-0.308798	-4.505643
H	-1.810408	0.173410	-3.232083
H	-1.170971	1.383066	-4.345589

Conformer 10

N	2.731631	1.522065	2.245788
N	0.787474	0.582360	2.450373
C	1.981761	0.736797	3.019067
H	2.290646	0.301143	3.956978
C	0.772319	1.288975	1.268322
H	-0.085862	1.292039	0.611094
C	1.989614	1.879639	1.140755
H	2.387602	2.519385	0.368494
C	4.100505	1.950920	2.530307
H	4.121437	3.031266	2.681012
H	4.744374	1.674917	1.694474
H	4.448719	1.451187	3.433966
C	-0.300988	-0.268430	2.963436
H	-0.197826	-0.296824	4.050531
H	-1.233001	0.251590	2.737252
C	-0.262742	-1.678764	2.374099
H	-1.091110	-2.232133	2.834598
H	0.660667	-2.169262	2.707821
C	-0.368774	-1.772715	0.843388
H	0.453870	-1.184938	0.411731
C	-0.159673	-3.227433	0.411872
H	-0.150162	-3.323998	-0.677040
H	0.793368	-3.618280	0.784136
H	-0.961154	-3.867768	0.798289

C	-1.705836	-1.214451	0.327829
H	-1.911986	-0.230918	0.774449
H	-2.510120	-1.876357	0.676021
C	-1.808293	-1.048016	-1.193514
H	-1.640139	-2.008588	-1.694659
H	-2.840185	-0.767446	-1.426463
C	-0.855840	-0.011956	-1.755175
H	0.197683	-0.252980	-1.608112
C	-1.196900	1.468743	-1.699979
H	-0.417836	2.197458	-1.480720
H	-2.181098	1.745541	-1.327111
C	-1.128768	0.752663	-3.027744
C	0.065590	1.051995	-3.913249
H	0.978041	1.201512	-3.324441
H	0.248736	0.227003	-4.610923
H	-0.106064	1.959018	-4.503476
C	-2.412222	0.529471	-3.803882
H	-2.342141	-0.371646	-4.423507
H	-3.287255	0.428035	-3.156462
H	-2.598059	1.379182	-4.469629

Conformer 11

N	-2.060615	-0.566020	1.484641
N	-0.138296	-1.464190	1.933999
C	-1.216077	-1.543193	1.158258
H	-1.379133	-2.276412	0.383697
C	-0.301438	-0.399979	2.793089
H	0.446408	-0.137622	3.524676
C	-1.503890	0.165776	2.509966

H	-2.008430	1.013683	2.945761
C	-3.338657	-0.286814	0.832471
H	-3.268426	0.661970	0.297980
H	-4.126545	-0.242937	1.585382
H	-3.557610	-1.087138	0.125771
C	1.069348	-2.308263	1.848179
H	1.241635	-2.695812	2.854896
H	0.818090	-3.157855	1.208779
C	2.297344	-1.543576	1.335179
H	3.175304	-2.037601	1.765208
H	2.288253	-0.533494	1.761142
C	2.488762	-1.483914	-0.194894
H	3.345375	-0.815491	-0.353517
C	2.867938	-2.857591	-0.758230
H	3.096931	-2.786715	-1.825611
H	3.748657	-3.268784	-0.254669
H	2.051481	-3.584139	-0.654303
C	1.299553	-0.900354	-0.970792
H	0.446904	-1.593223	-0.900328
H	1.558159	-0.887722	-2.036403
C	0.860706	0.510058	-0.569460
H	0.665341	0.571308	0.509517
H	1.689865	1.201313	-0.758416
C	-0.373454	0.948399	-1.329267
H	-1.191919	0.225721	-1.272048
C	-0.260085	1.669373	-2.652150
H	-0.939522	1.400451	-3.455881
H	0.739489	1.936669	-2.986858
C	-0.812361	2.391777	-1.450179
C	-2.303273	2.667522	-1.430223

H	-2.873314	1.848142	-1.884282
H	-2.663089	2.816962	-0.402753
H	-2.539804	3.578838	-1.989682
C	0.009838	3.491106	-0.806813
H	-0.226351	3.586525	0.260346
H	1.085757	3.320092	-0.899831
H	-0.208850	4.453159	-1.282347

Conformer 12

N	3.620909	1.398031	1.237451
N	1.685620	0.596640	1.801126
C	2.973594	0.653570	2.134435
H	3.419802	0.178348	2.995240
C	1.502590	1.323358	0.646124
H	0.535045	1.415437	0.176184
C	2.714170	1.828328	0.293597
H	3.005856	2.453469	-0.536075
C	5.048397	1.716457	1.255190
H	5.518230	1.320303	0.353953
H	5.503952	1.259186	2.133492
H	5.177205	2.799275	1.302294
C	0.656037	-0.190199	2.504679
H	0.889280	-0.144015	3.571064
H	-0.289291	0.333694	2.353940
C	0.603901	-1.637864	2.016240
H	-0.178266	-2.138197	2.601193
H	1.549055	-2.129158	2.281954
C	0.328924	-1.833751	0.516614
H	1.110870	-1.301822	-0.048510

C	0.449368	-3.322069	0.174848
H	0.358213	-3.493679	-0.900305
H	1.417030	-3.725382	0.491646
H	-0.336503	-3.899266	0.675367
C	-1.033618	-1.260240	0.101367
H	-1.141815	-0.225732	0.456095
H	-1.829040	-1.828120	0.604108
C	-1.286966	-1.249541	-1.408642
H	-0.449425	-0.750526	-1.914954
H	-1.305568	-2.273238	-1.799625
C	-2.595562	-0.575583	-1.758193
H	-3.465243	-1.120267	-1.391498
C	-2.785571	0.131480	-3.076108
H	-3.726910	0.009073	-3.604091
H	-1.918880	0.209500	-3.728882
C	-2.776157	0.928894	-1.795311
C	-4.097022	1.481193	-1.296855
H	-4.923617	0.797200	-1.514622
H	-4.072739	1.641237	-0.211496
H	-4.324998	2.441834	-1.773447
C	-1.605112	1.855033	-1.531490
H	-1.525100	2.083781	-0.457869
H	-0.653602	1.434590	-1.878323
H	-1.747044	2.808738	-2.051905

Conformer 13

N	-0.153949	-3.443748	0.916155
N	0.872781	-1.646913	1.561558
C	-0.246803	-2.117796	1.019290

H	-1.090586	-1.518607	0.711588
C	1.714034	-2.706097	1.815562
H	2.689544	-2.569364	2.254343
C	1.071252	-3.833566	1.412733
H	1.373526	-4.868607	1.438361
C	-1.177541	-4.337190	0.374368
H	-0.770242	-4.874726	-0.482976
H	-2.034914	-3.743334	0.056991
H	-1.489534	-5.041355	1.146991
C	1.177301	-0.223134	1.787973
H	0.259432	0.324115	1.565308
H	1.384672	-0.106985	2.854737
C	2.348220	0.289711	0.951158
H	3.264412	-0.238195	1.243721
H	2.492114	1.328054	1.270119
C	2.196403	0.242547	-0.583585
H	2.993305	0.893598	-0.966205
C	2.445652	-1.150309	-1.177924
H	2.542965	-1.083582	-2.265613
H	3.368501	-1.595876	-0.791019
H	1.621342	-1.844801	-0.975487
C	0.862848	0.813896	-1.085092
H	0.031543	0.164567	-0.769460
H	0.861528	0.762316	-2.181012
C	0.561790	2.254872	-0.663185
H	0.526135	2.341113	0.430262
H	1.385026	2.906883	-0.983104
C	-0.734647	2.759539	-1.257705
H	-0.706390	2.827990	-2.344677
C	-1.560865	3.813129	-0.565018

H	-2.012294	4.598362	-1.163939
H	-1.231305	4.141272	0.418224
C	-2.096985	2.408510	-0.693546
C	-3.215977	2.162917	-1.685951
H	-3.108142	2.794277	-2.573314
H	-3.223834	1.117728	-2.020024
H	-4.192365	2.380980	-1.238363
C	-2.215124	1.570668	0.563796
H	-2.172241	0.497019	0.322594
H	-1.433051	1.799136	1.295709
H	-3.177537	1.750984	1.055660

Conformer 14

N	1.205085	2.163791	1.658112
N	0.028036	0.519995	2.442110
C	-0.014036	1.628526	1.707688
H	-0.884101	2.016404	1.201645
C	1.323020	0.336821	2.877641
H	1.605457	-0.498385	3.498078
C	2.061063	1.365534	2.385859
H	3.108334	1.600287	2.493392
C	1.586443	3.345725	0.888020
H	0.683521	3.864879	0.568528
H	2.160619	3.039252	0.011846
H	2.183675	4.007701	1.515494
C	-1.101696	-0.392130	2.697538
H	-1.301872	-0.368501	3.772012
H	-1.967500	0.033225	2.190812
C	-0.806608	-1.821762	2.239393

H	-1.730035	-2.392979	2.395817
H	-0.072870	-2.255285	2.930917
C	-0.305643	-2.035804	0.798383
H	0.579883	-1.403619	0.643344
C	0.154955	-3.493605	0.665817
H	0.595477	-3.672691	-0.319257
H	0.905696	-3.750908	1.420374
H	-0.689883	-4.182016	0.783915
C	-1.343665	-1.726087	-0.298617
H	-2.297300	-2.194903	-0.021006
H	-1.017123	-2.229671	-1.216437
C	-1.589962	-0.254295	-0.660795
H	-2.380473	-0.221029	-1.416896
H	-2.010304	0.295055	0.192300
C	-0.361263	0.461236	-1.182286
H	0.508801	0.387865	-0.528919
C	-0.483156	1.768605	-1.938419
H	0.231313	2.567224	-1.749543
H	-1.486280	2.118739	-2.171248
C	0.006601	0.530136	-2.648047
C	1.485049	0.458881	-2.977824
H	1.698820	0.975599	-3.920090
H	2.096275	0.924700	-2.195010
H	1.812587	-0.582203	-3.083322
C	-0.877915	-0.110226	-3.699389
H	-0.680921	-1.186005	-3.774370
H	-1.943864	0.027437	-3.498171
H	-0.674239	0.334488	-4.679390

Conformer 15

N	0.428828	-0.636845	3.974142
N	0.844363	-1.360741	1.972220
C	0.063161	-0.560229	2.693601
H	-0.739139	0.047487	2.301064
C	1.743327	-1.970874	2.817788
H	2.483891	-2.667561	2.457532
C	1.482901	-1.519747	4.073163
H	1.949530	-1.747826	5.019100
C	-0.192879	0.077131	5.088346
H	-0.669335	-0.637832	5.759587
H	-0.940978	0.763065	4.694494
H	0.569332	0.643273	5.624733
C	0.781853	-1.518439	0.509569
H	0.707250	-2.588464	0.303062
H	-0.150891	-1.049241	0.191337
C	1.986749	-0.880107	-0.179153
H	2.900130	-1.406431	0.124519
H	2.084643	0.151460	0.176733
C	1.882729	-0.903380	-1.717352
H	2.759088	-0.348964	-2.077548
C	1.973035	-2.328797	-2.272180
H	2.042145	-2.310199	-3.363715
H	2.856491	-2.853937	-1.892821
H	1.086706	-2.923735	-2.018919
C	0.629458	-0.189367	-2.247874
H	-0.274111	-0.742190	-1.951238
H	0.647736	-0.243112	-3.343473
C	0.486015	1.277793	-1.833249
H	0.550683	1.380853	-0.742235

H	1.328440	1.856559	-2.234403
C	-0.815922	1.874824	-2.317990
H	-0.921947	1.849598	-3.402335
C	-1.437694	3.067850	-1.636871
H	-1.885176	3.844403	-2.250532
H	-0.949756	3.441189	-0.738395
C	-2.120549	1.723770	-1.561591
C	-3.380992	1.516267	-2.377681
H	-3.326737	2.046137	-3.333928
H	-3.539375	0.452829	-2.593659
H	-4.262139	1.886981	-1.841502
C	-2.151765	1.008131	-0.223759
H	-2.238166	-0.079772	-0.360017
H	-1.252611	1.222314	0.367815
H	-3.018322	1.335875	0.363614

Conformer 16

N	3.002904	2.660930	-0.030495
N	2.298895	0.920800	1.054043
C	1.917926	2.005697	0.383750
H	0.895084	2.304478	0.205832
C	3.675710	0.874925	1.064706
H	4.217511	0.080586	1.553019
C	4.117963	1.964463	0.384849
H	5.117985	2.303774	0.165382
C	3.011488	3.902161	-0.804236
H	3.558590	4.666982	-0.252677
H	1.984519	4.232145	-0.957601
H	3.484878	3.724881	-1.769901

C	1.395567	-0.081440	1.648575
H	1.598473	-0.104125	2.722346
H	0.379934	0.294672	1.506126
C	1.570685	-1.460939	1.019067
H	0.838758	-2.115913	1.506144
H	2.554079	-1.865208	1.290242
C	1.400397	-1.517083	-0.508373
H	2.248636	-0.977796	-0.956825
C	1.488469	-2.971813	-0.978504
H	1.415056	-3.033046	-2.068652
H	2.438314	-3.428107	-0.680146
H	0.681369	-3.577778	-0.552932
C	0.120490	-0.824683	-1.006573
H	0.083492	-0.918445	-2.099131
H	0.181070	0.256467	-0.815842
C	-1.199430	-1.330038	-0.416360
H	-1.382965	-2.364706	-0.728997
H	-1.142498	-1.349977	0.678963
C	-2.353262	-0.451463	-0.845806
H	-2.502003	-0.439045	-1.925424
C	-2.620040	0.866605	-0.149965
H	-2.882995	1.737443	-0.744896
H	-2.034912	1.096002	0.741580
C	-3.614354	-0.266003	-0.039190
C	-4.911531	-0.137375	-0.813681
H	-4.757850	0.355046	-1.779774
H	-5.348225	-1.124015	-1.005335
H	-5.644039	0.450667	-0.249479
C	-3.779887	-0.956661	1.300818
H	-4.059915	-2.008044	1.167187

H	-2.870448	-0.925173	1.910439
H	-4.573887	-0.469843	1.877730

Conformer 17

N	3.242153	2.720129	0.598336
N	2.336514	1.133073	1.768723
C	2.087151	2.160949	0.961236
H	1.107453	2.487380	0.648453
C	3.700153	1.024039	1.922973
H	4.145007	0.257627	2.538153
C	4.268886	2.019270	1.192096
H	5.303370	2.290822	1.049976
C	3.397054	3.881497	-0.276954
H	3.885624	4.688064	0.272636
H	2.411325	4.211272	-0.606803
H	3.995293	3.601435	-1.145873
C	1.338173	0.200500	2.322038
H	1.558917	0.092603	3.386444
H	0.364390	0.686408	2.231161
C	1.369425	-1.146226	1.603498
H	0.635244	-1.787444	2.104046
H	2.345895	-1.618671	1.770257
C	1.079713	-1.087743	0.095773
H	1.866902	-0.477884	-0.374563
C	1.191215	-2.494032	-0.502710
H	0.989891	-2.474128	-1.577999
H	2.194401	-2.907393	-0.355623
H	0.478514	-3.185700	-0.041570
C	-0.269356	-0.427884	-0.243968

H	-0.396690	-0.456488	-1.332355
H	-0.249032	0.643023	0.013135
C	-1.504650	-1.055885	0.409001
H	-1.529856	-2.129701	0.196075
H	-1.450525	-0.970744	1.503731
C	-2.786031	-0.404343	-0.061876
H	-2.877263	0.638147	0.244903
C	-4.080063	-1.179154	-0.112559
H	-4.994201	-0.689259	0.210509
H	-4.034966	-2.238508	0.129445
C	-3.431227	-0.723793	-1.395475
C	-4.081514	0.427793	-2.137033
H	-4.516326	1.156927	-1.444995
H	-3.351757	0.951812	-2.766435
H	-4.885456	0.066312	-2.788110
C	-2.825729	-1.767640	-2.314107
H	-2.068239	-1.323340	-2.972059
H	-2.357889	-2.592016	-1.767207
H	-3.601895	-2.201110	-2.954323

Conformer 18

N	3.273415	-0.108546	1.197417
N	1.670113	-0.782737	2.493004
C	2.523158	-1.155332	1.542175
H	2.591642	-2.143606	1.114477
C	1.872169	0.552551	2.759142
H	1.287163	1.083207	3.493145
C	2.878637	0.975671	1.950520
H	3.347075	1.941924	1.849844

C	4.347966	-0.113452	0.205002
H	4.164554	0.675333	-0.525779
H	4.358636	-1.078260	-0.301654
H	5.305826	0.049605	0.701484
C	0.587450	-1.613518	3.049690
H	0.809664	-2.646266	2.774234
H	0.646313	-1.539237	4.137958
C	-0.773983	-1.156572	2.524777
H	-0.993563	-0.162599	2.934637
H	-1.526068	-1.835227	2.945649
C	-0.891947	-1.117942	0.994296
H	-0.075288	-0.489428	0.608097
C	-0.770332	-2.512885	0.374197
H	-0.797431	-2.465246	-0.718495
H	0.164752	-3.017739	0.646763
H	-1.596639	-3.156058	0.699849
C	-2.202107	-0.422198	0.597578
H	-2.236844	0.552619	1.101860
H	-3.052121	-1.003510	0.979617
C	-2.376893	-0.191737	-0.909666
H	-2.564002	-1.143747	-1.421211
H	-3.284425	0.402695	-1.052687
C	-1.186652	0.492799	-1.550303
H	-0.337052	-0.172407	-1.707865
C	-0.811588	1.916267	-1.190195
H	0.236879	2.162655	-1.037934
H	-1.494260	2.468495	-0.548071
C	-1.286421	1.590303	-2.585293
C	-0.250818	1.578265	-3.692864
H	0.711744	1.191266	-3.338851

H	-0.578993	0.948317	-4.527391
H	-0.083256	2.589334	-4.081257
C	-2.640513	2.107655	-3.028518
H	-3.105361	1.425429	-3.751241
H	-3.333073	2.239990	-2.191580
H	-2.527523	3.083869	-3.514557

Conformer 19

N	4.507097	-0.473714	0.046316
N	2.805947	-0.951627	1.303863
C	3.623209	-1.420397	0.364303
H	3.573542	-2.405905	-0.072679
C	3.172484	0.343164	1.592338
H	2.648783	0.933555	2.327385
C	4.240786	0.642630	0.808053
H	4.832464	1.540808	0.730343
C	5.584328	-0.598122	-0.935642
H	5.468182	0.173555	-1.698299
H	5.524698	-1.579187	-1.402781
H	6.546867	-0.489912	-0.435037
C	1.637727	-1.655435	1.862522
H	1.766496	-2.714526	1.631913
H	1.686375	-1.543674	2.948118
C	0.331291	-1.097616	1.300501
H	0.229191	-0.057877	1.630720
H	-0.484882	-1.653183	1.778688
C	0.187198	-1.185965	-0.226483
H	1.037664	-0.656758	-0.686266
C	0.197135	-2.638561	-0.713633

H	0.085715	-2.681038	-1.801111
H	1.123402	-3.171778	-0.464237
H	-0.633530	-3.201878	-0.272575
C	-1.090988	-0.472369	-0.694314
H	-1.959339	-0.913078	-0.186547
H	-1.227832	-0.680570	-1.763053
C	-1.099302	1.046119	-0.494690
H	-1.044688	1.298059	0.571108
H	-0.201530	1.481411	-0.957481
C	-2.328800	1.685240	-1.101023
H	-2.372519	1.580425	-2.184761
C	-2.898496	2.970447	-0.552162
H	-3.251242	3.723539	-1.251141
H	-2.438521	3.379492	0.344899
C	-3.679741	1.687215	-0.416085
C	-4.899294	1.509211	-1.298683
H	-4.753245	1.967062	-2.282817
H	-5.120008	0.446021	-1.451818
H	-5.781131	1.973227	-0.842157
C	-3.844426	1.082218	0.964763
H	-4.046082	0.005669	0.902656
H	-2.962594	1.227473	1.596699
H	-4.692391	1.546632	1.480287

Conformer 20

N	2.653193	2.563021	1.216614
N	1.771492	1.098161	2.551210
C	1.513840	1.976812	1.585119
H	0.540845	2.173851	1.162023

C	3.124473	1.115079	2.804836
H	3.572082	0.479107	3.552363
C	3.677762	2.036303	1.972368
H	4.699381	2.363923	1.857466
C	2.795360	3.598321	0.193111
H	3.148372	4.521323	0.656371
H	1.824338	3.772905	-0.272623
H	3.505595	3.261342	-0.563259
C	0.798026	0.170915	3.154801
H	0.980455	0.173205	4.231655
H	-0.192040	0.597793	2.987505
C	0.929029	-1.236226	2.571081
H	0.155872	-1.849661	3.049818
H	1.892674	-1.657905	2.883259
C	0.798536	-1.335279	1.042683
H	1.580810	-0.703545	0.591076
C	1.072014	-2.778814	0.608082
H	1.105912	-2.869737	-0.480441
H	2.031874	-3.134309	0.997290
H	0.288885	-3.450856	0.978128
C	-0.566121	-0.835227	0.546609
H	-0.764762	0.176095	0.933474
H	-1.359387	-1.468787	0.966476
C	-0.716408	-0.786923	-0.976505
H	0.127373	-0.231817	-1.413136
H	-0.654957	-1.801844	-1.379840
C	-2.025166	-0.146533	-1.383580
H	-2.122308	0.886161	-1.043218
C	-3.309850	-0.945699	-1.373315
H	-4.212543	-0.484731	-0.981721

H	-3.230123	-2.011544	-1.168413
C	-2.746809	-0.444010	-2.678967
C	-3.457285	0.718767	-3.345989
H	-3.854963	1.424943	-2.608866
H	-2.775619	1.267563	-4.007036
H	-4.297420	0.362645	-3.952722
C	-2.179441	-1.446694	-3.665673
H	-1.399146	-0.989462	-4.285627
H	-1.752651	-2.327990	-3.177771
H	-2.971197	-1.800643	-4.334719

Conformer 21

N	-1.333155	1.371474	4.125310
N	-0.703024	1.045190	2.075683
C	-1.253361	1.921620	2.912956
H	-1.579358	2.916914	2.651948
C	-0.428293	-0.109635	2.772997
H	0.024679	-0.964071	2.296097
C	-0.820185	0.094031	4.058089
H	-0.774621	-0.547135	4.923677
C	-1.862887	2.015860	5.327440
H	-2.687804	1.419565	5.720664
H	-2.226338	3.010103	5.066378
H	-1.071349	2.101934	6.074265
C	-0.512702	1.238706	0.627693
H	-0.291822	2.296880	0.467821
H	0.378443	0.668945	0.355837
C	-1.741958	0.783280	-0.158776
H	-2.607559	1.379495	0.157107

H	-1.961867	-0.257145	0.107497
C	-1.566588	0.909364	-1.684428
H	-2.464575	0.453748	-2.121389
C	-1.522039	2.373430	-2.132881
H	-1.516611	2.438949	-3.224645
H	-2.391862	2.932324	-1.771202
H	-0.616698	2.882072	-1.779224
C	-0.347555	0.137494	-2.212020
H	0.579802	0.622972	-1.875567
H	-0.336140	0.226833	-3.305083
C	-0.304122	-1.347249	-1.841468
H	-0.319001	-1.473467	-0.750687
H	-1.210512	-1.845697	-2.210094
C	0.917877	-2.031289	-2.409583
H	0.946233	-2.017444	-3.498047
C	1.507245	-3.261520	-1.767010
H	1.856108	-4.068689	-2.404394
H	1.065513	-3.598232	-0.831673
C	2.280003	-1.965420	-1.749989
C	3.485861	-1.844465	-2.660582
H	3.326573	-2.375721	-3.603907
H	3.693955	-0.794676	-2.898818
H	4.379475	-2.266606	-2.186922
C	2.458964	-1.254350	-0.422279
H	2.593328	-0.172914	-0.565209
H	1.607478	-1.414549	0.251126
H	3.350449	-1.630907	0.093291

Conformer 22

N	-1.589014	-0.798139	2.183446
N	0.296547	-1.757577	1.704160
C	-0.268616	-0.741338	2.353235
H	0.257152	0.001842	2.932448
C	-0.697227	-2.484781	1.090614
H	-0.479945	-3.359168	0.498927
C	-1.879513	-1.882855	1.387222
H	-2.890939	-2.130273	1.106706
C	-2.570946	0.115968	2.765315
H	-3.235016	0.471506	1.976901
H	-2.049147	0.966808	3.204017
H	-3.140903	-0.404862	3.537163
C	1.741720	-2.060177	1.687397
H	2.072377	-2.068893	2.728837
H	1.831344	-3.079909	1.310325
C	2.587499	-1.087604	0.867763
H	3.623693	-1.381320	1.073757
H	2.480175	-0.075056	1.275100
C	2.380205	-1.076732	-0.660054
H	3.266742	-0.565397	-1.057619
C	2.373527	-2.488297	-1.253323
H	2.444447	-2.444388	-2.343621
H	3.216199	-3.087288	-0.890467
H	1.447098	-3.028455	-1.019511
C	1.169454	-0.263255	-1.145656
H	0.225074	-0.720613	-0.818996
H	1.156992	-0.326463	-2.241078
C	1.190944	1.218560	-0.749811
H	0.910683	1.342089	0.305288
H	2.216282	1.604654	-0.830838

C	0.293448	2.057620	-1.633527
H	0.620176	2.060049	-2.672969
C	-0.323860	3.347294	-1.154678
H	-0.343606	4.197224	-1.830087
H	-0.177926	3.616365	-0.110806
C	-1.205708	2.160959	-1.458428
C	-2.055542	2.201011	-2.712438
H	-1.539237	2.718613	-3.526778
H	-2.293668	1.187951	-3.058251
H	-2.999771	2.726725	-2.529941
C	-1.879476	1.461870	-0.296519
H	-2.096263	0.411966	-0.540583
H	-1.256335	1.486058	0.604929
H	-2.829119	1.957710	-0.057570

Conformer 23

N	1.819471	-2.310340	0.403746
N	-0.236184	-2.981687	0.236483
C	0.685178	-2.511834	1.074024
H	0.531736	-2.305912	2.122031
C	0.327510	-3.088266	-1.013630
H	-0.230963	-3.445870	-1.863955
C	1.616367	-2.669606	-0.909969
H	2.399110	-2.597627	-1.648374
C	3.050074	-1.738758	0.949616
H	3.896551	-2.356886	0.649053
H	2.984434	-1.724093	2.037693
H	3.170393	-0.720964	0.573647
C	-1.659984	-3.188378	0.555995

H	-1.908135	-4.214076	0.272338
H	-1.751102	-3.113426	1.641772
C	-2.581089	-2.192903	-0.153552
H	-3.597762	-2.499948	0.119422
H	-2.503436	-2.339411	-1.238164
C	-2.402919	-0.697344	0.183286
H	-3.283136	-0.202727	-0.248205
C	-2.434018	-0.447615	1.694858
H	-2.567855	0.613271	1.919242
H	-3.258185	-0.987101	2.174452
H	-1.498512	-0.759729	2.178857
C	-1.169321	-0.076255	-0.492709
H	-1.161806	-0.360385	-1.554151
H	-0.248101	-0.484043	-0.058845
C	-1.057337	1.445934	-0.392117
H	-1.928607	1.921670	-0.859167
H	-1.064157	1.746519	0.660732
C	0.216415	1.922156	-1.057968
H	0.229772	1.739803	-2.132545
C	1.551914	1.742030	-0.365648
H	2.409362	1.405141	-0.944680
H	1.534568	1.389530	0.665768
C	1.010074	3.129773	-0.622466
C	1.671002	3.944204	-1.717777
H	1.983510	3.313987	-2.557499
H	0.982107	4.702436	-2.106650
H	2.558719	4.460728	-1.335961
C	0.560651	3.964415	0.560881
H	-0.217698	4.675567	0.261855
H	0.166502	3.357876	1.382006

H	1.404886	4.540143	0.956274

Conformer 24

N	-0.367780	1.980013	1.869354
N	-0.544299	-0.078531	2.530673
C	-1.218609	0.977753	2.084628
H	-2.282493	1.010402	1.906333
C	0.788134	0.259559	2.607155
H	1.537091	-0.438872	2.945208
C	0.900845	1.548247	2.190712
H	1.762317	2.191335	2.102726
C	-0.710739	3.299531	1.340461
H	-0.322614	4.068126	2.009735
H	-1.795448	3.386875	1.280638
H	-0.278190	3.411284	0.344535
C	-1.107266	-1.422151	2.764047
H	-0.851788	-1.699954	3.789540
H	-2.193410	-1.321522	2.709235
C	-0.584341	-2.467566	1.775780
H	-0.942106	-3.433133	2.152594
H	0.510307	-2.504200	1.845720
C	-1.000148	-2.321899	0.298311
H	-0.537910	-3.179846	-0.208029
C	-2.515633	-2.439955	0.112370
H	-2.780431	-2.443968	-0.948778
H	-2.905549	-3.363356	0.553006
H	-3.048963	-1.596137	0.570492
C	-0.441861	-1.048872	-0.360531
H	0.529596	-0.794757	0.080504

H	-1.111847	-0.202888	-0.149285
C	-0.250132	-1.118529	-1.876680
H	0.504657	-1.877235	-2.115358
H	-1.174072	-1.442447	-2.371492
C	0.154183	0.236634	-2.416754
H	-0.656521	0.965462	-2.372793
C	1.093120	0.402078	-3.583078
H	0.872467	1.165613	-4.323472
H	1.542796	-0.497811	-3.995522
C	1.533503	0.831328	-2.205357
C	1.610071	2.316631	-1.914233
H	0.804972	2.870086	-2.410457
H	1.537908	2.499955	-0.831183
H	2.562870	2.739539	-2.252275
C	2.624239	0.033448	-1.517728
H	2.599760	0.184625	-0.429420
H	2.543274	-1.040467	-1.709919
H	3.610037	0.355571	-1.871413

Conformer 25

N	1.094107	1.567425	3.697052
N	1.935278	1.100730	1.752683
C	0.910340	1.673121	2.380160
H	0.067777	2.144543	1.897098
C	2.800701	0.601692	2.698887
H	3.707893	0.086657	2.425274
C	2.276710	0.896960	3.918206
H	2.642351	0.694543	4.913604
C	0.200648	2.083250	4.733987

H	-0.157127	1.256237	5.348466
H	-0.646684	2.576065	4.259081
H	0.739933	2.802801	5.350220
C	2.060490	0.939258	0.294019
H	1.444496	1.717196	-0.165824
H	3.103032	1.144353	0.039668
C	1.603494	-0.446785	-0.153760
H	0.623273	-0.636197	0.296190
H	2.291068	-1.212113	0.233942
C	1.503909	-0.555898	-1.682483
H	0.939120	0.316701	-2.038879
C	2.885285	-0.539943	-2.343528
H	2.791524	-0.613544	-3.431039
H	3.443476	0.379240	-2.132000
H	3.490576	-1.389499	-2.005150
C	0.718718	-1.809533	-2.097461
H	1.262411	-2.706012	-1.769394
H	0.708828	-1.844604	-3.194152
C	-0.730303	-1.878945	-1.592806
H	-1.206052	-2.757058	-2.045935
H	-0.747528	-2.061683	-0.511429
C	-1.536400	-0.640244	-1.930823
H	-1.405304	-0.318081	-2.964127
C	-1.742971	0.488850	-0.938627
H	-1.691402	1.516919	-1.289778
H	-1.375863	0.340822	0.076943
C	-2.910572	-0.366796	-1.371691
C	-3.927229	0.256466	-2.308095
H	-3.454270	0.945590	-3.015374
H	-4.443964	-0.516563	-2.887825

H	-4.683659	0.816518	-1.746926
C	-3.519563	-1.327192	-0.368636
H	-3.976443	-2.182822	-0.879115
H	-2.784021	-1.716476	0.341930
H	-4.306081	-0.825951	0.207449

Conformer 26

N	1.439345	-2.147157	3.815746
N	1.322258	-1.602704	1.719615
C	1.311193	-2.621620	2.575781
H	1.214241	-3.662632	2.308875
C	1.454970	-0.434069	2.434787
H	1.487918	0.528041	1.947616
C	1.531984	-0.774074	3.748519
H	1.648228	-0.165645	4.632053
C	1.484431	-2.945392	5.040551
H	2.444327	-2.794622	5.537578
H	1.369819	-3.997491	4.782930
H	0.668828	-2.643082	5.698229
C	1.130318	-1.693950	0.262027
H	1.844774	-1.003832	-0.193357
H	1.408565	-2.707274	-0.033616
C	-0.298075	-1.341135	-0.138652
H	-0.543520	-0.371287	0.305007
H	-0.994843	-2.076043	0.285974
C	-0.465814	-1.272038	-1.664147
H	0.365122	-0.671922	-2.060442
C	-0.403691	-2.662611	-2.302714
H	-0.519455	-2.592073	-3.388833

H	0.549442	-3.171762	-2.115938
H	-1.210513	-3.302792	-1.924443
C	-1.770250	-0.557884	-2.050155
H	-2.627303	-1.136901	-1.679425
H	-1.843066	-0.574719	-3.145106
C	-1.893347	0.896960	-1.574385
H	-2.803146	1.322670	-2.015270
H	-2.050413	0.930113	-0.488926
C	-0.698261	1.747164	-1.958328
H	-0.375727	1.588040	-2.988050
C	0.422964	2.046784	-0.981659
H	1.452500	2.027254	-1.332030
H	0.291225	1.716582	0.049097
C	-0.486835	3.154493	-1.457586
C	0.087657	4.158923	-2.437140
H	0.794161	3.688308	-3.129385
H	-0.709510	4.618778	-3.032563
H	0.617443	4.960447	-1.908752
C	-1.470909	3.759187	-0.475511
H	-2.346492	4.156252	-0.999883
H	-1.825597	3.036068	0.264201
H	-1.004947	4.588649	0.067708

Conformer 27

N	3.210259	0.393799	1.943545
N	1.788001	-1.217200	2.240297
C	2.218420	-0.050275	2.716157
H	1.829274	0.452715	3.588361
C	2.523204	-1.525644	1.118673

H	2.344012	-2.425136	0.551472
C	3.417999	-0.519475	0.933726
H	4.177140	-0.376261	0.181159
C	3.970519	1.627027	2.144691
H	3.918303	2.230562	1.238697
H	5.007808	1.382895	2.374386
H	3.534289	2.183100	2.973629
C	0.636768	-1.978724	2.760922
H	0.765328	-3.004615	2.411919
H	0.718745	-1.989394	3.850369
C	-0.691859	-1.375296	2.306178
H	-0.801649	-0.384421	2.766537
H	-1.486765	-2.000966	2.730801
C	-0.873255	-1.255947	0.786347
H	-0.021790	-0.686571	0.385392
C	-0.909494	-2.625134	0.102629
H	-0.970279	-2.521516	-0.984488
H	-0.019549	-3.229928	0.314494
H	-1.782576	-3.201207	0.431013
C	-2.136303	-0.433357	0.490427
H	-2.053606	0.525546	1.019894
H	-3.008657	-0.950214	0.913243
C	-2.388926	-0.141213	-0.994227
H	-2.665508	-1.061255	-1.523267
H	-3.265645	0.510683	-1.057998
C	-1.202353	0.497579	-1.686192
H	-0.384428	-0.198362	-1.876920
C	-0.763607	1.907066	-1.347368
H	0.298463	2.119300	-1.245366
H	-1.398200	2.482321	-0.676195

C	-1.309570	1.595756	-2.719548
C	-0.324562	1.543415	-3.871376
H	0.643672	1.137843	-3.555838
H	-0.705576	0.910580	-4.681241
H	-0.150950	2.544766	-4.281675
C	-2.661094	2.160813	-3.110191
H	-3.167196	1.505759	-3.828729
H	-3.324959	2.299393	-2.251674
H	-2.533848	3.141192	-3.582669

Conformer 28

N	4.262440	0.209836	0.745371
N	2.800083	-1.367109	1.029287
C	3.219780	-0.185116	1.476645
H	2.786360	0.363076	2.299539
C	3.595620	-1.737207	-0.030866
H	3.439118	-2.661697	-0.564164
C	4.515080	-0.751689	-0.208127
H	5.319672	-0.653060	-0.919997
C	5.019722	1.448007	0.928933
H	6.057851	1.206886	1.162842
H	4.583128	2.011815	1.752712
H	4.968267	2.041490	0.015259
C	1.614255	-2.091504	1.525122
H	1.727545	-3.126055	1.197136
H	1.661338	-2.085327	2.616713
C	0.317735	-1.461011	1.018982
H	0.237053	-0.454412	1.446116
H	-0.508876	-2.042104	1.446699

C	0.168142	-1.405015	-0.509178
H	1.045546	-0.885160	-0.925977
C	0.101334	-2.807777	-1.120502
H	0.004095	-2.749178	-2.208475
H	0.992240	-3.412018	-0.909123
H	-0.766837	-3.356926	-0.738054
C	-1.070530	-0.586065	-0.904501
H	-1.960692	-1.018201	-0.427508
H	-1.223082	-0.696579	-1.985459
C	-0.993117	0.908588	-0.579296
H	-0.921416	1.066715	0.503612
H	-0.072954	1.329493	-1.011311
C	-2.184740	1.667344	-1.119700
H	-2.238286	1.656949	-2.208192
C	-2.674677	2.932574	-0.458955
H	-2.982496	3.763414	-1.087513
H	-2.189030	3.233757	0.466985
C	-3.531257	1.691067	-0.426908
C	-4.762253	1.662122	-1.311389
H	-4.591401	2.190998	-2.254915
H	-5.047792	0.630756	-1.550821
H	-5.612621	2.138810	-0.810313
C	-3.727920	0.981859	0.899080
H	-3.996654	-0.070937	0.747078
H	-2.836140	1.016560	1.533516
H	-4.543632	1.452029	1.459619

Conformer 29

N	4.360582	0.422295	1.268121

N	2.671864	0.027602	2.569851
C	3.512049	-0.515054	1.691892
H	3.503464	-1.545667	1.371544
C	2.987433	1.360928	2.708153
H	2.436945	2.010855	3.369845
C	4.047217	1.609110	1.894961
H	4.604368	2.514556	1.713456
C	5.449046	0.226291	0.310395
H	5.328459	0.925396	-0.518025
H	5.408686	-0.794523	-0.068986
H	6.405709	0.393351	0.807548
C	1.525664	-0.651328	3.200558
H	1.711277	-1.724223	3.123834
H	1.543083	-0.388206	4.260601
C	0.205408	-0.246371	2.546729
H	0.030642	0.820159	2.736769
H	-0.588777	-0.786024	3.077495
C	0.099676	-0.523034	1.039124
H	0.913629	0.019013	0.530402
C	0.235120	-2.015383	0.721609
H	0.192584	-2.196957	-0.354952
H	1.180421	-2.444429	1.076643
H	-0.576931	-2.584097	1.188953
C	-1.224557	0.053697	0.514132
H	-1.309436	1.097043	0.847857
H	-2.063334	-0.484203	0.977500
C	-1.386530	0.015995	-1.007946
H	-0.522848	0.504809	-1.482496
H	-1.386573	-1.022583	-1.355075
C	-2.665880	0.699034	-1.438130

H	-2.719603	1.741205	-1.122052
C	-3.980990	-0.047764	-1.418023
H	-4.867327	0.458876	-1.046590
H	-3.945724	-1.110275	-1.184490
C	-3.388463	0.398473	-2.731585
C	-4.045392	1.571747	-3.432819
H	-4.422302	2.309232	-2.716064
H	-3.334908	2.078888	-4.096308
H	-4.892052	1.235145	-4.041934
C	-2.855584	-0.649965	-3.688718
H	-2.055773	-0.238553	-4.315964
H	-2.463932	-1.532603	-3.174239
H	-3.656409	-0.992457	-4.353576

Conformer 30

N	4.027656	2.282187	1.137014
N	2.065712	1.480354	1.602192
C	3.362694	1.413654	1.899270
H	3.803062	0.762992	2.639634
C	1.895306	2.420821	0.611398
H	0.924051	2.647802	0.202096
C	3.123731	2.925469	0.319367
H	3.429630	3.680014	-0.388304
C	5.469976	2.524749	1.170307
H	5.657748	3.553413	1.480034
H	5.887638	2.348577	0.178887
H	5.928588	1.840694	1.883426
C	1.018809	0.620221	2.188414
H	1.157682	0.633342	3.272018

H	0.065119	1.104294	1.970941
C	1.074530	-0.801082	1.633518
H	0.304412	-1.376012	2.160377
H	2.034002	-1.256245	1.913202
C	0.873456	-0.918823	0.115162
H	1.684962	-0.360839	-0.377392
C	1.017838	-2.384870	-0.307162
H	0.882384	-2.491633	-1.387758
H	2.009294	-2.774200	-0.052002
H	0.276612	-3.021550	0.187368
C	-0.454442	-0.311860	-0.373953
H	-0.523547	-0.478713	-1.455187
H	-0.442501	0.782360	-0.253618
C	-1.723675	-0.857548	0.289182
H	-1.730768	-1.951497	0.235070
H	-1.735738	-0.613334	1.361413
C	-2.977628	-0.299535	-0.346254
H	-3.089731	0.776125	-0.206067
C	-4.264646	-1.087698	-0.354068
H	-5.198090	-0.565141	-0.164286
H	-4.232128	-2.097696	0.048335
C	-3.540305	-0.826080	-1.650690
C	-4.142459	0.192289	-2.598733
H	-4.617768	1.016098	-2.055382
H	-3.374876	0.619282	-3.255808
H	-4.905644	-0.271967	-3.233747
C	-2.878852	-1.991006	-2.361673
H	-2.076845	-1.644867	-3.025177
H	-2.453331	-2.723814	-1.669481
H	-3.611918	-2.518726	-2.981882

Conformer 31

N	0.169827	-2.032812	4.572429
N	-0.371976	-1.558179	2.526780
C	0.064246	-2.520757	3.335365
H	0.293517	-3.533022	3.038109
C	-0.546909	-0.411477	3.268639
H	-0.895287	0.506870	2.822127
C	-0.211197	-0.708574	4.551363
H	-0.214677	-0.101762	5.443158
C	0.587531	-2.780189	5.758852
H	1.416590	-2.259080	6.240517
H	0.913813	-3.774845	5.455406
H	-0.253977	-2.866185	6.448851
C	-0.572062	-1.668247	1.071355
H	-0.646655	-2.732407	0.837472
H	-1.539978	-1.210746	0.854772
C	0.561141	-0.991549	0.303238
H	1.501801	-1.506674	0.533801
H	0.666946	0.036965	0.668079
C	0.341090	-0.978819	-1.220684
H	1.184738	-0.411348	-1.632548
C	0.399110	-2.387130	-1.819584
H	0.367817	-2.337978	-2.912018
H	1.320676	-2.908029	-1.537139
H	-0.453077	-3.002800	-1.505047
C	-0.951586	-0.258514	-1.640291
H	-1.822902	-0.878203	-1.381923
H	-0.953654	-0.193086	-2.734224

C	-1.138534	1.152338	-1.068321
H	-2.075062	1.563212	-1.460319
H	-1.281965	1.110156	0.023546
C	0.010739	2.088218	-1.375606
H	0.937528	1.832397	-0.860334
C	-0.211198	3.569199	-1.571247
H	0.502559	4.265432	-1.139370
H	-1.239112	3.925101	-1.563679
C	0.223198	2.717636	-2.737493
C	1.655795	2.851771	-3.214840
H	2.341413	3.046958	-2.383019
H	1.987008	1.935421	-3.718430
H	1.753388	3.678310	-3.927710
C	-0.766344	2.475031	-3.860580
H	-0.510338	1.570209	-4.425034
H	-1.794476	2.371392	-3.501834
H	-0.749504	3.315796	-4.562585

Conformer 32

N	-4.318554	-0.798581	0.911909
N	-2.355468	-1.063006	0.030390
C	-3.257311	-1.603515	0.846900
H	-3.145604	-2.539373	1.373159
C	-2.852739	0.130365	-0.439142
H	-2.287998	0.753443	-1.114350
C	-4.084682	0.297732	0.111057
H	-4.809295	1.089075	-0.000731
C	-5.539285	-1.050217	1.677829
H	-6.372467	-1.216399	0.992768

H	-5.744236	-0.190641	2.317458
H	-5.393132	-1.934669	2.297741
C	-1.007300	-1.600026	-0.229049
H	-1.115954	-2.670500	-0.420699
H	-0.666918	-1.126366	-1.152170
C	-0.024227	-1.325136	0.906389
H	0.924703	-1.755497	0.569653
H	-0.315844	-1.894327	1.798823
C	0.152727	0.165770	1.259807
H	-0.085583	0.759009	0.365048
C	-0.786034	0.598110	2.393098
H	-0.682598	1.668806	2.594226
H	-1.842541	0.410707	2.167541
H	-0.543127	0.062276	3.318202
C	1.606354	0.492029	1.640694
H	1.895056	-0.111707	2.512047
H	1.637554	1.538019	1.971652
C	2.641293	0.304234	0.523349
H	3.610321	0.657627	0.896314
H	2.781196	-0.761883	0.307332
C	2.281886	1.049850	-0.745947
H	1.931307	2.066127	-0.561529
C	1.625210	0.349790	-1.920643
H	0.831304	0.853866	-2.468533
H	1.516942	-0.732866	-1.863566
C	3.033473	0.881807	-2.043025
C	3.243897	2.122656	-2.888001
H	3.355917	1.858200	-3.945702
H	2.401139	2.817682	-2.802474
H	4.150091	2.654626	-2.576593

C	4.176092	-0.111264	-2.129491
H	5.103353	0.329336	-1.746256
H	3.981198	-1.026583	-1.563343
H	4.349885	-0.398584	-3.172142

Conformer 33

N	3.804398	2.247736	0.338704
N	1.984375	1.243272	0.957528
C	3.311694	1.246353	1.068424
H	3.894942	0.552464	1.654743
C	1.615593	2.275734	0.125159
H	0.582111	2.471461	-0.115003
C	2.754862	2.907651	-0.262343
H	2.909381	3.758342	-0.907500
C	5.218861	2.591578	0.196653
H	5.522111	2.461216	-0.842979
H	5.810056	1.935156	0.834243
H	5.370119	3.626792	0.504681
C	1.073634	0.275991	1.601390
H	1.190428	0.382699	2.682850
H	0.063629	0.599471	1.342793
C	1.330415	-1.160173	1.152425
H	0.585459	-1.778824	1.666446
H	2.303088	-1.497070	1.535159
C	1.268574	-1.405198	-0.365165
H	2.131548	-0.894839	-0.819985
C	1.423535	-2.902957	-0.643600
H	1.423880	-3.100190	-1.720025
H	2.361488	-3.290693	-0.231312

H	0.603386	-3.476099	-0.197714
C	0.011116	-0.818280	-1.027929
H	0.058585	-1.034403	-2.102469
H	0.031286	0.278148	-0.957602
C	-1.334766	-1.303470	-0.478898
H	-1.485004	-2.361776	-0.723713
H	-1.342768	-1.241799	0.616290
C	-2.472818	-0.478263	-1.037822
H	-2.540924	-0.524193	-2.124608
C	-2.816799	0.865638	-0.431453
H	-3.050982	1.699610	-1.087907
H	-2.304940	1.153134	0.487543
C	-3.793362	-0.283930	-0.334601
C	-5.032069	-0.225485	-1.207157
H	-4.818106	0.227653	-2.181031
H	-5.429379	-1.231463	-1.384643
H	-5.818917	0.366788	-0.726307
C	-4.041705	-0.917217	1.020926
H	-4.288231	-1.980511	0.917157
H	-3.180952	-0.834655	1.692875
H	-4.886832	-0.425400	1.515776

Conformer 34

N	-1.163765	-0.204575	4.568180
N	-1.102257	-1.222951	2.654006
C	-1.355791	-0.062988	3.255906
H	-1.664336	0.844295	2.758731
C	-0.727773	-2.137677	3.611992
H	-0.469079	-3.153573	3.358353

C	-0.770871	-1.502363	4.812696
H	-0.563799	-1.857217	5.809722
C	-1.361919	0.831076	5.582374
H	-2.203406	0.557629	6.221727
H	-1.572603	1.778423	5.086018
H	-0.452848	0.929186	6.177912
C	-1.120927	-1.449834	1.198835
H	-1.475200	-2.471063	1.043213
H	-1.868988	-0.771932	0.781815
C	0.258026	-1.215398	0.585315
H	0.979715	-1.892009	1.059986
H	0.574254	-0.193870	0.828945
C	0.291041	-1.425562	-0.939528
H	1.299112	-1.128408	-1.254560
C	0.105611	-2.898295	-1.316939
H	0.251174	-3.037086	-2.392252
H	0.825862	-3.540963	-0.798950
H	-0.904174	-3.258751	-1.084087
C	-0.711362	-0.539292	-1.696656
H	-1.735181	-0.899153	-1.515882
H	-0.536535	-0.684731	-2.768624
C	-0.634935	0.961814	-1.389532
H	-1.363111	1.480500	-2.022258
H	-0.961594	1.162365	-0.356142
C	0.743052	1.556961	-1.586549
H	1.476476	1.225739	-0.850314
C	0.920754	2.981652	-2.055809
H	1.695386	3.589528	-1.596362
H	0.024201	3.535012	-2.326244
C	1.334055	1.842158	-2.952222

C	2.818277	1.584761	-3.123782
H	3.374361	1.801412	-2.205148
H	3.005422	0.538377	-3.393574
H	3.232710	2.213894	-3.919437
C	0.528421	1.581749	-4.210115
H	0.657553	0.548867	-4.554141
H	-0.541648	1.760970	-4.070113
H	0.866637	2.241321	-5.016506

Conformer 35

N	0.561069	1.247030	4.830921
N	-0.122130	0.816155	2.817620
C	0.944152	1.099745	3.561841
H	1.954896	1.197997	3.195772
C	-1.226132	0.772370	3.638724
H	-2.211147	0.555010	3.256423
C	-0.800067	1.046319	4.899928
H	-1.342262	1.119097	5.830079
C	1.430070	1.581235	5.959527
H	1.126980	2.542991	6.376476
H	2.459650	1.644914	5.607953
H	1.353559	0.800917	6.718789
C	-0.115774	0.518773	1.374765
H	-0.947336	1.072831	0.933877
H	0.808403	0.933452	0.964277
C	-0.212517	-0.979990	1.101355
H	-1.093835	-1.395763	1.608078
H	0.667074	-1.469802	1.537082
C	-0.292895	-1.276402	-0.405708

H	0.430899	-0.623766	-0.912722
C	0.106179	-2.729253	-0.673179
H	0.023696	-2.971291	-1.735354
H	1.140585	-2.922485	-0.370635
H	-0.543962	-3.418096	-0.121536
C	-1.691461	-0.968467	-0.968231
H	-2.097458	-0.056620	-0.505659
H	-2.372086	-1.780889	-0.680172
C	-1.733915	-0.768761	-2.488465
H	-1.396474	-1.676031	-3.001304
H	-2.779425	-0.628662	-2.788425
C	-0.929517	0.433702	-2.936798
H	-1.157164	1.328417	-2.352766
C	-0.638669	0.702240	-4.391958
H	-0.747599	1.716782	-4.765437
H	-0.923994	-0.066225	-5.106509
C	0.498118	0.386200	-3.454757
C	1.412285	1.521724	-3.039262
H	0.864980	2.465799	-2.944514
H	1.888988	1.307343	-2.072276
H	2.211683	1.671900	-3.773861
C	1.222582	-0.932274	-3.639260
H	1.724276	-1.251664	-2.716750
H	0.553471	-1.737707	-3.955411
H	1.992226	-0.828795	-4.412538

Conformer 36

N	-1.677667	-3.937907	1.093941
N	-1.299178	-1.954118	1.884263

C	-1.928242	-2.637986	0.932434
H	-2.534325	-2.208087	0.149846
C	-0.620787	-2.844702	2.684471
H	-0.029276	-2.521698	3.526027
C	-0.859513	-4.088692	2.192468
H	-0.524979	-5.057985	2.526028
C	-2.203872	-5.023788	0.267061
H	-1.377517	-5.658983	-0.055239
H	-2.693111	-4.597769	-0.609008
H	-2.924468	-5.606766	0.843566
C	-1.268617	-0.486080	2.011182
H	-1.990180	-0.095990	1.290568
H	-1.636144	-0.246689	3.012085
C	0.119452	0.111144	1.787502
H	0.804740	-0.262411	2.558705
H	0.003977	1.182390	1.988339
C	0.755263	-0.080007	0.396058
H	1.607626	0.610355	0.377730
C	1.334689	-1.483016	0.170702
H	1.960568	-1.489685	-0.726530
H	1.960126	-1.801757	1.011291
H	0.557327	-2.241256	0.018478
C	-0.172680	0.321572	-0.759656
H	-1.009142	-0.392610	-0.832210
H	0.388571	0.205438	-1.693656
C	-0.727273	1.751648	-0.696359
H	-1.317233	1.932712	-1.601192
H	-1.437580	1.857766	0.136761
C	0.343965	2.812138	-0.553970
H	0.813402	2.835394	0.429346

C	0.195098	4.165902	-1.205366
H	0.492799	5.048391	-0.646347
H	-0.669783	4.317603	-1.846849
C	1.264839	3.219790	-1.687079
C	2.702025	3.537078	-1.323561
H	2.774706	3.998139	-0.332705
H	3.315767	2.627649	-1.319395
H	3.141421	4.232865	-2.047203
C	1.113193	2.603118	-3.064293
H	1.710998	1.686885	-3.154246
H	0.073400	2.357075	-3.303677
H	1.467616	3.302929	-3.830313

Conformer 37

N	1.238652	0.301845	4.932136
N	0.794990	-0.694967	3.057647
C	1.407766	0.349558	3.609972
H	1.953789	1.111304	3.073740
C	0.207944	-1.434669	4.059427
H	-0.349916	-2.333989	3.849783
C	0.485173	-0.811723	5.234798
H	0.221269	-1.064902	6.249785
C	1.771457	1.258595	5.902559
H	0.947868	1.690602	6.472531
H	2.296706	2.050528	5.369218
H	2.465512	0.748421	6.572061
C	0.676730	-0.962384	1.613235
H	1.530057	-0.485009	1.123690
H	0.782377	-2.041450	1.486702

C	-0.645361	-0.440810	1.055403
H	-0.724705	0.627697	1.292258
H	-1.478354	-0.944595	1.564663
C	-0.773048	-0.639469	-0.463150
H	0.060193	-0.106440	-0.941859
C	-0.696817	-2.121278	-0.846591
H	-0.913980	-2.262251	-1.907627
H	0.295647	-2.551951	-0.672955
H	-1.430536	-2.707067	-0.278362
C	-2.081758	0.001607	-0.952652
H	-2.216264	0.969742	-0.450298
H	-2.926147	-0.627730	-0.640345
C	-2.151086	0.244154	-2.466111
H	-2.080863	-0.702795	-3.013198
H	-3.144728	0.646515	-2.698276
C	-1.103139	1.225166	-2.949726
H	-1.064583	2.128083	-2.337777
C	-0.851346	1.459594	-4.418289
H	-0.734141	2.481198	-4.768629
H	-1.360573	0.805402	-5.121953
C	0.232295	0.848120	-3.568288
C	1.422805	1.712692	-3.203243
H	1.132188	2.758451	-3.057953
H	1.890758	1.360200	-2.273846
H	2.185598	1.683560	-3.989548
C	0.600802	-0.599902	-3.819956
H	1.064978	-1.056116	-2.935981
H	-0.262283	-1.209782	-4.102361
H	1.324073	-0.663758	-4.640635

Conformer 38

N	-1.612783	-1.133082	4.511129
N	-1.984357	-1.075850	2.374858
C	-1.036598	-1.126756	3.308212
H	0.026481	-1.157967	3.119061
C	-3.210376	-1.037395	3.000684
H	-4.133753	-0.990648	2.443588
C	-2.979291	-1.077177	4.339584
H	-3.661323	-1.076121	5.176282
C	-0.917359	-1.200066	5.796570
H	-1.185429	-0.330548	6.398296
H	0.157743	-1.199223	5.618790
H	-1.199492	-2.117432	6.314951
C	-1.749985	-0.972793	0.924196
H	-0.780121	-1.434710	0.727338
H	-2.513193	-1.581217	0.431742
C	-1.791699	0.478119	0.453407
H	-1.015268	1.046390	0.981557
H	-2.754649	0.920095	0.738390
C	-1.601316	0.615810	-1.066106
H	-2.447251	0.107887	-1.551162
C	-1.668578	2.095591	-1.456851
H	-1.559026	2.212756	-2.539182
H	-2.626710	2.539573	-1.166284
H	-0.874133	2.676375	-0.975191
C	-0.314962	-0.057445	-1.580264
H	-0.233645	0.149288	-2.653340
H	-0.404622	-1.151004	-1.505269
C	0.987215	0.382448	-0.902439

H	1.068836	1.474792	-0.918515
H	0.983811	0.103478	0.163558
C	2.200430	-0.238810	-1.558463
H	2.228335	-1.325620	-1.470157
C	3.544055	0.449342	-1.533415
H	4.435752	-0.149380	-1.369116
H	3.587789	1.442070	-1.090447
C	2.804158	0.298129	-2.839588
C	3.331087	-0.722438	-3.829545
H	3.747912	-1.598075	-3.320502
H	2.532722	-1.068726	-4.496807
H	4.123040	-0.288801	-4.450661
C	2.230394	1.537116	-3.499467
H	1.401917	1.278825	-4.170970
H	1.863922	2.271870	-2.775743
H	3.000320	2.031329	-4.102705

Conformer 39

N	-3.584179	-1.185245	0.821514
N	-1.778308	-0.973182	2.003508
C	-2.526544	-1.830433	1.314112
H	-2.306779	-2.876734	1.167609
C	-2.377339	0.264484	1.953483
H	-1.952609	1.124614	2.446378
C	-3.508070	0.134244	1.211107
H	-4.260719	0.855599	0.934782
C	-4.638088	-1.769605	-0.007462
H	-5.606953	-1.591051	0.461281
H	-4.470063	-2.843456	-0.092406

H	-4.610151	-1.316408	-0.999668
C	-0.488533	-1.278322	2.648679
H	-0.578456	-0.973781	3.694285
H	-0.379338	-2.364658	2.629362
C	0.699867	-0.583684	1.984641
H	1.560247	-0.828510	2.618947
H	0.574443	0.502650	2.063188
C	1.034256	-0.982102	0.532787
H	2.038925	-0.580269	0.354300
C	1.124398	-2.500661	0.359409
H	1.552409	-2.750358	-0.615642
H	1.755921	-2.960830	1.127486
H	0.137004	-2.980248	0.402487
C	0.127707	-0.360211	-0.544349
H	-0.887352	-0.779542	-0.485920
H	0.520356	-0.682683	-1.515808
C	0.046048	1.172908	-0.544276
H	-0.531462	1.484949	-1.421795
H	-0.531796	1.527997	0.320574
C	1.395838	1.858327	-0.536605
H	1.921010	1.779506	0.415588
C	1.620057	3.158667	-1.270011
H	2.211254	3.932071	-0.787990
H	0.799641	3.539262	-1.874249
C	2.312556	1.905779	-1.742814
C	3.800393	1.782393	-1.478819
H	4.079919	2.245706	-0.526633
H	4.107334	0.729683	-1.446314
H	4.377782	2.273508	-2.270109
C	1.880786	1.296886	-3.062754

H	2.154219	0.235673	-3.115963
H	0.802591	1.380222	-3.232565
H	2.381779	1.806845	-3.893358

Conformer 40

N	-3.318285	-0.646615	2.288219
N	-1.215777	-0.430855	2.773993
C	-2.154996	-1.289989	2.388014
H	-1.998140	-2.337409	2.181565
C	-1.795546	0.807612	2.926484
H	-1.226147	1.671399	3.230089
C	-3.113248	0.674301	2.622446
H	-3.916049	1.394705	2.618964
C	-4.598299	-1.239516	1.901270
H	-5.291662	-1.191998	2.742729
H	-4.437049	-2.280411	1.619787
H	-5.002854	-0.693155	1.047951
C	0.218563	-0.735797	2.924901
H	0.498490	-0.455082	3.943212
H	0.317219	-1.820030	2.841385
C	1.095299	-0.015655	1.901526
H	2.124548	-0.284133	2.167995
H	1.018455	1.067728	2.056120
C	0.863811	-0.343705	0.413497
H	1.721364	0.102267	-0.104178
C	0.897325	-1.850838	0.147115
H	0.893495	-2.060695	-0.924832
H	1.795507	-2.315772	0.568712
H	0.022097	-2.360221	0.573003

C	-0.392003	0.319145	-0.182758
H	-0.517652	1.318783	0.257353
H	-1.290537	-0.255691	0.085893
C	-0.354178	0.476302	-1.709669
H	-0.266352	-0.505298	-2.188543
H	-1.320019	0.881263	-2.036585
C	0.746646	1.404156	-2.179630
H	0.774898	2.337726	-1.615872
C	1.082362	1.546558	-3.643483
H	1.245337	2.543295	-4.043151
H	0.591861	0.866210	-4.335888
C	2.102658	0.960153	-2.703608
C	3.294773	1.816388	-2.323857
H	3.022595	2.874099	-2.246273
H	3.710714	1.503505	-1.357653
H	4.090658	1.730362	-3.071869
C	2.443276	-0.508863	-2.853642
H	2.856422	-0.922196	-1.924211
H	1.578490	-1.114589	-3.142693
H	3.201929	-0.637446	-3.633830

Conformer 41

N	-1.398077	2.599377	4.361036
N	-1.188644	1.608414	2.442037
C	-1.510781	2.752515	3.041090
H	-1.817612	3.657054	2.538303
C	-0.840838	0.694462	3.410308
H	-0.534180	-0.310028	3.160797
C	-0.976260	1.312525	4.613993

H	-0.816131	0.949461	5.618072
C	-1.681513	3.619132	5.371553
H	-2.495432	3.278631	6.014023
H	-1.975571	4.542030	4.871304
H	-0.783719	3.798961	5.964677
C	-1.117129	1.385141	0.987834
H	-1.477988	0.369605	0.810801
H	-1.823570	2.074763	0.517672
C	0.300305	1.587861	0.460078
H	0.977667	0.889661	0.967978
H	0.634727	2.599528	0.723192
C	0.400138	1.399217	-1.062504
H	-0.238170	2.162082	-1.531155
C	1.839458	1.664975	-1.515584
H	1.926556	1.554455	-2.600573
H	2.157157	2.680420	-1.255000
H	2.544914	0.967021	-1.051102
C	-0.110372	0.026404	-1.539164
H	0.062836	-0.039772	-2.619165
H	-1.201782	-0.034124	-1.418965
C	0.531527	-1.192689	-0.868219
H	1.623170	-1.123459	-0.927791
H	0.294866	-1.210962	0.207533
C	0.058737	-2.489941	-1.486160
H	-1.009061	-2.666628	-1.351266
C	0.925787	-3.725936	-1.477559
H	0.464145	-4.690371	-1.283944
H	1.930071	-3.624588	-1.071358
C	0.623712	-3.032360	-2.782624
C	-0.354731	-3.704293	-3.726426

H	-1.148899	-4.223850	-3.179881
H	-0.826867	-2.969072	-4.389028
H	0.156095	-4.442677	-4.354818
C	1.747978	-2.308596	-3.498573
H	1.354683	-1.537268	-4.173096
H	2.451531	-1.830502	-2.809785
H	2.321266	-3.016580	-4.108542

Conformer 42

N	2.286577	3.552905	-0.222935
N	1.964932	1.571452	0.599208
C	1.361041	2.660146	0.131686
H	0.293586	2.796615	0.052103
C	3.325841	1.768831	0.537140
H	4.027052	1.017394	0.863918
C	3.528671	3.011300	0.025380
H	4.438453	3.553711	-0.177280
C	2.032467	4.887135	-0.764103
H	2.425250	5.636930	-0.076532
H	0.956892	5.025628	-0.877156
H	2.514347	4.981202	-1.738863
C	1.293213	0.333760	1.036617
H	1.668014	0.100697	2.035863
H	0.230079	0.559723	1.122624
C	1.537684	-0.817633	0.067401
H	1.010064	-1.683578	0.478696
H	2.604643	-1.076140	0.076827
C	1.091640	-0.571220	-1.379859
H	1.672381	0.279656	-1.769565

C	1.461714	-1.787222	-2.235652
H	1.191872	-1.624034	-3.283189
H	2.537420	-1.985970	-2.193335
H	0.946494	-2.691385	-1.894296
C	-0.395297	-0.195082	-1.542178
H	-0.565911	-0.066881	-2.617349
H	-0.584658	0.797176	-1.104562
C	-1.444754	-1.178944	-0.987616
H	-2.339777	-1.104869	-1.616688
H	-1.087401	-2.208381	-1.102607
C	-1.859145	-0.920438	0.451106
H	-1.998545	0.142031	0.665920
C	-1.383677	-1.761750	1.621062
H	-1.135301	-1.274052	2.561466
H	-0.776602	-2.637552	1.400453
C	-2.827789	-1.810670	1.194625
C	-3.848192	-1.131040	2.086408
H	-3.441152	-0.223976	2.547044
H	-4.738885	-0.847672	1.514047
H	-4.166389	-1.801334	2.892539
C	-3.344482	-3.072087	0.529884
H	-4.179534	-2.843758	-0.142026
H	-2.574419	-3.585542	-0.053016
H	-3.708416	-3.775084	1.286789

Conformer 43

N	-1.454533	-3.316361	2.318391
N	-0.666801	-1.340933	2.740264
C	-1.712562	-2.009120	2.262183

H	-2.618037	-1.563458	1.880338
C	0.295909	-2.248868	3.115733
H	1.242760	-1.939241	3.527143
C	-0.197034	-3.487814	2.853904
H	0.230875	-4.466846	3.001397
C	-2.356466	-4.390015	1.902329
H	-1.856938	-5.009833	1.155623
H	-3.253936	-3.950374	1.466228
H	-2.630278	-4.993422	2.770069
C	-0.528830	0.126087	2.777672
H	-1.502987	0.537525	2.505787
H	-0.329633	0.401226	3.816340
C	0.572258	0.648966	1.856100
H	1.541309	0.255130	2.187585
H	0.608358	1.729063	2.041160
C	0.400777	0.413342	0.342400
H	1.139519	1.074309	-0.126403
C	0.744550	-1.015577	-0.097174
H	0.799023	-1.080105	-1.186315
H	1.717632	-1.330969	0.294751
H	-0.005509	-1.746413	0.226696
C	-0.982788	0.857011	-0.158031
H	-1.288436	1.765798	0.380651
H	-1.729759	0.085379	0.086959
C	-1.053756	1.155589	-1.662336
H	-0.775618	0.267056	-2.240446
H	-2.098866	1.365818	-1.920634
C	-0.206687	2.346652	-2.059567
H	-0.371913	3.208050	-1.410653
C	0.036224	2.694486	-3.507114

H	-0.047475	3.734104	-3.809939
H	-0.308816	1.985431	-4.256012
C	1.195263	2.275932	-2.640953
C	2.173056	3.344855	-2.194607
H	1.670943	4.301528	-2.017907
H	2.676484	3.051969	-1.264414
H	2.946249	3.508718	-2.953445
C	1.857527	0.945504	-2.936921
H	2.386920	0.555306	-2.058236
H	1.144332	0.186625	-3.273176
H	2.597688	1.067891	-3.735419

Conformer 44

N	3.409615	2.518316	-0.217620
N	1.612564	1.473324	0.403691
C	2.930735	1.557265	0.573336
H	3.516667	0.947927	1.244511
C	1.235519	2.407167	-0.532932
H	0.207934	2.522150	-0.840009
C	2.360207	3.065308	-0.922053
H	2.502599	3.868043	-1.628725
C	4.807389	2.939906	-0.305712
H	5.133501	2.885095	-1.344738
H	5.419009	2.272075	0.300541
H	4.904730	3.961367	0.065434
C	0.731887	0.485536	1.056741
H	0.920084	0.538523	2.131890
H	-0.290301	0.823633	0.883929
C	0.953167	-0.925481	0.522571

H	0.263850	-1.570650	1.074351
H	1.964480	-1.255610	0.798303
C	0.773754	-1.099554	-0.995534
H	1.538134	-0.469599	-1.477247
C	1.082360	-2.548750	-1.381847
H	0.962371	-2.699128	-2.459250
H	2.110529	-2.818347	-1.118104
H	0.414235	-3.249968	-0.870169
C	-0.581906	-0.628092	-1.561700
H	-0.452498	-0.526158	-2.646521
H	-0.811737	0.382820	-1.198203
C	-1.812424	-1.516823	-1.327682
H	-2.648072	-1.064456	-1.874058
H	-1.655625	-2.492916	-1.802329
C	-2.217091	-1.743686	0.114997
H	-1.569839	-2.434114	0.652320
C	-3.671923	-1.856783	0.494680
H	-3.964664	-2.620146	1.209765
H	-4.407361	-1.673829	-0.285279
C	-2.866149	-0.681674	0.986738
C	-2.505120	-0.661920	2.459440
H	-2.266233	-1.665158	2.828589
H	-1.639100	-0.015517	2.657141
H	-3.338306	-0.277460	3.058744
C	-3.195187	0.686930	0.421340
H	-2.365576	1.396211	0.556620
H	-3.434062	0.652756	-0.645296
H	-4.062147	1.111467	0.939332

Conformer 45

N	-0.663353	-1.542084	3.970457
N	-0.558967	-2.125670	1.883841
C	-1.011103	-1.201632	2.728922
H	-1.567449	-0.318967	2.451377
C	0.109974	-3.086630	2.607010
H	0.568266	-3.940556	2.131972
C	0.042570	-2.724510	3.915505
H	0.425740	-3.203319	4.803241
C	-0.992520	-0.803512	5.189480
H	-1.617685	-1.425606	5.831803
H	-1.536433	0.102113	4.920286
H	-0.071335	-0.533951	5.708245
C	-0.670301	-2.069039	0.416043
H	-0.852270	-3.089444	0.072827
H	-1.561091	-1.479457	0.190568
C	0.583833	-1.461847	-0.209167
H	1.453681	-2.065269	0.078646
H	0.735956	-0.464726	0.217458
C	0.520208	-1.366180	-1.744194
H	1.424722	-0.819176	-2.035979
C	0.574351	-2.747625	-2.404188
H	0.654369	-2.649010	-3.490653
H	1.439454	-3.324407	-2.059590
H	-0.330138	-3.335576	-2.204262
C	-0.690603	-0.566097	-2.251225
H	-1.617071	-1.125027	-2.054849
H	-0.617362	-0.509230	-3.344291
C	-0.821828	0.859355	-1.699828
H	-1.728243	1.308990	-2.123706

H	-0.981442	0.834469	-0.612113
C	0.376420	1.726532	-2.033234
H	0.717032	1.590814	-3.059973
C	1.474400	2.023589	-1.032951
H	2.506901	2.018477	-1.370692
H	1.342392	1.672060	-0.011076
C	0.559537	3.127526	-1.501899
C	1.129766	4.161683	-2.452697
H	1.851461	3.716176	-3.145644
H	0.333778	4.623805	-3.047871
H	1.642677	4.957487	-1.900465
C	-0.449376	3.695812	-0.522426
H	-1.321383	4.102784	-1.049913
H	-0.808954	2.945128	0.189869
H	0.001420	4.511747	0.055340

Conformer 46

N	-0.010648	-2.268803	4.000759
N	-0.545570	-1.748928	1.964453
C	-0.200591	-2.749578	2.770827
H	-0.093745	-3.782995	2.477429
C	-0.570483	-0.585181	2.699847
H	-0.819045	0.364652	2.251903
C	-0.240652	-0.910351	3.977321
H	-0.154674	-0.301351	4.863357
C	0.344105	-3.053454	5.183597
H	1.200710	-2.591511	5.674177
H	0.608910	-4.063843	4.874434
H	-0.505290	-3.089673	5.866692

C	-0.776342	-1.840753	0.512470
H	-0.994215	-2.887461	0.286703
H	-1.678678	-1.260933	0.304132
C	0.430003	-1.320053	-0.267140
H	1.295691	-1.956668	-0.044474
H	0.675534	-0.315790	0.094725
C	0.209045	-1.269831	-1.788815
H	1.113724	-0.800312	-2.193773
C	0.094091	-2.671592	-2.395772
H	0.058849	-2.615223	-3.487746
H	0.949739	-3.299263	-2.123386
H	-0.822059	-3.182718	-2.073688
C	-0.991012	-0.401924	-2.202457
H	-1.928746	-0.905278	-1.925578
H	-1.003585	-0.356820	-3.298595
C	-1.004584	1.031760	-1.657096
H	-1.908448	1.528875	-2.030402
H	-1.104771	1.020117	-0.562068
C	0.216704	1.834799	-2.060141
H	0.503678	1.670915	-3.099069
C	1.373352	2.096356	-1.117028
H	2.387587	2.039290	-1.502514
H	1.275392	1.768478	-0.083071
C	0.486417	3.233373	-1.558765
C	1.055017	4.231079	-2.548223
H	1.724759	3.747254	-3.266826
H	0.252733	4.719910	-3.112742
H	1.625410	5.010855	-2.030792
C	-0.450686	3.854907	-0.541315
H	-1.330419	4.287314	-1.032113

H	-0.804726	3.130090	0.199694
H	0.057517	4.660262	0.001067

Conformer 47

N	-0.741139	-4.157108	1.772324
N	-1.137774	-2.025575	1.812241
C	-0.331717	-2.985934	2.259545
H	0.521838	-2.840082	2.903403
C	-2.096508	-2.601570	1.011975
H	-2.862698	-2.015822	0.528801
C	-1.849467	-3.938325	0.984593
H	-2.360876	-4.743559	0.480551
C	-0.126311	-5.458061	2.034549
H	-0.827890	-6.088698	2.583039
H	0.774437	-5.310861	2.630054
H	0.139504	-5.927328	1.086055
C	-0.978507	-0.582748	2.078520
H	-1.826298	-0.093898	1.596122
H	-1.082550	-0.436029	3.156609
C	0.348489	-0.014471	1.577484
H	1.176841	-0.482332	2.125918
H	0.345227	1.035435	1.892756
C	0.625916	-0.090694	0.063111
H	1.486897	0.569452	-0.099692
C	1.052730	-1.485052	-0.414583
H	1.450699	-1.426868	-1.431852
H	1.841118	-1.905132	0.220819
H	0.215215	-2.192275	-0.445245
C	-0.526863	0.448647	-0.795641

H	-1.394561	-0.225604	-0.718598
H	-0.211404	0.397371	-1.843893
C	-0.963985	1.887770	-0.488157
H	-1.743104	2.169447	-1.204846
H	-1.446081	1.947519	0.498834
C	0.170564	2.890351	-0.532013
H	0.860299	2.812401	0.309318
C	-0.051450	4.297318	-1.032293
H	0.419195	5.119313	-0.500283
H	-1.034095	4.533423	-1.434288
C	0.819974	3.353042	-1.820843
C	2.317744	3.586468	-1.785949
H	2.643919	3.963534	-0.810526
H	2.864363	2.656952	-1.989327
H	2.615732	4.320734	-2.542929
C	0.317337	2.852760	-3.161045
H	0.822579	1.922980	-3.449798
H	-0.761275	2.669111	-3.164182
H	0.523938	3.593996	-3.940638

Conformer 48

N	-0.272029	-4.603579	0.704096
N	-0.358563	-2.512654	1.270987
C	0.452641	-3.550625	1.081882
H	1.524838	-3.539532	1.202141
C	-1.646714	-2.914785	1.006330
H	-2.482922	-2.238778	1.088507
C	-1.595400	-4.225462	0.649758
H	-2.376195	-4.915054	0.369484

C	0.246981	-5.936766	0.399342
H	-0.171585	-6.659550	1.101815
H	1.333125	-5.925121	0.493551
H	-0.025222	-6.203555	-0.622784
C	0.070951	-1.146529	1.629768
H	-0.843998	-0.559699	1.724598
H	0.534203	-1.198316	2.618347
C	1.025844	-0.525615	0.612173
H	1.961990	-1.098845	0.591917
H	1.291820	0.452798	1.024869
C	0.507119	-0.338081	-0.826743
H	1.234176	0.334932	-1.297163
C	0.501201	-1.632394	-1.652291
H	0.360699	-1.401100	-2.712878
H	1.450166	-2.175244	-1.559549
H	-0.316595	-2.306485	-1.366983
C	-0.863327	0.351595	-0.898837
H	-1.634813	-0.306259	-0.468963
H	-1.131408	0.451460	-1.958197
C	-0.944504	1.736963	-0.244325
H	-1.966967	2.115176	-0.368137
H	-0.783249	1.657712	0.839156
C	0.038310	2.722579	-0.845847
H	0.086526	2.658860	-1.933518
C	1.345145	3.084133	-0.169621
H	2.239352	3.189176	-0.777922
H	1.531358	2.687914	0.827156
C	0.251548	4.114572	-0.300412
C	0.448581	5.229767	-1.308434
H	0.988446	4.880092	-2.194871

H	-0.516340	5.631393	-1.638686
H	1.023224	6.052937	-0.869159
C	-0.486804	4.548047	0.950960
H	-1.501289	4.885198	0.708724
H	-0.566438	3.745233	1.690632
H	0.036117	5.383492	1.429125

Conformer 49

N	-4.465478	-1.662858	-0.803716
N	-2.967776	-0.920561	0.579598
C	-3.777664	-0.585793	-0.421857
H	-3.861415	0.398411	-0.856281
C	-3.133027	-2.261912	0.841696
H	-2.575147	-2.764417	1.615945
C	-4.074985	-2.727367	-0.020686
H	-4.502172	-3.710739	-0.139937
C	-5.470637	-1.710616	-1.865424
H	-5.170169	-2.447683	-2.612023
H	-5.540320	-0.728306	-2.333869
H	-6.438403	-1.979625	-1.438170
C	-1.972541	-0.039537	1.218206
H	-2.274886	0.987260	1.003313
H	-2.060415	-0.191707	2.296223
C	-0.556473	-0.333487	0.727188
H	-0.280658	-1.351585	1.030277
H	0.111637	0.342544	1.268444
C	-0.351785	-0.171872	-0.786879
H	-1.078178	-0.831581	-1.288088
C	-0.619284	1.262622	-1.254241

H	-0.368733	1.378915	-2.313158
H	-1.670537	1.555346	-1.140106
H	-0.019114	1.980956	-0.686530
C	1.033018	-0.676897	-1.242511
H	0.986071	-0.818992	-2.329140
H	1.200026	-1.675043	-0.814699
C	2.253187	0.206986	-0.944238
H	3.110225	-0.251866	-1.453300
H	2.118480	1.186422	-1.417122
C	2.593283	0.375363	0.524436
H	2.489694	-0.545036	1.100801
C	2.286931	1.653519	1.282913
H	1.936941	1.590714	2.311085
H	1.867899	2.485920	0.720546
C	3.716675	1.271132	0.990823
C	4.557890	0.775702	2.151027
H	3.961150	0.193096	2.861205
H	5.374340	0.137355	1.794756
H	5.001970	1.616243	2.695866
C	4.498387	2.086690	-0.020799
H	5.272797	1.475013	-0.498372
H	3.862325	2.500423	-0.808649
H	4.996872	2.926847	0.475444

The following are the XYZ coordinates for the Omim Conformers

Confomer 1 (lowest energy)

N	0.712789	1.664724	1.565854
N	-0.390026	-0.005993	2.400581
C	0.640728	0.336387	1.631757

H	1.296033	-0.351560	1.122398
C	-1.005044	1.145800	2.838939
H	-1.872131	1.121225	3.479745
C	-0.311301	2.193643	2.320584
H	-0.451313	3.258085	2.428205
C	1.695926	2.427069	0.797430
H	1.170339	3.131974	0.152201
H	2.269044	1.739647	0.173998
H	2.362164	2.959512	1.477303
C	-0.891825	-1.379069	2.577282
H	-0.053451	-2.047929	2.372010
H	-1.158658	-1.497253	3.629965
C	-2.085536	-1.657780	1.657155
H	-2.953865	-1.097004	2.022176
H	-2.331528	-2.719912	1.769155
C	-1.838224	-1.317594	0.184152
H	-2.776539	-1.464580	-0.361549
C	-0.741682	-2.145900	-0.496225
H	-1.101862	-3.174603	-0.612192
H	0.151754	-2.221060	0.142875
C	-0.314343	-1.586933	-1.858710
H	0.240704	-2.357740	-2.404874
H	-1.207048	-1.368539	-2.458576
C	0.556707	-0.331053	-1.768289
H	1.471662	-0.573463	-1.200295
C	0.967386	0.232802	-3.128380
C	1.821688	1.493824	-3.009604
H	1.269384	2.298151	-2.505938
H	2.739805	1.298731	-2.439453
H	2.121044	1.867462	-3.992697

H	0.031704	0.457322	-1.204738
H	0.065264	0.452260	-3.714025
H	-1.618254	-0.244755	0.096866
H	1.518505	-0.535898	-3.685086

Conformer 2

N	-0.669252	-2.018208	0.766337
N	-1.481006	-0.341157	1.876536
C	-1.632662	-1.097039	0.790678
H	-2.404081	-0.974578	0.045924
C	-0.377754	-0.788428	2.567304
H	-0.050650	-0.322257	3.483134
C	0.129849	-1.841731	1.874301
H	0.977521	-2.478682	2.073112
C	-0.494132	-3.057339	-0.247926
H	0.520772	-3.000005	-0.643341
H	-1.204218	-2.886916	-1.056653
H	-0.674242	-4.036198	0.199225
C	-2.266807	0.860738	2.205256
H	-3.219583	0.765833	1.678180
H	-2.473380	0.820743	3.277179
C	-1.556166	2.157820	1.814564
H	-0.645592	2.275102	2.414882
H	-2.229930	2.965591	2.119382
C	-1.227142	2.269777	0.318209
H	-2.022726	1.783774	-0.267177
C	0.135707	1.689840	-0.076418
H	0.264168	0.689494	0.353872
H	0.928882	2.304459	0.369258

C	0.335499	1.617909	-1.590677
H	-0.512273	1.081639	-2.043510
H	0.306796	2.633347	-2.003681
C	1.638901	0.931876	-2.011403
H	2.491903	1.433903	-1.535500
C	1.686750	-0.564366	-1.688793
C	2.949654	-1.245043	-2.213423
H	3.021462	-1.150091	-3.302133
H	3.847173	-0.790796	-1.779987
H	2.966681	-2.313627	-1.970623
H	1.772697	1.058747	-3.092361
H	0.799226	-1.045392	-2.127480
H	-1.255954	3.324271	0.024610
H	1.623907	-0.713011	-0.600857

Conformer 3

N	-2.553712	0.752378	0.137387
N	-0.976780	1.678393	1.305550
C	-1.659129	0.551339	1.104890
H	-1.509725	-0.376721	1.635246
C	-1.446561	2.630525	0.430059
H	-1.040281	3.629207	0.402607
C	-2.435855	2.052277	-0.301894
H	-3.061751	2.449568	-1.085386
C	-3.488726	-0.241627	-0.388935
H	-4.505275	0.146938	-0.316089
H	-3.406301	-1.154678	0.200656
H	-3.238364	-0.457752	-1.428491
C	0.159510	1.831032	2.236242

H	0.401744	2.896047	2.248277
H	-0.189130	1.553306	3.233838
C	1.353160	0.989384	1.791098
H	1.061241	-0.068094	1.806330
H	2.135685	1.098474	2.550448
C	1.889472	1.382702	0.412651
H	1.057001	1.465411	-0.298793
C	2.918714	0.396523	-0.152123
H	3.815683	0.400779	0.478826
H	3.232928	0.766586	-1.135153
C	2.414682	-1.046429	-0.296255
H	3.176101	-1.623413	-0.834255
H	2.332610	-1.521075	0.691270
C	1.077796	-1.180646	-1.027703
H	1.125179	-0.648595	-1.988498
C	0.664439	-2.632845	-1.266234
C	-0.733807	-2.758331	-1.866997
H	-1.488967	-2.345060	-1.184042
H	-0.807618	-2.216682	-2.816927
H	-0.998652	-3.802538	-2.055727
H	0.282937	-0.681878	-0.448096
H	0.708328	-3.184938	-0.318060
H	2.339541	2.381252	0.474958
H	1.393882	-3.110820	-1.930676

Conformer 4

N	-3.916915	1.293157	0.421618
N	-2.444761	0.972205	1.982652
C	-2.810558	1.776765	0.987547

H	-2.291649	2.673693	0.686180
C	-3.342903	-0.068996	2.051886
H	-3.249627	-0.854599	2.785221
C	-4.268080	0.132911	1.076618
H	-5.140082	-0.438166	0.798882
C	-4.648241	1.899386	-0.691341
H	-4.743178	1.171061	-1.498205
H	-4.093121	2.766140	-1.050103
H	-5.635855	2.213931	-0.349917
C	-1.225854	1.111714	2.799085
H	-0.896466	2.148868	2.696977
H	-1.517705	0.953193	3.839682
C	-0.132930	0.141316	2.357206
H	-0.462381	-0.890110	2.533882
H	0.722268	0.309124	3.021770
C	0.295382	0.315901	0.900331
H	-0.545686	0.078057	0.233016
C	1.487066	-0.567080	0.526869
H	1.242048	-1.616608	0.739367
H	2.339875	-0.312692	1.169953
C	1.896012	-0.427298	-0.939333
H	1.045576	-0.696938	-1.581915
H	2.124465	0.626059	-1.154883
C	3.102531	-1.290308	-1.308565
H	2.878494	-2.343328	-1.087915
C	3.510304	-1.156923	-2.776331
C	4.721035	-2.016605	-3.134308
H	5.593635	-1.736500	-2.533818
H	4.518169	-3.077849	-2.952016
H	4.990491	-1.903658	-4.188601

H	3.953535	-1.017127	-0.669397
H	3.729722	-0.103627	-2.995817
H	0.555092	1.369041	0.717795
H	2.660290	-1.434109	-3.414073

Conformer 5

N	-3.392029	-1.035876	0.355762
N	-2.195191	-0.146768	1.930779
C	-2.177857	-0.995530	0.905584
H	-1.316972	-1.554954	0.572169
C	-3.461951	0.381839	2.036559
H	-3.717447	1.101139	2.798866
C	-4.214279	-0.178273	1.053220
H	-5.253053	-0.046788	0.794469
C	-3.795806	-1.868812	-0.777066
H	-4.490117	-2.636822	-0.435920
H	-2.911469	-2.337619	-1.204070
H	-4.270155	-1.239931	-1.532660
C	-1.032158	0.247424	2.744600
H	-1.368025	0.281107	3.783335
H	-0.297138	-0.555312	2.659280
C	-0.461163	1.592211	2.294583
H	0.377624	1.815071	2.964158
H	-1.211053	2.373886	2.465925
C	0.011037	1.629362	0.838496
H	-0.839660	1.463573	0.161043
C	1.136869	0.645817	0.511348
H	0.799556	-0.394282	0.647682
H	1.959713	0.786240	1.225641

C	1.669572	0.801089	-0.913328
H	0.845797	0.670605	-1.630024
H	2.031386	1.828715	-1.049615
C	2.794055	-0.180035	-1.243178
H	2.431129	-1.210884	-1.117896
C	3.347821	-0.008656	-2.658208
C	4.478583	-0.985988	-2.971660
H	5.316185	-0.853102	-2.278253
H	4.138287	-2.024295	-2.887865
H	4.859135	-0.839909	-3.986921
H	3.609196	-0.053455	-0.517453
H	3.707575	1.020878	-2.781219
H	0.357350	2.647140	0.626574
H	2.534652	-0.138805	-3.384734

Conformer 6

N	0.636962	2.226504	1.285652
N	-0.816876	0.822255	2.070069
C	0.334607	0.934679	1.410128
H	0.921552	0.116238	1.019333
C	-1.272108	2.086847	2.369386
H	-2.195876	2.249130	2.902154
C	-0.359466	2.967967	1.882636
H	-0.328228	4.045811	1.914431
C	1.825034	2.764040	0.622822
H	1.517938	3.399136	-0.209286
H	2.423040	1.934963	0.244687
H	2.412307	3.339958	1.339369
C	-1.553499	-0.433436	2.297726

H	-0.824349	-1.241312	2.215452
H	-1.911711	-0.418380	3.329291
C	-2.708347	-0.597504	1.301975
H	-3.522972	0.079222	1.584361
H	-3.092740	-1.615835	1.429139
C	-2.315781	-0.342573	-0.156344
H	-3.212681	-0.434763	-0.778110
C	-1.217699	-1.263998	-0.698224
H	-1.632669	-2.259323	-0.899861
H	-0.445687	-1.426730	0.065758
C	-0.556436	-0.708561	-1.962272
H	-1.287969	-0.709570	-2.778728
H	-0.293411	0.348083	-1.794135
C	0.704375	-1.462347	-2.394612
H	1.007598	-1.101982	-3.385077
C	1.888810	-1.298397	-1.436335
C	3.175194	-1.927824	-1.968372
H	3.038122	-2.999969	-2.144690
H	3.469863	-1.469236	-2.918194
H	4.003763	-1.808022	-1.263512
H	0.478811	-2.530298	-2.513374
H	1.653034	-1.747993	-0.460611
H	-1.997877	0.705332	-0.260435
H	2.055373	-0.222111	-1.262198

Conformer 7

N	-2.783838	1.156368	0.337395
N	-1.349158	1.029171	1.959336
C	-1.688954	1.705169	0.863736

H	-1.156699	2.554468	0.462961
C	-2.252389	0.004702	2.136072
H	-2.180066	-0.684041	2.963600
C	-3.154451	0.085884	1.122890
H	-4.022527	-0.514104	0.897928
C	-3.472738	1.603834	-0.873370
H	-3.454667	0.804024	-1.615406
H	-2.959416	2.479316	-1.270390
H	-4.502667	1.867200	-0.627907
C	-0.131967	1.248483	2.760607
H	0.179389	2.281854	2.587503
H	-0.418936	1.155631	3.810280
C	0.972512	0.264181	2.378316
H	0.639651	-0.758410	2.597132
H	1.821203	0.462086	3.042397
C	1.407880	0.372761	0.918048
H	0.539185	0.215103	0.264630
C	2.488159	-0.643744	0.546999
H	2.094433	-1.655164	0.708697
H	3.338100	-0.534054	1.231215
C	2.991036	-0.502819	-0.894854
H	3.538357	0.443351	-0.992402
H	3.721008	-1.299278	-1.082041
C	1.902483	-0.556954	-1.974098
H	2.387703	-0.492989	-2.956041
C	1.025782	-1.810653	-1.934974
C	0.017969	-1.854712	-3.082449
H	-0.642814	-0.978260	-3.060252
H	0.527227	-1.854746	-4.052137
H	-0.608189	-2.751260	-3.034298

H	1.260102	0.334262	-1.906962
H	0.486681	-1.864445	-0.977976
H	1.774414	1.389619	0.717448
H	1.666201	-2.701832	-1.971633

Conformer 8

N	2.037567	3.279380	1.642750
N	0.697538	1.608369	1.986166
C	1.850982	1.975334	1.432432
H	2.523106	1.322744	0.895583
C	0.121164	2.716709	2.565989
H	-0.827079	2.664375	3.078508
C	0.962036	3.763553	2.355081
H	0.891669	4.799066	2.650622
C	3.194634	4.062986	1.211207
H	2.851632	4.914630	0.622256
H	3.841076	3.435281	0.597331
H	3.745830	4.409998	2.087106
C	0.089010	0.268141	1.913846
H	0.887308	-0.429456	1.650836
H	-0.252309	0.019340	2.921338
C	-1.054913	0.223546	0.902851
H	-1.851712	0.902567	1.230430
H	-1.477156	-0.786406	0.951400
C	-0.641887	0.560619	-0.532917
H	-1.540003	0.512895	-1.157358
C	0.440701	-0.351063	-1.127241
H	1.403813	-0.176721	-0.622843
H	0.600303	-0.050070	-2.169654

C	0.127701	-1.851040	-1.082123
H	0.968850	-2.390625	-1.534260
H	0.077970	-2.200800	-0.040224
C	-1.160953	-2.240788	-1.807311
H	-1.128335	-1.851889	-2.834757
C	-1.392438	-3.751815	-1.843191
C	-2.674173	-4.134013	-2.579655
H	-3.553019	-3.684798	-2.102966
H	-2.647329	-3.791051	-3.619906
H	-2.817194	-5.218644	-2.589316
H	-2.026447	-1.761926	-1.327275
H	-1.428047	-4.137089	-0.815224
H	-0.306668	1.606027	-0.585618
H	-0.533230	-4.237368	-2.323893

Conformer 9

N	-0.941207	1.168124	1.866713
N	0.613863	-0.306911	2.192146
C	0.362220	0.996935	2.088671
H	1.096390	1.785285	2.155077
C	-0.567952	-0.991885	2.029278
H	-0.612497	-2.068801	2.069058
C	-1.544953	-0.068409	1.828519
H	-2.604081	-0.182359	1.657759
C	-1.619396	2.447558	1.660015
H	-2.042958	2.468225	0.654062
H	-0.894990	3.254614	1.767762
H	-2.407638	2.564438	2.405324
C	1.953010	-0.914683	2.296519

H	2.472346	-0.433893	3.129243
H	1.785610	-1.959190	2.568082
C	2.755757	-0.810899	0.997779
H	3.686930	-1.355743	1.186030
H	3.045787	0.233902	0.828438
C	2.054409	-1.366244	-0.253190
H	2.820027	-1.727728	-0.947907
C	1.178744	-0.346491	-0.988723
H	1.801289	0.509738	-1.284692
H	0.412709	0.051634	-0.313396
C	0.482485	-0.916124	-2.223522
H	1.234521	-1.341020	-2.899490
H	-0.160735	-1.753673	-1.922199
C	-0.335949	0.128009	-2.993854
H	-0.804389	-0.360768	-3.856591
C	-1.426374	0.839087	-2.181029
C	-2.470951	-0.106902	-1.590518
H	-2.956746	-0.691221	-2.378376
H	-2.023569	-0.817987	-0.885937
H	-3.257369	0.445591	-1.062881
H	0.346831	0.883385	-3.402743
H	-1.926097	1.564774	-2.831986
H	1.456510	-2.249670	0.014286
H	-0.961589	1.429306	-1.377724

Conformer 10

N	-1.095860	3.431252	2.448865
N	-2.044587	1.542882	1.959833
C	-1.325029	2.189127	2.875620

H	-0.986253	1.775375	3.813219
C	-2.276970	2.398426	0.906670
H	-2.837336	2.091780	0.037847
C	-1.685281	3.583222	1.212704
H	-1.636840	4.512165	0.667126
C	-0.357716	4.467085	3.172385
H	0.482511	4.801477	2.563262
H	0.015958	4.050272	4.107036
H	-1.023205	5.303875	3.387300
C	-2.435609	0.121843	2.029112
H	-2.754109	-0.076676	3.055064
H	-3.309244	0.013406	1.382243
C	-1.303527	-0.807490	1.597326
H	-1.676432	-1.831359	1.716153
H	-0.461485	-0.703624	2.293838
C	-0.829150	-0.590918	0.160471
H	-1.687311	-0.663574	-0.522981
C	0.240184	-1.599678	-0.261175
H	-0.163402	-2.616531	-0.166406
H	1.089833	-1.539344	0.432980
C	0.735456	-1.379422	-1.690353
H	-0.114214	-1.445115	-2.384311
H	1.132910	-0.358661	-1.785710
C	1.810713	-2.381424	-2.110328
H	1.414844	-3.401979	-2.013152
C	2.307190	-2.165888	-3.540455
C	3.386167	-3.167140	-3.948151
H	4.260904	-3.093331	-3.292214
H	3.011757	-4.195223	-3.889133
H	3.722003	-2.992096	-4.974522

H	2.660928	-2.315229	-1.416769
H	2.697683	-1.143975	-3.637948
H	-0.423305	0.424420	0.047145
H	1.457825	-2.237933	-4.232592

Confomer 11

N	0.779853	2.992164	1.867834
N	-0.528208	1.337724	2.375057
C	0.622081	1.670256	1.793114
H	1.312238	0.980510	1.331894
C	-1.132759	2.487476	2.831596
H	-2.084339	2.468500	3.339204
C	-0.312052	3.524632	2.518533
H	-0.405086	4.582829	2.705756
C	1.928820	3.749660	1.371151
H	1.575462	4.555785	0.726619
H	2.571256	3.083764	0.794548
H	2.487583	4.159115	2.214293
C	-1.114635	-0.013257	2.418797
H	-0.295069	-0.720284	2.276178
H	-1.506890	-0.157879	3.427789
C	-2.205439	-0.186219	1.361107
H	-3.041086	0.483094	1.597257
H	-2.586969	-1.207976	1.468831
C	-1.736615	0.048927	-0.077281
H	-2.607853	-0.049059	-0.734296
C	-0.646954	-0.912516	-0.556506
H	-0.993198	-1.943540	-0.414367
H	0.261408	-0.815235	0.059602

C	-0.266008	-0.685376	-2.021119
H	-1.146375	-0.872644	-2.649912
H	-0.008487	0.373396	-2.163681
C	0.897391	-1.555605	-2.505187
H	1.796446	-1.331987	-1.911751
C	0.622886	-3.060919	-2.458043
C	1.746965	-3.879279	-3.091336
H	1.878881	-3.615014	-4.146256
H	2.700761	-3.700891	-2.581499
H	1.535094	-4.951186	-3.038957
H	1.137450	-1.271681	-3.537400
H	-0.322154	-3.270336	-2.977032
H	-1.397591	1.088547	-0.195938
H	0.483188	-3.385495	-1.418601

Conformer 12

N	-2.798009	1.644195	-0.100636
N	-1.404138	1.342690	1.534456
C	-1.771136	2.175865	0.563826
H	-1.307871	3.125852	0.347059
C	-2.217394	0.232395	1.484602
H	-2.104562	-0.589609	2.173187
C	-3.093053	0.421729	0.462143
H	-3.895032	-0.197880	0.093966
C	-3.507603	2.261870	-1.221086
H	-3.529242	1.563460	-2.058753
H	-2.978709	3.166952	-1.519550
H	-4.524215	2.515074	-0.915445
C	-0.243933	1.513870	2.427788

H	0.072445	2.556110	2.337227
H	-0.597119	1.354978	3.449123
C	0.882433	0.553237	2.054068
H	0.531082	-0.476342	2.188454
H	1.689470	0.699772	2.780597
C	1.405278	0.762113	0.632961
H	0.573206	0.680364	-0.079527
C	2.499071	-0.232928	0.228743
H	3.375659	-0.087163	0.871292
H	2.823460	0.016700	-0.788002
C	2.078895	-1.708473	0.285054
H	2.904656	-2.312728	-0.105785
H	1.961300	-2.023396	1.329633
C	0.792961	-2.047577	-0.480251
H	-0.066949	-1.564336	0.009078
C	0.797024	-1.663711	-1.965339
C	1.918183	-2.325458	-2.766511
H	2.906958	-1.979289	-2.445277
H	1.892455	-3.415107	-2.649988
H	1.822225	-2.100422	-3.832704
H	0.615985	-3.126907	-0.394475
H	0.870302	-0.572331	-2.072679
H	1.796391	1.784034	0.540584
H	-0.170472	-1.947602	-2.397835

Conformer 13

N	1.440749	2.330837	2.363513
N	0.198571	0.622115	2.855317
C	1.341811	1.003177	2.290260

H	2.068422	0.343413	1.841556
C	-0.463571	1.745294	3.296981
H	-1.421859	1.685581	3.788352
C	0.315735	2.816504	2.993318
H	0.172248	3.870044	3.173796
C	2.556574	3.137417	1.869512
H	2.184450	3.858941	1.140280
H	3.283328	2.481427	1.390607
H	3.027936	3.657540	2.705352
C	-0.325377	-0.754283	2.896710
H	0.520133	-1.418924	2.710700
H	-0.672549	-0.936713	3.916039
C	-1.446374	-0.956945	1.876315
H	-2.315512	-0.360255	2.178286
H	-1.752464	-2.006762	1.951851
C	-1.064903	-0.623462	0.431260
H	-1.962729	-0.744449	-0.182335
C	0.060910	-1.486932	-0.142706
H	-0.250398	-2.539535	-0.124323
H	0.952212	-1.428821	0.499654
C	0.468762	-1.101157	-1.568237
H	0.827458	-0.061228	-1.577618
H	1.323656	-1.722075	-1.863782
C	-0.641842	-1.262507	-2.608440
H	-1.456989	-0.553801	-2.407734
C	-0.147141	-1.052414	-4.040649
C	-1.260584	-1.192271	-5.076389
H	-1.722038	-2.184879	-5.025489
H	-2.049300	-0.448681	-4.912164
H	-0.876766	-1.053096	-6.091544

H	-1.077722	-2.268067	-2.519069
H	0.648884	-1.777503	-4.255106
H	-0.795278	0.440177	0.350326
H	0.311555	-0.057708	-4.124400

Conformer 14

N	1.356981	3.483962	1.147258
N	-0.033767	1.884556	1.613885
C	1.146414	2.169185	1.068111
H	1.823522	1.450922	0.632060
C	-0.604395	3.059561	2.051215
H	-1.570683	3.081496	2.529979
C	0.267917	4.061727	1.763396
H	0.211027	5.123245	1.945526
C	2.544115	4.194631	0.672271
H	2.243739	4.949454	-0.056342
H	3.218291	3.481515	0.198217
H	3.047534	4.666541	1.517616
C	-0.667377	0.554761	1.667516
H	0.116784	-0.178067	1.465505
H	-1.008099	0.404666	2.694337
C	-1.817778	0.437890	0.669801
H	-2.608566	1.145498	0.945458
H	-2.245628	-0.561294	0.801634
C	-1.412287	0.657511	-0.791608
H	-2.310862	0.534100	-1.406121
C	-0.319327	-0.282649	-1.314857
H	0.637910	-0.087403	-0.805227
H	-0.138234	-0.041867	-2.369221

C	-0.649581	-1.770904	-1.188667
H	-0.771637	-2.040184	-0.131054
H	-1.618818	-1.967285	-1.668246
C	0.421463	-2.665985	-1.815275
H	1.380422	-2.493653	-1.304303
C	0.088935	-4.161006	-1.771640
C	-0.035054	-4.731460	-0.358204
H	0.867418	-4.523952	0.230722
H	-0.892851	-4.311198	0.179277
H	-0.170268	-5.816734	-0.386899
H	0.570209	-2.363200	-2.859773
H	0.873312	-4.704628	-2.310655
H	-1.097802	1.700872	-0.932367
H	-0.842980	-4.340623	-2.323326

Conformer 15

N	-0.773710	3.893235	1.290229
N	-1.567131	1.932811	1.770366
C	-1.507285	2.877175	0.834273
H	-1.974210	2.824905	-0.137797
C	-0.841813	2.354861	2.861862
H	-0.745100	1.750920	3.750673
C	-0.345786	3.584372	2.563118
H	0.262742	4.262157	3.141877
C	-0.486870	5.135544	0.572936
H	-0.949757	5.972683	1.096645
H	-0.895487	5.065625	-0.434372
H	0.592501	5.277897	0.516305
C	-2.215080	0.616883	1.628467

H	-2.784490	0.443078	2.544687
H	-2.926162	0.702703	0.802375
C	-1.198350	-0.492070	1.369449
H	-1.761808	-1.431823	1.335208
H	-0.516684	-0.573227	2.225882
C	-0.404053	-0.314090	0.075988
H	-1.100786	-0.202492	-0.768062
C	0.538623	-1.485976	-0.200012
H	-0.048088	-2.413032	-0.238085
H	1.237444	-1.596603	0.641386
C	1.327889	-1.316108	-1.498726
H	0.628103	-1.280226	-2.344426
H	1.838749	-0.343287	-1.483978
C	2.373912	-2.411833	-1.732874
H	3.139020	-2.346339	-0.947833
C	1.823577	-3.842631	-1.775046
C	0.738668	-4.058673	-2.829062
H	-0.167576	-3.485337	-2.603854
H	1.087217	-3.751145	-3.820901
H	0.451825	-5.112960	-2.887869
H	2.886583	-2.201358	-2.680242
H	1.437549	-4.124883	-0.787153
H	0.186924	0.612739	0.121740
H	2.658900	-4.523495	-1.973176

Conformer 16

N	0.236489	3.985984	1.750835
N	-0.138928	1.852328	1.627857
C	-0.691198	3.037639	1.883192

H	-1.722461	3.203553	2.156024
C	1.186801	2.050344	1.315971
H	1.842359	1.230949	1.066237
C	1.424622	3.386726	1.394873
H	2.326307	3.956100	1.232361
C	0.037151	5.418954	1.968894
H	0.624316	5.740355	2.830574
H	-1.020698	5.606634	2.157849
H	0.347571	5.964638	1.076694
C	-0.862007	0.565498	1.606506
H	-0.097947	-0.213883	1.590697
H	-1.405607	0.478613	2.550192
C	-1.798949	0.458934	0.404593
H	-2.591918	1.211578	0.497775
H	-2.295778	-0.513944	0.481529
C	-1.106250	0.600869	-0.954927
H	-1.873839	0.495511	-1.729897
C	0.014728	-0.409815	-1.224231
H	0.859815	-0.241773	-0.539681
H	0.413082	-0.214186	-2.226748
C	-0.413030	-1.875511	-1.134542
H	-0.731967	-2.119173	-0.110735
H	-1.292342	-2.034472	-1.773271
C	0.698381	-2.841454	-1.545939
H	1.581527	-2.673080	-0.912424
C	0.285718	-4.311067	-1.456120
C	1.403781	-5.263079	-1.876165
H	1.711139	-5.076343	-2.911186
H	2.286851	-5.140846	-1.238695
H	1.082883	-6.306619	-1.806124

H	1.012173	-2.614640	-2.574125
H	-0.596157	-4.476908	-2.088505
H	-0.711049	1.620550	-1.061681
H	-0.025570	-4.538963	-0.427655

Conformer 17

N	1.353996	-0.260290	-4.050406
N	2.017235	-0.853582	-2.072500
C	2.024743	0.132315	-2.966732
H	2.494922	1.094972	-2.832679
C	1.310967	-1.912514	-2.597507
H	1.168571	-2.831394	-2.049952
C	0.898077	-1.543336	-3.838412
H	0.331516	-2.078409	-4.584642
C	1.149063	0.525706	-5.267149
H	1.641915	0.030920	-6.105430
H	1.578395	1.517548	-5.126429
H	0.079649	0.617775	-5.460793
C	2.583732	-0.790463	-0.713382
H	3.136958	-1.719448	-0.556546
H	3.302179	0.033482	-0.708686
C	1.499933	-0.586634	0.341854
H	1.998513	-0.617548	1.317877
H	0.810581	-1.438752	0.320559
C	0.738000	0.731311	0.190293
H	1.461555	1.557355	0.144593
C	-0.252208	0.993773	1.329895
H	-0.669976	2.000085	1.201112
H	0.296288	1.008236	2.279800

C	-1.402255	-0.014794	1.398423
H	-1.862283	-0.094413	0.403038
H	-1.014167	-1.013492	1.642864
C	-2.482945	0.351144	2.420629
H	-3.312960	-0.359720	2.321816
C	-2.001486	0.347543	3.872932
C	-3.133662	0.602663	4.865955
H	-3.598728	1.578383	4.689386
H	-3.914976	-0.159443	4.775534
H	-2.769296	0.588155	5.897184
H	-2.895429	1.339365	2.175616
H	-1.223518	1.108264	4.012296
H	0.188481	0.735636	-0.762773
H	-1.531382	-0.620399	4.092362

Conformer 18

N	-3.305575	0.824231	0.643297
N	-1.831987	0.468343	2.194388
C	-2.200556	1.297514	1.220618
H	-1.684036	2.203149	0.941530
C	-2.726446	-0.577796	2.237073
H	-2.629454	-1.381188	2.950064
C	-3.652452	-0.354204	1.268039
H	-4.522439	-0.920430	0.975380
C	-4.039049	1.455767	-0.453994
H	-4.133899	0.746959	-1.277344
H	-3.485930	2.331242	-0.792606
H	-5.026318	1.760500	-0.104111
C	-0.610680	0.587159	3.010748

H	-0.291320	1.630733	2.949761
H	-0.896928	0.383714	4.045090
C	0.488379	-0.355307	2.525375
H	0.161092	-1.395607	2.647249
H	1.340222	-0.219342	3.201570
C	0.919021	-0.102732	1.081175
H	0.067806	-0.273042	0.408267
C	2.085535	-0.996976	0.653591
H	1.773960	-2.048662	0.719172
H	2.908689	-0.871744	1.368287
C	2.598819	-0.703748	-0.759238
H	2.966725	0.329761	-0.798474
H	3.467740	-1.345218	-0.952170
C	1.560907	-0.931698	-1.862189
H	0.748415	-0.195731	-1.769016
C	2.136172	-0.846492	-3.279748
C	2.699903	0.528399	-3.638426
H	1.944918	1.312414	-3.498005
H	3.569203	0.786715	-3.024129
H	3.018835	0.557891	-4.684568
H	1.103080	-1.920841	-1.720273
H	1.347490	-1.109815	-3.994650
H	1.210114	0.952096	0.963691
H	2.920043	-1.606013	-3.395424

Conformer 19

N	-1.212336	-2.238356	-0.307657
N	-1.939152	-0.492278	0.752581
C	-2.082435	-1.228632	-0.347057

H	-2.778225	-1.029936	-1.147807
C	-0.942019	-1.046726	1.520874
H	-0.642812	-0.616970	2.462361
C	-0.486065	-2.141642	0.858260
H	0.286378	-2.850297	1.111846
C	-1.046349	-3.271614	-1.329174
H	-1.718365	-3.061155	-2.160440
H	-1.286744	-4.246234	-0.903476
H	-0.015227	-3.259254	-1.685362
C	-2.607735	0.791791	1.020632
H	-3.486573	0.830698	0.371403
H	-2.957544	0.759168	2.055448
C	-1.692139	1.992222	0.777575
H	-0.853764	1.960504	1.483259
H	-2.284346	2.871271	1.054582
C	-1.186739	2.142528	-0.666440
H	-1.999348	1.874797	-1.356891
C	0.081934	1.354636	-1.028694
H	0.220131	1.411185	-2.115903
H	-0.042866	0.289015	-0.800393
C	1.353951	1.869629	-0.352168
H	1.493006	2.921742	-0.625175
H	1.240349	1.855748	0.738903
C	2.612724	1.087712	-0.747704
H	3.489684	1.602245	-0.335197
C	2.649723	-0.376381	-0.289928
C	2.681077	-0.546300	1.228571
H	3.548282	-0.035113	1.660517
H	1.787049	-0.127139	1.704508
H	2.748773	-1.602606	1.512127

H	2.722540	1.124063	-1.839253
H	3.541266	-0.848778	-0.717583
H	-0.987874	3.204470	-0.848579
H	1.791789	-0.924012	-0.708567

Conformer 20

N	-3.633686	-1.048666	-0.406589
N	-2.454550	-0.031079	1.102738
C	-2.433062	-0.979241	0.168940
H	-1.580025	-1.590500	-0.083253
C	-3.711275	0.531142	1.122516
H	-3.968774	1.327521	1.803096
C	-4.452097	-0.108141	0.180007
H	-5.481342	0.017682	-0.117386
C	-4.026160	-1.978741	-1.465806
H	-4.756234	-2.688741	-1.075833
H	-3.143520	-2.514888	-1.812677
H	-4.454363	-1.416582	-2.296124
C	-1.305537	0.413852	1.912459
H	-1.657613	0.500693	2.942699
H	-0.564305	-0.387713	1.880499
C	-0.732279	1.733799	1.399851
H	0.074986	2.012672	2.084834
H	-1.494111	2.516838	1.493572
C	-0.218436	1.683161	-0.042973
H	-1.064038	1.526951	-0.726933
C	0.865566	0.633918	-0.321581
H	1.144515	0.716545	-1.376937
H	0.462572	-0.384873	-0.205973

C	2.114931	0.764638	0.552814
H	2.523124	1.778388	0.441935
H	1.840105	0.660492	1.611248
C	3.206439	-0.262849	0.234026
H	2.795250	-1.277447	0.341679
C	3.836537	-0.115741	-1.153224
C	4.980440	-1.103288	-1.379159
H	5.777469	-0.956307	-0.642165
H	4.630998	-2.138170	-1.289402
H	5.418594	-0.982798	-2.374326
H	3.996647	-0.174741	0.989732
H	4.206847	0.911031	-1.272205
H	0.178823	2.674278	-0.287659
H	3.077567	-0.263095	-1.932440

Conformer 21

N	-3.571489	0.547309	0.473454
N	-2.110585	0.208718	2.040369
C	-2.473887	1.029252	1.057513
H	-1.959580	1.935418	0.777480
C	-3.000718	-0.841054	2.081859
H	-2.906745	-1.638539	2.801633
C	-3.918395	-0.628632	1.102629
H	-4.782896	-1.200658	0.806154
C	-4.297230	1.166354	-0.636288
H	-4.359115	0.458610	-1.464417
H	-3.757846	2.056354	-0.960265
H	-5.298413	1.446613	-0.304890
C	-0.899963	0.338761	2.871026

H	-0.588962	1.384973	2.815403
H	-1.197285	0.131644	3.901287
C	0.212472	-0.594873	2.399439
H	-0.118289	-1.636572	2.495001
H	1.045817	-0.471154	3.100266
C	0.686624	-0.317609	0.973282
H	-0.140825	-0.470088	0.264491
C	1.854225	-1.218811	0.562819
H	1.562193	-2.265845	0.714759
H	2.703551	-1.033123	1.233746
C	2.300493	-1.026854	-0.889660
H	3.063159	-1.780968	-1.119956
H	1.453194	-1.236245	-1.555802
C	2.874227	0.361526	-1.187558
H	3.639462	0.595572	-0.435475
C	3.490998	0.489629	-2.584626
C	2.485243	0.311245	-3.722876
H	2.069595	-0.702255	-3.746267
H	1.650256	1.016541	-3.622134
H	2.958050	0.492378	-4.692823
H	2.090031	1.124708	-1.079223
H	4.299175	-0.245407	-2.691214
H	0.983307	0.736524	0.891937
H	3.960148	1.476559	-2.671070

Conformer 22

N	-0.735348	-4.103932	0.669537
N	-1.421386	-2.273137	1.607711
C	-0.479197	-3.214474	1.629181

H	0.354768	-3.251610	2.314309
C	-2.306743	-2.565970	0.594960
H	-3.154972	-1.935117	0.380528
C	-1.880362	-3.715418	0.008401
H	-2.288661	-4.286789	-0.810538
C	0.049945	-5.303649	0.378615
H	-0.547584	-6.190288	0.594873
H	0.942997	-5.304504	1.003234
H	0.343311	-5.294532	-0.671452
C	-1.447040	-1.075347	2.468770
H	-2.483304	-0.729272	2.480470
H	-1.192417	-1.398314	3.481172
C	-0.502096	0.013197	1.966139
H	0.531225	-0.356991	1.999402
H	-0.556808	0.834717	2.690069
C	-0.831535	0.533101	0.566987
H	-0.754940	-0.282330	-0.166724
C	0.093334	1.672657	0.137699
H	1.132570	1.321240	0.176439
H	0.017062	2.494407	0.863116
C	-0.229073	2.202580	-1.260911
H	-0.113272	1.390328	-1.993303
H	-1.286360	2.498468	-1.291471
C	0.634398	3.394207	-1.685555
H	0.518429	4.205609	-0.953252
C	2.120809	3.071362	-1.854567
C	2.927282	4.271679	-2.346327
H	2.556400	4.624564	-3.315051
H	2.858369	5.106729	-1.640258
H	3.985614	4.019525	-2.464468

H	0.248341	3.784708	-2.635782
H	2.229542	2.238493	-2.562955
H	-1.874723	0.879817	0.542774
H	2.541425	2.724287	-0.901772

Conformer 23

N	2.928523	1.691452	0.288537
N	1.800801	0.899395	1.963556
C	1.760630	1.737305	0.930034
H	0.916966	2.351549	0.654533
C	3.033890	0.286547	1.978640
H	3.299967	-0.441284	2.728761
C	3.743199	0.785097	0.932039
H	4.748118	0.581562	0.596528
C	3.289524	2.477390	-0.891573
H	4.131325	3.129080	-0.650652
H	2.433800	3.083729	-1.188915
H	3.557119	1.802763	-1.705745
C	0.678972	0.594366	2.868489
H	1.068514	0.632148	3.888344
H	-0.043153	1.405892	2.759504
C	0.058209	-0.767379	2.554394
H	-0.784485	-0.893070	3.243821
H	0.779873	-1.554655	2.802522
C	-0.413740	-0.935723	1.107617
H	0.444694	-0.855969	0.423963
C	-1.509574	0.041498	0.673965
H	-1.181255	1.079696	0.833991
H	-2.388711	-0.094514	1.317921

C	-1.920195	-0.111764	-0.794496
H	-2.632609	0.685225	-1.044051
H	-1.039173	0.051392	-1.430906
C	-2.556999	-1.464777	-1.125039
H	-3.355825	-1.664836	-0.398412
C	-3.136822	-1.543088	-2.541173
C	-2.091912	-1.396580	-3.646973
H	-1.630317	-0.403326	-3.643770
H	-1.294150	-2.139757	-3.532972
H	-2.543410	-1.542698	-4.632656
H	-1.817140	-2.267726	-1.003761
H	-3.908399	-0.771522	-2.659297
H	-0.779690	-1.961235	0.998804
H	-3.646564	-2.506180	-2.655571

Conformer 24

N	-0.233260	4.193990	0.936909
N	-0.430196	2.125688	1.558937
C	-0.905987	3.059877	0.739166
H	-1.704029	2.919073	0.026310
C	0.587658	2.677454	2.303170
H	1.130589	2.105499	3.039236
C	0.709999	3.974264	1.916629
H	1.374750	4.753841	2.254072
C	-0.469400	5.465218	0.252186
H	-0.857699	6.196238	0.963942
H	-1.196771	5.309829	-0.545550
H	0.468574	5.820337	-0.178547
C	-0.849742	0.713165	1.592741

H	-0.989417	0.447625	2.643138
H	-1.824703	0.662088	1.101186
C	0.161139	-0.199370	0.903018
H	-0.214196	-1.221393	1.024517
H	1.120327	-0.157316	1.435007
C	0.362141	0.116319	-0.579225
H	-0.604194	0.041468	-1.096019
C	1.388867	-0.797846	-1.260954
H	2.379691	-0.599166	-0.832691
H	1.447089	-0.513325	-2.318447
C	1.090054	-2.297803	-1.157860
H	1.873072	-2.839285	-1.703135
H	1.180344	-2.622614	-0.113381
C	-0.275459	-2.703900	-1.717788
H	-0.371569	-2.309486	-2.738760
C	-0.511235	-4.217912	-1.736317
C	-0.556708	-4.851529	-0.345068
H	0.406084	-4.774025	0.171517
H	-1.316429	-4.366944	0.281500
H	-0.807779	-5.914400	-0.406996
H	-1.079313	-2.234133	-1.130349
H	0.274548	-4.698549	-2.333645
H	0.700023	1.155625	-0.694631
H	-1.456865	-4.418604	-2.253821

Conformer 25

N	0.331677	2.754861	3.082805
N	0.064980	0.653260	2.616344
C	-0.408673	1.673758	3.329525

H	-1.253001	1.631222	4.001115
C	1.140894	1.098779	1.881711
H	1.687061	0.445969	1.219073
C	1.311488	2.414710	2.175516
H	2.039218	3.129458	1.825534
C	0.148418	4.075992	3.684789
H	-0.016109	4.812054	2.897569
H	1.035205	4.335405	4.263272
H	-0.719691	4.049885	4.343372
C	-0.533892	-0.693368	2.554717
H	0.271551	-1.377656	2.281445
H	-0.855044	-0.949880	3.567050
C	-1.699645	-0.755233	1.567604
H	-2.501415	-0.098230	1.926719
H	-2.092486	-1.777663	1.613036
C	-1.339647	-0.401648	0.121946
H	-2.264447	-0.422493	-0.465627
C	-0.322083	-1.342864	-0.527283
H	-0.683101	-2.378142	-0.446676
H	0.636548	-1.318578	0.015347
C	-0.060585	-1.009197	-1.996782
H	-1.013702	-1.040474	-2.539245
H	0.306803	0.024582	-2.079439
C	0.942217	-1.960393	-2.652008
H	0.545149	-2.984310	-2.608578
C	1.275495	-1.610018	-4.106156
C	0.073619	-1.671432	-5.049600
H	-0.420796	-2.648848	-4.991733
H	-0.671894	-0.903687	-4.814273
H	0.385183	-1.516582	-6.087047

H	1.870379	-1.964590	-2.063101
H	2.047000	-2.302871	-4.461573
H	-0.978618	0.635480	0.065777
H	1.721946	-0.607261	-4.145142

Conformer 26

N	0.085741	-1.975334	2.199069
N	-1.664385	-0.698737	2.105235
C	-0.442220	-0.825609	2.618255
H	0.048079	-0.108691	3.258359
C	-1.927488	-1.805478	1.331739
H	-2.861203	-1.924702	0.805480
C	-0.831323	-2.607579	1.388803
H	-0.627824	-3.562604	0.930951
C	1.416532	-2.481718	2.534892
H	1.321166	-3.446069	3.035277
H	1.907606	-1.773597	3.201399
H	2.002358	-2.587413	1.620957
C	-2.539415	0.479555	2.261243
H	-3.458755	0.241701	1.721533
H	-2.788135	0.567034	3.321974
C	-1.917660	1.769988	1.728845
H	-1.028364	2.025788	2.319980
H	-2.652019	2.553917	1.944064
C	-1.577871	1.788110	0.232753
H	-2.434189	1.413612	-0.344783
C	-0.306425	1.034491	-0.176314
H	-0.434440	-0.048959	-0.049807
H	0.521833	1.334341	0.483078

C	0.091374	1.296934	-1.629108
H	-0.750026	1.039945	-2.287240
H	0.273419	2.370853	-1.767392
C	1.329852	0.509973	-2.057227
H	1.143712	-0.565949	-1.918739
C	1.734580	0.766333	-3.509426
C	2.969267	-0.029536	-3.927570
H	3.834357	0.228628	-3.306146
H	2.796246	-1.107539	-3.829772
H	3.235785	0.171610	-4.969193
H	2.171235	0.765362	-1.396712
H	1.923459	1.838660	-3.646344
H	-1.457891	2.837353	-0.060115
H	0.893030	0.515325	-4.168000

Conformer 27

N	-3.810176	0.623402	0.367863
N	-2.350900	0.307728	1.940893
C	-2.705658	1.107110	0.937734
H	-2.179904	1.999275	0.633634
C	-3.254196	-0.729390	2.011290
H	-3.169511	-1.509353	2.751592
C	-4.171766	-0.530290	1.029039
H	-5.044672	-1.098930	0.749602
C	-4.532138	1.224145	-0.753863
H	-4.637512	0.486005	-1.550576
H	-3.964120	2.076552	-1.124446
H	-5.514316	1.557995	-0.417859
C	-1.140684	0.447879	2.770119

H	-0.827902	1.492774	2.699260
H	-1.440564	0.257196	3.802951
C	-0.028214	-0.493889	2.315875
H	-0.362082	-1.533780	2.422095
H	0.800738	-0.363331	3.020966
C	0.458173	-0.235011	0.890204
H	-0.360997	-0.402586	0.175105
C	1.634707	-1.136825	0.507939
H	1.343094	-2.182382	0.670969
H	2.472615	-0.939664	1.189918
C	2.109514	-0.969088	-0.938774
H	2.865148	-1.737236	-1.135598
H	1.276172	-1.177010	-1.625406
C	2.694574	0.410511	-1.251035
H	3.512550	0.620633	-0.547399
C	3.213847	0.556311	-2.686194
C	4.399623	-0.349775	-3.018724
H	5.218064	-0.201490	-2.304495
H	4.125811	-1.410026	-2.999281
H	4.785982	-0.131760	-4.018906
H	1.933393	1.183654	-1.079132
H	3.508538	1.600572	-2.843148
H	0.749582	0.819783	0.797293
H	2.393188	0.360291	-3.389504

Conformer 28

N	-0.024280	3.453036	2.288164
N	-0.912294	1.501759	1.957213
C	-0.212826	2.242783	2.814858

H	0.139375	1.916724	3.781888
C	-1.175071	2.261862	0.840264
H	-1.726049	1.867418	0.001053
C	-0.620902	3.485909	1.046792
H	-0.602997	4.368954	0.427515
C	0.673295	4.568829	2.927852
H	1.481425	4.907574	2.278543
H	1.089199	4.230294	3.876305
H	-0.030969	5.382218	3.107484
C	-1.263806	0.081238	2.143443
H	-1.513864	-0.053577	3.198448
H	-2.173330	-0.087834	1.561913
C	-0.141722	-0.855215	1.701715
H	-0.489505	-1.874912	1.905279
H	0.738706	-0.696915	2.337707
C	0.239559	-0.724265	0.227255
H	-0.667065	-0.804353	-0.388491
C	1.249957	-1.787473	-0.206911
H	0.793825	-2.779080	-0.096157
H	2.107554	-1.768389	0.478127
C	1.760894	-1.602601	-1.639988
H	2.341203	-0.671689	-1.694525
H	2.464996	-2.414627	-1.858736
C	0.676450	-1.578712	-2.723535
H	1.168530	-1.475120	-3.698603
C	-0.219742	-2.818101	-2.749692
C	-1.183731	-2.817694	-3.934750
H	-1.834182	-1.935250	-3.914544
H	-0.637336	-2.803930	-4.883860
H	-1.823556	-3.705233	-3.930038

H	0.049563	-0.681783	-2.614080
H	-0.794955	-2.885571	-1.816074
H	0.664584	0.271024	0.033565
H	0.409068	-3.717297	-2.785398

Conformer 29

N	-1.338656	4.081379	1.146718
N	-1.458675	2.047079	1.890792
C	-1.936578	2.900010	0.987645
H	-2.685333	2.670174	0.244724
C	-0.515231	2.701776	2.650461
H	0.016402	2.209501	3.450214
C	-0.446362	3.979260	2.191307
H	0.152466	4.816519	2.514949
C	-1.599356	5.289429	0.363864
H	-2.017658	6.059885	1.013481
H	-2.311507	5.053416	-0.426619
H	-0.666611	5.638724	-0.081351
C	-1.811584	0.620604	2.004660
H	-2.028593	0.429999	3.058500
H	-2.737286	0.480116	1.440323
C	-0.704127	-0.288593	1.478346
H	-1.039392	-1.314884	1.660155
H	0.204014	-0.149570	2.079346
C	-0.391447	-0.082254	-0.004650
H	-1.305822	-0.249235	-0.592333
C	0.721274	-0.996129	-0.531294
H	1.640978	-0.815848	0.042991
H	0.940600	-0.697234	-1.562170

C	0.381552	-2.488411	-0.492149
H	0.196666	-2.799107	0.544306
H	-0.556121	-2.659758	-1.040381
C	1.478379	-3.387500	-1.073653
H	1.209772	-4.433233	-0.877446
C	1.713991	-3.216583	-2.576369
C	2.738977	-4.210112	-3.120815
H	3.708093	-4.086155	-2.624697
H	2.411241	-5.242499	-2.956798
H	2.893492	-4.074167	-4.195502
H	2.419374	-3.208592	-0.534362
H	2.055218	-2.196320	-2.792818
H	-0.092299	0.961959	-0.173314
H	0.760149	-3.343647	-3.106057

Conformer 30

N	-0.163182	-1.922501	1.595990
N	-0.985555	0.021409	2.096798
C	-1.223053	-1.125007	1.463420
H	-2.126194	-1.367997	0.925187
C	0.272691	-0.040274	2.649729
H	0.693077	0.781443	3.207441
C	0.788572	-1.258808	2.336879
H	1.739012	-1.708084	2.577873
C	-0.048275	-3.287340	1.083298
H	0.928587	-3.406729	0.613691
H	-0.823661	-3.452355	0.334264
H	-0.165233	-3.998097	1.903844
C	-1.888506	1.184501	2.138270

H	-2.849580	0.844622	1.743755
H	-2.031434	1.438817	3.191477
C	-1.370366	2.381457	1.343867
H	-0.437286	2.747540	1.790382
H	-2.110559	3.173419	1.502489
C	-1.179604	2.142172	-0.160310
H	-2.009673	1.532531	-0.546400
C	0.159263	1.515365	-0.563062
H	0.270105	0.511927	-0.134820
H	0.977029	2.115318	-0.140288
C	0.331846	1.445125	-2.083312
H	-0.528786	0.924324	-2.528082
H	0.295186	2.469215	-2.474016
C	1.636086	0.767157	-2.539146
H	2.415597	0.929426	-1.781445
C	1.517916	-0.731776	-2.837431
C	1.056405	-1.590221	-1.660035
H	1.700410	-1.430295	-0.785436
H	0.025573	-1.347138	-1.372696
H	1.086807	-2.653309	-1.919684
H	1.996900	1.257882	-3.448909
H	2.492155	-1.097117	-3.181485
H	-1.265796	3.109571	-0.667821
H	0.823523	-0.876174	-3.674275

Conformer 31

N	-0.556627	3.664214	2.235572
N	-1.612794	1.841185	1.719979
C	-0.843938	2.425638	2.637323

H	-0.510094	1.970837	3.558245
C	-1.819399	2.734130	0.692930
H	-2.410128	2.478791	-0.173031
C	-1.160547	3.878302	1.015903
H	-1.072346	4.817142	0.491343
C	0.243582	4.642828	2.971905
H	1.052671	4.998123	2.332665
H	0.665400	4.164867	3.856020
H	-0.390455	5.477519	3.274767
C	-2.077797	0.441733	1.764780
H	-2.412005	0.243407	2.786120
H	-2.952737	0.389889	1.112062
C	-0.993542	-0.537837	1.322157
H	-1.417762	-1.543408	1.426436
H	-0.149139	-0.486541	2.022293
C	-0.502690	-0.326613	-0.109663
H	-1.358779	-0.365241	-0.798847
C	0.535126	-1.367372	-0.531275
H	0.098211	-2.369269	-0.429032
H	1.388717	-1.330673	0.161058
C	1.032753	-1.159408	-1.962191
H	0.191385	-1.279439	-2.657682
H	1.375674	-0.121663	-2.075747
C	2.178192	-2.096979	-2.360528
H	3.056757	-1.867231	-1.742798
C	1.866630	-3.594189	-2.246561
C	0.657319	-4.038864	-3.067947
H	-0.273145	-3.594107	-2.697317
H	0.767833	-3.748720	-4.118514
H	0.536387	-5.125839	-3.032639

H	2.462987	-1.871689	-3.396214
H	1.716398	-3.866384	-1.193864
H	-0.062589	0.675778	-0.213008
H	2.750489	-4.152916	-2.574700

Conformer 32

N	-0.853655	1.084502	-4.060402
N	0.067162	1.081790	-2.096212
C	0.148032	0.591596	-3.332112
H	0.903804	-0.091860	-3.687548
C	-1.026894	1.913371	-2.030824
H	-1.298235	2.426999	-1.122432
C	-1.603943	1.918300	-3.261454
H	-2.471426	2.438689	-3.634822
C	-1.105796	0.806226	-5.474687
H	-2.117863	0.415581	-5.589056
H	-0.390140	0.062075	-5.823939
H	-0.988591	1.724134	-6.052076
C	0.960138	0.717710	-0.978786
H	1.987541	0.814901	-1.337345
H	0.806575	1.471881	-0.204652
C	0.676173	-0.690359	-0.458945
H	1.404537	-0.882956	0.336359
H	0.893497	-1.416604	-1.251899
C	-0.749149	-0.901044	0.061479
H	-1.464539	-0.790199	-0.764796
C	-1.163222	0.024327	1.214354
H	-2.163208	-0.278620	1.546012
H	-1.276718	1.055256	0.848718

C	-0.213196	0.029101	2.417195
H	0.760991	0.442341	2.122083
H	-0.611851	0.720958	3.169495
C	-0.020585	-1.346276	3.060833
H	0.443354	-2.037846	2.342885
C	0.833735	-1.318777	4.332375
C	2.286474	-0.907220	4.090154
H	2.758772	-1.559162	3.344454
H	2.366485	0.126309	3.735063
H	2.871807	-0.979716	5.011363
H	-1.007602	-1.765421	3.296066
H	0.814877	-2.316174	4.785811
H	-0.832400	-1.943767	0.384213
H	0.375057	-0.640049	5.063074

Conformer 33

N	1.816672	-0.970137	1.569388
N	1.020095	1.046825	1.502164
C	0.765460	-0.208377	1.870273
H	-0.149728	-0.557204	2.324783
C	2.278215	1.088282	0.947140
H	2.707315	2.004642	0.573781
C	2.778571	-0.174990	0.985736
H	3.726052	-0.571401	0.656054
C	1.923892	-2.410546	1.797890
H	2.048587	-2.917488	0.840316
H	1.010109	-2.762341	2.275934
H	2.776617	-2.612786	2.447393
C	0.066103	2.166865	1.624127

H	-0.196778	2.238702	2.682219
H	0.617363	3.072101	1.358857
C	-1.186619	2.000094	0.757208
H	-1.964695	2.613645	1.223585
H	-1.542137	0.963987	0.830219
C	-1.061157	2.426565	-0.710786
H	-2.056304	2.328506	-1.157803
C	-0.042850	1.676309	-1.579965
H	0.977168	1.965078	-1.293165
H	-0.165337	2.032027	-2.609584
C	-0.148046	0.148433	-1.564678
H	0.599464	-0.260120	-2.256510
H	0.130176	-0.226386	-0.572487
C	-1.523187	-0.408341	-1.935700
H	-1.791440	-0.060509	-2.940779
C	-1.589493	-1.938678	-1.892276
C	-1.363164	-2.526498	-0.497555
H	-0.342433	-2.337327	-0.140975
H	-2.063791	-2.093679	0.228376
H	-1.512833	-3.609958	-0.497519
H	-2.289403	-0.006135	-1.258333
H	-0.853130	-2.357585	-2.589751
H	-0.827298	3.497891	-0.743193
H	-2.572037	-2.259466	-2.254384

Conformer 34

N	-0.251310	3.512510	2.283674
N	-1.200310	1.610880	1.848862
C	-0.446977	2.270513	2.726982

H	-0.060902	1.866033	3.650080
C	-1.490751	2.457831	0.803781
H	-2.084883	2.139102	-0.037550
C	-0.899013	3.650924	1.075676
H	-0.885277	4.576499	0.522651
C	0.497894	4.562830	2.974471
H	1.249314	4.970390	2.296430
H	0.993256	4.132016	3.844948
H	-0.187142	5.349973	3.294659
C	-1.569634	0.185528	1.943372
H	-1.865269	-0.004852	2.977660
H	-2.453329	0.055804	1.314822
C	-0.432070	-0.731348	1.500219
H	-0.782592	-1.759665	1.645492
H	0.424503	-0.600552	2.173975
C	0.002210	-0.533245	0.048142
H	-0.872007	-0.644115	-0.607161
C	1.089469	-1.526545	-0.366988
H	0.700114	-2.546845	-0.258369
H	1.931827	-1.445813	0.332259
C	1.608583	-1.317033	-1.793108
H	2.093404	-0.334107	-1.858675
H	2.397257	-2.054096	-1.981111
C	0.542633	-1.415728	-2.893338
H	1.045889	-1.325414	-3.863886
C	-0.286861	-2.705440	-2.881866
C	0.549029	-3.980088	-3.001408
H	1.182164	-3.950672	-3.895533
H	1.204813	-4.121085	-2.135390
H	-0.091697	-4.863603	-3.074927

H	-0.136291	-0.554453	-2.829667
H	-1.000314	-2.662920	-3.712866
H	0.379117	0.489104	-0.099267
H	-0.894845	-2.752319	-1.967046

Conformer 35

N	-2.376803	0.786051	-0.447187
N	-0.883518	1.873395	0.689815
C	-1.373168	1.660262	-0.530283
H	-1.018862	2.123308	-1.438372
C	-1.594151	1.103251	1.582919
H	-1.373154	1.106280	2.638830
C	-2.532515	0.424816	0.872758
H	-3.291246	-0.273767	1.188657
C	-3.147856	0.254762	-1.570412
H	-2.893535	-0.796221	-1.717434
H	-2.903764	0.820713	-2.468638
H	-4.211870	0.358107	-1.357245
C	0.314485	2.662616	1.027721
H	0.399369	3.465646	0.291008
H	0.119045	3.119521	2.000045
C	1.559465	1.774744	1.048975
H	1.331146	0.871092	1.626308
H	2.338987	2.304122	1.606961
C	2.085355	1.411465	-0.346652
H	1.248209	1.264217	-1.044709
C	2.953165	0.139570	-0.360177
H	3.405050	-0.005339	0.629976
H	3.787556	0.274566	-1.055377

C	2.186639	-1.123092	-0.773829
H	1.880612	-1.019148	-1.824593
H	2.872909	-1.977930	-0.742321
C	0.949870	-1.450446	0.065033
H	0.227856	-0.620512	0.006011
C	0.251244	-2.734403	-0.385095
C	-1.038717	-3.017247	0.380831
H	-0.853890	-3.087792	1.458488
H	-1.772785	-2.214994	0.223846
H	-1.499065	-3.955536	0.058240
H	1.228567	-1.539456	1.124769
H	0.940271	-3.578719	-0.264582
H	2.649753	2.266701	-0.734175
H	0.035345	-2.670604	-1.460107

Conformer 36

N	-0.365883	-3.119586	0.778394
N	-1.485199	-1.633838	1.892269
C	-1.347655	-2.221537	0.705646
H	-1.929479	-1.998142	-0.175431
C	-0.555819	-2.169930	2.753783
H	-0.471149	-1.840969	3.777735
C	0.144324	-3.104162	2.058169
H	0.948736	-3.756438	2.360179
C	0.096934	-3.976211	-0.313358
H	1.156279	-3.786121	-0.493630
H	-0.470910	-3.740641	-1.214144
H	-0.059873	-5.022613	-0.046715
C	-2.365071	-0.489012	2.180179

H	-3.191801	-0.542356	1.466872
H	-2.777464	-0.645975	3.179645
C	-1.636535	0.851105	2.070766
H	-0.883147	0.926046	2.864930
H	-2.390322	1.614750	2.290927
C	-0.990241	1.113358	0.700585
H	-1.591020	0.639016	-0.092383
C	0.471252	0.656741	0.592443
H	0.588431	-0.364411	0.980510
H	1.083050	1.291663	1.247205
C	1.029276	0.709965	-0.832620
H	2.052376	0.312491	-0.822288
H	0.444277	0.037471	-1.479173
C	1.051134	2.109807	-1.449271
H	1.556802	2.799132	-0.758089
C	1.754071	2.151550	-2.806971
C	1.773063	3.549340	-3.421641
H	0.755835	3.922256	-3.587601
H	2.285778	4.260507	-2.764290
H	2.291453	3.551104	-4.385184
H	0.026544	2.487998	-1.567273
H	1.256664	1.453317	-3.493982
H	-1.038062	2.187129	0.493587
H	2.783347	1.787194	-2.689782

Conformer 37

N	-0.324888	-2.930302	0.674862
N	-1.645209	-1.239777	0.994578
C	-1.363172	-2.254369	0.181571

H	-1.887318	-2.489787	-0.732011
C	-0.759696	-1.268965	2.048541
H	-0.794589	-0.541052	2.843674
C	0.068632	-2.327326	1.849251
H	0.893682	-2.700898	2.435189
C	0.307247	-4.099020	0.063669
H	1.305800	-3.831555	-0.283462
H	-0.297422	-4.429708	-0.779548
H	0.368435	-4.899956	0.800751
C	-2.686431	-0.214361	0.793903
H	-3.332017	-0.567249	-0.014892
H	-3.280733	-0.202550	1.711012
C	-2.110776	1.172820	0.486547
H	-1.219436	1.341001	1.103982
H	-2.852764	1.902131	0.829062
C	-1.810815	1.454901	-0.991271
H	-2.752743	1.408240	-1.552799
C	-0.785308	0.546407	-1.682196
H	-0.637397	0.931434	-2.698222
H	-1.211424	-0.457912	-1.818189
C	0.581041	0.432902	-0.996406
H	0.484898	-0.068966	-0.021512
H	1.221440	-0.216689	-1.608302
C	1.289539	1.771935	-0.785659
H	0.687370	2.417942	-0.130504
C	2.684689	1.618617	-0.179399
C	3.382792	2.959013	0.040066
H	3.506023	3.495903	-0.906563
H	2.803074	3.600250	0.713768
H	4.375587	2.823244	0.478900

H	1.364254	2.298236	-1.747615
H	3.296946	0.987108	-0.836728
H	-1.475550	2.495517	-1.061513
H	2.606240	1.082843	0.777466

Conformer 38

N	2.515345	-0.428153	1.824605
N	0.630793	-1.364116	2.349674
C	1.746358	-1.501404	1.637627
H	1.984398	-2.339823	1.001367
C	0.684959	-0.161048	3.015976
H	-0.114727	0.174466	3.656413
C	1.866050	0.426119	2.689007
H	2.297865	1.365438	2.995259
C	3.832554	-0.200763	1.229899
H	4.031171	-0.983408	0.498515
H	4.595005	-0.225539	2.009627
H	3.835891	0.768570	0.730321
C	-0.514931	-2.291086	2.345915
H	-0.200766	-3.174205	1.783617
H	-0.682926	-2.594473	3.382198
C	-1.777245	-1.680345	1.738660
H	-2.080726	-0.802741	2.323646
H	-2.562493	-2.428959	1.891498
C	-1.700140	-1.324058	0.248553
H	-1.275997	-2.170149	-0.310643
C	-0.940528	-0.037968	-0.098678
H	0.132034	-0.142099	0.117927
H	-1.303220	0.783698	0.535690

C	-1.095756	0.352912	-1.569121
H	-0.788108	-0.489413	-2.205669
H	-2.158276	0.525685	-1.779901
C	-0.279362	1.592536	-1.939117
H	0.772612	1.417442	-1.669424
C	-0.353628	1.971376	-3.422039
C	-1.753565	2.369415	-3.890718
H	-2.454851	1.529757	-3.843756
H	-2.158614	3.180597	-3.274478
H	-1.731621	2.717645	-4.927308
H	-0.616830	2.443335	-1.330108
H	0.012672	1.132606	-4.028647
H	-2.728849	-1.216553	-0.114241
H	0.335452	2.804705	-3.602145

Conformer 39

N	-0.395246	-2.121159	1.493162
N	-1.492434	-0.306413	1.948135
C	-1.537024	-1.463319	1.292615
H	-2.358316	-1.804950	0.681270
C	-0.279734	-0.219367	2.592991
H	-0.010969	0.633349	3.196063
C	0.409601	-1.354987	2.307222
H	1.389528	-1.684761	2.615584
C	-0.031605	-3.410374	0.905677
H	0.750844	-3.260470	0.159524
H	-0.911208	-3.846160	0.432517
H	0.321199	-4.077189	1.693026
C	-2.519063	0.751404	1.894890

H	-3.352659	0.348623	1.313655
H	-2.867923	0.910140	2.918344
C	-2.005206	2.055083	1.282655
H	-1.153049	2.422609	1.868731
H	-2.808128	2.785479	1.432574
C	-1.636660	2.010002	-0.205262
H	-2.498160	1.655497	-0.787189
C	-0.395527	1.184738	-0.561891
H	-0.607114	0.110574	-0.469862
H	0.405806	1.405642	0.154908
C	0.105067	1.440634	-1.984830
H	-0.747631	1.422338	-2.675951
H	0.525774	2.452974	-2.041260
C	1.146278	0.419299	-2.451838
H	1.452153	0.667788	-3.475494
C	2.392120	0.333970	-1.566031
C	3.438069	-0.632652	-2.118545
H	3.027002	-1.644763	-2.219175
H	3.777529	-0.316727	-3.110782
H	4.316817	-0.687962	-1.468066
H	0.677083	-0.574789	-2.509269
H	2.106892	0.016942	-0.552024
H	-1.466367	3.044406	-0.525813
H	2.832261	1.334554	-1.460261

Conformer 40

N	-1.709379	0.246328	3.648523
N	-0.755865	1.234495	1.970207
C	-0.999691	0.054473	2.535524

H	-0.673884	-0.901179	2.153687
C	-1.334077	2.216380	2.743615
H	-1.268901	3.260414	2.480897
C	-1.929121	1.598779	3.797370
H	-2.479575	1.999134	4.633976
C	-2.168522	-0.794907	4.567889
H	-1.699906	-0.651598	5.541917
H	-1.888463	-1.768855	4.169215
H	-3.253665	-0.740104	4.661334
C	-0.064793	1.444709	0.686474
H	0.617405	2.288398	0.820444
H	0.540950	0.554302	0.504155
C	-1.055259	1.692231	-0.448298
H	-1.692536	2.546607	-0.191851
H	-1.714276	0.820925	-0.537683
C	-0.345845	1.967092	-1.780356
H	-1.118955	2.084402	-2.547970
C	0.647965	0.883765	-2.217026
H	1.510052	0.873946	-1.537100
H	1.053805	1.167962	-3.194876
C	0.043400	-0.518770	-2.321579
H	-0.336199	-0.844191	-1.339813
H	-0.830577	-0.478250	-2.984390
C	1.023269	-1.578269	-2.834440
H	1.419122	-1.264990	-3.809826
C	2.187453	-1.867411	-1.882971
C	3.089137	-2.995115	-2.381772
H	2.526620	-3.927441	-2.497527
H	3.522863	-2.745688	-3.355309
H	3.912881	-3.183432	-1.687250

H	0.473342	-2.511085	-3.012939
H	1.784796	-2.131339	-0.893016
H	0.174357	2.930740	-1.717606
H	2.792071	-0.962455	-1.741627

Conformer 41

N	-1.871064	0.711879	3.879544
N	-0.759211	1.499884	2.192008
C	-1.831542	0.795565	2.549354
H	-2.552483	0.364260	1.870764
C	-0.083473	1.872445	3.332621
H	0.825676	2.453459	3.293150
C	-0.781125	1.382504	4.390754
H	-0.601112	1.456741	5.452502
C	-2.896996	0.028393	4.668119
H	-2.434402	-0.773485	5.245068
H	-3.641764	-0.392364	3.993320
H	-3.376020	0.744777	5.336601
C	-0.315756	1.754158	0.809810
H	-1.204499	1.718716	0.173559
H	0.067764	2.777676	0.783504
C	0.735137	0.745241	0.355975
H	0.310875	-0.264038	0.422041
H	1.593572	0.777672	1.040111
C	1.200554	1.034565	-1.073106
H	0.342015	0.968846	-1.754310
C	2.312932	0.090875	-1.544041
H	3.203086	0.249768	-0.922331
H	2.598438	0.384298	-2.560026

C	1.952063	-1.400753	-1.521702
H	2.796536	-1.958973	-1.943107
H	1.860074	-1.744994	-0.482780
C	0.678721	-1.773673	-2.289000
H	-0.200530	-1.312924	-1.812711
C	0.700587	-1.391268	-3.770247
C	-0.535122	-1.879726	-4.522782
H	-0.616882	-2.971250	-4.477838
H	-1.451635	-1.459449	-4.092245
H	-0.498960	-1.590262	-5.577168
H	0.527593	-2.857174	-2.203468
H	1.603772	-1.808378	-4.233754
H	1.564350	2.068293	-1.132825
H	0.778831	-0.301303	-3.875366

Conformer 42

N	-3.222243	0.016322	2.731824
N	-1.974072	-0.054864	0.959888
C	-3.214519	0.135278	1.403416
H	-4.075253	0.349568	0.788059
C	-1.157301	-0.298859	2.040993
H	-0.099564	-0.479736	1.924968
C	-1.939625	-0.260743	3.151456
H	-1.698969	-0.408097	4.192424
C	-4.397729	0.126777	3.595936
H	-4.596427	-0.835503	4.069290
H	-5.256339	0.419735	2.992561
H	-4.213363	0.887316	4.354348
C	-1.521543	0.062712	-0.436507

H	-0.833088	-0.767197	-0.610823
H	-2.393712	-0.089472	-1.077524
C	-0.858069	1.412644	-0.695927
H	-0.038871	1.549682	0.019485
H	-1.584999	2.210303	-0.502600
C	-0.325687	1.531791	-2.129497
H	-1.169425	1.534089	-2.830881
C	0.675923	0.444382	-2.540523
H	1.046700	0.687397	-3.543202
H	0.166588	-0.524505	-2.647461
C	1.864642	0.289335	-1.590087
H	2.352022	1.265210	-1.450096
H	1.509740	-0.017641	-0.594719
C	2.892817	-0.726946	-2.089525
H	3.245606	-0.412251	-3.079670
C	4.097666	-0.904666	-1.160361
C	3.751103	-1.498589	0.205653
H	3.112511	-0.828338	0.793600
H	3.227370	-2.455708	0.094829
H	4.654925	-1.680913	0.794329
H	2.400042	-1.699528	-2.233639
H	4.596819	0.063487	-1.023988
H	0.150505	2.514473	-2.220611
H	4.825817	-1.556945	-1.655420

Conformer 43

N	0.403219	1.992266	1.524188
N	-0.781289	0.276272	2.120317
C	0.347563	0.661141	1.530917

H	1.089247	0.001065	1.107456
C	-1.478696	1.401599	2.500282
H	-2.433252	1.340217	2.998306
C	-0.734619	2.477962	2.131978
H	-0.910457	3.535404	2.253151
C	1.486745	2.794931	0.958212
H	1.073858	3.490104	0.226054
H	2.194707	2.129809	0.463296
H	1.991037	3.343144	1.754336
C	-1.273612	-1.109997	2.180635
H	-0.404990	-1.757549	2.044550
H	-1.658613	-1.277774	3.189070
C	-2.346346	-1.360962	1.117417
H	-3.256783	-0.817576	1.395992
H	-2.594970	-2.427216	1.164376
C	-1.933120	-0.972600	-0.305323
H	-2.797169	-1.126392	-0.961164
C	-0.746866	-1.758992	-0.871836
H	-1.027247	-2.817136	-0.936411
H	0.113778	-1.721207	-0.188823
C	-0.309242	-1.257015	-2.254080
H	0.321116	-2.019380	-2.727036
H	-1.202411	-1.177860	-2.884820
C	0.448822	0.087472	-2.250283
H	0.227040	0.643470	-1.324092
C	1.968897	-0.043341	-2.413267
C	2.679321	-0.795098	-1.287866
H	2.596564	-0.260151	-0.330771
H	2.272128	-1.802752	-1.148437
H	3.747517	-0.900877	-1.496052

H	0.075766	0.719488	-3.062961
H	2.406864	0.957346	-2.511709
H	-1.728208	0.106803	-0.343898
H	2.173698	-0.554619	-3.361705

Conformer 44

N	-2.123729	-1.397400	3.426048
N	-2.072179	-1.162481	1.270349
C	-1.370064	-1.543039	2.335561
H	-0.354346	-1.908625	2.317192
C	-3.316865	-0.753135	1.693042
H	-4.063944	-0.395502	1.001946
C	-3.351764	-0.903284	3.043427
H	-4.136372	-0.709316	3.757882
C	-1.728361	-1.727816	4.795547
H	-1.848831	-0.846740	5.427115
H	-0.682560	-2.034545	4.798861
H	-2.349558	-2.544615	5.166034
C	-1.564161	-1.099560	-0.110695
H	-0.737439	-1.810164	-0.175931
H	-2.362968	-1.459574	-0.764416
C	-1.122811	0.313827	-0.481280
H	-0.350298	0.638895	0.225822
H	-1.970656	0.998546	-0.361710
C	-0.597321	0.398067	-1.919396
H	-1.416064	0.180709	-2.616532
C	0.588659	-0.524730	-2.232789
H	0.915368	-0.316380	-3.256937
H	0.257245	-1.572029	-2.246648

C	1.782783	-0.392799	-1.278216
H	1.501446	-0.754199	-0.276689
H	2.573259	-1.070138	-1.623556
C	2.359952	1.021715	-1.148153
H	3.192489	0.992114	-0.433627
C	2.861076	1.617032	-2.465837
C	3.518640	2.982646	-2.276239
H	2.814364	3.702291	-1.843130
H	4.381232	2.914601	-1.604255
H	3.868579	3.390024	-3.229252
H	1.613460	1.700047	-0.709588
H	2.028004	1.714289	-3.174002
H	-0.312226	1.439372	-2.104523
H	3.575958	0.921895	-2.925428

Conformer 45

N	-2.134402	-0.260963	4.540670
N	-1.075395	-0.576763	2.673389
C	-2.106993	0.043408	3.242706
H	-2.809658	0.685921	2.733916
C	-0.414165	-1.297390	3.641931
H	0.461714	-1.884916	3.415291
C	-1.077665	-1.102473	4.812246
H	-0.895334	-1.490920	5.802693
C	-3.122489	0.208898	5.512913
H	-3.662400	-0.646489	5.922913
H	-3.824269	0.875372	5.011803
H	-2.614751	0.751293	6.312167
C	-0.657801	-0.442067	1.266892

H	-0.314191	-1.427885	0.946492
H	-1.550880	-0.198020	0.684848
C	0.420763	0.626685	1.099185
H	1.296745	0.352788	1.701104
H	0.039929	1.574538	1.498590
C	0.832424	0.807340	-0.365998
H	-0.050882	1.081919	-0.957374
C	1.527229	-0.413857	-0.978062
H	0.828911	-1.261477	-1.038628
H	2.342536	-0.726595	-0.311102
C	2.096428	-0.154961	-2.376947
H	2.793369	0.692859	-2.332366
H	2.692349	-1.025832	-2.676751
C	1.036347	0.110212	-3.447726
H	0.456345	1.008386	-3.194484
C	1.633457	0.290714	-4.844187
C	0.571389	0.535318	-5.914198
H	-0.126880	-0.307159	-5.974665
H	-0.011093	1.436845	-5.692238
H	1.024296	0.665297	-6.901609
H	0.321933	-0.725668	-3.464235
H	2.219561	-0.600373	-5.103787
H	1.513115	1.665825	-0.418179
H	2.340403	1.130190	-4.827216

Conformer 46

N	-1.726427	-0.552193	4.660454
N	-0.688358	0.373894	2.996047
C	-1.074858	-0.786791	3.521033

H	-0.889438	-1.760009	3.093235
C	-1.111886	1.388600	3.824756
H	-0.913260	2.425360	3.603189
C	-1.758901	0.809234	4.870006
H	-2.230114	1.241480	5.738348
C	-2.295722	-1.564396	5.550644
H	-1.802905	-1.510267	6.522590
H	-2.135361	-2.551001	5.115915
H	-3.366337	-1.386210	5.660886
C	0.007365	0.547823	1.708873
H	0.806189	1.276772	1.871868
H	0.475836	-0.409370	1.471074
C	-0.947527	1.001304	0.606298
H	-1.463610	1.912342	0.932810
H	-1.716054	0.231289	0.461685
C	-0.219115	1.269669	-0.715018
H	-0.957916	1.650477	-1.429525
C	0.476125	0.045710	-1.316455
H	-0.239226	-0.787338	-1.377866
H	1.292420	-0.292131	-0.661464
C	1.052465	0.319375	-2.706233
H	0.238172	0.632587	-3.371285
H	1.749733	1.166895	-2.650380
C	1.774730	-0.892522	-3.296815
H	1.064177	-1.726247	-3.389963
C	2.426068	-0.630798	-4.658717
C	1.431712	-0.303648	-5.772787
H	0.679518	-1.094823	-5.872898
H	0.904763	0.638299	-5.587713
H	1.942687	-0.206793	-6.735280

H	2.548394	-1.223447	-2.590477
H	3.003576	-1.518281	-4.943016
H	0.511610	2.077435	-0.573293
H	3.151475	0.187717	-4.560525

Conformer 47

N	0.001019	2.450797	1.333021
N	-1.576150	0.973685	1.150590
C	-0.274198	1.147136	1.362574
H	0.446329	0.359935	1.520407
C	-2.157202	2.211053	0.981867
H	-3.213331	2.326482	0.796417
C	-1.170324	3.137173	1.098912
H	-1.198801	4.213323	1.038069
C	1.320920	3.047676	1.537402
H	2.080567	2.272679	1.429958
H	1.376632	3.485781	2.534579
H	1.484942	3.814426	0.780633
C	-2.270836	-0.322085	1.044338
H	-1.539756	-1.090743	1.293931
H	-3.045196	-0.336295	1.815313
C	-2.880452	-0.539560	-0.341859
H	-3.720827	0.154745	-0.462721
H	-3.321711	-1.542911	-0.329561
C	-1.944704	-0.396737	-1.550089
H	-2.589376	-0.413570	-2.435425
C	-0.858785	-1.467298	-1.730489
H	-0.526057	-1.424240	-2.774713
H	-1.295076	-2.465727	-1.595977

C	0.381018	-1.320361	-0.843687
H	0.696272	-0.266830	-0.856345
H	0.143947	-1.574235	0.200306
C	1.552700	-2.198953	-1.284845
H	1.889981	-1.870865	-2.276996
C	2.734590	-2.182387	-0.310013
C	3.337265	-0.795238	-0.078872
H	3.620068	-0.323573	-1.026292
H	2.629249	-0.122592	0.424977
H	4.231172	-0.852612	0.548530
H	1.201247	-3.231504	-1.403169
H	3.516128	-2.846726	-0.695020
H	-1.479473	0.599863	-1.547034
H	2.418209	-2.609712	0.651285

Conformer 48

N	4.243615	0.640427	0.987956
N	2.271749	-0.264282	1.022015
C	3.506429	-0.373340	0.533393
H	3.854542	-1.157669	-0.122017
C	2.215436	0.861761	1.811902
H	1.309725	1.159974	2.315699
C	3.450807	1.429305	1.792984
H	3.831301	2.313655	2.279794
C	5.659550	0.870187	0.700024
H	5.781045	1.863227	0.267078
H	6.004339	0.120876	-0.011758
H	6.235488	0.789259	1.622659
C	1.146320	-1.157818	0.686220

H	1.508352	-2.184710	0.777293
H	0.389523	-1.003686	1.458021
C	0.595677	-0.877174	-0.710791
H	-0.198166	-1.611180	-0.887641
H	1.376141	-1.088383	-1.452473
C	0.060205	0.543747	-0.910064
H	0.880596	1.266662	-0.802073
C	-1.089075	0.942552	0.025800
H	-1.423461	1.946002	-0.263124
H	-0.719446	1.044260	1.056840
C	-2.295193	-0.004205	0.027881
H	-2.012236	-0.985970	0.437296
H	-3.037527	0.402515	0.723541
C	-2.934189	-0.200545	-1.348907
H	-2.199373	-0.637951	-2.038705
C	-4.178118	-1.096016	-1.335161
C	-5.358755	-0.514855	-0.557137
H	-5.142499	-0.422533	0.512627
H	-5.624104	0.480086	-0.932985
H	-6.241050	-1.154143	-0.656147
H	-3.197976	0.782141	-1.765349
H	-3.912351	-2.078861	-0.922801
H	-0.270723	0.630253	-1.950048
H	-4.486599	-1.273572	-2.371842

Conformer 49

N	-3.576668	0.280758	2.074315
N	-1.433618	-0.043818	1.983086
C	-2.606713	-0.617450	2.246846

H	-2.748888	-1.642673	2.552819
C	-1.661754	1.264874	1.626437
H	-0.856459	1.931983	1.362662
C	-3.004265	1.470914	1.685003
H	-3.594571	2.351822	1.487389
C	-5.005486	0.054285	2.294178
H	-5.329678	0.597914	3.183610
H	-5.179546	-1.012314	2.429782
H	-5.559858	0.399980	1.421015
C	-0.128750	-0.734200	1.997606
H	0.630496	0.046869	1.926360
H	-0.021142	-1.214518	2.973088
C	-0.005152	-1.741505	0.856000
H	-0.732847	-2.548951	1.006390
H	0.982507	-2.204590	0.952690
C	-0.184593	-1.139570	-0.541815
H	-0.054955	-1.948302	-1.269876
C	0.772671	0.004674	-0.896317
H	0.579366	0.883566	-0.263190
H	0.538127	0.326992	-1.916102
C	2.255232	-0.358513	-0.802214
H	2.501398	-0.666216	0.222733
H	2.449028	-1.234703	-1.432810
C	3.197877	0.787195	-1.190720
H	4.230265	0.437802	-1.065168
C	3.028352	1.324680	-2.617369
C	3.179481	0.256101	-3.700209
H	4.133761	-0.274386	-3.598360
H	2.377300	-0.490459	-3.652823
H	3.150929	0.703563	-4.698214

H	3.066301	1.614162	-0.479098
H	3.778776	2.108226	-2.775257
H	-1.221231	-0.794691	-0.659859
H	2.054306	1.820497	-2.722609

Conformer 50

N	-1.794526	-0.406531	4.301167
N	-0.809989	-0.628412	2.381292
C	-1.303162	0.251348	3.250491
H	-1.304502	1.323230	3.124339
C	-0.995624	-1.894144	2.890774
H	-0.674792	-2.774879	2.356827
C	-1.608401	-1.756274	4.095750
H	-1.921306	-2.491302	4.819638
C	-2.414579	0.198228	5.480540
H	-3.429843	-0.185106	5.589515
H	-2.447377	1.279445	5.349708
H	-1.823885	-0.043924	6.365230
C	-0.253188	-0.310149	1.054843
H	0.139915	0.708825	1.103761
H	0.591483	-0.983859	0.899773
C	-1.305805	-0.447208	-0.043674
H	-2.155551	0.200589	0.203238
H	-1.680823	-1.478292	-0.057999
C	-0.749639	-0.075234	-1.422008
H	-1.586333	-0.063223	-2.129927
C	0.330454	-1.029190	-1.939845
H	-0.045213	-2.059571	-1.880015
H	1.218807	-0.987042	-1.295339

C	0.750509	-0.740544	-3.386116
H	1.509760	-1.476923	-3.676696
H	-0.110101	-0.909036	-4.045711
C	1.301241	0.668988	-3.633959
H	0.508529	1.416846	-3.492385
C	2.506146	1.039233	-2.766504
C	3.097326	2.398172	-3.137588
H	3.438505	2.405171	-4.178482
H	2.351940	3.194739	-3.028530
H	3.953434	2.649202	-2.503665
H	1.589683	0.742078	-4.689896
H	3.275465	0.261310	-2.863508
H	-0.364122	0.951933	-1.389251
H	2.215563	1.051505	-1.705791

^{1}H and ^{13}C NMR Spectra of the IL Products

PhytylL-1

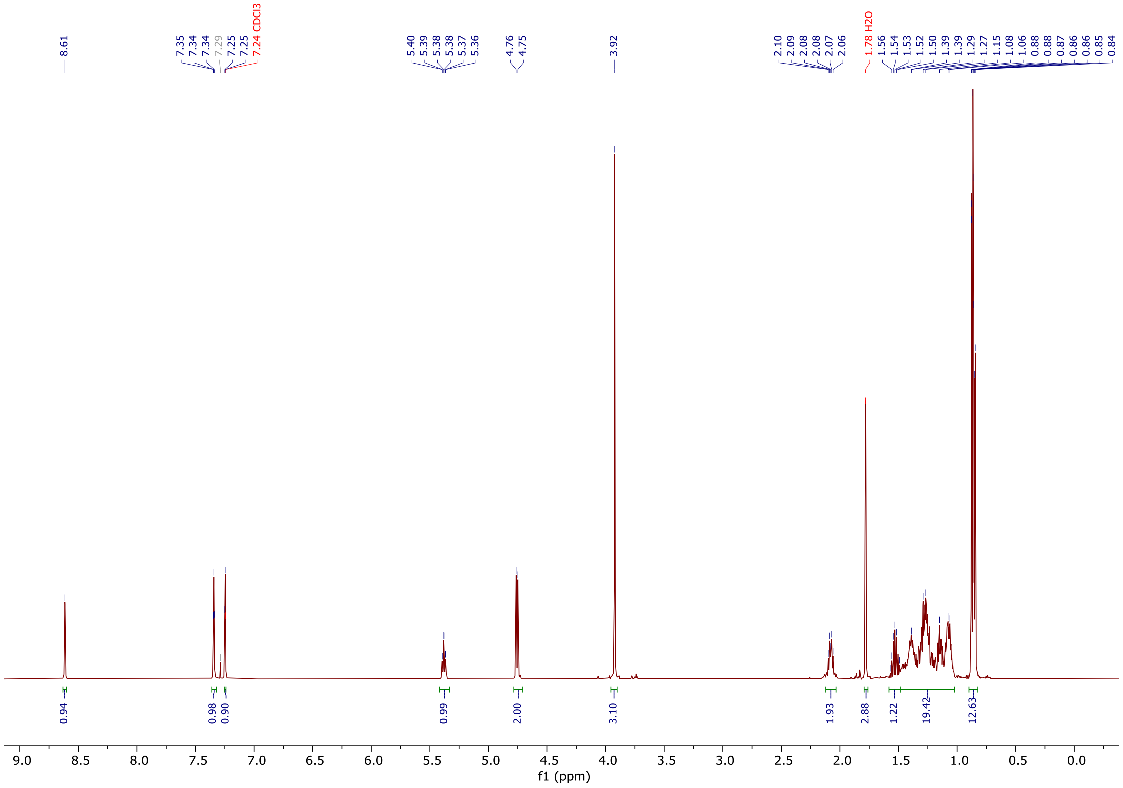

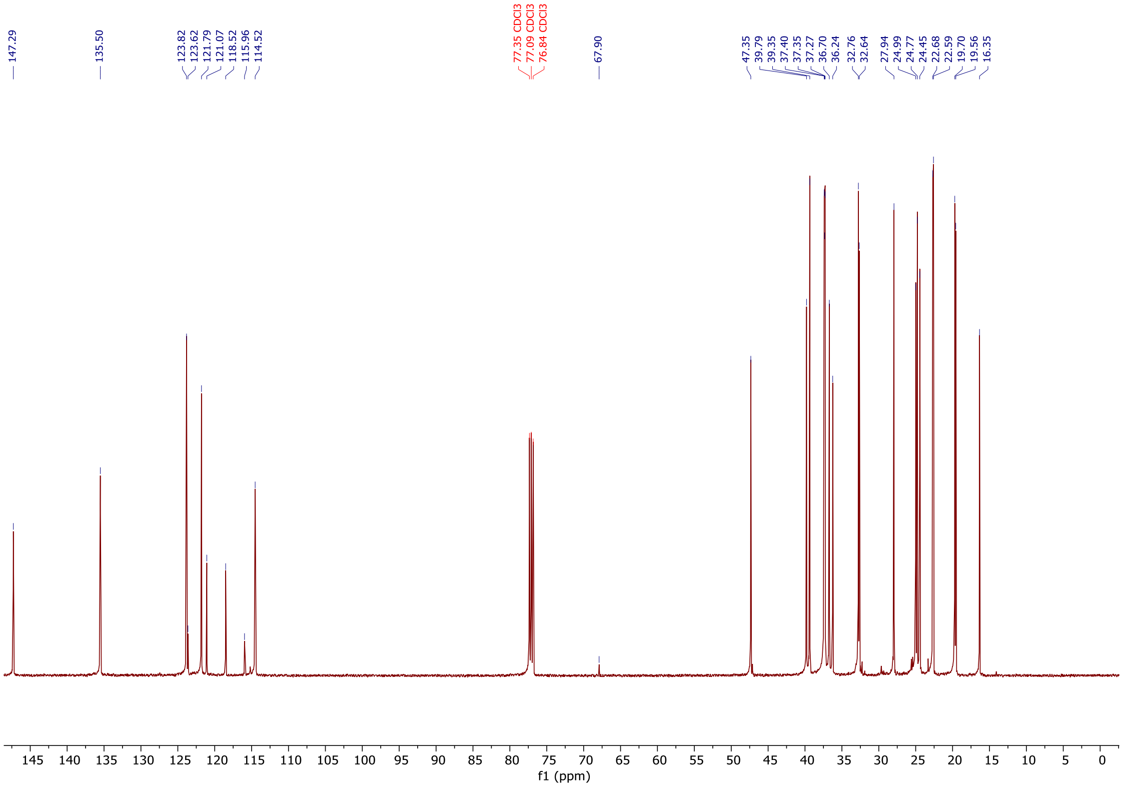

PhytylL-2

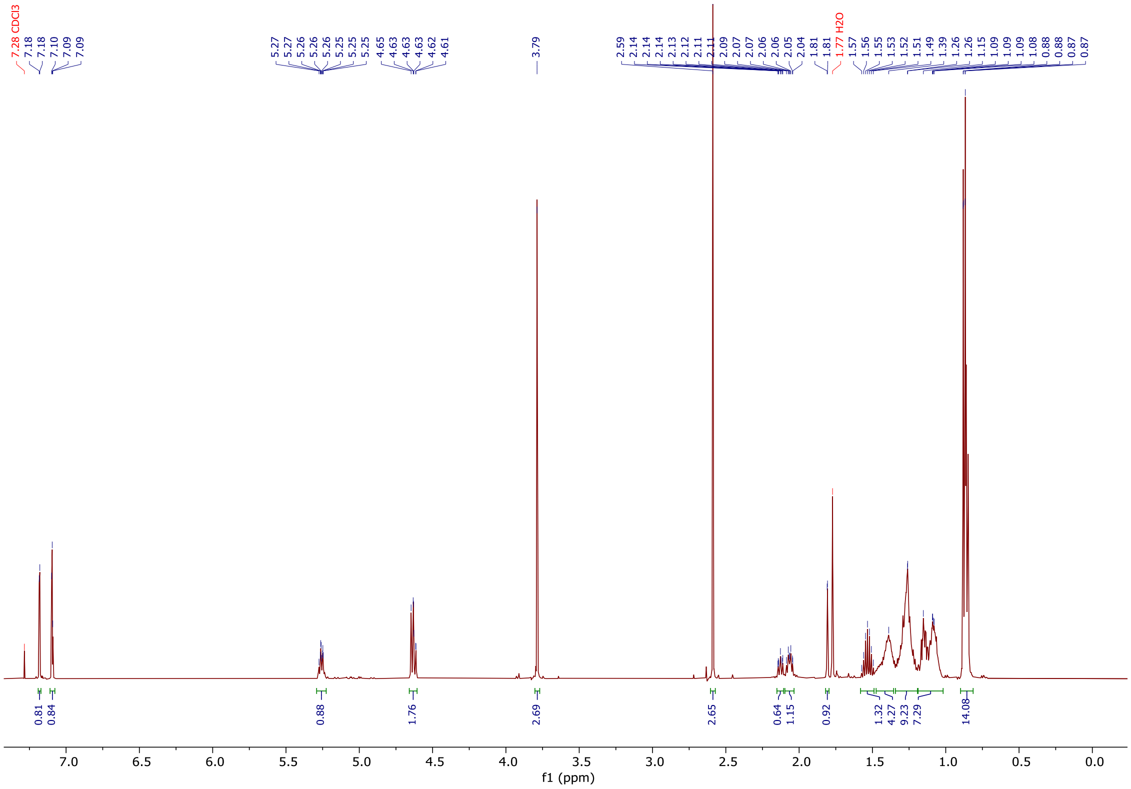

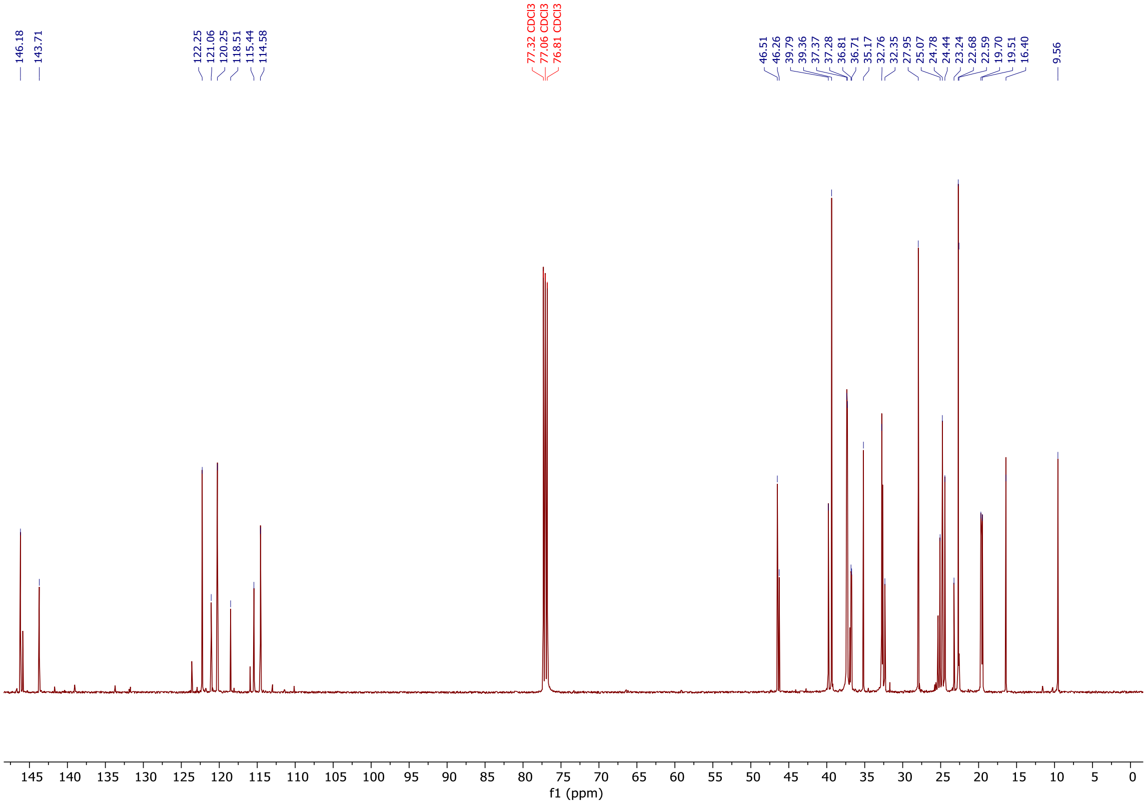

FarnesylL-2

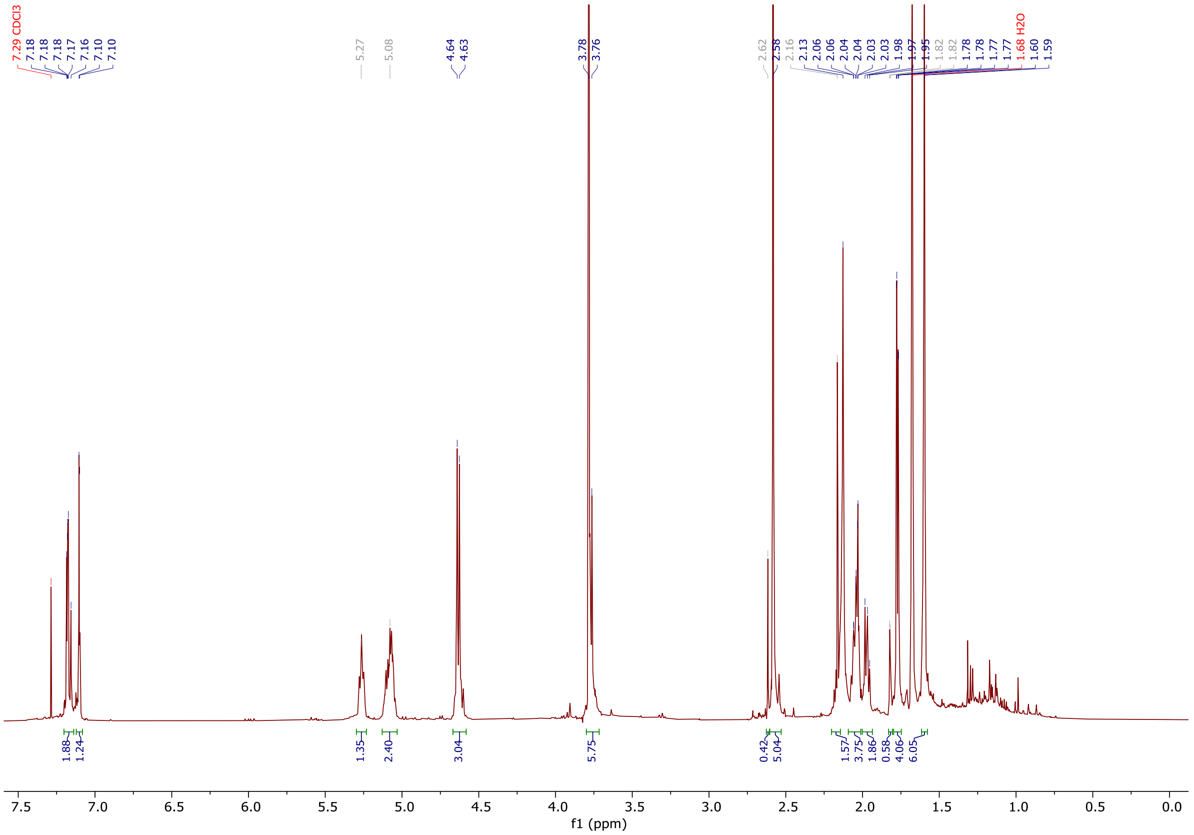

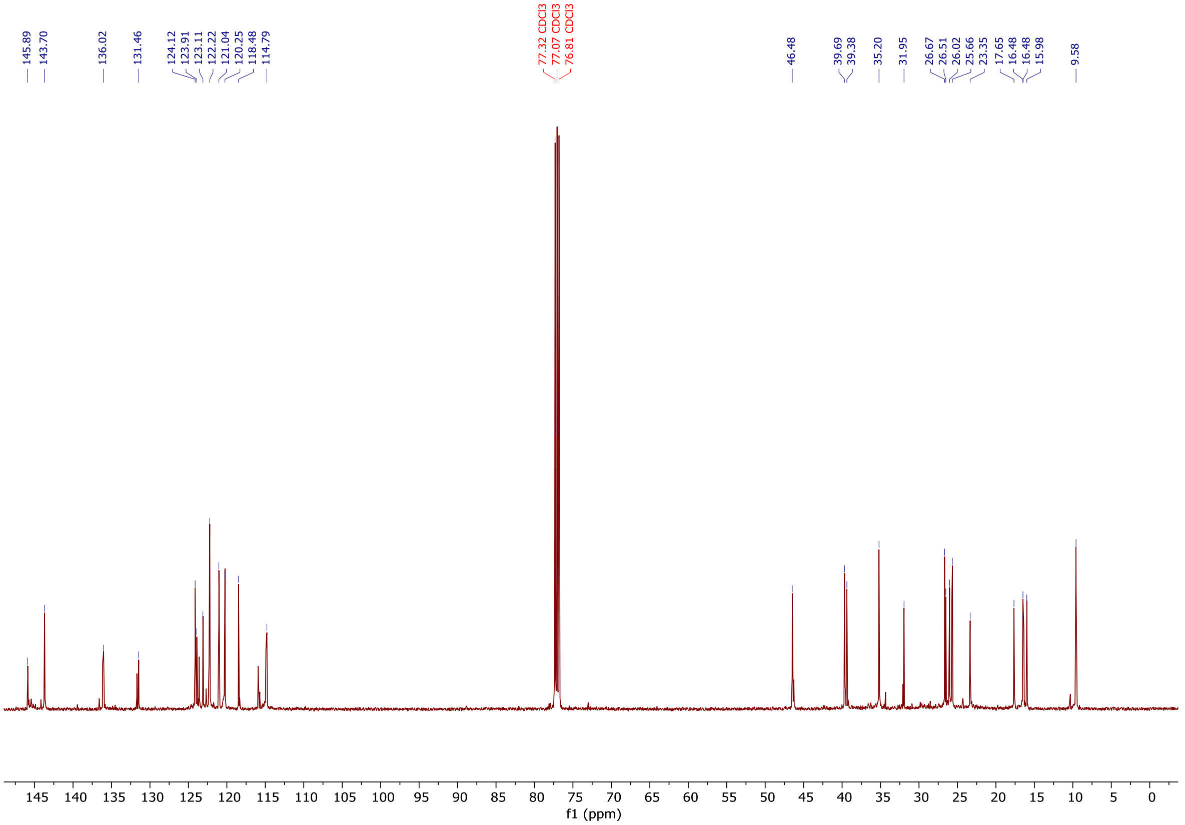

GeranylL-1

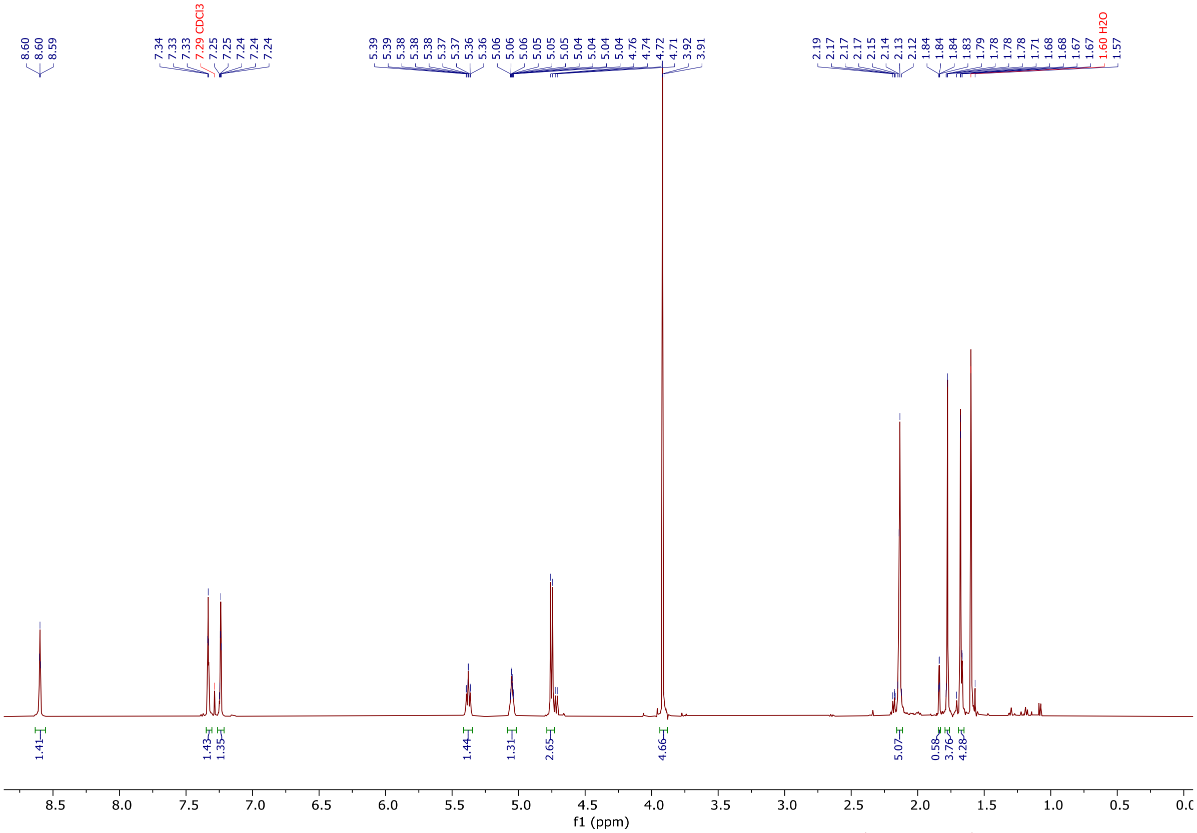

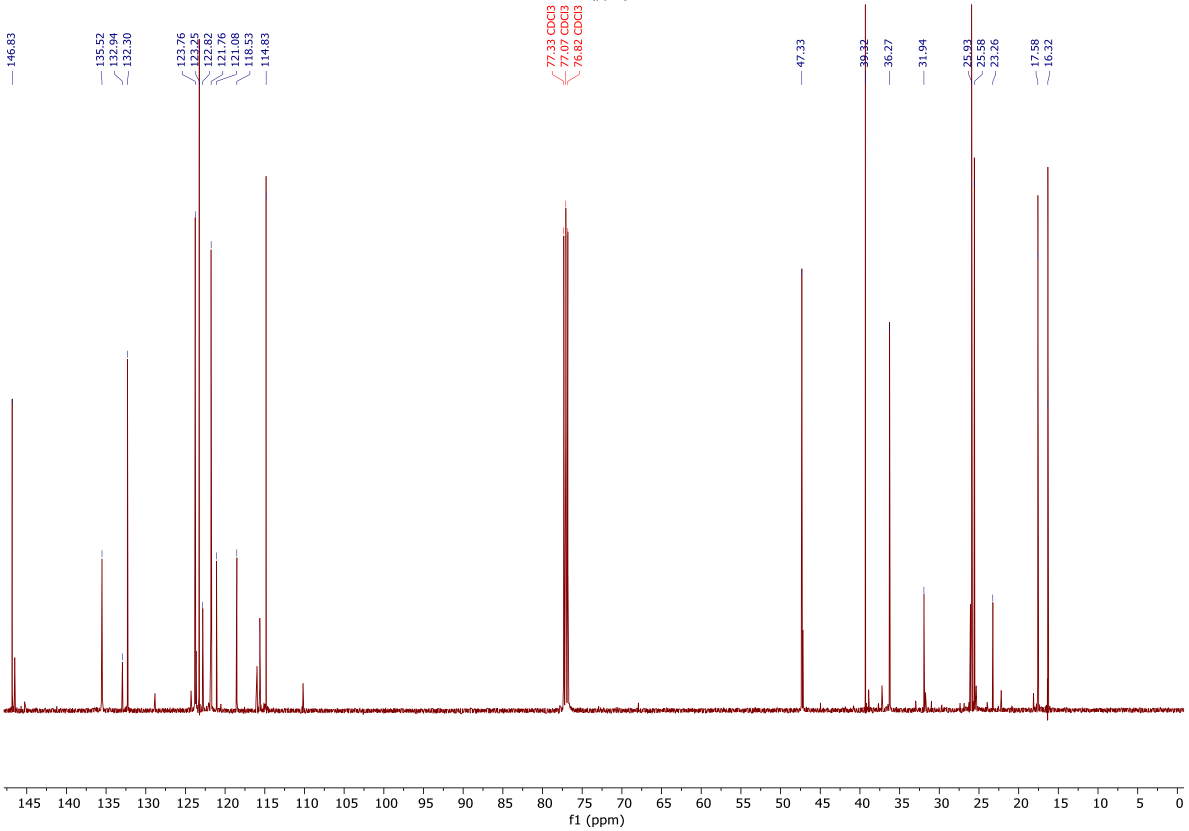

GeranylL-2

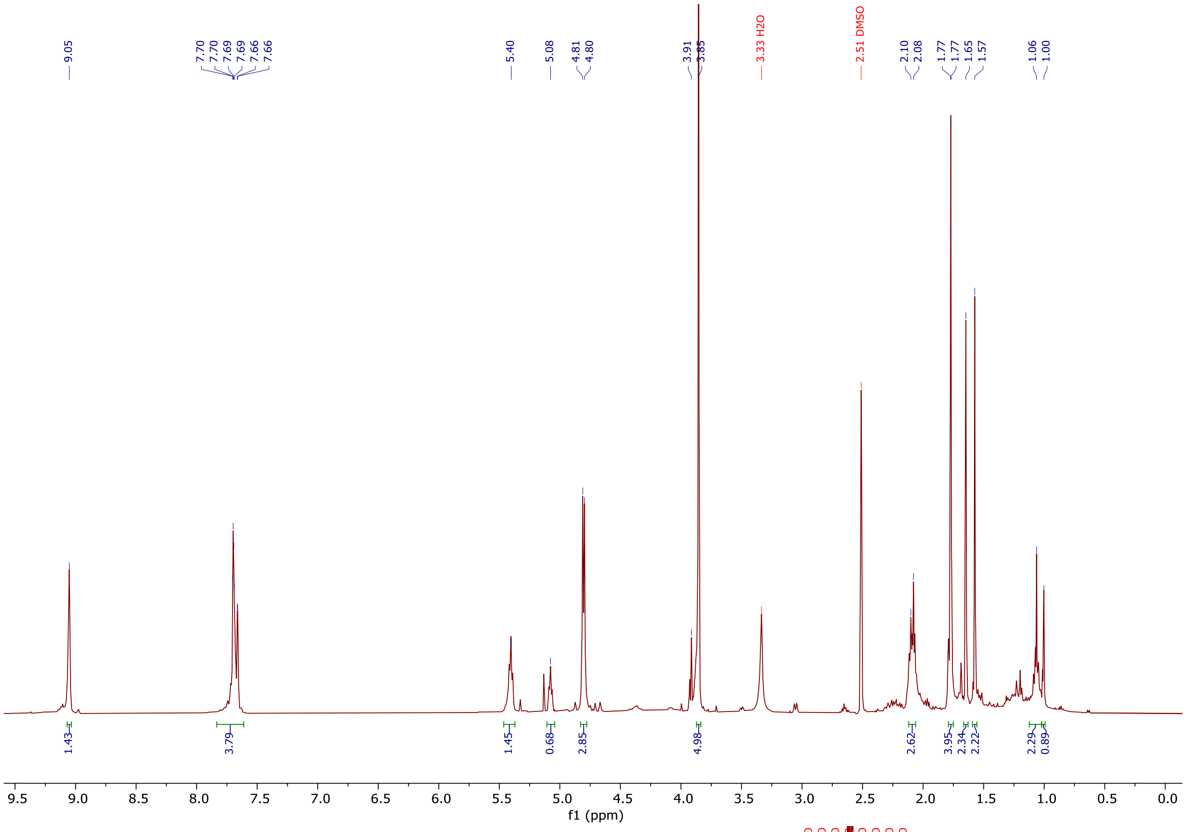

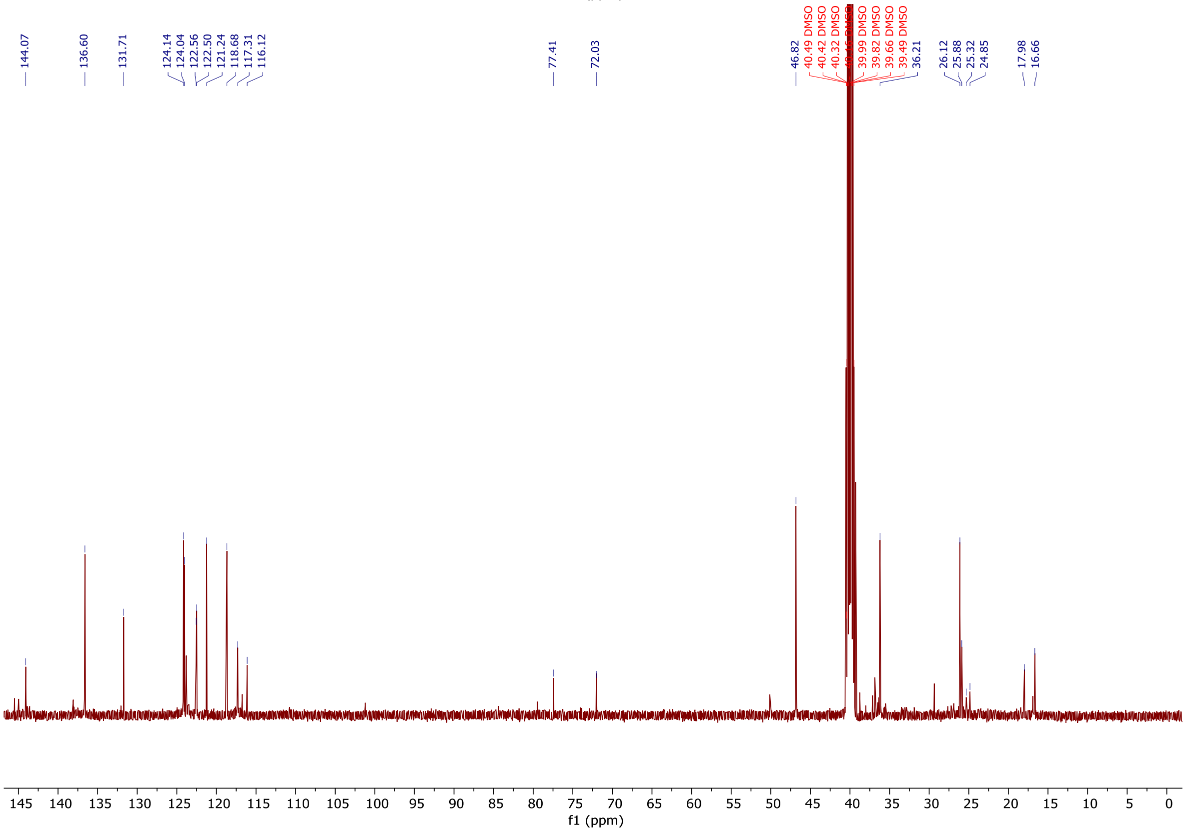

DSC and TGA Thermograms of the IL Products

PhytylL-1

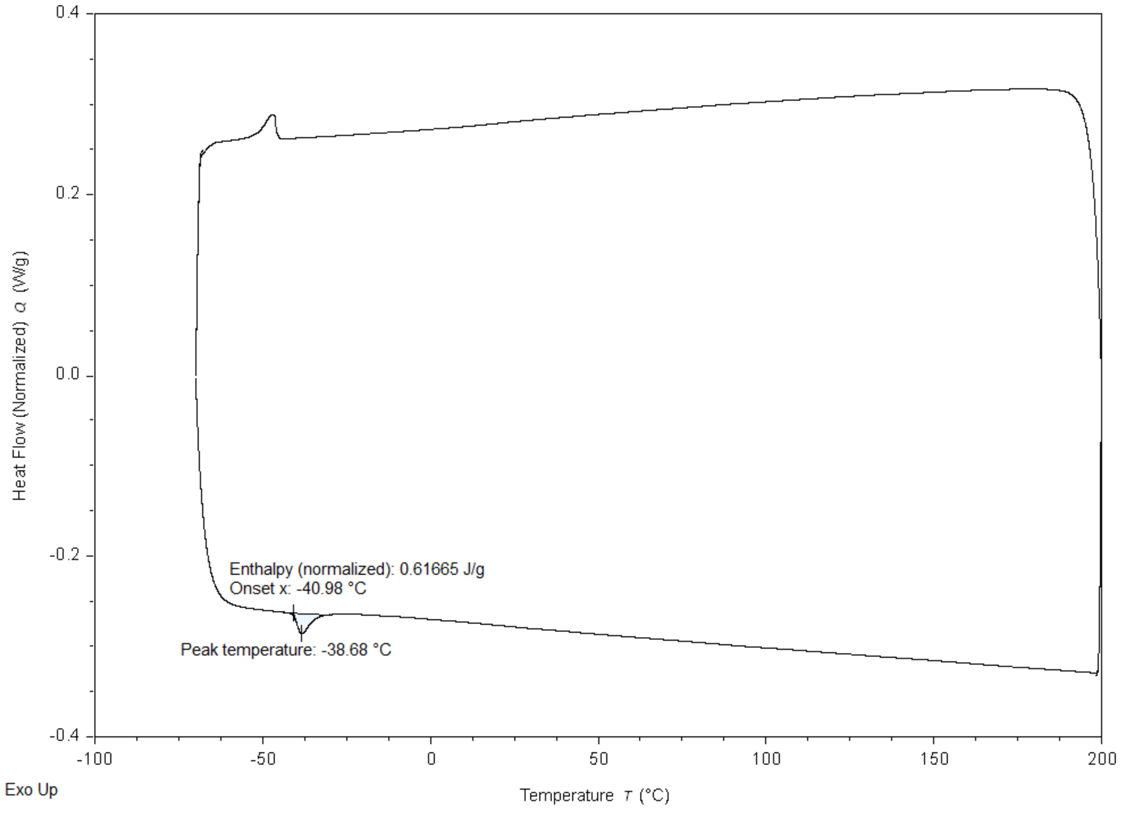

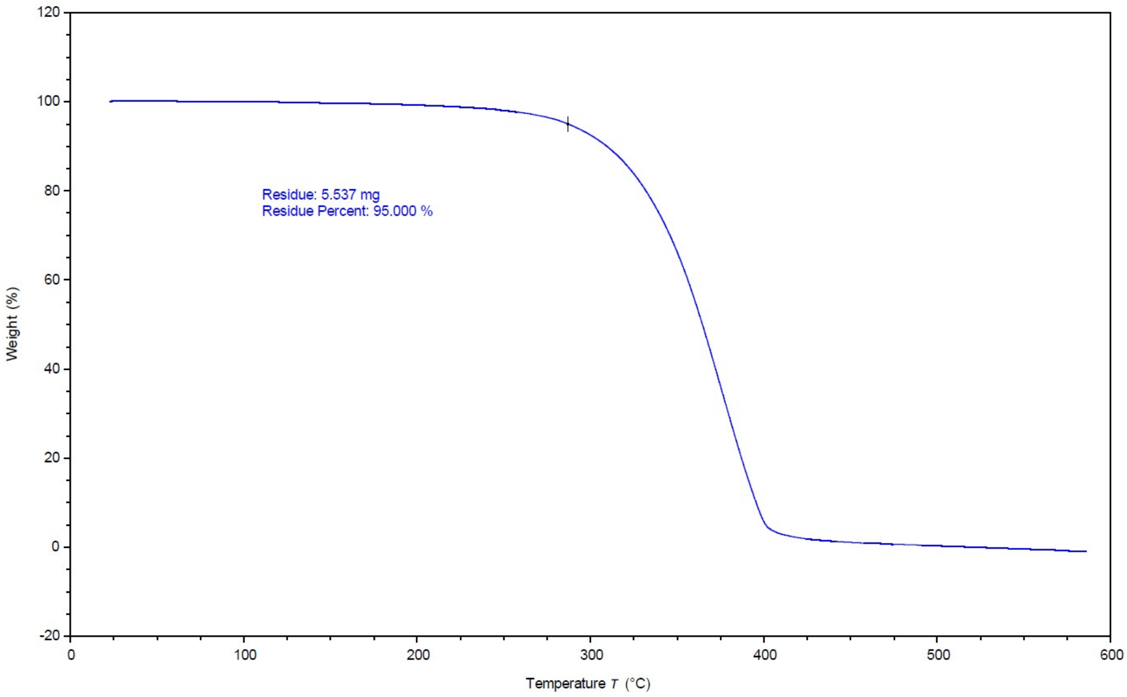

PhytyIL-2

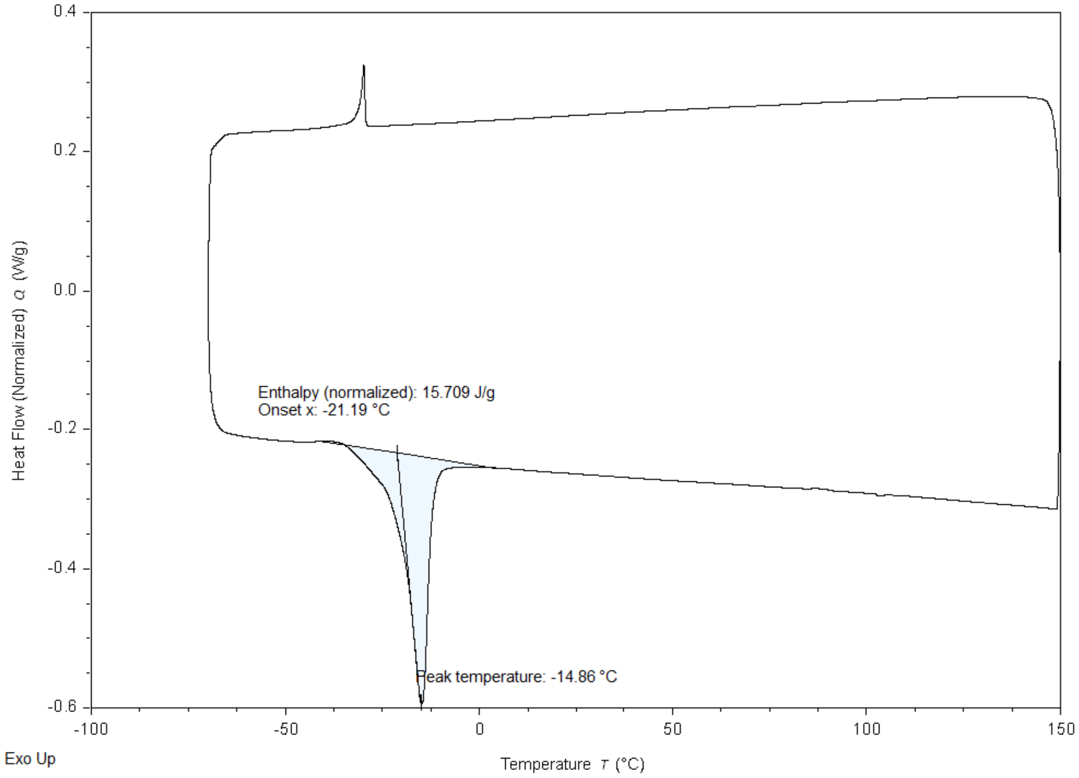
Enthalpy (normalized): 15.709 J/g
Onset x: -21.19 °C
Peak temperature: -14.86 °C
Heat Flow (Normalized) Q (W/g)
Temperature T (°C)
Exo Up
0.4
0.2
0.0
-0.2
-0.4
-0.6
-100
-50
0
50
100
150

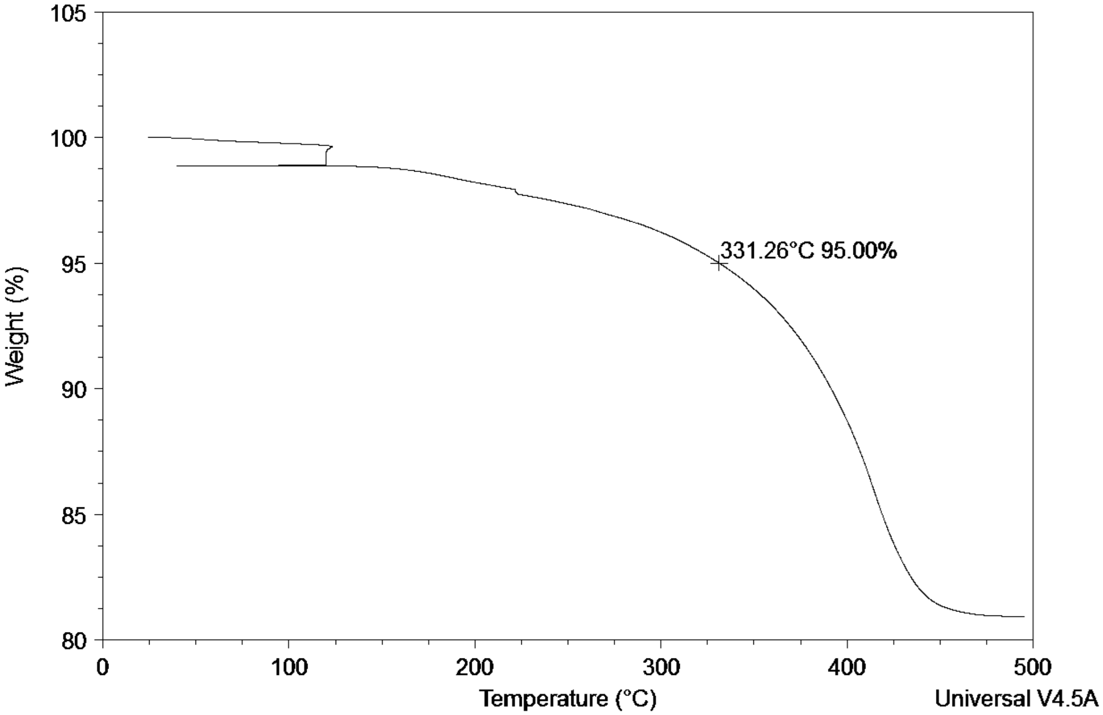
331.26°C 95.00%
Weight (%)
Temperature (°C)
Universal V4.5A
105
100
95
90
85
80
0
100
200
300
400
500

FarnesylL-1

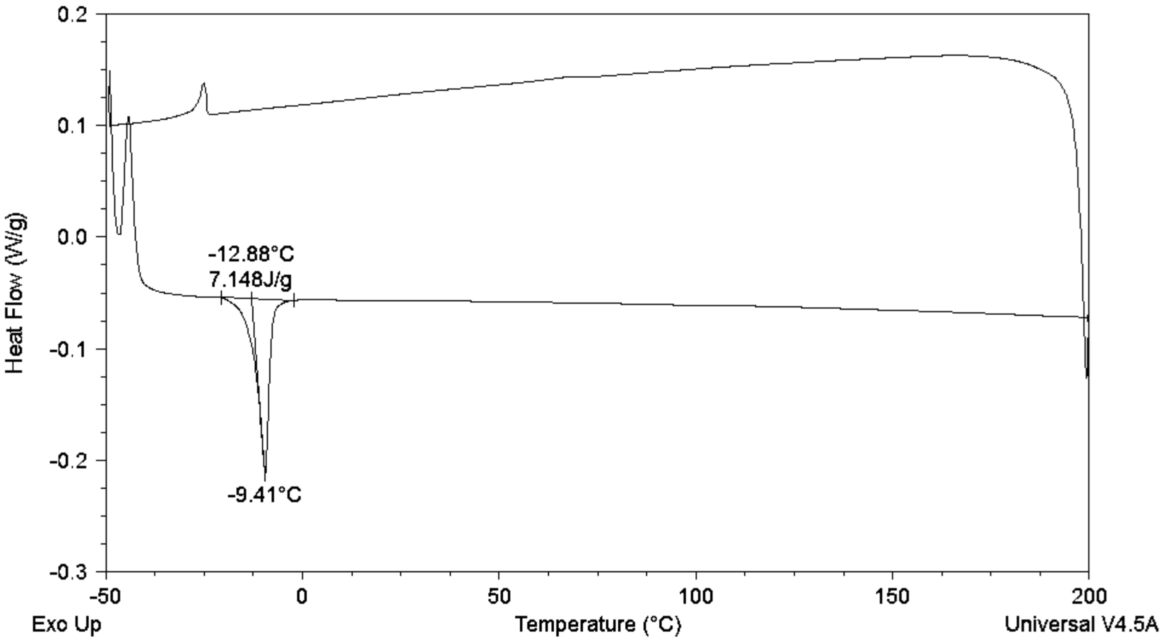
0.2
0.1
0.0
-0.1
-0.2
-0.3
Heat Flow (W/g)
-12.88°C
7.148J/g
-9.41°C
-50
0
50
100
150
200
Exo Up
Temperature (°C)
Universal V4.5A

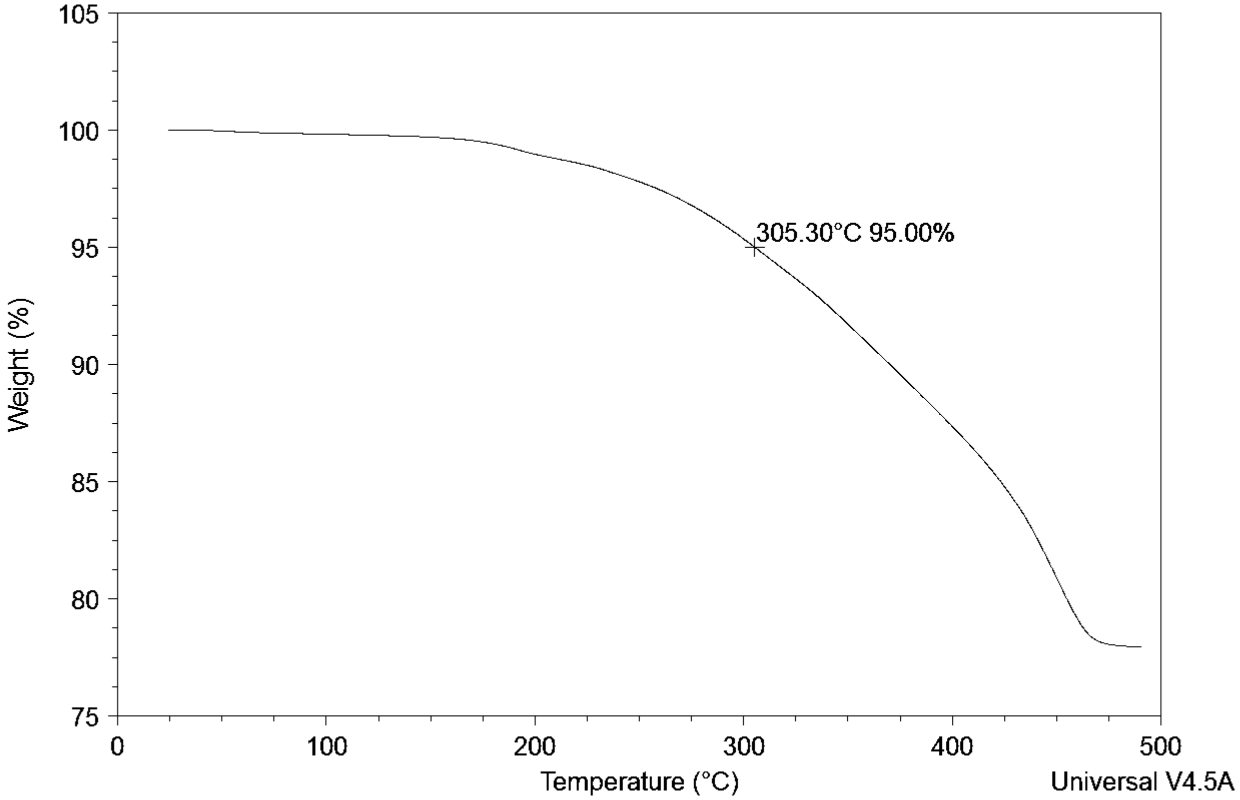
105
100
95
90
85
80
75
Weight (%)
305.30°C 95.00%
0
100
200
300
400
500
Temperature (°C)
Universal V4.5A

FarnesylL-2

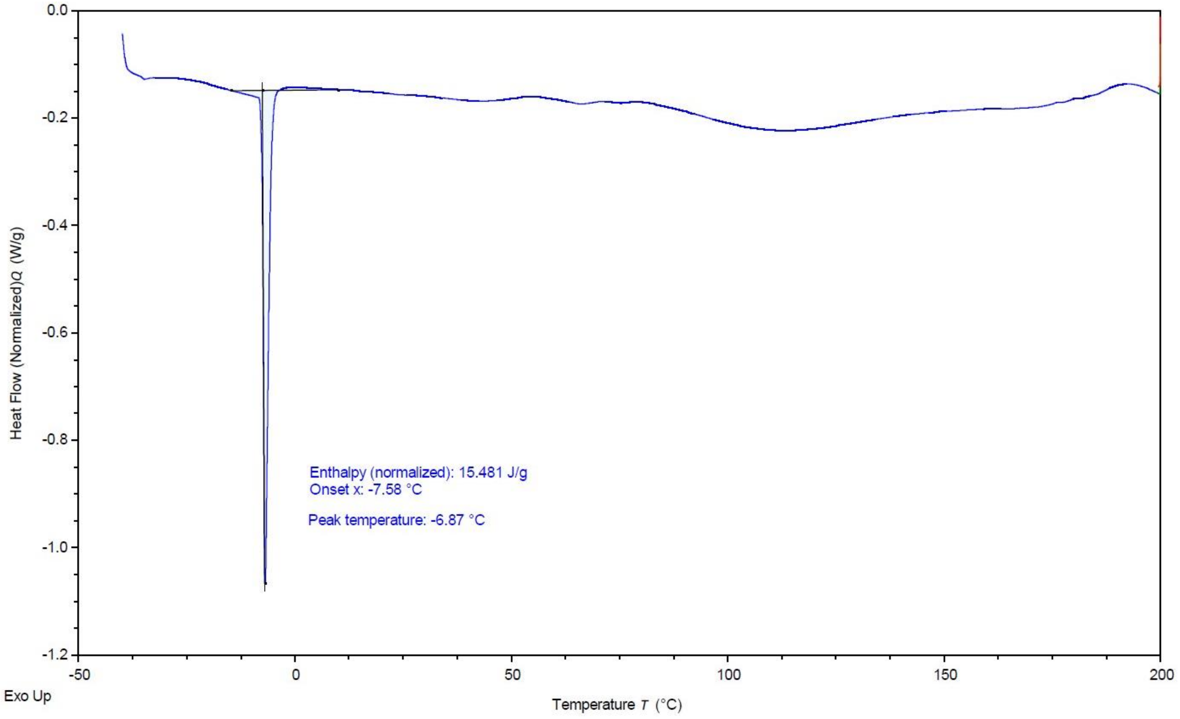

Enthalpy (normalized): 15.481 J/g
Onset x: -7.58 °C
Peak temperature: -6.87 °C
Heat Flow (Normalized)Q (W/g)
Temperature T (°C)
Exo Up
0.0
-0.2
-0.4
-0.6
-0.8
-1.0
-1.2
-50
0
50
100
150
200

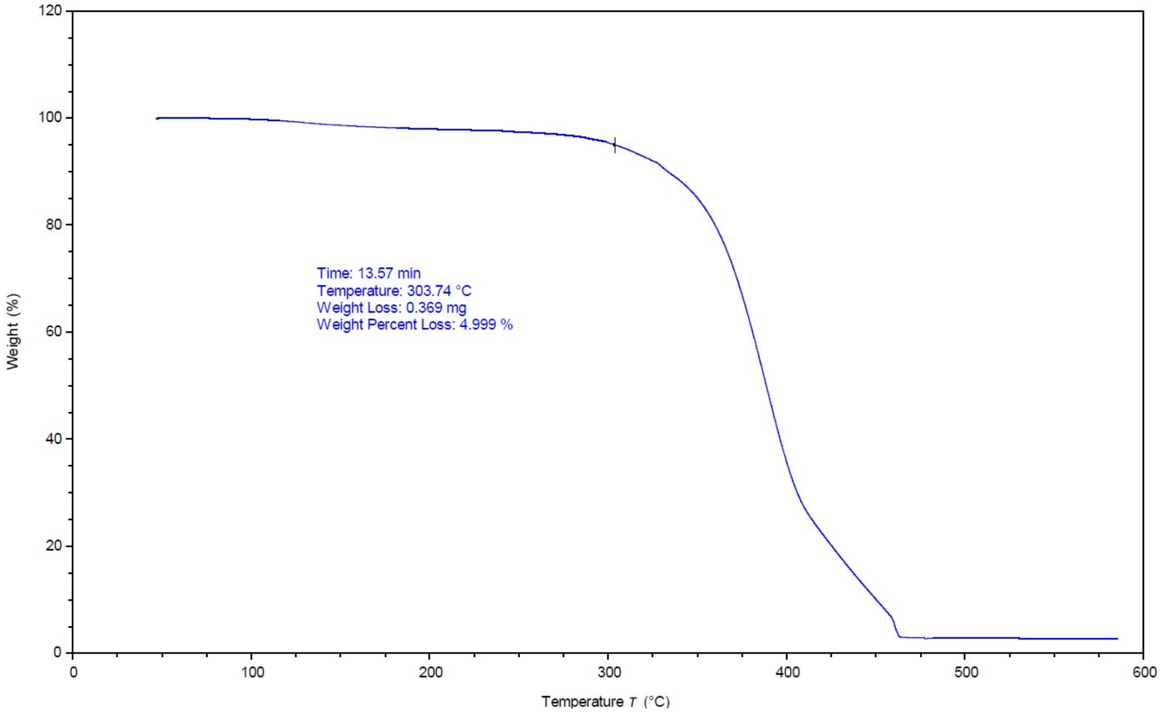

Time: 13.57 min
Temperature: 303.74 °C
Weight Loss: 0.369 mg
Weight Percent Loss: 4.999 %
Weight (%)
Temperature T (°C)
120
100
80
60
40
20
0
0
100
200
300
400
500
600

GeranylL-1

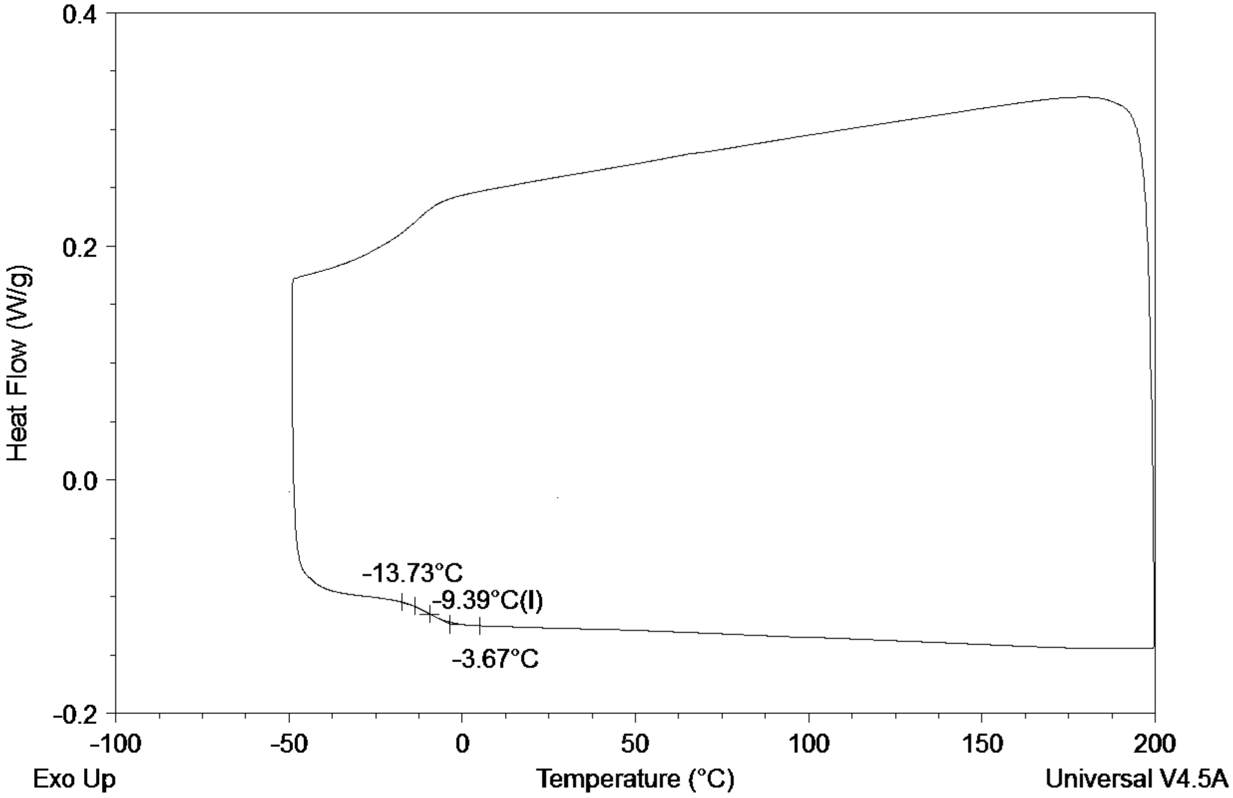

0.4
0.2
0.0
-0.2
Heat Flow (W/g)
-13.73°C
-9.39°C(I)
-3.67°C
-100
-50
0
50
100
150
200
Exo Up
Temperature (°C)
Universal V4.5A

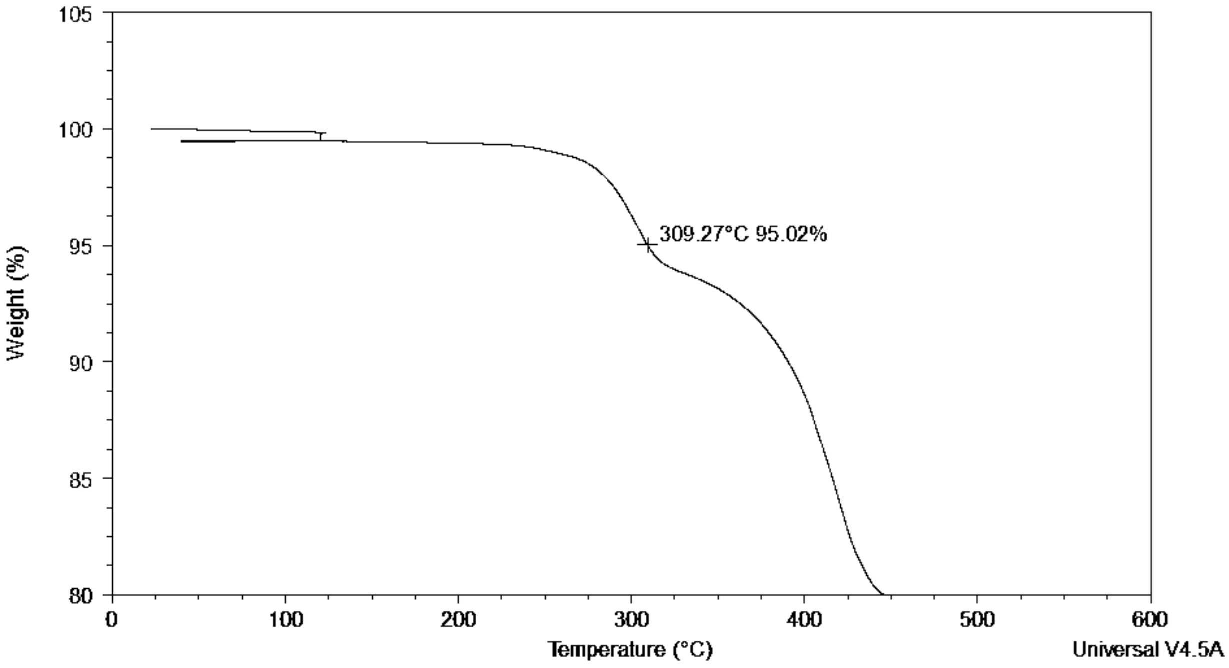

105
100
95
90
85
80
Weight (%)
309.27°C 95.02%
0
100
200
300
400
500
600
Temperature (°C)
Universal V4.5A

GeranylL-2

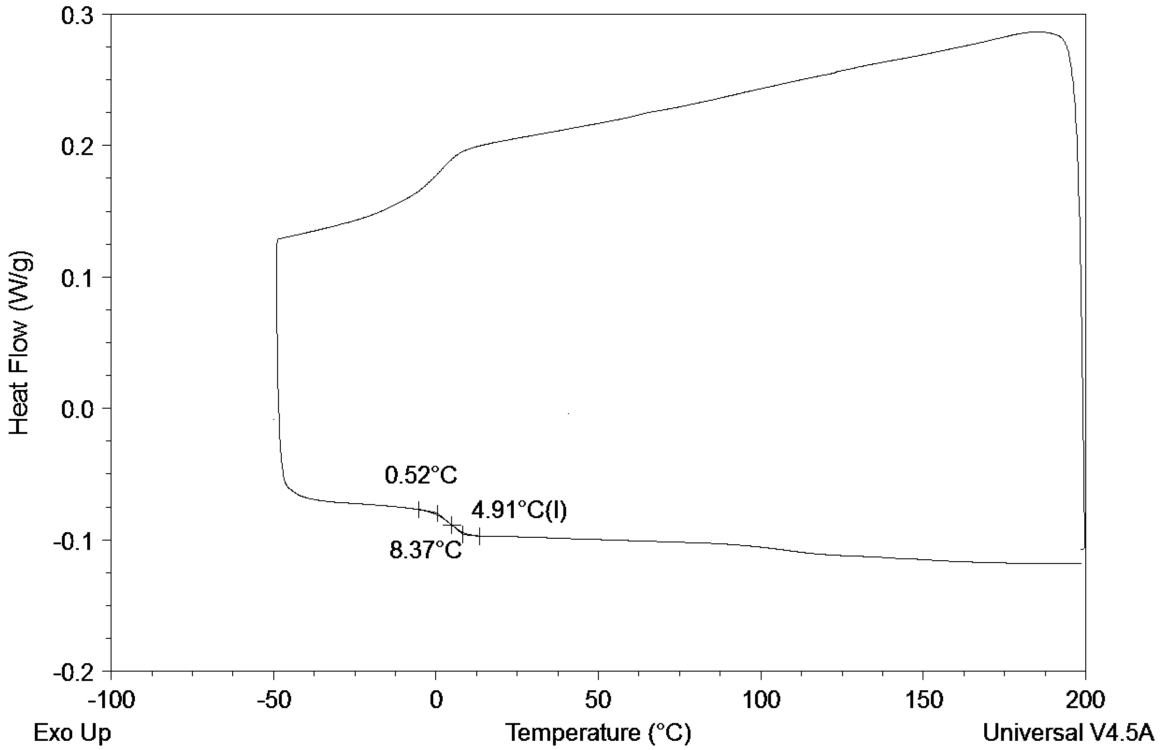

0.3
0.2
0.1
0.0
-0.1
-0.2
Heat Flow (W/g)
0.52°C
4.91°C(I)
8.37°C
-100
-50
0
50
100
150
200
Exo Up
Temperature (°C)
Universal V4.5A

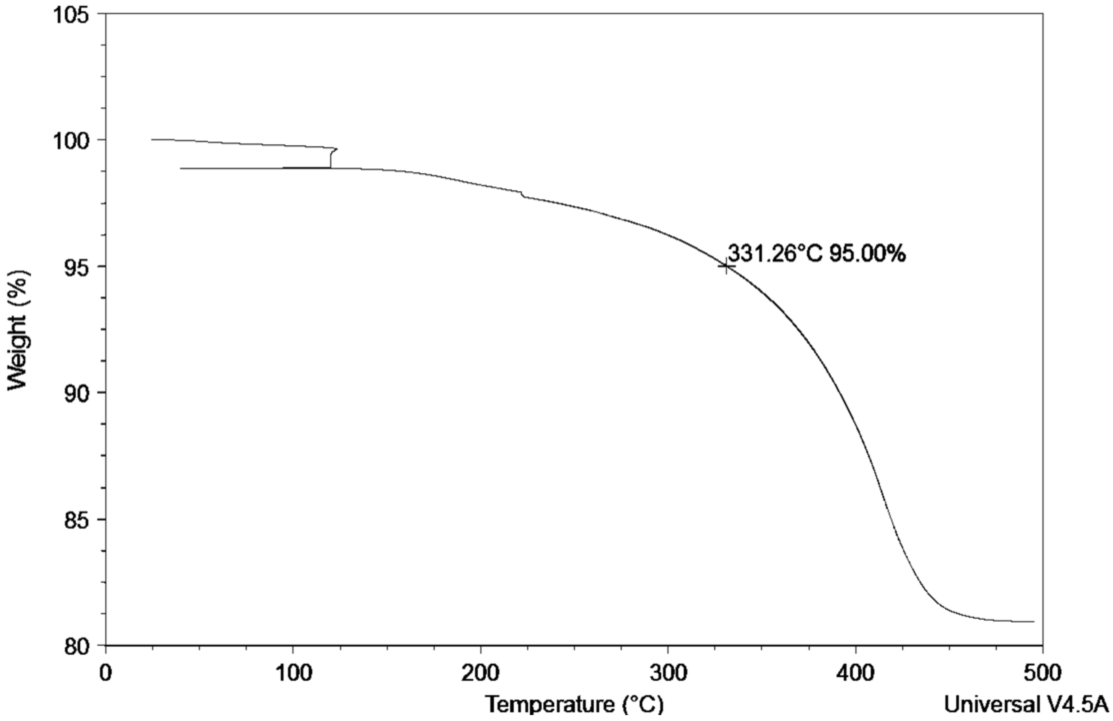

105
100
95
90
85
80
Weight (%)
331.26°C 95.00%
0
100
200
300
400
500
Temperature (°C)
Universal V4.5A
