# Supplementary material for: Lipid-Inspired Low Melting Ionic Liquids via Synergistic Cyclopropanation and Branching of Terpenoids
Source: ACS Mater Au. 2025 Jul 21;5(5):878–85. doi: 10.1021/acsmaterialsau.5c00089 (PMC12426778; doi:10.1021/acsmaterialsau.5c00089)
Supplement: Supplementary file 3 [file mg5c00089_si_003.pdf]

checkCIF (basic structural check) running

Checking for embedded fcf data in CIF ...
Found embedded fcf data in CIF. Extracting fcf data from uploaded CIF, please wait . . . .

checkCIF/PLATON (basic structural check)

Structure factors have been supplied for datablock(s) MM_IL1_BPh4_cyclopropyl_0m

THIS REPORT IS FOR GUIDANCE ONLY. IF USED AS PART OF A REVIEW PROCEDURE FOR PUBLICATION, IT SHOULD NOT REPLACE THE EXPERTISE OF AN EXPERIENCED CRYSTALLOGRAPHIC REFEREE.

No syntax errors found. CIF dictionary
Please wait while processing .... Interpreting this report

Structure factor report

Datablock: MM_IL1_BPh4_cyclopropyl_0m

Bond precision:	= 0.0000 A		Wavelength=1.54178
Cell:	a=11.2099(5)	b=37.121(2)	c=16.4619(9)
	alpha=90	beta=102.007(4)	gamma=90
Temperature:	150 K		

	Calculated	Reported
Volume	6700.3(6)	6700.2(6)
Space group	P 21/n	P 21/n
Hall group	-P 2yn	-P 2yn
Moiety formula	C24 H20 B, C15 H27 N2	C24 H20 B, C15 H27 N2
Sum formula	C39 H47.01 B N2	C39 H47 B N2
Mr	554.71	554.59
Dx,g cm-3	1.100	1.100
Z	8	8
Mu (mm-1)	0.469	0.469
F000	2400.5	2400.0
F000'	2406.38	
h,k,lmax	14,47,21	14,46,21
Nref	14645	14223
Tmin,Tmax	0.899,0.959	0.603,0.754
Tmin'	0.861	

Correction method= # Reported T Limits: Tmin=0.603 Tmax=0.754 AbsCorr = MULTI-SCAN

Data completeness= 0.971 Theta(max)= 80.247

R(reflections)= 0.0692(11040) wR2(reflections)= 0.2419(14223)

S = 1.079 Npar= 2421

The following ALERTS were generated. Each ALERT has the format
test-name_ALERT_alert-type_alert-level.
Click on the hyperlinks for more details of the test.

Alert level A

PLAT088_ALERT_3_A Poor Data / Parameter Ratio .... 5.87 Note

Author Response: Extensive disorder is present in this structure, which is well enough resolved to require modelling. An approximation of a rigid body refinement (with large numbers of chemically reasonable restraints) was used to refine the disorder and bolster data to parameter ratio.

Alert level C

PLAT041_ALERT_1_C Calc. and Reported SumFormula Strings Differ Please Check
Calc: C39 H47.01 B N2
Rep.: C39 H47 B N2
PLAT260_ALERT_2_C Large Average Ueq of Residue Including N1_1 0.128 Check
And 9 other PLAT260 Alerts
More ...
PLAT906_ALERT_3_C Large K Value in the Analysis of Variance ...... 3.872 Check
PLAT911_ALERT_3_C Missing FCF Refl Between Thmin & STh/L= 0.600 9 Report
-3 0 1, -3 1 2, -3 14 3, 5 37 3, 12 1 5, 11 0 7,
11 1 7, 11 2 7, -10 0 14,

Alert level G

FORMU01_ALERT_2_G There is a discrepancy between the atom counts in the
_chemical_formula_sum and the formula from the _atom_site* data.
Atom count from _chemical_formula_sum:C39 H47 B1 N2
Atom count from the _atom_site data: C39.00750 H47.01349 B1 N2.001
CELLZ01_ALERT_1_G Difference between formula and atom_site contents detected.
CELLZ01_ALERT_1_G ALERT: check formula stoichiometry or atom site occupancies.
From the CIF: _cell_formula_units_Z 8
From the CIF: _chemical_formula_sum C39 H47 B N2
TEST: Compare cell contents of formula and atom_site data

atom	Z*formula	cif sites	diff
C	312.00	312.06	-0.06
H	376.00	376.11	-0.11
B	8.00	8.00	0.00
N	16.00	16.01	-0.01

PLAT002_ALERT_2_G Number of Distance or Angle Restraints on AtSite 270 Note
PLAT003_ALERT_2_G Number of Uiso or U(i,j) Restrained non-H Atoms 270 Report
PLAT068_ALERT_1_G Reported F000 Differs from Calcd (or Missing)... Please Check
PLAT171_ALERT_4_G The CIF-Embedded .res File Contains EADP Records 1 Report
PLAT174_ALERT_4_G The CIF-Embedded .res File Contains FLAT Records 5 Report
PLAT175_ALERT_4_G The CIF-Embedded .res File Contains SAME Records 12 Report
PLAT176_ALERT_4_G The CIF-Embedded .res File Contains SADI Records 8 Report
PLAT178_ALERT_4_G The CIF-Embedded .res File Contains SIMU Records 1 Report
PLAT188_ALERT_3_G A Non-default SIMU Restraint Value has been used 0.0200 Report
PLAT301_ALERT_3_G Main Residue Disorder ....(Resd 1) 100% Note
And 2 other PLAT301 Alerts
More ...
PLAT302_ALERT_4_G Anion/Solvent/Minor-Residue Disorder (Resd 2) 100% Note
And 10 other PLAT302 Alerts
More ...
PLAT304_ALERT_4_G Non-Integer Number of Atoms in ..... (Resd 1) 23.67 Check
And 12 other PLAT304 Alerts
More ...
PLAT793_ALERT_4_G Model has Chirality at C7_1 (Centro SpGr) S Verify
And 3 other PLAT793 Alerts
More ...
PLAT860_ALERT_3_G Number of Least-Squares Restraints .... 14659 Note
PLAT910_ALERT_3_G Missing # of FCF Reflection(s) Below Theta(Min). 1 Note
0 2 0,
PLAT912_ALERT_4_G Missing # of FCF Reflections Above STh/L= 0.600 405 Note
PLAT941_ALERT_3_G Average HKL Measurement Multiplicity .... 3.8 Low
PLAT969_ALERT_5_G The 'Henn et al.' R-Factor-gap value .... 4.319 Note
Predicted wR2: Based on SigI**2 5.60 or SHELX Weight 22.43
PLAT992_ALERT_5_G Repd & Actual _reflns_number_gt Values Differ by 10 Check

1 **ALERT level A** = Most likely a serious problem - resolve or explain
0 **ALERT level B** = A potentially serious problem, consider carefully
13 **ALERT level C** = Check. Ensure it is not caused by an omission or oversight
49 **ALERT level G** = General information/check it is not something unexpected

4 ALERT type 1 CIF construction/syntax error, inconsistent or missing data
13 ALERT type 2 Indicator that the structure model may be wrong or deficient
10 ALERT type 3 Indicator that the structure quality may be low
34 ALERT type 4 Improvement, methodology, query or suggestion
2 ALERT type 5 Informative message, check

It is advisable to attempt to resolve as many as possible of the alerts in all categories. Often the minor alerts point to easily fixed oversights, errors and omissions in your CIF or refinement strategy, so attention to these fine details can be worthwhile. In order to resolve some of the more serious problems it may be necessary to carry out additional measurements or structure refinements. However, the purpose of your study may justify the reported deviations and the more serious of these should normally be commented upon in the discussion or experimental section of a paper or in the "special_details" fields of the CIF. checkCIF was carefully designed to identify outliers and unusual parameters, but every test has its limitations and alerts that are not important in a particular case may appear. Conversely, the absence of alerts does not guarantee there are no aspects of the results needing attention. It is up to the individual to critically assess their own results and, if necessary, seek expert advice.

Publication of your CIF in IUCr journals

A basic structural check has been run on your CIF. These basic checks will be run on all CIFs submitted for publication in IUCr journals (*Acta Crystallographica*, *Journal of Applied Crystallography*, *Journal of Synchrotron Radiation*); however, if you intend to submit to *Acta Crystallographica Section C* or *E* or *IUCrData*, you should make sure that full publication checks are run on the final version of your CIF prior to submission.

Publication of your CIF in other journals

Please refer to the *Notes for Authors* of the relevant journal for any special instructions relating to CIF submission.

PLATON version of 13/05/2024; check.def file version of 04/05/2024

Datablock MM_IL1_BPh4_cyclopropyl_0m - ellipsoid plot

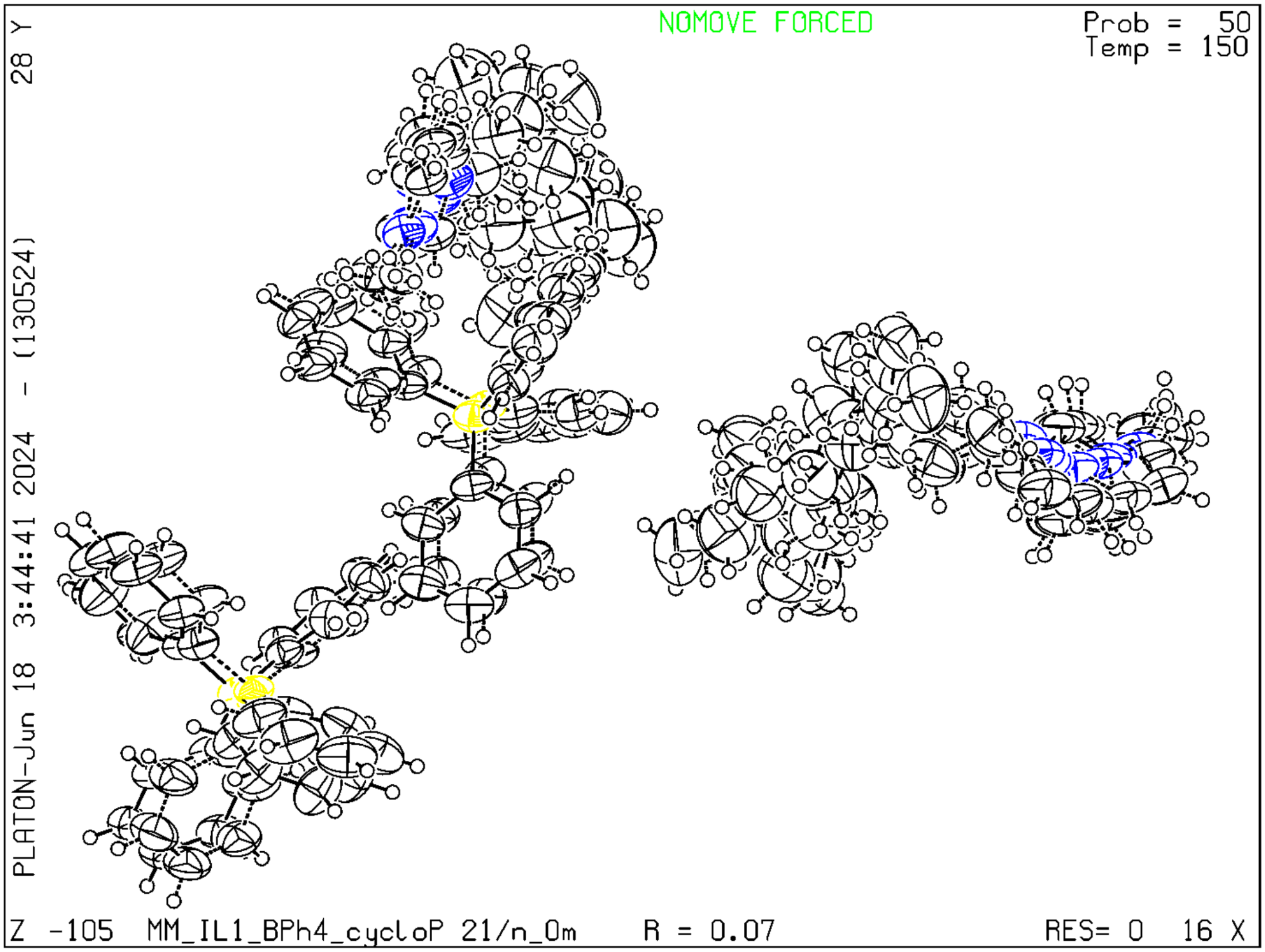

Download CIF editor (publCIF) from the IUCr

Download CIF editor (enCIFer) from the CCDC
Test a new CIF entry
